# Prediction of mitochondrial genome-wide variation through sequencing of mitochondrion-enriched extracts

**DOI:** 10.1038/s41598-020-76088-0

**Published:** 2020-11-05

**Authors:** Kelsey E. Fisher, Steven P. Bradbury, Brad S. Coates

**Affiliations:** 1 Department of Entomology, Iowa State University, Ames, IA 50011 USA; 2 Department of Natural Resource Ecology and Management, Iowa State University, Ames, IA 50011 USA; 3 Department of Agriculture, Agriculture Research Station, Corn Insects and Crop Genetics Research Unit, Ames, IA 50011 USA

**Keywords:** Biological techniques, Computational biology and bioinformatics, Genetics

## Abstract

Although mitochondrial DNA (mtDNA) haplotype variation is often applied for estimating population dynamics and phylogenetic relationships, economical and generalized methods for entire mtDNA genome enrichment prior to high-throughput sequencing are not readily available. This study demonstrates the utility of differential centrifugation to enrich for mitochondrion within cell extracts prior to DNA extraction, short-read sequencing, and assembly using exemplars from eight maternal lineages of the insect species, *Ostrinia nubilalis*. Compared to controls, enriched extracts showed a significant mean increase of 48.2- and 86.1-fold in mtDNA based on quantitative PCR, and proportion of subsequent short sequence reads that aligned to the *O. nubilalis* reference mitochondrial genome, respectively. Compared to the reference genome, our de novo assembled *O. nubilalis* mitochondrial genomes contained 82 intraspecific substitution and insertion/deletion mutations, and provided evidence for correction of mis-annotated 28 C-terminal residues within the NADH dehydrogenase subunit 4. Comparison to a more recent *O. nubilalis* mtDNA assembly from unenriched short-read data analogously showed 77 variant sites. Twenty-eight variant positions, and a triplet ATT codon (Ile) insertion within ATP synthase subunit 8, were unique within our assemblies. This study provides a generalizable pipeline for whole mitochondrial genome sequence acquisition adaptable to applications across a range of taxa.

## Introduction

Insect mitochondrial genomes are relatively small (range: 14 to 40 kbp) and encode 13 protein-coding genes, 22 transfer RNAs, and two ribosomal RNAs^1^ in a highly conserved order and orientation^2^. Due to higher rates of sequence evolution^3,4^, low recombination rates^5^, and lower effective population size of the maternally-inherited molecule compared to biparental nuclear loci, mitochondrial DNA (mtDNA) is often used to predict evolutionary relationships and estimate species divergence times^6–11^, albeit for shallow time scales due to accumulating effects of homoplasy^12^. Additionally, intraspecies variation can shed light on population genetics, demographics, and female dispersal^13^. Although the majority of studies are based on haplotype variation estimated from a relatively small number of mitochondrial genes (typically one or a few), whole mtDNA genome analyses offer advantages for phylogenetic, population genetic, and functional genomic studies^14–16^.

*Ostrinia nubilalis* is a lepidopteran pest that feeds on and causes yield loss to cultivated maize and other crops across its native range of Europe and western Asia, as well as within introduced and invasive areas of North America^17^. Feeding damage was reduced in the United States following the widespread adoption of transgenic maize encoding insecticidal *Bacillus thuringiensis* (Bt) toxins^18^, yet *O. nubilalis* remains a model for the study of sympatric population divergence and incipient species formation^19^. Although low-levels of mtDNA cytochrome c oxidase subunit I (*cox*I) variation were significant between sympatric and allopatric *O. nubilalis* ecotypes differing in the number of annual reproductive generations^20^, mtDNA variation is generally considered to be low and uninformative within this species^21,22^. This is in stark contrast to the greater inter-individual (haplotype) variation reported in the related species, *O. furnacalis*^23,24^, and other species of Lepidoptera^25,26^. The evolutionary rate of nucleotide change varies across mitochondrial genome regions for animals^27^, including insects^7^, indicating studies based on short mtDNA gene fragments may be skewed by genome sampling ascertainment bias^14^.

The number of sequenced whole mitochondrial genomes has greatly increased in recent years^28^. This increase has facilitated mitochondrial genome assemblies directly from high-throughput short-read sequencing of shotgun total genomic libraries^8^, or libraries enriched for mtDNA using commercial miniprep-^29^, rolling circle-^30^, or probe-based methods^31^. Regardless, many of these methods co-enrich nuclear-fragments with integrated mitochondrial DNA fragments (NUMTs)^32–36^, or arguably require expensive commercial reagents. The current study establishes a relatively rapid, less expensive, and portable enrichment method, which, when applied to samples prior to high-throughput DNA sequence library construction and sequencing, allows for downstream assessment of full mtDNA genome variation. Differential centrifugation, adapted from prior methods^37^, is used to obtain mitochondrial- and nuclear-enriched fractions from *Ostrinia nubilalis* thorax homogenates. This enrichment method, which minimized the number of reads derived from NUMTs, may be especially useful for assembly of novel reference mitochondrial genomes. The de novo mitochondrial genome assembly and annotation are described, and we report two major differences compared to the reference assemblies (AF442957.1 and MN793322.1). This study demonstrates the utility of mitochondrion enrichments within a full mtDNA genome sequencing protocol, and how detected variation can be used in population and phylogenetic studies.

## Results

### Optimization of differential centrifugation and DNA extraction

**Figure 1:**
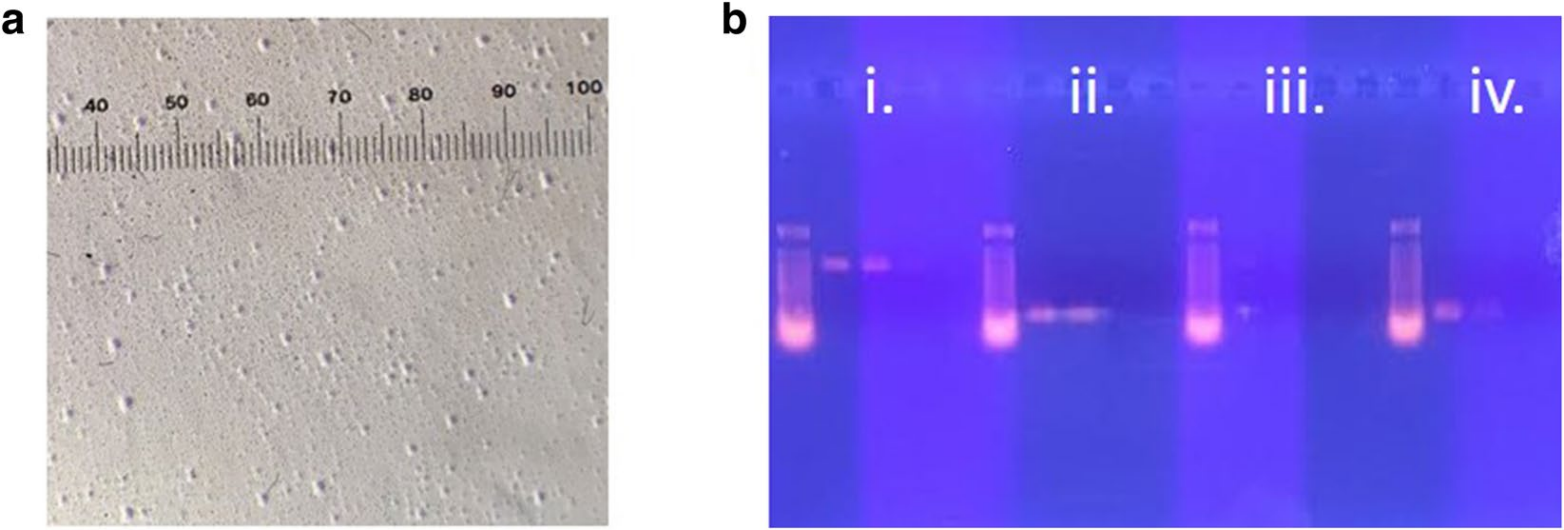
Representative example of differentially fractionated samples of *Ostrinia nubilalis* homogenized thorax tissue to acquire nuclear and mitochondrial components at 6000 and 13,000 rcf, respectively. Janus Green B staining of the mitochondrial fraction indicated abundant mitochondria (**a**). Gel electrophoresis of serial-diluted DNA extracted from the mitochondrial and nuclear fractions amplified in duplicate with mitochondrial primers (HCO/LCO;^59^) and nuclear primers (On*apn*1-F and -R;^46^) (**b**). Mitochondrial fraction (13,000 rcf) was PCR amplified with mitochondrial primers (i) and nuclear primers (ii). Nuclear fraction (6000 rcf) was PCR amplified with mitochondrial primers (iii) and nuclear primers (iv). Although nuclear DNA was present in both the mitochondrial and the nuclear fractions, mtDNA was only present in the mitochondrial fraction.

Following initial centrifugation at 1000 relative centrifugation force (rcf) to remove cell debris, a second centrifugation at 6000 rcf optimally separated intact nuclei from mitochondria within thorax cellular homogenates. This combination resulted in the greatest sedimentation of nuclei (chromosomal DNA) and retention of mitochondria (mtDNA) in the supernatant. Subsequent centrifugation of the supernatant at 13,000 rcf provided a precipitate rich in mitochondria, as estimated by Janus Green B stained organelles (Fig. 1a). Enrichment was also demonstrated by the ratio of amplified fragment intensities by semi-quantitative PCR of mitochondrial (*cox*I) compared to the *apn*1 nuclear target gene (Fig. 1b, S1). By comparison, DNA extracts centrifuged at 2000 and 4000 rcf resulted in either no or inconsistent nuclear amplification, respectively, indicating ineffective separation of nuclei and mitochondria (data not shown). Mitochondrial *cox*I amplification was also inconsistent or non-existent within DNA extracts obtained from fractions pelleted at 2000 and 4000 rcf. Nuclear and mitochondrial target genes were both PCR amplified from fractions collected at 8000 rcf, suggesting co-precipitation of both organelles (data not shown).

### Quantification of mitochondrion enrichment at optimized parameters

**Figure 2:**
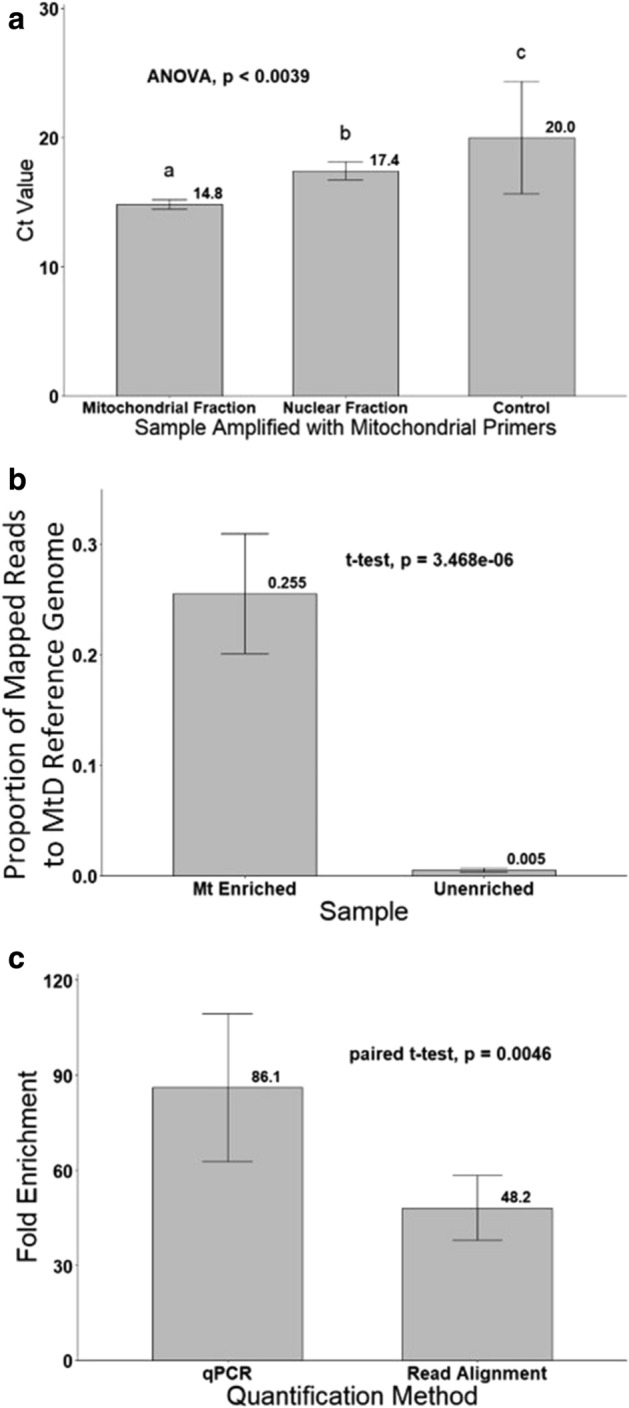
Estimates of mitochondrion enrichment of *Ostrinia nubilalis* from homogenized thorax tissue through differential fractionation prior to DNA extraction. (**a**) Significantly lower C_T_ values were estimated by real-time PCR among mitochondrial fractions (14.83 ± 0.37) amplified with mitochondrial cytochrome c oxidase subunit I (*cox*I) primers (OnCOXIrtS and OnCOXIrtA) compared to the nuclear fractions (17.43 ± 0.67) or unenriched controls (20.0 ± 4.34) (ANOVA, *p* < 0.001). Different letters above bars represent a significant difference. (**b**) Significant increase in the ratio of reads from enriched mitochondrion samples (0.255 ± 0.054) that aligned to the *O. nubilalis* mitochondrial genome reference (AF442957.1) compared to unenriched controls (0.0005 ± 0.0017; two-sample t-test, *p* < 0.0001) (**c**) Difference in fold-enrichment estimates for mitochondrion fractions estimated by with mitochondrial target amplicons estimate enrichment by real-time quantitative PCR (mean 86.1 ± 22.3) and read alignment (mean 48.2 ± 10.2). Although significant (paired t-test, *p* = 0.0046), both estimation methods indicate consistent trends in enrichment.

Using mitochondrial *cox*I primers to amplify mitochondrial fractions as template with real-time quantitative PCR (qPCR) resulted in an estimated mean C_T_ (14.83 ± 0.09) that was significantly lower compared to that estimated from nuclear fractions (17.43 ± 0.17) or unenriched controls (17.54 ± 0.41) (Fig. 2a; − 5.069 < Z > 3.205; df = 2, 34; *p* < 0.0039; Table S1). By comparison, when mitochondrial fractions, nuclear fractions, and unenriched control samples were amplified with nuclear *apn*1 primers, the mean C_T_ values were 30.25 ± 0.21, 24.56 ± 0.29, and 26.58 ± 0.21, respectively, and were significantly different from each other (− 6.905 < Z > 3.763; df = 2, 34; *p* < 0.0005; Table S1).

A mean of 3,047,025 (± 162,751) reads was obtained across eight individual mitochondrion-enriched Illumina MiSeq libraries (BioProject: PRJNA604593; accessions SRR5182721-SRR5182724; Table S1). Among these reads, a mean of 781,200 (± 77,773) aligned to the *O. nubilalis* reference mitochondrial genome sequence (AF442957.1) with a *Q*-score > 30 (mean ~ 26.7 ± 2.0% alignment rate). The unenriched controls had a mean of 183,349 (± 34,965) mapped reads with *Q*-score > 30 from among a mean of 35,319,000 (± 4,800,041) total reads (~ 0.6 ± 0.1% mean alignment rate). The increase in the proportions of reads that aligned to the mitochondrial reference from libraries generated from mitochondrion enriched (~ 26.7%) compared to unenriched libraries (~ 0.6%) was significant (Fig. 2b; t = 13.065; df = 1, 11; *p* < 0.0001; Table S1).

The resulting ratio of C_T_ values estimated from real-time qPCR amplification of mitochondrial-enriched extracts compared to the mean C_T_ of unenriched extracts showed an estimated mean 86.1 ± 8.25-fold enrichment (Fig. 2c). Based on the ratio of aligned Illumina MiSeq reads from enriched to unenriched libraries, a mean enrichment of 48.2 ± 3.6-fold was estimated. Although there was a significant difference in the estimated fold-enrichments based on real-time qPCR compared to Illumina MiSeq read alignment (Fig. 2c, t = − 4.0958; df = 1, 7; *p* = 0.0046), a ≥ 29.8-fold mtDNA enrichment was predicted among all samples with both estimation methods (Table S1).

### De novo mitochondrial genome assembly, annotation and variant prediction

Mitochondrion-enriched short read library data were de novo assembled, annotated from query results of *O. nubilalis* mitochondrial genome RefSeq models using the BLASTn algorithm, and submitted to NCBI GenBank (MT492030.1-MT492037.1; Table 1). Each assembly was annotated with 13 protein-coding genes (PCGs), 22 tRNAs, and two rRNAs typical of animal mtDNA genomes. Based on the invertebrate mitochondrial code, all PCG translations were initiated with Met start codon (ATA or ATG), with the exception of *cox*I that had an atypical Arg (CGA). Stop codons, TAA or TAG, were predicted for all PCGs, with the exception of *cox*II that ends in a single T nucleotide that putatively forms a functional TAA stop codon following polyadenylation during transcript maturation.

**Table 1:** *Ostrinia nubilalis* mitochondrial genome sequence assembly accessions.

Accession	Description	Assembly Len	Citation	Read type/platform
AF442957.1	RefSeq NC_003367.1	14,535 bp	Coates et al. 2004^20^	Sanger/ABI3700
MN792233.1	Yili, Xinjiang Region	15,248 bp	Zhou et al. 2020^35^	Illumina/HiSeq
MT492030.1	F1_Family_ID_02	14,838 bp	This study	Illumina/MiSeq
MT492031.1	F1_Family_ID_03	14,817 bp	This study	Illumina/MiSeq
MT492032.1	F1_Family_ID_04	14,691 bp	This study	Illumina/MiSeq
MT492033.1	F1_Family_ID_06	14,621 bp	This study	Illumina/MiSeq
MT492034.1	F1_Family_ID_07	14,622 bp	This study	Illumina/MiSeq
MT492035.1	F1_Family_ID_11	15,082 bp	This study	Illumina/MiSeq
MT492036.1	F1_Family_ID_12	14,809 bp	This study	Illumina/MiSeq
MT492037.1	F1_Family_ID_18	15,064 bp	This study	Illumina/MiSeq

**Figure 3:**
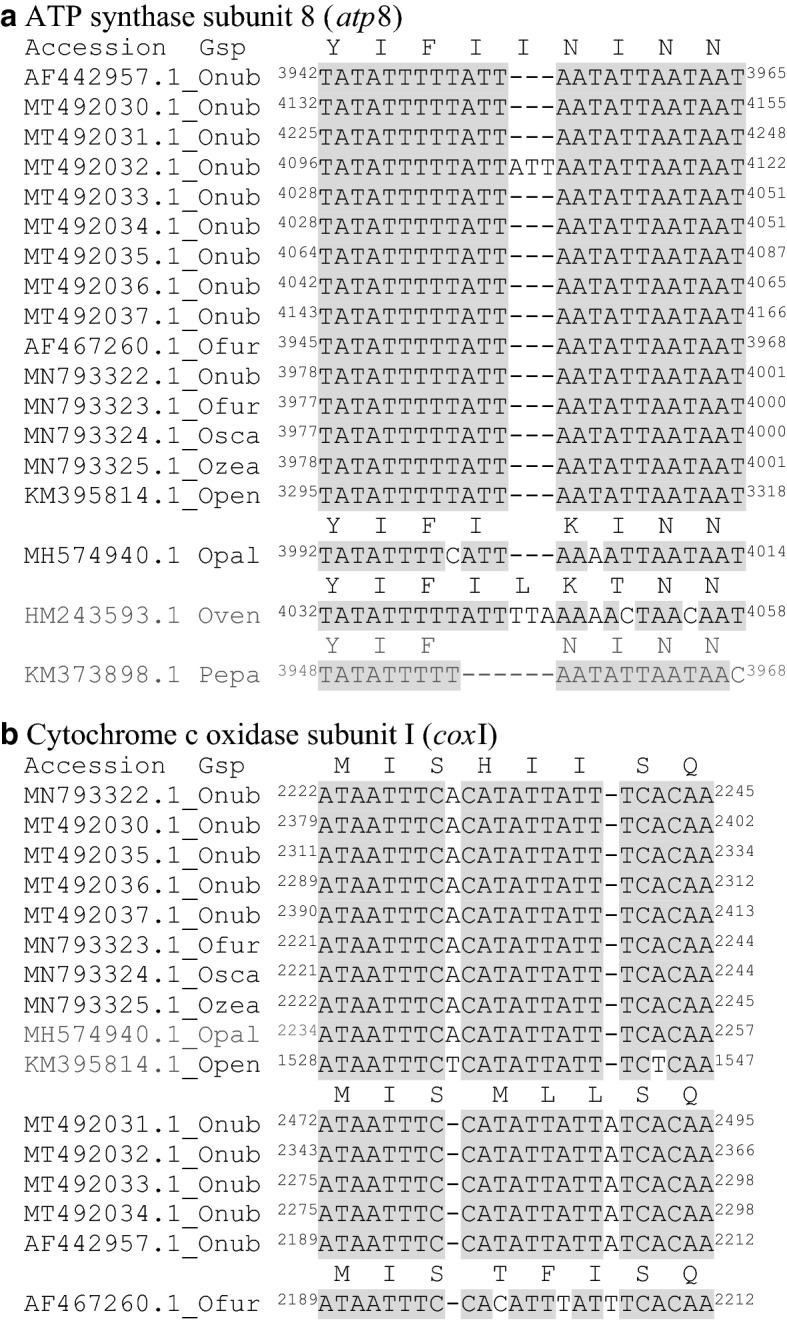
In-frame and frameshift mutations predicted among assembled *Ostrinia* mitochondrial genomes. (**a**) Multiple sequence alignment of an ATP synthase subunit 8 (atp8) gene region showing a putative in-frame ATT nucleotide (Isoleucine, I) duplication within F1_Family_ID_07 (MT492034.1) compared to other *Ostrinia*. (**b**) Frameshift impacting three codons of the COI protein-coding sequence caused by flanking compensatory insertion/deletion mutations that return sequences to the same downstream frame. Genus species (Gsp) abbreviations: Onub, *O. nubilalis*; Ofur, *O. furnacalis*; Osca, *O. scapulalis*; Ozea, *O. zealis*; Opal, *O. palustralis*. Open, *O. penitalis*; Oven, *Ochlodes venata* (Lepidoptera: Hesperiidae); Pepa, *Parnassius epaphus* (Lepidoptera: Papilionidae)*.* Conserved sites highlighted in grey.

Our assemblies were compared to the Sanger sequence-based reference *O. nubilalis* mitochondrial genome assembly, AF442957.1^38^ (RefSeq NC_003367.1), and a recent Illumina short shotgun read-based assembly^39^. For the former comparison, a total of 82 mutations were predicted among our de novo assemblies (MT492030.1–MT492037.1; Table 1) and the reference AF442957.1 (RefSeq NC_003367.1; Table S2). Because each of our de novo mitochondrial genomes had a different start position due to starts at random seeds, homologous nucleotide sites with respect to the reference were defined within each accession (Table S3). Among these predicted variants, 58 (69.8%) were within protein-coding sequences (CDS; *cox*I, *cox*II, *atp*8, *atp*6, *cox*III, *nd*3, *nd*5, *nd*4, *nd*4L, *cytb*, or *nd*1; Table S2), for which 35 generated nonsynonymous (amino acid) changes (60.3% of CDS mutations; 42.2% of all mutations). Twenty-two of the remaining CDS mutations were synonymous (silent; 26.8%). In addition, an in-frame insertion of an intact triplet ATT (Ile) codon was predicted within *atp*8 from F_1__Family_ID_04 (accession MT492032.1; Fig. 3a). This inserted ATT codon was not present in any other previously sequenced *Ostrinia* mitochondrial genome, but was found in the *atp*8 CDS of the butterfly species, *Ochlodes venata* (Lepidoptera: Hesperiidae). Moreover, the two adjacent Ile residues within this region of ATP8 showed variable presence among species of Lepidoptera, with both omitted from *Parnassius epaphus* (Lepidoptera: Papilionidae) (Fig. 3a). Our analyses also predicted a frameshift impacting three consecutive codons in *cox*I among 4 of our assemblies (MT492030.1, MT492035.1 to MT492037.1) compared to the reference, and shared with other *Ostrinia* including the recent Illumina sequence-base *O. nubilalis* assembly (MN793322.1;^39^) (Fig. 3b). This frameshift is predicted to result from upstream insertion and downstream deletion mutations that function in tandem to maintain the frame within the downstream CDS beyond the three affected codons (Fig. 3b). The remaining 25 predicted mutations compared to the reference were within rRNA^LSU^ (8 SNVs and three indels), rRNA^SSU^ (6 indels), and among tRNAs (5 SNVs and two indels; Table S2).

**Figure 4:**
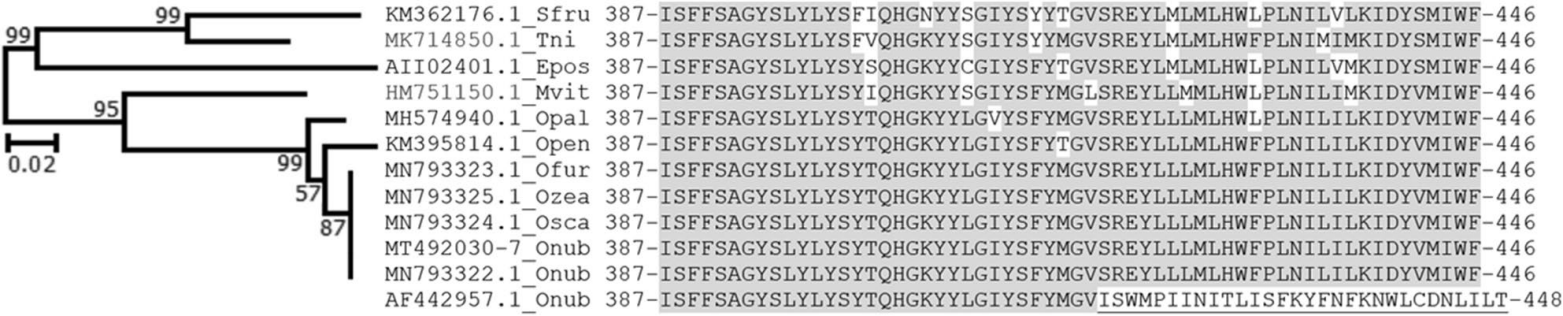
Alignment of 60 C-terminal residues from representative NADH Dehydrogenase subunit 4 (ND4) protein sequence accessions. Conserved residues (highlighted) showed ≥ 83.33% identity between species, with the exception of the 29 terminal residues from *O. nubilalis* AAL66246.1 predicted in the reference mitochondrial genome (AF442957.1). Frameshift in ND4 accession AAL66246.1 predicted from the reference AF442957.1 putatively caused by insertion of a cytosine (C) nucleotide at position 1254 of the ND4 CDS (genome position 8013; Table S2). Companion Maximum-Likelihood (ML) constructed based on the mtREV + F + G model of sequence evolution that maximized the Bayesian Information Criterion (BIC) score at 4929.63, and used an empirically-derived discrete gamma distribution (shape parameter; G) of 0.3988 that minimized the log-likelihood score (-2330.81). Total branch length of 0.56 amino acid changes per site. Species abbreviations: Sfru, *Spodoptera frugiperda*; Tni; *Trichoplusia ni*; Epos, *Epiphyas postvittana*; Mvit, *Maruca vitrata*; *Ostrinia* species abbreviations as in Fig. 3).

The frequency of each variant site among the eight F_1_ family-specific assemblies compared to the reference ranged from 0.13 to 1.00, of which 20 of the 82 variable positions (22.4%; *cox*I frameshift not counted as variable position) were conserved across all eight F_1_ families (Freq = 1.00 in Table S2). All of the 20 fixed differences with respect to the reference were SNVs, with the exception of a deletion in *nd*4. For this *nd*4 deletion, all assemblies from this study lacked a C nucleotide reported for position 8200 in reference AF442957.1, which caused a discrepancy in predicted C-terminal amino acids starting at position 418 of *nd*4 (Fig. 4). Subsequent multiple *nd*4 protein sequence alignment and phylogenetic comparison showed ≥ 83.33% amino acid identity between the terminal 60 residues of *nd*4 from our *O. nubilalis* assemblies and other lepidopteran species (Fig. 4), whereas *nd*4 from AF442957.1 showed complete mismatch in the terminal 29 residues. Inspection of bam alignments of short reads from each F_1_ family-specific library confirmed a lack of this C compared to the *O. nubilalis* reference (Fig. S2). Seven substitutions predicted in *cox*I and *cox*II genes among our assemblies were previously observed in a prior population genetics study, of which mutations at reference positions 2,554 and 3,050 were validated using *Hae*III and *Sau*3AI PCR-restriction fragment length polymorphism (PCR–RFLP) assays within that study (Table S2)^20^.

Analogously, there were a total of 77 variant sites, plus an Ile (ATT) triplet codon insertion within *atp*8 and a frameshift mutation in *cox*I identified within the multiple sequence alignment among our eight assemblies and a previous unenriched Illumina HiSeq read data-assembled mitochondrial genome sequence^39^ (MN792233.1; Fig. S2). Among these 77 variant sites, 43 (55.1%) were within CDS, of which 24 (55.8%) were nonsynonymous and predicted to cause an amino acid change (Table S4; Fig S3). Eight mutations were fixed differently between MT492031.1 to MT492034.1 compared to MN792233.1. Identical to comparison with AF442957.1, the Ile codon insertion within our F_1_ Family_ID_04 (MT492032.1) was not present with the *atp*8 CDS of MN792233.1 (Fig. 3a; Fig S3). Additionally, compared to reference AF442957.1 (RefSeq NC_003367.1), the *cox*1 frameshift identified within four of our eight assemblies was also present in MT492032.1 (Fig. 3b; Fig S3). The remaining 35 mutations were within the rRNA^LSU^ (*n* = 14), rRNA^SSU^ (*n* = 6), tRNAs (*n* = 14), and non-coding sequence (*n* = 1). Three of the substitutions within *cox*I and *cox*II genes were previously described by Coates et al.^20^, including two validated by *Hae*III and *Sau*3AI PCR–RFLP assays (Table S4).

### Comparison between enriched and unenriched reads for de novo mitochondrial genome assembly

Our use of reads from mitochondrion-enriched and unenriched libraries as input for SPAdes and SOAPdenovo2, respectively, resulted in assemblies that showed different levels of contiguity and computational efficiency. Specifically, library read data from enriched samples were assembled in 52 ± 8 s compared to 1380 ± 180 s among the unenriched libraries. Additionally, the total number of contigs within resulting assembles were lower among enriched (98 ± 41) compared to unenriched libraries (337,151 ± 154,655), wherein mean contig lengths were also greater for enriched (1110 ± 469 bp) compared to unenriched libraries (117 ± 992 bp) (remaining data not shown). Among the assembled contigs a mean of 31% and 0.0006% from enriched and unenriched libraries, respectively, showed homology to mtDNA based on BLASTn query with the mitochondrial reference genome sequence (Table S6). Moreover, among these mtDNA contigs, normalized read depth (RPKM) the estimated mean was ~ 65 times higher and significantly greater (t = 14.435; df = 1, 667; *p* < 2.2 × 10^–16^) among enriched samples compared to unenriched samples (Fig. S4). Furthermore, there was a distribution of contigs with both high RPKM estimate and length across all assemblies generated from enriched libraries that were of predicted to have mtDNA origin (Fig S5), and deemed to be of putative mitochondrial genome origin. In addition, the observed distribution of contigs of mtDNA origin with correspondingly low RPKM estimates and comparative bias toward short contig length were categorized at putative NUMTs. In contrast, contigs with high RPKM estimates in assemblies from each of the unenriched libraries were mostly of non-mtDNA origin (Fig. S5).

## Discussion

Variation in mtDNA sequence data is frequently used within interspecies phylogenetic and intraspecies population genetic analyses. However, a vast majority of studies assess variation based on a few genes or gene fragments, which potentially impact estimates of genetic variation and bias results, leading to the possibility of arriving at specious conclusions. This premise resides in evidence that the mutation rates vary among regions of the mitochondrial genome within plant species^40^ and humans^41^, as well as between species^42^. Methods have recently been developed to more efficiently obtain sequence data from entire mtDNA genomes, capitalizing on advancements in DNA sequencing technologies. These include generation of low-pass short shotgun sequencing reads from total genomic DNA, from which success of mtDNA genome assemblies rely on the proportionally high copy number of mtDNA compared to nuclear DNA^8,10^, as recently reported for several species of *Ostrinia*^39^. Targeted mtDNA sequencing employs various enrichments prior to library construction, including those based on commercial reagents^43^, isolation of highly intact circular mtDNA^30^, the requirement to develop specific antibodies for organelle pull-down^44^, or necessitating prior knowledge of the mtDNA genome sequence for probe-based nucleotide capture^31,32^. Although differential centrifugation was analogously used in methods to isolate human mitochondrion prior to mtDNA genome sequencing, the commercial kit protocols used are tailored to specific species^45^ and not directly transferable to other organisms. Here, we developed and validated a relatively rapid and low-cost differential centrifugation method to prepare mitochondrion-rich fractions from *O. nubilalis* thorax homogenates prior to DNA extraction and subsequent high-throughput DNA sequence library construction and sequencing. Although not tested here, this enrichment method is likely transferrable to other non-model organisms where no prior genome assemblies are available, due to our evidence that enrichments lead to significant reductions in raw data input and computational time requirements, as well as increase contiguity of resulting mtDNA assemblies. Furthermore, the additive effect of these efficiencies likely may enable individual-wise assessment of mitochondrial genome-wide haplotype variation on a population scale.

Our protocol development empirically determined that a second centrifugation step of 6000 rcf maximized the separation of mitochondria and nuclei in thorax homogenates based on qualitative Janus-Green visualization and semi-quantitative PCR comparisons between enriched and unenriched extracts (Fig. 1). Subsequent RT-qPCR and read alignment analyses indicated that while our differential centrifugation did not provide pure organelle fractions (Fig. 2a), it did provide for subsequent extracts enriched with mtDNA (Fig. 2b). Although these two methods lead to significantly different estimates of enrichment, a mean fold-enrichment of ≥ 48.2 was estimated among the libraries (Fig. 2c). These results suggest that secondary enrichment steps, such as the use of equilibrium density-gradient centrifugation^46^, could likely be incorporated into our protocol in cases where higher purity isolations are desired. While the level of enrichment and purity of our mitochondrion fractions were lower compared to methods employing mitochondrion surface protein-specific antibody capture^44,47^ or probe-assisted mtDNA isolation^31,32^, our method has the advantage of not requiring upfront development of custom biological reagents or reliance upon tailored commercial kits. Our differential centrifugation achieved 26% of reads aligning to a reference mitochondrial genome, which is comparable to a 22% alignment rate obtained following a two-step protocol that used a commercial circular DNA miniprep followed by antibody tagged paramagnetic bead isolation^43^. Regardless, due to variation in the mass of organelles, it may be anticipated that rcf values will need to be optimized for application to different species. Since mitochondrion enrichment prior to sequencing produced at least a 29.8-fold greater read alignment rate to the mitochondrial reference compared to unenriched controls (Table S1), showing that our optimized protocol facilitates a significant reduction in short sequence read input required for downstream assemblies.

Comparison of processes involved in the assembly of enriched compared to unenriched libraries, showed is a significant (~ 27-fold) reduction in computation time and lower required CPU node count and random access memory. Moreover, although it was possible to de novo assemble the mitochondrial genome from unenriched samples, the resulting assemblies were less contiguous compared to those from enriched libraries (Table S6). Specifically, the mean size of mitochondrial-derived contigs among unenriched assemblies were nearly ten-fold shorter and more numerous compared to contigs assembled from enriched libraries (Fig. S4, S5). Interestingly, these increases were achieved despite a 11.7- to 65.7-fold reduction in input reads among enriched libraries (Table S1) compared to that within unenriched SRA data files (SRX2498822-SRX2498825). Furthermore, due to lower sequencing costs and computational time, as well as increased contiguity, we suggest that enrichment provides greater accessibility to the potential for higher throughput assessment of haplotype variation among haplotypes of a species or among species. This may provide for downstream large-scale population and phylogenetic analyses that arguably remain out of reach using other methods.

In this study, we applied the full mtDNA genome assemblies derived from our enrichment method to assess variation across *O. nubilalis* maternal lineages. The genus *Ostrinia* is a model for the study of sympatric population divergence and incipient species formation^19,48^ and comprises two corn borer species, *O. nubilalis,* and *O. furnacalis*, which are major pest insects that feed on cultivated maize in North America, Europe and/or Asia. Compared to prior *O. nubilalis* mtDNA genome assemblies AF442957.1^20^ and MN792233.1^39^, our eight F_1_ families showed 82 and 77 SNVs, respectively (Table S2, S4). These comparisons also predicted a single insertion and frameshift mutation. Among the mutations we predicted across all eight F_1_ family-based assemblies, 20 and 8 were fixed differently compared to the reference AF442957.1^20^ and MN792233.1^39^, respectively. Fixed differences could represent natural intraspecies haplotype differences, but given the small sample size of the eight F_1_ families, some of the fixed differences could be a consequence of sampling bias from a laboratory colony that has undergone a genetic bottleneck and influenced by random genetic drift for > 12 generations. Alternatively, some of these SNVs could have resulted from Sanger sequencing errors incorporated into the reference assembly AF442957.1^20^. The latter might be concluded, for a C nucleotide inserted within *nd*4 at position 8200 from the Sanger reference as compared to all Illumina-based assemblies (Fig. S2; MN792032.1), which caused a fixed discrepancy in the C-terminal 29 residues of *nd*4 compared to assemblies from other *Ostrinia* and lepidopteran species generated from short-read data (Fig. 4). This putative correction suggests that the high read depths obtained from short read sequencing of enriched mtDNA libraries provides superior error correction capabilities compared to dual-pass Sanger read data.

The structural gene annotation of our eight assembles showed two major differences compared to the reference. Firstly, our results also predicted a novel ATT insertion within F1 family 4 (MT492032.1) residing at reference position 3,953 within *atp*8, causing a putative in-frame Ile codon duplication at *atp*8 amino acid position 52 (Fig. 3a). This insertion is novel compared to all other exemplar sequences within the genus *Ostrinia* and other species of Lepidoptera, with the exception of a Leu codon (TTA) found at the orthologous position of the *Ochlodes venata* mitochondrial genome (Fig. 3b). Moreover, the two adjacent Ile residues are deleted at orthologous sites from the butterfly *Parnassius epaphus*. These comparisons suggest that sites orthologous to *Ostrinia atp*8 amino acid positions 52 and 53 may show relaxed functional constraint, and thus indels involving these amino acids might putatively be impacted by purifying selection to a lesser degree. Although intriguing, testing this hypothesis is beyond the scope of the current study. Secondly, out of eight assemblies, four show a pair of indels that cause a frameshift over three amino acids of *cox*I (Fig. 3b). Specifically, compensation for the upstream deletion by a 10 bp downstream insertion retains the predicted downstream coding frame. Typically, mitochondrial frameshifts are highly deleterious^49^, but can result in apparently non-deleterious changes depending upon position and context^50,51^. In other instances, suppressor tRNA mutations or “leaky” ribosomal frameshift compensation mechanisms have been proposed^52,53^. Regardless, the frameshift described here, resulting in the putative alteration of 3 amino acids of *cox*I, has an unknown functional consequence and was not tested further. Due to presence within reads at this position, the indel is likely valid, but may be unique among individuals within a relatively inbred laboratory colony and not representative of wild individuals under the influence of selective forces in field conditions.

The variation we detected cannot be interpreted as representing population frequencies, but would require large-scale screening and comparison of random samples from wild populations. Regardless, prior population screening of 1,414 *O. nubilalis* individuals from North American populations determined that 89.6% were comprised of a single haplotype based on PCR–RFLP, wherein the sequencing of a 2,156 bp fragment comprised of *cox*I and *cox*II from 14 individuals predicted 26 polymorphic positions^20^ (≤ 0.8% variation). Interestingly, of the 82 variant sites predicted in the current study, seven were identical to the set of 26 previously predicted by Coates et al.^20^. Furthermore, substitutions validated by *Hae*III and *Sau*3AI PCR–RFLP were predicted within comparisons of our assemblies to the Sanger sequence assembled reference^38^ and the more recent short read-based assembly^39^, suggesting variants are real as opposed to sequencing or assembly artifacts.

Prior phylogenetic reconstructions differ between *Ostrinia* species when using variation in sequence data from *cox*II^54^ *cox*I^55^ gene fragment data, as compared to full mitochondrial genome alignments^39^. Specifically, full genome analysis by Zhou et al.^39^ predicted that corn borer sister species, *O. nubilalis,* and *O. furnacalis*, were within a clade along with *O. scapulalis*. In comparison, an *O. nubilalis* and *O. scapulalis* clade were separated from *O. furnacalis* by *O. zealis* based on *cox*II gene^54^ or *cox*I sequence data^55^. Although not investigated further for *Ostrinia*, a disparity in the evolutionary rate between mitochondrial genome regions (genes or gene fragments) is described across taxa^7,27^, suggesting that phylogenetic relationships could be biased when applying a subset of mitochondrial haplotype variation within short fragments. Therefore, phylogenetic relationships predicted based on the full mtDNA genome sequence might arguably be more reflective of evolutionary divergence patterns, at least over appropriate timespans^14^.

Prior to our analysis, *O. nubilalis* mtDNA variation was generally estimated to be low and uninformative in a population context^21,22^. By implementing a mitochondrial enrichment method and predicting variation across the full mitochondrial genome sequences, we identified a greater number of variant sites within *O. nubilalis* than previously reported. The future application of analogous full mtDNA sequencing to population or phylogenetic studies will contribute to unbiased estimates of inter- and intra-species variation. This study demonstrated that a differential centrifugation method could be a component of this goal, due to demonstrated enrichment of cell homogenates for mitochondrion and correspondingly removing nuclei prior to DNA extraction. This method provides a low-cost option for organelle enrichment, resulting in samples with a high proportion of mtDNA for library construction and sequencing. Undoubtedly further cost efficiencies could be implemented through indexing of a greater number of sample libraries within higher capacity flow cells compared to the MiSeq that was used here. Furthermore, we demonstrate increased downstream computational efficiencies and resulting assembly contiguities achieved during assembly of reads from mitochondrion-enriched libraries compared to those obtained from unenriched libraries. Although we applied these methods to *O. nubilalis*, the protocol could be adapted and optimized for other species, thereby facilitating a higher throughput of mtDNA genome sequencing for application in unbiased population and phylogenetic studies, where additive effects of time and cost saving may facilitate performance on larger scales.

## Methods

### Specimens and sampling

Z-pheromone race *Ostrinia nubilalis* pupae were obtained from the United States Department of Agriculture, Agriculture Research Service, Corn Insects and Crop Genetics Unit (USDA-ARS, CICGRU) in Ames, IA. Pupae were individually maintained in a Percival incubator (Percival, Boone, IA) (16:8 [L:D] h; 26 °C; 40 to 60% RH). Upon eclosion, single mate pairs were initiated to create F_1_ families (maternal-specific haplotype lineages). Pairs were maintained in wire mesh cages with wax paper for oviposition substrate, as previously described^56^. Mated pairs were monitored, and eggs were collected daily for seven days. Wax papers with egg masses were stored in the incubator (16:8 [L:D] h; 26 °C; 40–60% RH) until near hatch. Egg masses from each F_1_ family were separately placed into individual 10-cm diameter plastic containers with approximately 50-ml of standard *O. nubilalis* meridic diet^57^. Pupae were collected from each family and placed into individual paper cups until eclosion. Adults were either live-dissected or frozen at − 20 °C.

### Optimization of differential centrifugation and DNA extraction

Thorax tissue containing flight muscle was dissected from three live *O. nubilalis* adults per F_1_ family. Since mitochondria are maternally inherited without recombination, tissues were pooled by family in 2.0-mL glass Dounce homogenizers (Corning Inc., Corning, NY) and homogenized on ice in 1.0-mL of a homogenization medium (0.32 M sucrose, 1.0 mM EDTA, 10.0 mM Tris–HCl, 4 °C, pH 7.8). Each F_1_ family homogenate was transferred to a separate 1.5 mL microcentrifuge tube.

Differential centrifugation was used to fractionate extracts into nuclear and non-nuclear fractions based on molecular mass. The insoluble exoskeleton, cell debris, and other particles were removed from samples at 1,000 relative centrifugal force (rcf) for 10 min at 4 °C in an Eppendorf 5417R centrifuge (Eppendorf, Hauppauge, NY). Aqueous supernatant was transferred to new 1.5-mL microcentrifuge tubes. Replicate samples within each F_1_ family were centrifuged at 2000, 4000, 6000, and 8000 rcf at 4 °C for 10 min to optimize the force that most effectively pelleted nuclei (referred to as nuclear fraction) while retaining mitochondria in the supernatant. The remaining supernatants of each replicate (F_1_ family pool) were transferred to new 1.5-mL microcentrifuge tubes and centrifuged at 4 °C for 10 min at 13,000 rcf to pellet the remaining organelles (referred to as mitochondrial fraction).

The optimal centrifugal force was determined using microscopy and the ratio of amplified DNA fragment intensities as determined by semi-quantitative/qualitative PCR of mitochondrial (*cox*I), and nuclear (*apn*1) indicator genes. Subsamples of the 13,000 rcf pellet were treated with Janus Green B cell normalization stain (Abcam Inc., Cambridge, MA); stained mitochondria were observed by light microscopy (Olympus Microscopy, model BH-2, Tokyo, Japan)^58^. DNA was then extracted from each of the fractionated replicates within F_1_ family pools using the DNEasy Blood and Tissue Extraction Kit according to manufacturer directions (Qiagen, Hilden, Germany), except DNA was eluted from silica columns using 50.0 µL of Elution Buffer. Samples were serially diluted using nuclease-free water, and 1.0 µL from each dilution and was PCR amplified in duplicate using mitochondrial primers (HCO/LCO)^59^ and nuclear primers (OnFLWA-01) in separate reactions as described earlier^60^. Entire products were separated by 2% agarose gel electrophoresis with the Lambda *Hind*III and *Eco*RI ladder. Based on staining and PCR results, the following method was employed: (1) initial pelleting of cell debris at 1000 rcf; (2) resultant supernatant centrifuged at 6000 rcf to obtain nuclear fraction; (3) resultant supernatant centrifuged at 13,000 rcf to obtain the mitochondrial fraction. All centrifugation steps were conducted for 10 min at 4 °C.

The empirically-determined optimal rcf was employed to pellet nuclei with three frozen/preserved adult individuals of each F_1_ family. Fractions were subsequently extracted with DNEasy extraction kits, as described above. Unenriched controls were prepared by identical DNA extraction from dissected thorax tissue without nuclear or mitochondrion fractionation from individual Z-strain *O. nubilalis* field samples collected at South Shore, SD (SDSS ♀1 and SDSS ♀2; contributed by Dr. Micheal Catangui, South Dakota State University).

### Quantification of mitochondrion enrichment at optimized parameters

Using the optimized centrifugation forces (6000 and 13,000 rcf), the fold-enrichment of mitochondrion compared to nuclei was estimated by two different methods: (1) analysis of cycle threshold (C_T_) obtained through real-time quantitative PCR (qPCR) with nuclear and mitochondrial primers, and (2) high-throughput read alignment rates to a mitochondrial reference genome of enriched and unenriched samples.

For real-time qPCR, DNA was quantified from the nuclear and mitochondrial fractions of the eight F_1_ families and the two unenriched controls on a NanoDrop 2000 spectrophotometer (Thermo Scientific, Wilmington, DE, USA). Concentrations were adjusted to ~ 1.0-ng/μl with nuclease-free water. All samples were PCR amplified in duplicate on an MX3000P real-time qPCR system (Stratagene, La Jolla, CA) with 2.0-ng of the template along with 10.0-nM of each forward and reverse primer in iQsupermix reactions per to manufacturer instructions (BioRad, Hercules, CA). Mitochondrial DNA-specific primers OnCOXIrtS, 5′-CCT GAT ATA GCA TTC CCA CGA ATA-3′, and OnCOXIrtA, 5′-AAC CAG TTC CTG CTC CAT TT-3′, were designed using the RealTime qPCR Assay tool^61^. Single locus nuclear primers, APN1RT-1F and APN1RT-1R, amplifying the aminopeptidase N1 gene (*apn*1), were designed and applied as previously described^56^. Statistical significance of differences in CT values from mitochondrial and nuclear primer amplification reactions of the mitochondrial and nuclear fractions, and unenriched controls, were analyzed with a general linearized model, one-way ANOVA in RStudio version 1.0.153^62,63^ with the Estimated Marginal Means (emmeans) package^64^. Summaries of paired comparisons were made with Tukey’s HSD method using the Visualizations of Paired Comparisons (multcompView) package^65^.

DNA extracts from each mitochondrial-enriched fraction for each F_1_ family were submitted to the Iowa State University (ISU) DNA Facility (≤ 100-ng each). Individual indexed short insert libraries were generated using AmpliSeq Library Plus methods according to manufacturer instructions (Illumina, San Diego, CA). All indexed libraries were sequenced in a single lane of a MiSeq system (Illumina, San Diego, CA). Data were received in fastq format and submitted to the National Center for Biotechnology Information (NCBI) GenBank short read archive (SRA). Additionally, four previously generated Illumina HiSeq reads from the whole genomic sequence of pooled *O. nubilalis* population samples were downloaded from the NCBI SRA database (Accessions SRX2498822-SRX2498825^66^).

Reads from each of our mitochondrion-enriched libraries (*n* = 8) and whole genomic libraries (*n* = 4) were aligned to the 14,535 nucleotides *O*. *nubilalis* mitochondrial genome reference sequence (NCBI GenBank accession AF442957.1; RefSeq NC_003367.1) assembled previously from Sanger sequencing data from overlapping PCR amplicons^38^. Nucleotide quality was initially assessed and visualized with fastqc^67^ (fastqc/0.11.7-d5mgqc7). Reads were trimmed for those with a Phred quality score (*q*) ≥ 20 over a 4-nucleotide sliding window: the first 15 nucleotides (HEADCROP:15) and the last ten nucleotides (TAILCROP:10) were removed using trimmomatic^68^ (trimmomatic/0.36-lkktrba). Paired reads were aligned to the reference sequence using bowtie2^69^ (bowtie2/2.3.4.1-py2-jl5zqym) in unpaired mode (-U). Resulting sequence alignment and map (.sam) files were converted to binary alignment and map (.bam) format, and inclusive reads filtered for those that mapped to the reference sequence (view –bhF 2) and had a mapping quality score (*Q*) ≥ 30 (view –bhq 30) using SAMtools^70^ (SAMtools/1.9-k6deoga). All bioinformatic operations were performed on the ISU Pronto server (See Table S5 for an example of Linux shell commands). The proportion of reads to meet filtering criteria in the mitochondrial-enriched and unenriched whole genomic libraries were calculated, and the statistical significance of the difference in the proportion of aligned reads was estimated using a two-sample t-test at a threshold α = 0.05^62,63^.

Mitochondrial fold-enrichment was calculated for real-time qPCR and MiSeq read alignment methods. For qPCR, the relative difference in copy number between C_T_ values from enriched and unenriched fractions was used to calculate relative fold enrichments. Delta C_T_ (ΔC_T_) values were calculated by subtracting the C_T_ value produced with mitochondrial primers from the C_T_ value produced from the same sample with nuclear primers. For each family, mitochondrial fold-enrichment was calculated with the formula: 2^(mitochondrial fraction ΔCT – unenriched control ΔCT)^. Likewise, nuclear fold-enrichment was calculated with 2^(nuclear fraction ΔCT – unenriched control ΔCT)^. In the read alignment method, mitochondrial fold-enrichment was calculated as the proportion of reads from each enriched sample that aligned to the reference divided by the mean proportion of reads that analogously aligned from the unenriched samples. Statistical significance in fold enrichment estimations was calculated using paired t-tests at a threshold α = 0.05^62,63^.

### De novo mitochondrial genome assembly, annotation, and variant prediction

Alignments for mitochondrion-enriched reads to the *O. nubilalis* mitochondrial genome reference (AF442957.1; RefSeq NC_003367.1) were sorted (SAMtools -sort), and the wrapper script plasmidspades.py was used to de novo assemble reads with SPAdes^71^ (spades/3.11.1-py2-arfy7sc; default parameters) to increase the coverage of the mitochondrial genome in comparison to the reference genome (See Table S5 for an example of Linux shell commands). Resulting Kmer contigs of the mitochondrion-enriched de novo assembled reads were used as subjects against the mitochondrial reference genome query^38^ (AF442957.1; RefSeq NC_003367.1) with the BLASTn algorithm^72^ (ncbi-rmblastn/2.6.0-2kyyml7; default parameters) because the de novo assembly increased the coverage of the mitochondrial genome in comparison to the reference genome. Kmer contigs from each F_1_ family that generated BLASTn hits with *E*-values ≥ 10^–90^ were assembled separately with the Sequence Assembly Program, CAP3^73^ (cap3/2015-02-11-2jwa5sb; default parameters). Gene features in each assembled CAP3 contig (mitochondrial genome) were annotated by queries with protein and RNA coding sequences from the reference assembly (AF442957.1; RefSeq NC_003367.1) using the BLASTn algorithm. Any discrepancies were corrected based on evidence from corresponding bowtie2-generated bam alignments visualized in Integrative Genome Viewer (IGV)^74^. Final annotated assemblies were submitted to the NCBI GenBank nr database. Variable nucleotide positions among the reference and F_1_ family-specific mitochondrial genomes were identified from corresponding filtered .bam files with bcftools^75^ (bcftools/1.9-womp5gh) using the call and varFilter options (See Table S5 for an example of Linux shell commands; minimum read depth ≥ 2,000). Results were output in VCF format v4.2^76^. Bedtools v 2.29.2^77^ was implemented to retrieve genome sequence features (CDS, rRNA, and tRNAs). Variations between our submitted annotated genomes and the reference genome were manually verified in Microsoft Word.

Additionally, a multiple sequence alignment was generated between our assembled mitochondrial genomes and the 15,248 bp *O. nubilalis* mitochondrial genome assembled from unenriched Illumina HiSeq read data^39^ (MN793322.1). This *O. nubilalis* sample was collected from the Yili area of the Xinjiang Autonomous region of western China. Nucleotide sequences for our eight assembles and MN793322.1 were loaded into the MEGA8.0 alignment utility^78^, and aligned using the ClustalW algorithm^79^ (default parameters) with adjustments to codon frame made manually. Wrapping to a width of 100 bp and demarcation of variant sites was performed with the Multiple Alignment Viewer^80^; alignment was manually decorated with corresponding gene intervals. The position of variant sites within each corresponding assembly and the consensus were retrieved from the multiple sequence alignment NCBI MSA viewer^81^. The location and impact of variants on codon use within protein CDS were identified manually and verified by the alignment of corresponding nucleotide and protein sequences retrieved from GenBank accessions.

A discrepancy in the putative translation of ND4 was predicted between the reference (AF442957.1; RefSeq NC_003367.1) and all subsequent Illumina short read-based sequence assemblies (MT793322.1 and this study). To investigate this discrepancy further, a multiple protein sequence alignment was generated among ND4 orthologs from *Ostrinia* species (*O*. *nubilalis*, *O. furnacalis*, *O. scapulalis*, *O. zealis*, *O. penitalis*, and *O. palustralis*) and a subset of related lepidopteran species using the ClustalW algorithm^79^ within the MEGA8.0 alignment utility^78^ (default parameters; accessions provided in Fig. 4). The general reversible mitochondrial model of protein sequence evolution^82^ (mtREV24) with among site frequency variation (F) and empirically-derived gamma distribution (G) maximized the Bayesian Information Criterion (BIC) and was chosen as the most appropriate model. The mtREV24 + G + F model was subsequently applied within a Maximum-Likelihood (ML) approach to reconstruct an unrooted phylogeny with a consensus tree constructed from 1,000 iterative bootstrap pseudo-replicate sampling steps.

### Comparison between enriched and unenriched reads for de novo mitochondrial genome assembly

Short Illumina reads from four unenriched libraries Accessions SRX2498822-SRX2498825^66^, that contained from 44.6 to 157.7 million reads, were de novo assembled with SOAPdenovo2^83^ (soapdenovo2/240-bg2qxy6; default parameters). Time to complete assemblies and total contigs assembled were quantified, and compared to those for our eight libraries constructed from mitochondrion-enriched extracts. Read depth among contigs within each resulting SOAPdenovo2 assembly were determined by realigning component reads using bowtie2^69^ (bowtie2/2.3.4.1-py2-jl5zqym) in unpaired mode (-U) as described above, and subsequent .bam file conversion, indexing, and retrieval of read counts performed using Samtools^70^-view, -index, and -idxstats commands respectively. Normalized read counts for contig length was performed by calculation of reads per kilo base per million mapped reads (RPKM; number of reads / (contig length/1000 * total number of reads/1,000,000). RPKM was analogously estimated from .bam output from SPAdes assemblies performed above for enriched library read data, and significant of comparative difference in assembled contig length and read depth estimates using paired t-tests within Rstudio^62,63^ and evaluated at a threshold α = 0.05. The SOAPdenovo2 assembled contigs assembled from each of the unenriched library reads were loaded into separate local BLAST databases. Each database was then queried with the entire reference *O. nubilalis* mitochondrial genome sequence (AF442957.1) using the BLASTn algorithm^72^, with results filtered by an *E*-value cutoff of ≤ 10^–40^ and sorted by query start position. The number and percent identity of MtD fragments within each assembly were compared to that obtained from separate assemblies derived from enriched libraries (see above).

### Ethics declarations

This research was not conducted on human subjects and was consistent with the United States Animal Welfare Act.

## Supplementary information

Supplementary Information.

## Data Availability

Illumina HiSeq3000 reads can be found on National Center for Biotechnology Information (NCBI) BioProject PRJNA604593 under Short Read Archive (SRA) accession numbers SRR11007774-SRR11007781. Annotated mitochondrial genome sequence assemblies are submitted to the NCBI non-redundant (nr) database under accessions MT492030.1-MT492037.1. The *Ostrinia nubilalis* mitochondrial genome used as a reference in this study can be found under NCBI GenBank accession AF442957.1. *O. nubilalis* population Pool-seq reads that were considered unenriched controls are available in the SRA database (Accessions SRX249882-SRX2498825^66^).
