# Supplementary material for: Prediction of mitochondrial genome-wide variation through sequencing of mitochondrion-enriched extracts
Source: Sci Rep. 2020 Nov 5;10:19123. doi: 10.1038/s41598-020-76088-0 (PMC7645498; doi:10.1038/s41598-020-76088-0)
Supplement: Supplementary file 1 — Supplementary Information. [file 41598_2020_76088_MOESM1_ESM.pdf]

SUPPLEMENTARY INFORMATION

Prediction of mitochondrial genome-wide variation through sequencing of mitochondrion-enriched extracts

Kelsey E. Fisher[1,4], Steven P. Bradbury[1,2], and Brad S. Coates[3]
Iowa State University, Ames, IA 50011

[1]Department of Entomology, Iowa State University, Ames, IA, 50011

[2]Department of Natural Resource Ecology and Management, Iowa State University, Ames, IA 50011

[3]United States Department of Agriculture, Agriculture Research Station, Corn Insects and Crop Genetics Research Unit, Ames, IA 50011

[4]Corresponding author, email: kefisher@iastate.edu, phone: 609-221-5129

Figure S1: Full gel electrophoresis of serial-diluted DNA extracted from the mitochondrial and nuclear fractions amplified in duplicate with mitochondrial primers (HCO/LCO; Folmer et al. 1994) and nuclear primers (Onapn1-F and -R; [46]). The cropped portion of the gel present in the manuscript is surrounded by the white box. Mitochondrial fraction (13,000 rcf) was PCR amplified with mitochondrial primers (i) and nuclear primers (ii). Nuclear fraction (6,000 rcf) was PCR amplified with mitochondrial primers (iii) and nuclear primers (iv). Lambda *Hind*III and *Eco*RI ladder appears to the left of each panel and was loaded alone in panel (v). Although nuclear DNA was present in both the mitochondrial and the nuclear fractions, mitochondrial DNA was only present in the mitochondrial fraction.

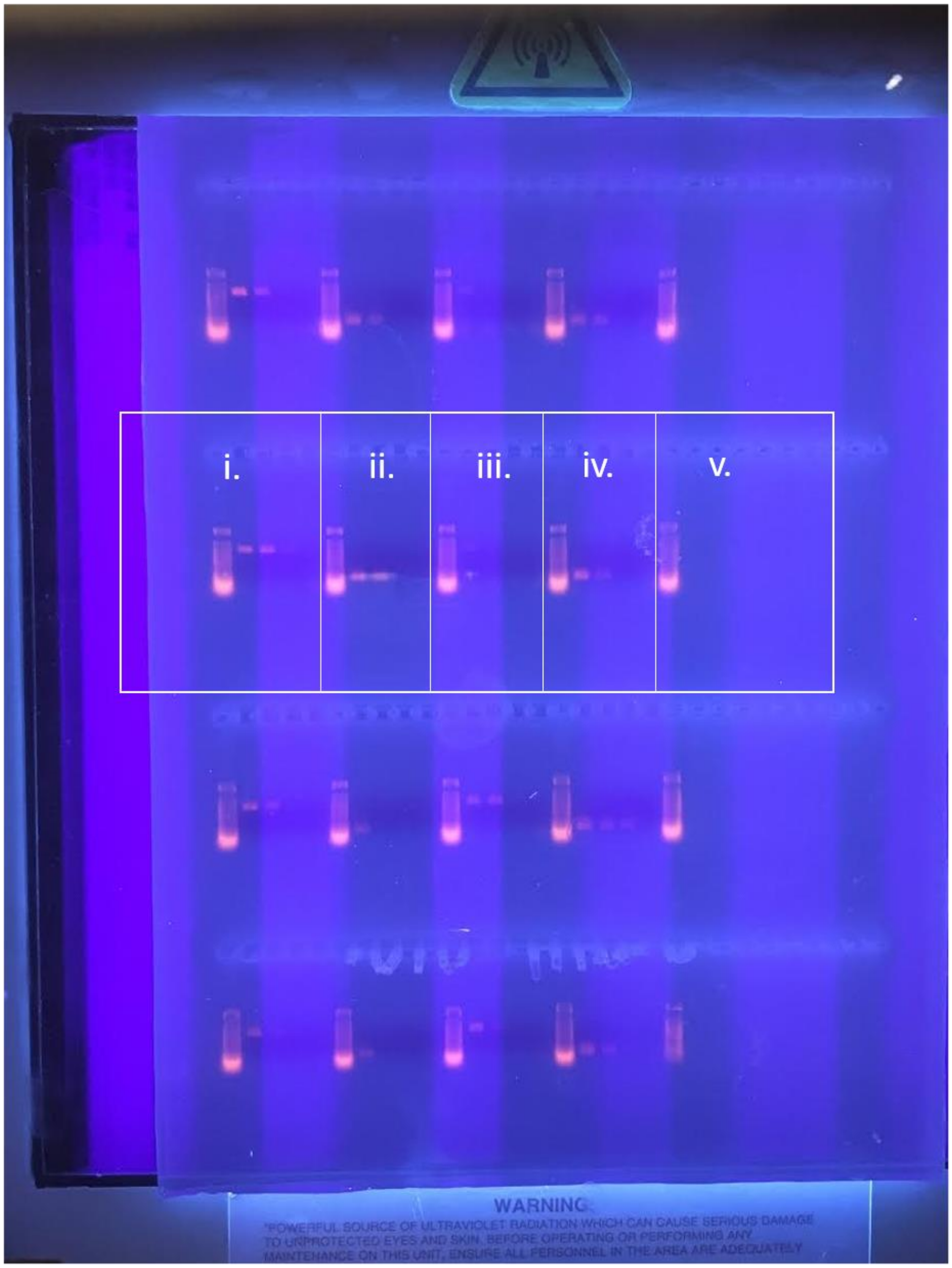

Figure S2: Alignment of short reads within bam file generated for MT492030.1 (F_1_Family_ID_02) showing gap and lack of C nucleotide insertion at position 8200 of the *Ostrinia nubilalis* reference AF4429573.1 mitochondrial genome sequence. Identical results for read alignments from remaining 7 families (accession MT492031.1 to MT492037.1; results not shown), which provided additional evidence for proposing an ND4 CDS correction that corresponds with phylogenetic evidence (Fig. 4) and the protein sequence annotated in the *O. nubilalis* mitochondrial genome assembly MN792233.1 [34].

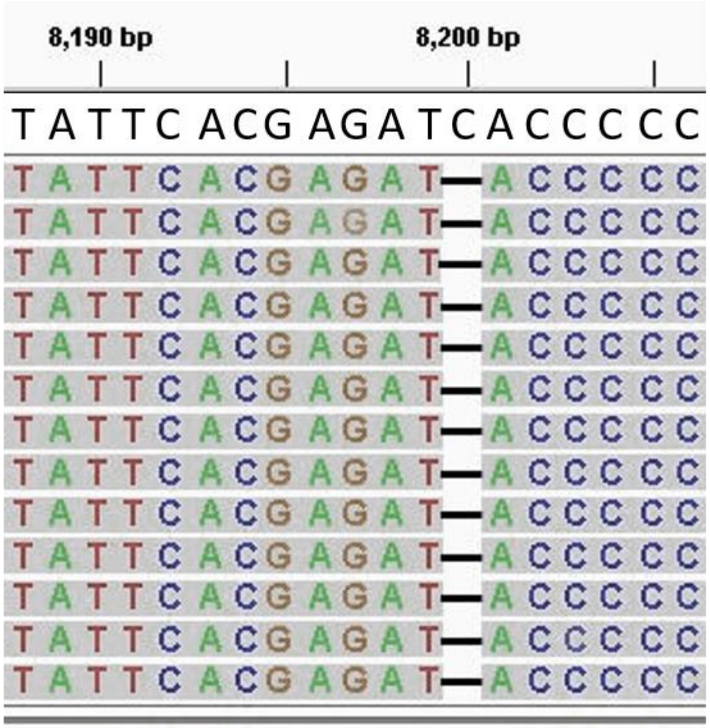

Figure S3. Multiple sequence alignment of Illumina short read sequence assembled Ostrinia nubilalis mitochondrial genome sequences. Corresponding protein coding sequence (CDS) and predicted amino acids, and RNA gene regions are indicated by strand. Highlighted nucleotides: substitutions (yellow), insertions/deletions (blue), start codons (green) and stop codons (*; red). Positional information given in Table S3.

```
             1 [         .         .         .         .         :         .         .         .         .         1 100
1 MN793322.1     ----------------------------------------------------------------------------------------------------
2 MT492036.1     ----------------------------------------------------------------------------------------------------
3 MT492037.1     --------------------------------------------------------------AATTAATTATTAAGTTTAATAATTAATTAAATTGATTT
4 MT492030.1     ------------------------------------------------------------------------------------TTAATTAAATTGATTT
5 MT492031.1     TAATTAATTATTAAGTTTAATAATTAATTAAATTGATTTATTAATATATTATAAATCAATTTAATTAATTATTAAACTTAATAATTAATTAAATTGATTT
6 MT492032.1     ----------------------------------------------------------------------------------------------------
7 MT492033.1     ----------------------------------------------------------------------------------------------------
8 MT492034.1     ----------------------------------------------------------------------------------------------------
9 MT492035.1     ----------------------------------------------------------------------------------------------------


            101          .         .         .         .         :         .         .         .         .         2 200
1 MN793322.1     ----------------------------------------------------------------------------------------------------
2 MT492036.1     ------------------------------------------------------------ATATATATA-TATATAT-ATAT------------------
3 MT492037.1     ATTAATATATTATAAATATAATTTCTATTATATATATATATATATATTAAATATAATAATTAATTAAATATTTAATATATAT------------------
4 MT492030.1     ATTAATATATTATAAATATAATTTCTATTATATATATATATATATATTAAATATAATAATTAATTAAATATTTAATATATATATATATATATATAT------
5 MT492031.1     ATTAATATATTATAAATATAATTTCTATTATATATATATATATATATTAAATATAATAATTAATTAAATATTTAATATATATATATATATATATATATATAT
6 MT492032.1     --------ATTATAAATATAATTTCTATTATATATATATATATATATTAAATATAATAATTAATTAAATATTTAATATATAT------------------
7 MT492033.1     ----------------------------------------------------------------------------ATATAT------------------
8 MT492034.1     ----------------------------------------------------------------------------ATATAT------------------
9 MT492035.1     ----------------------------------------AATTGATTTATTAATATATTATAAATATAATTTCTATTATAT------------------


            201          .         .         .         .         :         .         .         .         .         3 300
                                                                 |tRNA-Met (CAU)
                                                                 ttaaaaataagctaaataaagcttttgggttcatacctcaaatataaagga
1 MN793322.1     --------------------------------------------------TAAAAATAAGCTAAATAAAGCTTTTGGGTTCATACCTCAAATATAAAGGA
2 MT492036.1     --ATATATATATATAAACCATTCCTAATTTTTTTTCTTTTAATAATAAATTAAAAATAAGCTAAATAAAGCTTTTGGGTTCATACCTCAAATATAAAGGA
3 MT492037.1     --ATATATATATATAAACCATTCCTAATTTTTTTTCTTTTAATAATAAATTAAAAATAAGCTAAATAAAGCTTTTGGGTTCATACCTCAAATATAAAGGA
4 MT492030.1     --ATATATATATATAAACCATTCCTAATTTTTTTTCTTTTAATAATAAATTAAAAATAAGCTAAATAAAGCTTTTGGGTTCATACCTCAAATATAAAGGA
5 MT492031.1     ATATATATATATATAAACCATTCCTAATTTTTTTTCTTTTAATAATAAATTAAAAATAAGCTAAATAAAGCTTTTGGGTTCATACCTCAAATATAAAGGA
6 MT492032.1     --ATATATATATATAAACCATTCCTAATTTTTTTTCTTTTAATAATAAATTAAAAATAAGCTAAATAAAGCTTTTGGGTTCATACCTCAAATATAAAGGA
7 MT492033.1     --ATATATATATATAAACCATTCCTAATTTTTTTTCTTTTAATAATAAATTAAAAATAAGCTAAATAAAGCTTTTGGGTTCATACCTCAAATATAAAGGA
8 MT492034.1     --ATATATATATATAAACCATTCCTAATTTTTTTTCTTTTAATAATAAATTAAAAATAAGCTAAATAAAGCTTTTGGGTTCATACCTCAAATATAAAGGA
9 MT492035.1     --ATATATATATATAAACCATTCCTAATTTTTTTTCTTTTAATAATAAATTAAAAATAAGCTAAATAAAGCTTTTGGGTTCATACCTCAAATATAAAGGA

            301          .         .         .         .         :         .         .         .         .         4 400
                                 |tRNA-Ile (GAU)                                                    |
                 aatacctttttttaaaataaagtgcctgactaaaggattattctgataggataaattaagtagattattaactacctttatta
1 MN793322.1     AATACCTTTTTTTAAAATAAAGTGCCTGACTAAAGGATTATTCTGATAGGATAAATTAAGTAGATTATTAACTACCTTTATTATTATATTTTATAGAAT
2 MT492036.1     AATACCTTTTTTTAAAATAAAGTGCCTGACTAAAGGATTATTCTGATAGGATAAATTAAGTAGATTATTAACTACCTTTATTATTATATTTTATAGAAT
3 MT492037.1     AATACCTTTTTTTAAAATAAAGTGCCTGACTAAAGGATTATTCTGATAGGATAAATTAAGTAGATTATTAACTACCTTTATTATTATATTTTATAGAAT
4 MT492030.1     AATACCTTTTTTTAAAATAAAGTGCCTGACTAAAGGATTATTCTGATAGGATAAATTAAGTAGATTATTAACTACCTTTATTATTATATTTTATAGAAT
5 MT492031.1     AATACCTTTTTTTAAAATAAAGTGCCTGACTAAAGGATTATTCTGATAGGATAAATTAAGTAGATTATTAACTACCTTTATTATTATATTTTATAGAAT
6 MT492032.1     AATACCTTTTTTTAAAATAAAGTGCCTGACTAAAGGATTATTCTGATAGGATAAATTAAGTAGATTATTAACTACCTTTATTATTATATTTTATAGAAT
7 MT492033.1     AATACCTTTTTTTAAAATAAAGTGCCTGACTAAAGGATTATTCTGATAGGATAAATTAAGTAGATTATTAACTACCTTTATTATTATATTTTATAGAAT
8 MT492034.1     AATACCTTTTTTTAAAATAAAGTGCCTGACTAAAGGATTATTCTGATAGGATAAATTAAGTAGATTATTAACTACCTTTATTATTATATTTTATAGAAT
9 MT492035.1     AATACCTTTTTTTAAAATAAAGTGCCTGACTAAAGGATTATTCTGATAGGATAAATTAAGTAGATTATTAACTACCTTTATTATTATATTTTATAGAAT
                                                                                                     aatataaaatatctta
                                                                                                     |
```

```
             401         .         .         .         .         :         .         .         .         .        5 500
1 MN793322.1    TAAACTATATCTAATAGTATCAAAAACTATCGTGCATCATACACTAAAATATAAATAAATTAAAATATAAATTTAATTTTATATAAAATATTTTTTATTT
2 MT492036.1    TAAACTATATCTAATAGTATCAAAAACTATCGTGCATCATACACTAAAATATAAATAAATTAAAATATAAATTTAATTTTATATAAAATATTTTTTATTT
3 MT492037.1    TAAACTATATCTAATAGTATCAAAAACTATCGTGCATCATACACTAAAATATAAATAAATTAAAATATAAATTTAATTTTATATAAAATATTTTTTATTT
4 MT492030.1    TAAACTATATCTAATAGTATCAAAAACTATCGTGCATCATACACTAAAATATAAATAAATTAAAATATAAATTTAATTTTATATAAAATATTTTTTATTT
5 MT492031.1    TAAACTATATCTAATAGTATCAAAAACTATCGTGCATCATACACTAAAATATAAATAAATTAAAATATAAATTTAATTTTATATAAAATATTTTTTATTT
6 MT492032.1    TAAACTATATCTAATAGTATCAAAAACTATCGTGCATCATACCCTAAAATATAAATAAATTAAAATATAAATTTAATTTTATATAAAATATTTTTTATTT
7 MT492033.1    TAAACTATATCTAATAGTATCAAAAACTATCGTGCATCATACACTAAAATATAAATAAATTAAAATATAAATTTAATTTTATATAAAATATTTTTTATTT
8 MT492034.1    TAAACTATATCTAATAGTATCAAAAACTATCGTGCATCATACACTAAAATATAAATAAATTAAAATATAAATTTAATTTTATATAAAATATTTTTTATTT
9 MT492035.1    TAAACTATATCTAATAGTATCAAAAACTATCGTGCATCATACACTAAAATATAAATAAATTAAAATATAAATTTAATTTTATATAAAATATTTTTTATTT
                ATTTGATATAGATTATCATAGTTTTTGATAGCACGTAGTATGTGATTTTATAT
                atttgatatagattatcatagtttttgatagcacgtagtatgtgattttatat
                                                        <- tRNA-Gln |

                              ND2 ->
             501         .         .         .         .         :         .         .         .         .        6 600
                               M  N  S  N  K  M  F  F  F  F  I  L  F  F  S  T  L  I  S  I  S  S  N  S  W  F  G  C
1 MN793322.1    TAAATTTTTTCCCCTATAAATTCTAATAAAATATTTTTTTTTTTTATTTTATTCTTTAGAACATTAATTTCAATTTCCTCAAATTCATGATTTGGATGTT
2 MT492036.1    TAAATTTTTTCCCCTATAAATTCTAATAAAATATTTTTTTTTTTTATTTTATTCTTTAGAACATTAATTTCAATTTCCTCAAATTCATGATTTGGATGTT
3 MT492037.1    TAAATTTTTTCCCCTATAAATTCTAATAAAATATTTTTTTTTTTTATTTTATTCTTTAGAACATTAATTTCAATTTCCTCAAATTCATGATTTGGATGTT
4 MT492030.1    TAAATTTTTTCCCCTATAAATTCTAATAAAATATTTTTTTTTTTTATTTTATTCTTTAGAACATTAATTTCAATTTCCTCAAATTCATGATTTGGATGTT
5 MT492031.1    TAAATTTTTTCCCCTATAAATTCTAATAAAATATTTTTTTTTTTTATTTTATTCTTTAGAACATTAATTTCAATTTCCTCAAATTCATGATTTGGATGTT
6 MT492032.1    TAAATTTTTTCCCCTATAAATTCTAATAAAATATTTTTTTTTTTTATTTTATTCTTTAGAACATTAATTTCAATTTCCTCAAATTCATGATTTGGATGTT
7 MT492033.1    TAAATTTTTTCCCCTATAAATTCTAATAAAATATTTTTTTTTTTTATTTTATTCTTTAGAACATTAATTTCAATTTCCTCAAATTCATGATTTGGATGTT
8 MT492034.1    TAAATTTTTTCCCCTATAAATTCTAATAAAATATTTTTTTTTTTTATTTTATTCTTTAGAACATTAATTTCAATTTCCTCAAATTCATGATTTGGATGTT
9 MT492035.1    TAAATTTTTTCCCCTATAAATTCTAATAAAATATTTTTTTTTTTTATTTTATTCTTTAGAACATTAATTTCAATTTCCTCAAATTCATGATTTGGATGTT

             601         .         .         .         .         :         .         .         .         .        7 700
                W  I  G  L  E  I  N  L  L  S  F  I  P  I  I  N  N  S  N  N  I  L  T  S  E  A  S  L  K  Y  F  F  T  Q
1 MN793322.1    GAATTGGATTAGAAATTAATTTATTAAGATTTATTCCAATTATTAATAATTCTAATAATATTTTAACATCTGAAGCATCATTAAAATATTTTTTTACTCA
2 MT492036.1    GAATTGGATTAGAAATTAATTTATTAAGATTTATTCCAATTATTAATAATTCTAATAATATTTTAACATCTGAAGCATCATTAAAATATTTTTTTACTCA
3 MT492037.1    GAATTGGATTAGAAATTAATTTATTAAGATTTATTCCAATTATTAATAATTCTAATAATATTTTAACATCTGAAGCATCATTAAAATATTTTTTTACTCA
4 MT492030.1    GAATTGGATTAGAAATTAATTTATTAAGATTTATTCCAATTATTAATAATTCTAATAATATTTTAACATCTGAAGCATCATTAAAATATTTTTTTACTCA
5 MT492031.1    GAATTGGATTAGAAATTAATTTATTAAGATTTATTCCAATTATTAATAATTCTAATAATATTTTAACATCTGAAGCATCATTAAAATATTTTTTTACTCA
6 MT492032.1    GAATTGGATTAGAAATTAATTTATTAAGATTTATTCCAATTATTAATAATTCTAATAATATTTTAACATCTGAAGCATCATTAAAATATTTTTTTACTCA
7 MT492033.1    GAATTGGATTAGAAATTAATTTATTAAGATTTATTCCAATTATTAATAATTCTAATAATATTTTAACATCTGAAGCATCATTAAAATATTTTTTTACTCA
8 MT492034.1    GAATTGGATTAGAAATTAATTTATTAAGATTTATTCCAATTATTAATAATTCTAATAATATTTTAACATCTGAAGCATCATTAAAATATTTTTTTACTCA
9 MT492035.1    GAATTGGATTAGAAATTAATTTATTAAGATTTATTCCAATTATTAATAATTCTAATAATATTTTAACATCTGAAGCATCATTAAAATATTTTTTTACTCA

             701         .         .         .         .         :         .         .         .         .        8 800
                  A  I  A  S  I  N  L  L  F  C  I  I  I  K  M  M  F  M  K  N  F  E  M  N  N  T  I  M  I  M  M  N  S
1 MN793322.1    AGCTATTGCATCAATTAATTTATTATTTTGTATTATTATTAAAATAATATTTATAAAAAATTTTGAAATAAATAATACTATTATAATTATAATAAATTCT
2 MT492036.1    AGCTATTGCATCAATTAATTTATTATTTTGTATTATTATTAAAATAATATTTATAAAAAATTTTGAAATAAATAATACTATTATAATTATAATAAATTCT
3 MT492037.1    AGCTATTGCATCAATTAATTTATTATTTTGTATTATTATTAAAATAATATTTATAAAAAATTTTGAAATAAATAATACTATTATAATTATAATAAATTCT
4 MT492030.1    AGCTATTGCATCAATTAATTTATTATTTTGTATTATTATTAAAATAATATTTATAAAAAATTTTGAAATAAATAATACTATTATAATTATAATAAATTCT
5 MT492031.1    AGCTATTGCATCAATTAATTTATTATTTTGTATTATTATTAAAATAATATTTATAAAAAATTTTGAAATAAATAATACTATTATAATTATAATAAATTCT
6 MT492032.1    AGCTATTGCATCAATTAATTTATTATTTTGTATTATTATTAAAATAATATTTATAAAAAATTTTGAAATAAATAATACTATTATAATTATAATAAATTCT
                  A  I  A  S  I  N  L  L  F  C  I  I  I  K  M  M  L  M  K  N  F  E  M  N  N  T  I  M  I  M  M  N  S
7 MT492033.1    AGCTATTGCATCAATTAATTTATTATTTTGTATTATTATTAAAATAATATTAATAAAAAATTTTGAAATAAATAATACTATTATAATTATAATAAATTCT
8 MT492034.1    AGCTATTGCATCAATTAATTTATTATTTTGTATTATTATTAAAATAATATTTATAAAAAATTTTGAAATAAATAATACTATTATAATTATAATAAATTCT
9 MT492035.1    AGCTATTGCATCAATTAATTTATTATTTTGTATTATTATTAAAATAATATTTATAAAAAATTTTGAAATAAATAATACTATTATAATTATAATAAATTCT
```

```
              801          .           .           .           .           :           .           .           .           .          9 900
                  S  L  L  M  K  M  G  S  A  P  F  H  F  W  F  P  N  I  I  E  G  L  S  W  F  N  S  F  I  L  M  T  W
1 MN793322.1     TCTTTATTAATAAAAATAGGATCAGCCCCCTTTCATTTCTGATTCCCAAATATTATTGAAGGATTATCATGATTTAATAGATTTATTTTAATAACTTGAC
2 MT492036.1     TCTTTATTAATAAAAATAGGATCAGCCCCCTTTCATTTCTGATTCCCAAATATTATTGAAGGATTATCATGATTTAATAGATTTATTTTAATAACTTGAC
3 MT492037.1     TCTTTATTAATAAAAATAGGATCAGCCCCCTTTCATTTCTGATTCCCAAATATTATTGAAGGATTATCATGATTTAATAGATTTATTTTAATAACTTGAC
4 MT492030.1     TCTTTATTAATAAAAATAGGATCAGCCCCCTTTCATTTCTGATTCCCAAATATTATTGAAGGATTATCATGATTTAATAGATTTATTTTAATAACTTGAC
5 MT492031.1     TCTTTATTAATAAAAATAGGATCAGCCCCCTTTCATTTCTGATTCCCAAATATTATTGAAGGATTATCATGATTTAATAGATTTATTTTAATAACTTGAC
6 MT492032.1     TCTTTATTAATAAAAATAGGATCAGCCCCCTTTCATTTCTGATTCCCAAATATTATTGAAGGATTATCATGATTTAATAGATTTATTTTAATAACTTGAC
7 MT492033.1     TCTTTATTAATAAAAATAGGATCAGCCCCCTTTCATTTCTGATTCCCAAATATTATTGAAGGATTATCATGATTTAATAGATTTATTTTAATAACTTGAC
8 MT492034.1     TCTTTATTAATAAAAATAGGATCAGCCCCCTTTCATTTCTGATTCCCAAATATTATTGAAGGATTATCATGATTTAATAGATTTATTTTAATAACTTGAC
9 MT492035.1     TCTTTATTAATAAAAATAGGATCAGCCCCCTTTCATTTCTGATTCCCAAATATTATTGAAGGATTATCATGATTTAATAGATTTATTTTAATAACTTGAC

              901          .           .           .           .           :           .           .           .           .          0 1000
                 Q  K  I  S  P  M  I  L  L  S  Y  W  Y  N  N  N  F  L  I  L  I  I  I  L  N  S  I  I  G  A  I  G  G  L
1 MN793322.1     AAAAAATTTCCCCTATAATTTTATTATCATACTGATACAATAATAATTTTTTAATTTTAATTATTATTTTAAATTCTATTATTGGAGCTATTGGAGGATT
2 MT492036.1     AAAAAATTTCCCCTATAATTTTATTATCATACTGATACAATAATAATTTTTTAATTTTAATTATTATTTTAAATTCTATTATTGGAGCTATTGGAGGATT
3 MT492037.1     AAAAAATTTCCCCTATAATTTTATTATCATACTGATACAATAATAATTTTTTAATTTTAATTATTATTTTAAATTCTATTATTGGAGCTATTGGAGGATT
4 MT492030.1     AAAAAATTTCCCCTATAATTTTATTATCATACTGATACAATAATAATTTTTTAATTTTAATTATTATTTTAAATTCTATTATTGGAGCTATTGGAGGATT
5 MT492031.1     AAAAAATTTCCCCTATAATTTTATTATCATACTGATACAATAATAATTTTTTAATTTTAATTATTATTTTAAATTCTATTATTGGAGCTATTGGAGGATT
6 MT492032.1     AAAAAATTTCCCCTATAATTTTATTATCATACTGATACAATAATAATTTTTTAATTTTAATTATTATTTTAAATTCTATTATTGGAGCTATTGGAGGATT
7 MT492033.1     AAAAAATTTCCCCTATAATTTTATTATCATACTGATACAATAATAATTTTTTAATTTTAATTATTATTTTAAATTCTATTATTGGAGCTATTGGAGGATT
8 MT492034.1     AAAAAATTTCCCCTATAATTTTATTATCATACTGATACAATAATAATTTTTTAATTTTAATTATTATTTTAAATTCTATTATTGGAGCTATTGGAGGATT
9 MT492035.1     AAAAAATTTCCCCTATAATTTTATTATCATACTGATACAATAATAATTTTTTAATTTTAATTATTATTTTAAATTCTATTATTGGAGCTATTGGAGGATT

             1001          .           .           .           .           :           .           .           .           .          1 1100
                   N  Q  T  S  I  R  K  L  M  T  F  S  S  I  N  N  L  S  W  M  M  S  A  M  M  I  S  E  N  L  W  M  F
1 MN793322.1     AAATCAAACATCAATTCGAAAATTAATAACTTTTTCTTCTATTAATAATTTAAGATGAATAATATCTGCAATAATAATTAGAGAAAACTTATGAATATTT
2 MT492036.1     AAATCAAACATCAATTCGAAAATTAATAACTTTTTCTTCTATTAATAATTTAAGATGAATAATATCTGCAATAATAATTAGAGAAAACTTATGAATATTT
3 MT492037.1     AAATCAAACATCAATTCGAAAATTAATAACTTTTTCTTCTATTAATAATTTAAGATGAATAATATCTGCAATAATAATTAGAGAAAACTTATGAATATTT
4 MT492030.1     AAATCAAACATCAATTCGAAAATTAATAACTTTTTCTTCTATTAATAATTTAAGATGAATAATATCTGCAATAATAATTAGAGAAAACTTATGAATATTT
5 MT492031.1     AAATCAAACATCAATTCGAAAATTAATAACTTTTTCTTCTATTAATAATTTAAGATGAATAATATCTGCAATAATAATTAGAGAAAACTTATGAATATTT
6 MT492032.1     AAATCAAACATCAATTCGAAAATTAATAACTTTTTCTTCTATTAATAATTTAAGATGAATAATATCTGCAATAATAATTAGAGAAAACTTATGAATATTT
7 MT492033.1     AAATCAAACATCAATTCGAAAATTAATAACTTTTTCTTCTATTAATAATTTAAGATGAATAATATCTGCAATAATAATTAGAGAAAACTTATGAATATTT
8 MT492034.1     AAATCAAACATCAATTCGAAAATTAATAACTTTTTCTTCTATTAATAATTTAAGATGAATAATATCTGCAATAATAATTAGAGAAAACTTATGAATATTT
9 MT492035.1     AAATCAAACATCAATTCGAAAATTAATAACTTTTTCTTCTATTAATAATTTAAGATGAATAATATCTGCAATAATAATTAGAGAAAACTTATGAATATTT

             1101          .           .           .           .           :           .           .           .           .          2 1200
                  Y  F  F  M  Y  S  F  L  I  S  I  L  C  L  L  F  S  M  I  N  M  F  F  I  N  Q  L  F  F  F  N  I  N
1 MN793322.1     TATTTTTTTATATACTCATTTTTAATTAGAATTTTATGCTTATTATTTTCTATAATTAATATATTTTTTATTAATCAATTATTTTTTTTTAATATTAATT
2 MT492036.1     TATTTTTTTATATACTCATTTTTAATTAGAATTTTATGCTTATTATTTTCTATAATTAATATATTTTTTATTAATCAATTATTTTTTTTTAATATTAATT
3 MT492037.1     TATTTTTTTATATACTCATTTTTAATTAGAATTTTATGCTTATTATTTTCTATAATTAATATATTTTTTATTAATCAATTATTTTTTTTTAATATTAATT
4 MT492030.1     TATTTTTTTATATACTCATTTTTAATTAGAATTTTATGCTTATTATTTTCTATAATTAATATATTTTTTATTAATCAATTATTTTTTTTTAATATTAATT
5 MT492031.1     TATTTTTTTATATACTCATTTTTAATTAGAATTTTATGCTTATTATTTTCTATAATTAATATATTTTTTATTAATCAATTATTTTTTTTTAATATTAATT
6 MT492032.1     TATTTTTTTATATACTCATTTTTAATTAGAATTTTATGCTTATTATTTTCTATAATTAATATATTTTTTATTAATCAATTATTTTTTTTTAATATTAATT
7 MT492033.1     TATTTTTTTATATACTCATTTTTAATTAGAATTTTATGCTTATTATTTTCTATAATTAATATATTTTTTATTAATCAATTATTTTTTTTTAATATTAATT
8 MT492034.1     TATTTTTTTATATACTCATTTTTAATTAGAATTTTATGCTTATTATTTTCTATAATTAATATATTTTTTATTAATCAATTATTTTTTTTTAATATTAATT
9 MT492035.1     TATTTTTTTATATACTCATTTTTAATTAGAATTTTATGCTTATTATTTTCTATAATTAATATATTTTTTATTAATCAATTATTTTTTTTTAATATTAATT
```

```
              1201      .         .         .         .         :         .         .         .         .      3 1300
                  Y  L  I  K  L  S  L  L  I  N  F  L  S  L  G  G  L  P  P  F  M  G  F  L  P  K  W  I  I  I  N  F  M  L
1 MN793322.1     ATTTAATTAAATTATCATTATTAATTAATTTTTTATCATTGGGAGGACTTCCACCTTTTATAGGATTTTTACCTAAATGAATTATCATTAATTTTATATT
2 MT492036.1     ATTTAATTAAATTATCATTATTAATTAATTTTTTATCATTAGGAGGACTTCCACCTTTTATAGGATTTTTACCTAAATGAATTATCATTAATTTTATATT
3 MT492037.1     ATTTAATTAAATTATCATTATTAATTAATTTTTTATCATTAGGAGGACTTCCACCTTTTATAGGATTTTTACCTAAATGAATTATCATTAATTTTATATT
4 MT492030.1     ATTTAATTAAATTATCATTATTAATTAATTTTTTATCATTAGGAGGACTTCCACCTTTTATAGGATTTTTACCTAAATGAATTATCATTAATTTTATATT
5 MT492031.1     ATTTAATTAAATTATCATTATTAATTAATTTTTTATCATTAGGAGGACTTCCACCTTTTATAGGATTTTTACCTAAATGAATTATCATTAATTTTATATT
6 MT492032.1     ATTTAATTAAATTATCATTATTAATTAATTTTTTATCATTAGGAGGACTTCCACCTTTTATAGGATTTTTACCTAAATGAATTATCATTAATTTTATATT
7 MT492033.1     ATTTAATTAAATTATCATTATTAATTAATTTTTTATCATTAGGAGGACTTCCACCTTTTATAGGATTTTTACCTAAATGAATTATCATTAATTTTATATT
8 MT492034.1     ATTTAATTAAATTATCATTATTAATTAATTTTTTATCATTAGGAGGACTTCCACCTTTTATAGGATTTTTACCTAAATGAATTATCATTAATTTTATATT
9 MT492035.1     ATTTAATTAAATTATCATTATTAATTAATTTTTTATCATTAGGAGGACTTCCACCTTTTATAGGATTTTTACCTAAATGAATTATCATTAATTTTATATT


              1301      .         .         .         .         :         .         .         .         .      4 1400
                    N  S  N  L  Y  F  L  T  F  I  L  I  M  M  S  L  I  L  L  F  F  Y  I  R  I  L  Y  S  S  F  M  F  N
1 MN793322.1     AAATAGAAATCTTTATTTTTTAACTTTTATTTTAATTATAATAAGATTAATTTTATTATTTTTTTATATTCGAATTTTATATTCATCATTTATATTTAAT
2 MT492036.1     AAATAGAAATCTTTATTTTTTAACTTTTATTTTAATTATAATAAGATTAATTTTATTATTTTTTTATATTCGAATTTTATATTCATCATTTATATTTAAT
3 MT492037.1     AAATAGAAATCTTTATTTTTTAACTTTTATTTTAATTATAATAAGATTAATTTTATTATTTTTTTATATTCGAATTTTATATTCATCATTTATATTTAAT
4 MT492030.1     AAATAGAAATCTTTATTTTTTAACTTTTATTTTAATTATAATAAGATTAATTTTATTATTTTTTTATATTCGAATTTTATATTCATCATTTATATTTAAT
5 MT492031.1     AAATAGAAATCTTTATTTTTTAACTTTTATTTTAATTATAATAAGATTAATTTTATTATTTTTTTATATTCGAATTTTATATTCATCATTTATATTTAAT
6 MT492032.1     AAATAGAAATCTTTATTTTTTAACTTTTATTTTAATTATAATAAGATTAATTTTATTATTTTTTTATATTCGAATTTTATATTCATCATTTATATTTAAT
7 MT492033.1     AAATAGAAATCTTTATTTTTTAACTTTTATTTTAATTATAATAAGATTAATTTTATTATTTTTTTATATTCGAATTTTATATTCATCATTTATATTTAAT
8 MT492034.1     AAATAGAAATCTTTATTTTTTAACTTTTATTTTAATTATAATAAGATTAATTTTATTATTTTTTTATATTCGAATTTTATATTCATCATTTATATTTAAT
9 MT492035.1     AAATAGAAATCTTTATTTTTTAACTTTTATTTTAATTATAATAAGATTAATTTTATTATTTTTTTATATTCGAATTTTATATTCATCATTTATATTTAAT


              1401      .         .         .         .         :         .         .         .         .      5 1500
                   Y  L  K  L  K  W  I  K  I  K  I  K  N  N  M  F  N  I  M  N  F  L  S  F  F  S  I  L  G  L  I  T  S
1 MN793322.1     TATTTAAAATTAAAATGAATTAAAATCAAAATTAAAAATAATATATTTAACATTATAAATTTCTTATCTTTTTTCTCTATTTTAGGGTTAATTACAAGAA
2 MT492036.1     TATTTAAAATTAAAATGAATTAAAATCAAAATTAAAAATAATATATTTAACATTATAAATTTCTTATCTTTTTTCTCTATTTTAGGGTTAATTACAAGAA
3 MT492037.1     TATTTAAAATTAAAATGAATTAAAATCAAAATTAAAAATAATATATTTAACATTATAAATTTCTTATCTTTTTTCTCTATTTTAGGGTTAATTACAAGAA
4 MT492030.1     TATTTAAAATTAAAATGAATTAAAATCAAAATTAAAAATAATATATTTAACATTATAAATTTCTTATCTTTTTTCTCTATTTTAGGGTTAATTACAAGAA
5 MT492031.1     TATTTAAAATTAAAATGAATTAAAATCAAAATTAAAAATAATATATTTAACATTATAAATTTCTTATCTTTTTTCTCTATTTTAGGGTTAATTACAAGAA
6 MT492032.1     TATTTAAAATTAAAATGAATTAAAATCAAAATTAAAAATAATATATTTAACATTATAAATTTCTTATCTTTTTTCTCTATTTTAGGGTTAATTACAAGAA
7 MT492033.1     TATTTAAAATTAAAATGAATTAAAATCAAAATTAAAAATAATATATTTAACATTATAAATTTCTTATCTTTTTTCTCTATTTTAGGGTTAATTACAAGAA
8 MT492034.1     TATTTAAAATTAAAATGAATTAAAATCAAAATTAAAAATAATATATTTAACATTATAAATTTCTTATCTTTTTTCTCTATTTTAGGGTTAATTACAAGAA
9 MT492035.1     TATTTAAAATTAAAATGAATTAAAATCAAAATTAAAAATAATATATTTAACATTATAAATTTCTTATCTTTTTTCTCTATTTTAGGGTTAATTACAAGAA


                                         | tRNA-Trp ->                                                  |
              1501      .         .         .         .         :         .         .         .         .      6 1600
                 T  F  F  F  M  *        aaggttttaagttaaattaaactaataatcttcaaaattatttataaagaaaattctttaagcctta
1 MN793322.1     CTTTTTTTTTTTATATAAAATTATTAAGGTTTTAAGTTAAATTAAACTAATAATCTTCAAAATTATTTATAAAGAAAATTCTTTAAGCCTTAATAATTTTA
2 MT492036.1     CTTTTTTTTTTTATATAAAATTATTAAGGTTTTAAGTTAAATTAAACTAATAATCTTCAAAATTATTTATAAAGAAAATTCTTTAAGCCTTAATAATTTTA
3 MT492037.1     CTTTTTTTTTTTATATAAAATTATTAAGGTTTTAAGTTAAATTAAACTAATAATCTTCAAAATTATTTATAAAGAAAATTCTTTAAGCCTTAATAATTTTA
4 MT492030.1     CTTTTTTTTTTTATATAAAATTATTAAGGTTTTAAGTTAAATTAAACTAATAATCTTCAAAATTATTTATAAAGAAAATTCTTTAAGCCTTAATAATTTTA
5 MT492031.1     CTTTTTTTTTTTATATAAAATTATTAAGGTTTTAAGTTAAATTAAACTAATAATCTTCAAAATTATTTATAAAGAAAATTCTTTAAGCCTTAATAATTTTA
6 MT492032.1     CTTTTTTTTTTTATATAAAATTATTAAGGTTTTAAGTTAAATTAAACTAATAATCTTCAAAATTATTTATAAAGAAAATTCTTTAAGCCTTAATAATTTTA
7 MT492033.1     CTTTTTTTTTTTATATAAAATTATTAAGGTTTTAAGTTAAATTAAACTAATAATCTTCAAAATTATTTATAAAGAAAATTCTTTAAGCCTTAATAATTTTA
8 MT492034.1     CTTTTTTTTTTTATATAAAATTATTAAGGTTTTAAGTTAAATTAAACTAATAATCTTCAAAATTATTTATAAAGAAAATTCTTTAAGCCTTAATAATTTTA
9 MT492035.1     CTTTTTTTTTTTATATAAAATTATTAAGGTTTTAAGTTAAATTAAACTAATAATCTTCAAAATTATTTATAAAGAAAATTCTTTAAGCCTTAATAATTTTA
                                                                                      ttcggaattattaaaat
```

```
            1601         .         .         .         .         :         .         .         .         .       7 1700
1 MN793322.1    TTTATACCTTAAAATTTGCAATTTTACATCATAATTGAATATAAGACTTAATAAAAAAGAATTAATTCTTGTTAATAAATTTACAATTTATCGCTTAAAT
2 MT492036.1    TTTATACCTTAAAATTTGCAATTTTACATCATAATTGAATATAAGACTTAATAAAAAAGAATTAATTCTTGTTAATAAATTTACAATTTATCGCTTAAAT
3 MT492037.1    TTTATACCTTAAAATTTGCAATTTTACATCATAATTGAATATAAGACTTAATAAAAAAGAATTAATTCTTGTTAATAAATTTACAATTTATCGCTTAAAT
4 MT492030.1    TTTATACCTTAAAATTTGCAATTTTACATCATAATTGAATATAAGACTTAATAAAAAAGAATTAATTCTTGTTAATAAATTTACAATTTATCGCTTAAAT
5 MT492031.1    TTTATACCTTAAAATTTGCAATTTTACATCATAATTGAATATAAGACTTAATAAAAAAGAATTAATTCTTGTTAATAAATTTACAATTTATCGCTTAAAT
6 MT492032.1    TTTATACCTTAAAATTTGCAATTTTACATCATAATTGAATATAAGACTTAATAAAAAAGAATTAATTCTTGTTAATAAATTTACAATTTATCGCTTAAAT
7 MT492033.1    TTTATACCTTAAAATTTGCAATTTTACATCATAATTGAATATAAGACTTAATAAAAAAGAATTAATTCTTGTTAATAAATTTACAATTTATCGCTTAAAT
8 MT492034.1    TTTATACCTTAAAATTTGCAATTTTACATCATAATTGAATATAAGACTTAATAAAAAAGAATTAATTCTTGTTAATAAATTTACAATTTATCGCTTAAAT
9 MT492035.1    TTTATACCTTAAAATTTGCAATTTTACATCATAATTGAATATAAGACTTAATAAAAAAGAATTAATTCTTGTTAATAAATTTACAATTTATCGCTTAAAT
                Aaatatggaattttaaacgttaaaatgtagtattaacttatattctgaattatttttttcttaattaagaacaattatttaaatgttaaatagcgaattta
                                                    <- tRNA-Cys |

                                        | COI ->
            1701         .         .         .         .         :         .         .         .         .       8 1800
                                         R  K  W  L  Y  S  T  N  H  K  D  I  G  T  L  Y  F  I  F  G  I  W  S  G  M
1 MN793322.1    CTCAGCCATTTTATTTTAATT-AGCGAAAATGACTATATTCAACAAATCATAAAGATATTGGAACTTTATATTTTATTTTTGGAATTTGAAGAGGAATAG
2 MT492036.1    CTCAGCCATTTTATTTTAATT-AGCGAAAATGACTATATTCAACAAATCATAAAGATATTGGAACTTTATATTTTATTTTTGGAATTTGAAGAGGAATAG
3 MT492037.1    CTCAGCCATTTTATTTTAATTTAGCGAAAATGACTATATTCAACAAATCATAAAGATATTGGAACTTTATATTTTATTTTTGGAATTTGAAGAGGAATAG
4 MT492030.1    CTCAGCCATTTTATTTTAATT-AGCGAAAATGACTATATTCAACAAATCATAAAGATATTGGAACTTTATATTTTATTTTTGGAATTTGAAGAGGAATAG
5 MT492031.1    CTCAGCCATTTTATTTTAATTTAGCGAAAATGACTATATTCAACAAATCATAAAGATATTGGAACTTTATATTTTATTTTTGGAATTTGAAGAGGAATAG
6 MT492032.1    CTCAGCCATTTTATTTTAATT-AGCGAAAATGACTATATTCAACAAATCATAAAGATATTGGAACTTTATATTTTATTTTTGGAATTTGAAGAGGAATAG
7 MT492033.1    CTCAGCCATTTTATTTTAATT-AGCGAAAATGACTATATTCAACAAATCATAAAGATATTGGAACTTTATATTTTATTTTTGGAATTTGAAGAGGAATAG
8 MT492034.1    CTCAGCCATTTTATTTTAATT-AGCGAAAATGACTATATTCAACAAATCATAAAGATATTGGAACTTTATATTTTATTTTTGGAATTTGAAGAGGAATAG
9 MT492035.1    CTCAGCCATTTTATTTTAATT-AGCGAAAATGACTATATTCAACAAATCATAAAGATATTGGAACTTTATATTTTATTTTTGGAATTTGAAGAGGAATAG
                gagtcggtaaaataa
                 <-  tRNA-Tyr |

            1801         .         .         .         .         :         .         .         .         .       9 1900
                V  G  T  S  L  S  L  L  I  R  A  E  L  G  N  P  G  S  L  I  G  D  D  Q  I  Y  N  T  I  V  T  A  H  A
1 MN793322.1    TAGGAACTTCTCTAAGTTTATTAATTCGAGCTGAATTAGGTAATCCTGGATCATTAATTGGAGATGATCAAATTTATAATACAATTGTAACAGCTCATGC
2 MT492036.1    TAGGAACTTCTCTAAGTTTATTAATTCGAGCTGAATTAGGTAATCCTGGATCATTAATTGGAGATGATCAAATTTATAATACAATTGTAACAGCTCATGC
3 MT492037.1    TAGGAACTTCTCTAAGTTTATTAATTCGAGCTGAATTAGGTAATCCTGGATCATTAATTGGAGATGATCAAATTTATAATACAATTGTAACAGCTCATGC
4 MT492030.1    TAGGAACTTCTCTAAGTTTATTAATTCGAGCTGAATTAGGTAATCCTGGATCATTAATTGGAGATGATCAAATTTATAATACAATTGTAACAGCTCATGC
5 MT492031.1    TAGGAACTTCTCTAAGTTTATTAATTCGAGCTGAATTAGGTAATCCTGGATCATTAATTGGAGATGATCAAATTTATAATACAATTGTAACAGCTCATGC
6 MT492032.1    TAGGAACTTCTCTAAGTTTATTAATTCGAGCTGAATTAGGTAATCCTGGATCATTAATTGGAGATGATCAAATTTATAATACAATTGTAACAGCTCATGC
7 MT492033.1    TAGGAACTTCTCTAAGTTTATTAATTCGAGCTGAATTAGGTAATCCTGGATCATTAATTGGAGATGATCAAATTTATAATACAATTGTAACAGCTCATGC
8 MT492034.1    TAGGAACTTCTCTAAGTTTATTAATTCGAGCTGAATTAGGTAATCCTGGATCATTAATTGGAGATGATCAAATTTATAATACAATTGTAACAGCTCATGC
9 MT492035.1    TAGGAACTTCTCTAAGTTTATTAATTCGAGCTGAATTAGGTAATCCTGGATCATTAATTGGAGATGATCAAATTTATAATACAATTGTAACAGCTCATGC

            1901         .         .         .         .         :         .         .         .         .       0 2000
                  F  I  M  I  F  F  M  V  M  P  I  M  I  G  G  F  G  N  W  L  V  P  L  M  L  G  A  P  D  M  A  F  P
1 MN793322.1    ATTTATTATAATTTTTTTTATAGTAATACCTATTATAATTGGTGGATTTGGAAATTGATTAGTACCTCTAATATTAGGAGCTCCTGATATAGCATTCCCA
2 MT492036.1    ATTTATTATAATTTTTTTTATAGTAATACCTATTATAATTGGTGGATTTGGAAATTGATTAGTACCTCTAATATTAGGAGCTCCTGATATAGCATTCCCA
3 MT492037.1    ATTTATTATAATTTTTTTTATAGTAATACCTATTATAATTGGTGGATTTGGAAATTGATTAGTACCTCTAATATTAGGAGCTCCTGATATAGCATTCCCA
4 MT492030.1    ATTTATTATAATTTTTTTTATAGTAATACCTATTATAATTGGTGGATTTGGAAATTGATTAGTACCTCTAATATTAGGAGCTCCTGATATAGCATTCCCA
5 MT492031.1    ATTTATTATAATTTTTTTTATAGTAATACCTATTATAATTGGTGGATTTGGAAATTGATTAGTACCTCTAATATTAGGAGCTCCTGATATAGCATTCCCA
6 MT492032.1    ATTTATTATAATTTTTTTTATAGTAATACCTATTATAATTGGTGGATTTGGAAATTGATTAGTACCTCTAATATTAGGAGCTCCTGATATAGCATTCCCA
7 MT492033.1    ATTTATTATAATTTTTTTTATAGTAATACCTATTATAATTGGTGGATTTGGAAATTGATTAGTACCTCTAATATTAGGAGCTCCTGATATAGCATTCCCA
8 MT492034.1    ATTTATTATAATTTTTTTTATAGTAATACCTATTATAATTGGTGGATTTGGAAATTGATTAGTACCTCTAATATTAGGAGCTCCTGATATAGCATTCCCA
9 MT492035.1    ATTTATTATAATTTTTTTTATAGTAATACCTATTATAATTGGTGGATTTGGAAATTGATTAGTACCTCTAATATTAGGAGCTCCTGATATAGCATTCCCA
```

```
           2001      .         .         .         .         :         .         .         .         .        1 2100
               R  M  N  N  M  S  F  W  L  L  P  P  S  L  T  L  L  I  S  S  S  I  V  E  N  G  A  G  T  G  W  T  V
1 MN793322.1    CGAATAAATAATATAAGATTTTGATTATTACCCCCATCATTAACCCTTTTAATTTCAAGAAGAATCGTTGAAAATGGAGCAGGAACTGGTTGAACTGTTT
2 MT492036.1    CGAATAAATAATATAAGATTTTGATTATTACCCCCATCATTAACCCTTTTAATTTCAAGAAGAATCGTTGAAAATGGAGCAGGAACTGGTTGAACTGTTT
3 MT492037.1    CGAATAAATAATATAAGATTTTGATTATTACCCCCATCATTAACCCTTTTAATTTCAAGAAGAATCGTTGAAAATGGAGCAGGAACTGGTTGAACTGTTT
4 MT492030.1    CGAATAAATAATATAAGATTTTGATTATTACCCCCATCATTAACCCTTTTAATTTCAAGAAGAATCGTTGAAAATGGAGCAGGAACTGGTTGAACTGTTT
5 MT492031.1    CGAATAAATAATATAAGATTTTGATTATTACCCCCATCATTAACCCTTTTAATTTCAAGAAGAATCGTTGAAAATGGAGCAGGAACTGGTTGAACTGTTT
6 MT492032.1    CGAATAAATAATATAAGATTTTGATTATTACCCCCATCATTAACCCTTTTAATTTCAAGAAGAATCGTTGAAAATGGAGCAGGAACTGGTTGAACTGTTT
7 MT492033.1    CGAATAAATAATATAAGATTTTGATTATTACCCCCATCATTAACCCTTTTAATTTCAAGAAGAATCGTTGAAAATGGAGCAGGAACTGGTTGAACTGTTT
8 MT492034.1    CGAATAAATAATATAAGATTTTGATTATTACCCCCATCATTAACCCTTTTAATTTCAAGAAGAATCGTTGAAAATGGAGCAGGAACTGGTTGAACTGTTT
9 MT492035.1    CGAATAAATAATATAAGATTTTGATTATTACCCCCATCATTAACCCTTTTAATTTCAAGAAGAATCGTTGAAAATGGAGCAGGAACTGGTTGAACTGTTT

           2101      .         .         .         .         :         .         .         .         .        2 2200
              Y  P  P  L  S  S  N  I  A  H  G  G  S  S  V  D  L  A  I  F  S  L  H  L  A  G  I  S  S  I  L  G  A  I
1 MN793322.1    ACCCCCCACTTTCATCTAATATTGCTCATGGAGGTAGATCTGTAGACCTAGCTATTTTTTCATTACATTTAGCTGGTATTTCTTCAATTTTAGGAGCAAT
2 MT492036.1    ACCCCCCACTTTCATCTAATATTGCTCATGGAGGTAGATCTGTAGACCTAGCTATTTTTTCATTACATTTAGCTGGTATTTCTTCAATTTTAGGAGCAAT
3 MT492037.1    ACCCCCCACTTTCATCTAATATTGCTCATGGAGGTAGATCTGTAGACCTAGCTATTTTTTCATTACATTTAGCTGGTATTTCTTCAATTTTAGGAGCAAT
4 MT492030.1    ACCCCCCACTTTCATCTAATATTGCTCATGGAGGTAGATCTGTAGACCTAGCTATTTTTTCATTACATTTAGCTGGTATTTCTTCAATTTTAGGAGCAAT
5 MT492031.1    ACCCCCCACTTTCATCTAATATTGCTCATGGAGGTAGATCTGTAGACCTAGCTATTTTTTCATTACATTTAGCTGGTATTTCTTCAATTTTAGGAGCAAT
6 MT492032.1    ACCCCCCACTTTCATCTAATATTGCTCATGGAGGTAGATCTGTAGACCTAGCTATTTTTTCATTACATTTAGCTGGTATTTCTTCAATTTTAGGAGCAAT
7 MT492033.1    ACCCCCCACTTTCATCTAATATTGCTCATGGAGGTAGATCTGTAGACCTAGCTATTTTTTCATTACATTTAGCTGGTATTTCTTCAATTTTAGGAGCAAT
8 MT492034.1    ACCCCCCACTTTCATCTAATATTGCTCATGGAGGTAGATCTGTAGACCTAGCTATTTTTTCATTACATTTAGCTGGTATTTCTTCAATTTTAGGAGCAAT
9 MT492035.1    ACCCCCCACTTTCATCTAATATTGCTCATGGAGGTAGATCTGTAGACCTAGCTATTTTTTCATTACATTTAGCTGGTATTTCTTCAATTTTAGGAGCAAT

           2201      .         .         .         .         :         .         .         .         .        3 2300
                N  F  I  T  T  I  I  N  M  R  I  N  G  M  S  F  D  Q  M  P  L  F  V  W  S  V  G  I  T  A  L  L  L
1 MN793322.1    TAACTTCATTACAACAATTATTAACATACGAATTAACGGAATATCTTTTGATCAAATACCATTATTTGTATGATCTGTAGGAATTACAGCATTATTATTA
2 MT492036.1    TAACTTCATTACAACAATTATTAACATACGAATTAACGGAATATCTTTTGATCAAATACCATTATTTGTATGATCTGTAGGAATTACAGCATTATTATTA
3 MT492037.1    TAACTTCATTACAACAATTATTAACATACGAATTAACGGAATATCTTTTGATCAAATACCATTATTTGTATGATCTGTAGGAATTACAGCATTATTATTA
4 MT492030.1    TAACTTCATTACAACAATTATTAACATACGAATTAACGGAATATCTTTTGATCAAATACCATTATTTGTATGATCTGTAGGAATTACAGCATTATTATTA
5 MT492031.1    TAACTTCATTACAACAATTATTAACATACGAATTAACGGAATATCTTTTGATCAAATACCATTATTTGTATGATCTGTAGGAATTACAGCATTATTATTA
6 MT492032.1    TAACTTCATTACAACAATTATTAACATACGAATTAACGGAATATCTTTTGATCAAATACCATTATTTGTATGATCTGTAGGAATTACAGCATTATTATTA
7 MT492033.1    TAACTTCATTACAACAATTATTAACATACGAATTAACGGAATATCTTTTGATCAAATACCATTATTTGTATGATCTGTAGGAATTACAGCATTATTATTA
8 MT492034.1    TAACTTCATTACAACAATTATTAACATACGAATTAACGGAATATCTTTTGATCAAATACCATTATTTGTATGATCTGTAGGAATTACAGCATTATTATTA
9 MT492035.1    TAACTTCATTACAACAATTATTAACATACGAATTAACGGAATATCTTTTGATCAAATACCATTATTTGTATGATCTGTAGGAATTACAGCATTATTATTA

           2301      .         .         .         .         :         .         .         .         .        4 2400
               L  L  S  L  P  V  L  A  G  A  I  T  M  L  L  T  D  R  N  L  N  T  S  F  F  D  P  A  G  G  G  D  P
1 MN793322.1    TTATTATCACTTCCTGTTTTAGCTGGAGCTATCACTATATTATTAACAGATCGAAATTTAAATACATCATTTTTTGATCCTGCTGGAGGGGGAGATCCTA
2 MT492036.1    TTATTATCACTTCCTGTTTTAGCTGGAGCTATCACTATATTATTAACAGATCGAAATTTAAATACATCATTTTTTGATCCTGCTGGAGGGGGAGATCCTA
3 MT492037.1    TTATTATCACTTCCTGTTTTAGCTGGAGCTATCACTATATTATTAACAGATCGAAATTTAAATACATCATTTTTTGATCCTGCTGGAGGGGGAGATCCTA
4 MT492030.1    TTATTATCACTTCCTGTTTTAGCTGGAGCTATCACTATATTATTAACAGATCGAAATTTAAATACATCATTTTTTGATCCTGCTGGAGGGGGAGATCCTA
5 MT492031.1    TTATTATCACTTCCTGTTTTAGCTGGAGCTATCACTATATTATTAACAGATCGAAATTTAAATACATCATTTTTTGATCCTGCTGGAGGGGGAGATCCTA
6 MT492032.1    TTATTATCACTTCCTGTTTTAGCTGGAGCTATCACTATATTATTAACAGATCGAAATTTAAATACATCATTTTTTGATCCTGCTGGAGGGGGAGATCCTA
7 MT492033.1    TTATTATCACTTCCTGTTTTAGCTGGAGCTATCACTATATTATTAACAGATCGAAATTTAAATACATCATTTTTTGATCCTGCTGGAGGGGGAGATCCTA
8 MT492034.1    TTATTATCACTTCCTGTTTTAGCTGGAGCTTTCACTATATTATTAACAGATCGAAATTTAAATACATCATTTTTTGATCCTGCTGGAGGGGGAGATCCTA
9 MT492035.1    TTATTATCACTTCCTGTTTTAGCTGGAGCTATCACTATATTATTAACAGATCGAAATTTAAATACATCATTTTTTGATCCTGCTGGAGGGGGAGATCCTA
```

```
               2401      .         .         .         .         :         .         .         .         .      5 2500
                   I  L  Y  Q  H  L  F  W  F  F  G  H  P  E  V  Y  I  L  I  L  P  G  F  G  M  I  S  H  I  I   S  Q  E
1 MN793322.1       TTTTATATCAACATTTATTTTGATTTTTTGGACATCCAGAAGTTTATATTTAATTTTACCAGGATTTGGTATAATTTCACATATTATT-TCACAAGAAA
2 MT492036.1       TTTTATATCAACATTTATTTTGATTTTTTGGACATCCAGAAGTTTATATTTAATTTTACCAGGATTTGGTATAATTTCACATATTATT-TCACAAGAAA
3 MT492037.1       TTTTATATCAACATTTATTTTGATTTTTTGGACATCCAGAAGTTTATATTTAATTTTACCAGGATTTGGTATAATTTCACATATTATT-TCACAAGAAA
4 MT492030.1       TTTTATATCAACATTTATTTTGATTTTTTGGACATCCAGAAGTTTATATTTAATTTTACCAGGATTTGGTATAATTTCACATATTATT-TCACAAGAAA
                                                                                          S   M  L  L  S
5 MT492031.1       TTTTATATCAACATTTATTTTGATTTTTTGGACATCCAGAAGTTTATATTTAATTTTACCAGGATTTGGTATAATTTC-CATATTATTATCACAAGAAA
6 MT492032.1       TTTTATATCAACATTTATTTTGATTTTTTGGACATCCAGAAGTTTATATTTAATTTTACCAGGATTTGGTATAATTTC-CATATTATTATCACAAGAAA
7 MT492033.1       TTTTATATCAACATTTATTTTGATTTTTTGGACATCCAGAAGTTTATATTTAATTTTACCAGGATTTGGTATAATTTC-CATATTATTATCACAAGAAA
8 MT492034.1       TTTTATATCAACATTTATTTTGATTTTTTGGTCATCCAGAAGTTTATATTTAATTTTACCAGGATTTGGTATAATTTC-CATATTATTATCACAAGAAA
9 MT492035.1       TTTTATATCAACATTTATTTTGATTTTTTGGACATCCAGAAGTTTATATTTAATTTTACCAGGATTTGGTATAATTTCACATATTATT-TCACAAGAAA

               2501      .         .         .         .         :         .         .         .         .      6 2600
                  S  G  K  K  E  T  F  G  S  L  G  M  I  Y  A  M  M  A  I  G  L  L  G  F  V  V  W  A  H  H  M  F  T  V
1 MN793322.1       GAGGAAAAAAAGAAACATTTGGATCTTTAGGAATAATTTATGCTATAATAGCAATTGGTTTATTAGGATTTGTAGTATGAGCTCATCATATATTTACAGT
2 MT492036.1       GAGGAAAAAAAGAAACATTTGGATCTTTAGGAATAATTTATGCTATAATAGCAATTGGTTTATTAGGATTTGTAGTATGAGCTCATCATATATTTACAGT
3 MT492037.1       GAGGAAAAAAAGAAACATTTGGATCTTTAGGAATAATTTATGCTATAATAGCAATTGGTTTATTAGGATTTGTAGTATGAGCTCATCATATATTTACAGT
4 MT492030.1       GAGGAAAAAAAGAAACATTTGGATCTTTAGGAATAATTTATGCTATAATAGCAATTGGTTTATTAGGATTTGTAGTATGAGCTCATCATATATTTACAGT
5 MT492031.1       GAGGAAAAAAAGAAACATTTGGATCTTTAGGAATAATTTATGCTATAATAGCAATTGGTTTATTAGGATTTGTAGTATGAGCTCATCATATATTTACAGT
6 MT492032.1       GAGGAAAAAAAGAAACATTTGGATCTTTAGGAATAATTTATGCTATAATAGCAATTGGTTTATTAGGATTTGTAGTATGAGCTCATCATATATTTACAGT
7 MT492033.1       GAGGAAAAAAAGAAACATTTGGATCTTTAGGAATAATTTATGCTATAATAGCAATTGGTTTATTAGGATTTGTAGTATGAGCTCATCATATATTTACAGT
8 MT492034.1       GAGGAAAAAAAGAAACATTTGGATCTTTAGGAATAATTTATGCTATAATAGCAATTGGTTTATTAGGATTTGTAGTATGAGCTCATCATATATTTACAGT
9 MT492035.1       GAGGAAAAAAAGAAACATTTGGATCTTTAGGAATAATTTATGCTATAATAGCAATTGGTTTATTAGGATTTGTAGTATGAGCTCATCATATATTTACAGT

               2601      .         .         .         .         :         .         .         .         .      7 2700
                    G  M  D  I  D  T  R  A  Y  F  T  S  A  T  M  I  I  A  V  P  T  G  I  K  I  F  S  W  L  A  T  L  H
1 MN793322.1       AGGAATAGACATCGATACACGAGCTTACTTTACCTCAGCAACAATAATTATTGCTGTTCCAACAGGAATTAAAATTTTTAGTTGATTAGCAACCTTACAT
2 MT492036.1       AGGAATAGACATCGATACACGAGCTTACTTTACCTCAGCAACAATAATTATTGCTGTTCCAACAGGAATTAAAATTTTTAGTTGATTAGCAACCTTACAT
3 MT492037.1       AGGAATAGACATCGATACACGAGCTTACTTTACCTCAGCAACAATAATTATTGCTGTTCCAACAGGAATTAAAATTTTTAGTTGATTAGCAACCTTACAT
4 MT492030.1       AGGAATAGACATCGATACACGAGCTTACTTTACCTCAGCAACAATAATTATTGCTGTTCCAACAGGAATTAAAATTTTTAGTTGATTAGCAACCTTACAT
5 MT492031.1       AGGAATAGACATCGATACACGAGCTTACTTTACCTCAGCAACAATAATTATTGCTGTTCCAACAGGAATTAAAATTTTTAGTTGATTAGCAACCTTACAT
6 MT492032.1       AGGAATAGACATCGATACACGAGCTTACTTTACCTCAGCAACAATAATTATTGCTGTTCCAACAGGAATTAAAATTTTTAGTTGATTAGCAACCTTACAT
7 MT492033.1       AGGAATAGACATCGATACACGAGCTTACTTTACCTCAGCAACAATAATTATTGCTGTTCCAACAGGAATTAAAATTTTTAGTTGATTAGCAACCTTACAT
8 MT492034.1       AGGAATAGACATCGATACACGAGCTTACTTTACCTCAGCAACAATAATTATTGCTGTTCCAACAGGAATTAAAATTTTTAGTTGATTAGCAACCTTACAT
9 MT492035.1       AGGAATAGACATCGATACACGAGCTTACTTTACCTCAGCAACAATAATTATTGCTGTTCCAACAGGAATTAAAATTTTTAGTTGATTAGCAACCTTACAT

               2701      .         .         .         .         :         .         .         .         .      8 2800
                   G  T  Q  I  N  Y  S  P  S  I  L  W  S  L  G  F  V  F  L  F  T  V  G  G  L  T  G  V  V  L  A  N  S
1 MN793322.1       GGAACTCAAATTAATTATAGACCTTCAATTCTTTGAAGATTAGGATTTGTATTTTTATTCACTGTTGGTGGATTAACAGGAGTTGTATTAGCTAATTCAT
2 MT492036.1       GGAACTCAAATTAATTATAGACCTTCAATTCTTTGAAGATTAGGATTTGTATTTTTATTCACTGTTGGTGGATTAACAGGAGTTGTATTAGCTAATTCAT
3 MT492037.1       GGAACTCAAATTAATTATAGACCTTCAATTCTTTGAAGATTAGGATTTGTATTTTTATTCACTGTTGGTGGATTAACAGGAGTTGTATTAGCTAATTCAT
4 MT492030.1       GGAACTCAAATTAATTATAGACCTTCAATTCTTTGAAGATTAGGATTTGTATTTTTATTCACTGTTGGTGGATTAACAGGAGTTGTATTAGCTAATTCAT
5 MT492031.1       GGAACTCAAATTAATTATAGACCTTCAATTCTTTGAAGATTAGGATTTGTATTTTTATTCACTGTTGGTGGATTAACAGGAGTTGTATTAGCTAATTCAT
6 MT492032.1       GGAACTCAAATTAATTATAGACCTTCAATTCTTTGAAGATTAGGATTTGTATTTTTATTCACTGTTGGTGGATTAACAGGAGTTGTATTAGCTAATTCAT
7 MT492033.1       GGAACTCAAATTAATTATAGACCTTCAATTCTTTGAAGATTAGGATTTGTATTTTTATTCACTGTTGGTGGATTAACAGGAGTTGTATTAGCTAATTCAT
8 MT492034.1       GGAACTCAAATTAATTATAGACCTTCAATTCTTTGAAGATTAGGATTTGTATTTTTATTCACTGTTGGTGGATTAACAGGAGTTGTATTAGCTAATTCAT
9 MT492035.1       GGAACTCAAATTAATTATAGACCTTCAATTCTTTGAAGATTAGGATTTGTATTTTTATTCACTGTTGGTGGATTAACAGGAGTTGTATTAGCTAATCCAT
```

```
             2801            .         .         .         .         :         .         .         .         .         9 2900
                 S  I  D  I  A  L  H  D  T  Y  Y  V  V  A  H  F  H  Y  V  L  S  M  G  A  V  F  A  I  I  A  G  F  I  H
1 MN793322.1     CTATTGATATTGCCCTTCATGACACTTATTATGTAGTAGCCCACTTTCATTATGTATTATCTATAGGAGCTGTATTTGCTATTATTGCTGGATTTATTCA
2 MT492036.1     CTATTGATATTGCCCTTCATGACACTTATTATGTAGTAGCCCACTTTCATTATGTATTATCTATAGGAGCTGTATTTGCTATTATTGCTGGATTTATTCA
3 MT492037.1     CTATTGATATTGCCCTTCATGACACTTATTATGTAGTAGCCCACTTTCATTATGTATTATCTATAGGAGCTGTATTTGCTATTATTGCTGGATTTATTCA
4 MT492030.1     CTATTGATATTGCCCTTCATGACACTTATTATGTAGTAGCCCACTTTCATTATGTATTATCTATAGGAGCTGTATTTGCTATTATTGCTGGATTTATTCA
5 MT492031.1     CTATTGATATTGCCCTTCATGACACTTATTATGTAGTAGCCCACTTTCATTATGTATTATCTATAGGAGCTGTATTTGCTATTATTGCTGGATTTATTCA
6 MT492032.1     CTATTGATATTGCCCTTCATGACACTTATTATGTAGTAGCCCACTTTCATTATGTATTATCTATAGGAGCTGTATTTGCTATTATTGCTGGATTTATTCA
7 MT492033.1     CTATTGATATTGCCCTTCATGACACTTATTATGTAGTAGCCCACTTTCATTATGTATTATCTATAGGAGCTGTATTTGCTATTATTGCTGGATTTATTCA
8 MT492034.1     CTATTGATATTGCCCTTCATGACACTTATTATGTAGTAGCCCACTTTCATTATGTATTATCTATAGGAGCTGTATTTGCTATTATTGCTGGATTTATTCA
9 MT492035.1     CTATTGATATTGCCCTTCATGACACTTATTATGTAGTGGCCCACTTTCATTATGTATTATCTATAGGAGCTGTATTTGCTATTATTGCTGGATTTATTCA
                                                     + HaeIII (GGCC)
                                                     Ref position 2,554

             2901            .         .         .         .         :         .         .         .         .         0 3000
                  W  Y  P  L  F  T  G  L  S  L  N  P  Y  F  L  K  I  Q  F  F  T  M  F  I  G  V  N  L  T  F  F  P  Q
1 MN793322.1     CTGATATCCCCTATTTACAGGATTAAGCTTAAATCCTTATTTTTTAAAAATTCAATTTTTTACAATATTTATTGGAGTAAATTTAACTTTCTTCCCACAA
2 MT492036.1     CTGATATCCCCTATTTACAGGATTAAGCTTAAATCCTTATTTTTTAAAAATTCAATTTTTTACAATATTTATTGGAGTAAATTTAACTTTCTTCCCACAA
3 MT492037.1     CTGATATCCCCTATTTACAGGATTAAGCTTAAATCCTTATTTTTTAAAAATTCAATTTTTTACAATATTTATTGGAGTAAATTTAACTTTCTTCCCACAA
4 MT492030.1     CTGATATCCCCTATTTACAGGATTAAGCTTAAATCCTTATTTTTTAAAAATTCAATTTTTTACAATATTTATTGGAGTAAATTTAACTTTCTTCCCACAA
5 MT492031.1     CTGATATCCCCTATTTACAGGATTAAGCTTAAATCCTTATTTTTTAAAAATTCAATTTTTTACAATATTTATTGGAGTAAATTTAACTTTCTTCCCACAA
6 MT492032.1     CTGATATCCCCTATTTACAGGATTAAGCTTAAATCCTTATTTTTTAAAAATTCAATTTTTTACAATATTTATTGGAGTAAATTTAACTTTCTTCCCACAA
7 MT492033.1     CTGATATCCCCTATTTACAGGATTAAGCTTAAATCCTTATTTTTTAAAAATTCAATTTTTTACAATATTTATTGGAGTAAATTTAACTTTCTTCCCACAA
8 MT492034.1     CTGATATCCCCTATTTACAGGATTAAGCTTAAATCCTTATTTTTTAAAAATTCAATTTTTTACAATATTTATTGGAGTAAATTTAACTTTCTTCCCACAA
9 MT492035.1     CTGATATCCCCTATTTACAGGATTAAGCTTAAATCCTTATTTTTTAAAAATTCAATTTTTTACAATATTTATTGGAGTAAATTTAACTTTCTTCCCACAA

             3001            .         .         .         .         :         .         .         .         .         1 3100
                  H  F  L  G  L  A  G  M  P  R  R  Y  S  D  Y  P  D  A  Y  I  S  W  N  I  I  S  S  L  G  S  Y  I  S
1 MN793322.1     CATTTCTTAGGGCTAGCTGGTATACCTCGACGATATTCAGATTATCCAGATGCCTATATTTCATGAAATATTATCTCATCATTAGGGTCATATATTTCTT
2 MT492036.1     CATTTCTTAGGGCTAGCTGGTATACCTCGACGATATTCAGATTATCCAGATGCCTATATTTCATGAAATATTATCTCATCATTAGGGTCATATATTTCTT
3 MT492037.1     CATTTCTTAGGGCTAGCTGGTATACCTCGACGATATTCAGATTATCCAGATGCCTATATTTCATGAAATATTATCTCATCATTAGGGTCATATATTTCTT
4 MT492030.1     CATTTCTTAGGGCTAGCTGGTATACCTCGACGATATTCAGATTATCCAGATGCCTATATTTCATGAAATATTATCTCATCATTAGGGTCATATATTTCTT
5 MT492031.1     CATTTCTTAGGGCTAGCTGGTATACCTCGACGATATTCAGATTATCCAGATGCCTATATTTCATGAAATATTATCTCATCATTAGGGTCATATATTTCTT
6 MT492032.1     CATTTCTTAGGGCTAGCTGGTATACCTCGACGATATTCAGATTATCCAGATGCCTATATTTCATGAAATATTATCTCATCATTAGGGTCATATATTTCTT
7 MT492033.1     CATTTCTTAGGGCTAGCTGGTATACCTCGACGATATTCAGATTATCCAGATGCCTATATTTCATGAAATATTATCTCATCATTAGGGTCATATATTTCTT
8 MT492034.1     CATTTCTTAGGGCTAGCTGGTATACCTCGACGATATTCAGATTATCCAGATGCCTATATTTCATGAAATATTATCTCATCATTAGGGTCATATATTTCTT
9 MT492035.1     CATTTCTTAGGGCTAGCTGGTATACCTCGACGATATTCAGATTATCCAGATGCCTATATTTCATGAAATATTATCTCATCATTAGGGTCATATATTTCTT

             3101            .         .         .         .         :         .         .         .         .         2 3200
                 L  L  A  V  M  L  I  L  I  I  I  W  E  S  M  I  N  Q  R  M  I  L  F  S  L  N  L  T  S  S  I  E  W  Y
1 MN793322.1     TACTAGCAGTGATATTAATTTTAATTATTATTTGAGAATCTATAATTAATCAACGTATAATCTTATTTTCATTAAATTTAACATCATCAATTGAATGATA
2 MT492036.1     TACTAGCAGTGATATTAATTTTAATTATTATTTGAGAATCTATAATTAATCAACGTATAATCTTATTTTCATTAAATTTAACATCATCAATTGAATGATA
3 MT492037.1     TACTAGCAGTGATATTAATTTTAATTATTATTTGAGAATCTATAATTAATCAACGTATAATCTTATTTTCATTAAATTTAACATCATCAATTGAATGATA
4 MT492030.1     TACTAGCAGTGATATTAATTTTAATTATTATTTGAGAATCTATAATTAATCAACGTATAATCTTATTTTCATTAAATTTAACATCATCAATTGAATGATA
5 MT492031.1     TACTAGCAGTGATATTAATTTTAATTATTATTTGAGAATCTATAATTAATCAACGTATAATCTTATTTTCATTAAATTTAACATCATCAATGGAATGATA
6 MT492032.1     TACTAGCAGTGATATTAATTTTAATTATTATTTGAGAATCTATAATTAATCAACGTATAATCTTATTTTCATTAAATTTAACATCATCAATTGAATGATA
7 MT492033.1     TACTAGCAGTGATATTAATTTTAATTATTATTTGAGAATCTATAATTAATCAACGTATAATCTTATTTTCATTAAATTTAACATCATCAATTGAATGATA
8 MT492034.1     TACTAGCAGTGATATTAATTTTAATTATTATTTGAGAATCTATAATTAATCAACGTATAATCTTATTTTCATTAAATTTAACATCATCAATTGAATGATA
9 MT492035.1     TACTAGCAGGGATATTAATTTTAATTATTATTTGAGAATCTATAATTAATCAACGTATAATCTTATTTTCATTAAATTTAACATCATCAATTGAATGATA
                          +
                          Ref position 2,829
```

```
                                                 | tRNA-Leu (UAA) ->
         3201      .     .     .     .     :     .     .     .     .     3 3300
           Q  N  L  P  P  A  E  H  S  Y  N  E  L  P  I  L  S  N  F  *
                                                         tctaatatggcagattatatgtaatggatttaaaccccatttat
1 MN793322.1   TCAAAATCTACCACCAGCAGAACATTCATATAACGAACTTCCAATTTTAAGAAATTTCTAAATATGGCAGATTATATGTAATGGATTTAAACCCCATTTAT
2 MT492036.1   TCAAAATCTACCACCAGCAGAACATTCATATAACGAACTTCCAATTTTAAGAAATTTCTAAATATGGCAGATTATATGTAATGGATTTAAACCCCATTTAT
3 MT492037.1   TCAAAATCTACCACCAGCAGAACATTCATATAACGAACTTCCAATTTTAAGAAATTTCTAAATATGGCAGATTATATGTAATGGATTTAAACCCCATTTAT
4 MT492030.1   TCAAAATCTACCACCAGCAGAACATTCATATAACGAACTTCCAATTTTAAGAAATTTCTAAATATGGCAGATTATATGTAATGGATTTAAACCCCATTTAT
5 MT492031.1   TCAAAACCTACCACCAGCAGAACATTCATATAACGAACTTCCAATTTTAAGAAATTTCTAAATATGGCAGATTATATGTAATGGATTTAAACCCCATTTAT
6 MT492032.1   TCAAAATCTACCACCAGCAGAACATTCATATAACGAACTTCCAATTTTAAGAAATTTCTAAATATGGCAGATTATATGTAATGGATTTAAACCCCATTTAT
7 MT492033.1   TCAAAATCTACCACCAGCAGAACATTCATATAACGAACTTCCAATTTTAAGAAATTTCTAAATATGGCAGATTATATGTAATGGATTTAAACCCCATTTAT
8 MT492034.1   TCAAATTCTACCACCAGCAGAACATTCATATAACGAACTTCCAATTTTAAGAAATTTCTAAATATGGCAGATTATATGTAATGGATTTAAACCCCATTTAT
9 MT492035.1   TCAAAATCTACCACCAGCAGAACATTCATATAACGAACTTCCAATTTTAAGAAATTTCTAAATATGGCAGATTATATGTAATGGATTTAAACCCCATTTAT

                                | COII ->
         3301      .     .     .     .     :     .     .     .     .     4 3400
               aaaggattatccttttttttagaa M  A  T  W  S  N  F  S  L  Q  N  S  A  S  P  L  M  E  Q  I  I  F  F  Y  D  H
1 MN793322.1   AAAGGATTATCCTTTTTTTTAGAAATAGCAACTTGATCTAATTTTAGTCTACAAAATAGAGCCTCTCCATTAATAGAACAAATTATTTTTTTTTATGATCA
2 MT492036.1   AAAGGATTATCCTTTTTTTTAGAAATAGCAACTTGATCTAATTTTAGTCTACAAAATAGAGCCTCTCCATTAATAGAACAAATTATTTTTTTTTATGATCA
3 MT492037.1   AAAGGATTATCCTTTTTTTTAGAAATAGCAACTTGATCTAATTTTAGTCTACAAAATAGAGCCTCTCCATTAATAGAACAAATTATTTTTTTTTATGATCA
4 MT492030.1   AAAGGATTATCCTTTTTTTTAGAAATAGCAACTTGATCTAATTTTAGTCTACAAAATAGAGCCTCTCCATTAATAGAACAAATTATTTTTTTTTATGATCA
5 MT492031.1   AAAGGATTATCCTTTTTTTTAGAAATAGCAACTTCATCTAATTTTAGTCTACAAAATAGAGCCTCTCCATTAATAGAACAAATTATTTTTTTTTATGATCA
6 MT492032.1   AAAGGATTATCCTTTTTTTTAGAAATAGCAACTTGATCTAATTTTAGTCTACAAAATAGAGCCTCTCCATTAATAGAACAAATTATTTTTTTTTATGATCA
7 MT492033.1   AAAGGATTATCCTTTTTTTTAGAAATAGCAACTTGATCTAATTTTAGTCTACAAAATAGAGCCTCTCCATTAATAGAACAAATTATTTTTTTTTATGATCA
8 MT492034.1   AAAGGATTATCCTTTTTTTTAGAAATAGCAACTTCATCTAATTTTAGTCTACAAAATAGAGCCTCTCCATTAATAGAACAAATTATTTTTTTTTATGATCA
9 MT492035.1   AAAGGATTATCCTTTTTTTTAGAAATAGCAACTTGATCTAATTTTAGTCTACAAAATAGAGCCTCTCCATTAATAGAACAAATTATTTTTTTTTATGATCA
                                                  + Sau3AI (GATC)
                                                  Ref position 3,050

         3401      .     .     .     .     :     .     .     .     .     5 3500
           T  L  I  I  L  V  M  I  T  I  L  V  G  Y  L  M  M  S  L  F  F  N  K  Y  I  N  R  F  L  L  E  G  Q
1 MN793322.1   TACATTAATTATTTTAGTTATAATTACAATTTTAGTAGGATATTTAATAATAAGATTATTTTTTAATAAATATATTAATCGATTTCTACTTGAAGGTCAA
2 MT492036.1   TACATTAATTATTTTAGTTATAATTACAATTTTAGTAGGATATTTAATAATAAGATTATTTTTTAATAAATATATTAATCGATTTCTACTTGAAGGTCAA
3 MT492037.1   TACATTAATTATTTTAGTTATAATTACAATTTTAGTAGGATATTTAATAATAAGATTATTTTTTAATAAATATATTAATCGATTTCTACTTGAAGGTCAA
4 MT492030.1   TACATTAATTATTTTAGTTATAATTACAATTTTAGTAGGATATTTAATAATAAGATTATTTTTTAATAAATATATTAATCGATTTCTACTTGAAGGTCAA
5 MT492031.1   TACATTAATTATTTTAGTTATAATTACAATTTTAGTAGGATATTTAATAATAAGATTATTTTTTAATAAATATATTAATCGATTTCTACTAGAAGGTCAA
6 MT492032.1   TACATTAATTATTTTAGTTATAATTACAATTTTAGTAGGATATTTAATAATAAGATTATTTTTTAATAAATATATTAATCGATTTCTACTTGAAGGTCAA
7 MT492033.1   TACATTAATTATTTTAGTTATAATTACAATTTTAGTAGGATATTTAATAATAAGATTATTTTTTAATAAATATATTAATCGATTTCTACTTGAAGGTCAA
8 MT492034.1   TACATTAATTATTTTAGTTATAATTACAATTTTAGTAGGATATTTAATAATAAGATTATTTTTTAATAAATATATTAATCGATTTCTACTAGAAGGTCAA
9 MT492035.1   TACATTAATTATTTTAGTTATAATTACAATTTTAGTAGGATATTTAATAATAAGATTATTTTTTAATAAATATATTAATCGATTTCTACTTGAAGGTCAA

         3501      .     .     .     .     :     .     .     .     .     6 3600
          M  I  E  L  I  W  T  I  I  P  A  I  T  L  I  F  I  A  L  P  S  L  R  L  L  Y  L  L  D  E  L  N  K
1 MN793322.1   ATAATTGAATTAATTTGAACAATTATTCCAGCTATCACATTAATCTTTATTGCTTTACCATCACTTCGATTATTATATTTACTAGATGAATTAAATAAAC
2 MT492036.1   ATAATTGAATTAATTTGAACAATTATTCCAGCTATCACATTAATCTTTATTGCTTTACCATCACTTCGATTATTATATTTACTAGATGAATTAAATAAAC
3 MT492037.1   ATAATTGAATTAATTTGAACAATTATTCCAGCTATCACATTAATCTTTATTGCTTTACCATCACTTCGATTATTATATTTACTAGATGAATTAAATAAAC
4 MT492030.1   ATAATTGAATTAATTTGAACAATTATTCCAGCTATCACATTAATCTTTATTGCTTTACCATCACTTCGATTATTATATTTACTAGATGAATTAAATAAAC
5 MT492031.1   ATAATTGAATTAATTTGAACTATTATACCAGCTATCACATTAATCTTTATTGCTTTACCATCACTTCGATTATTATATTTACTAGATGAATTAAATAAAC
6 MT492032.1   ATAATTGAATTAATTTGAACAATTATTCCAGCTATCACATTAATCTTTATTGCTTTACCATCACTTCGATTATTATATTTACTAGATGAATTAAATAAAC
7 MT492033.1   ATAATTGAATTAATTTGAACAATTATTCCAGCTATCACATTAATCTTTATTGCTTTACCATCACTTCGATTATTATATTTACTAGATGAATTAAATAAAC
8 MT492034.1   ATAATTGAATTAATTTGAACTATTATACCAGCTATCACATTAATCTTTATTGCTTTACCATCACTTCGATTATTATATTTACTAGATGAATTAAATAAAC
9 MT492035.1   ATAATTGAATTAATTTGAACAATTATTCCAGCTATCACATTAATCTTTATTGCTTTACCATCACTTCGATTATTATATTTACTAGATGAATTAAATAAAC
```

```
              3601       .         .         .         .         :         .         .         .         .         7 3700
                  P  L  I  T  L  K  S  I  G  H  Q  W  Y  W  S  Y  E  Y  S  D  F  N  N  I  E  F  D  S  Y  M  T  P  M  N
1 MN793322.1     CATTAATTACATTAAAATCAATTGGACATCAATGATATTGAAGTTATGAATATTCAGATTTTAATAACATTGAATTTGATTCTTATATAACCCCTATAAA
2 MT492036.1     CATTAATTACATTAAAATCAATTGGACATCAATGATATTGAAGTTATGAATATTCAGATTTTAATAACATTGAATTTGATTCTTATATAACCCCTATAAA
3 MT492037.1     CATTAATTACATTAAAATCAATTGGACATCAATGATATTGAAGTTATGAATATTCAGATTTTAATAATATTGAATTTGATTCTTATATAACCCCTATAAA
4 MT492030.1     CATTAATTACATTAAAATCAATTGGACATCAATGATATTGAAGTTATGAATATTCAGATTTTAATAACATTGAATTTGATTCTTATATAACCCCTATAAA
5 MT492031.1     CATTAATTACATTAAAATCAATTGGACATCAATGATATTGAAGTTATGAATATTCAGATTTTAATAACATTGAATTTGATTCTTATATAACCCCTATAAA
6 MT492032.1     CATTAATTACATTAAAATCAATTGGACATCAATGATATTGAAGTTATGAATATTCAGATTTTAATAATATTGAATTTGATTCTTATATAACCCCTATAAA
7 MT492033.1     CATTAATTACATTAAAATCAATTGGACATCAATGATATTGAAGTTATGAATATTCAGATTTTAATAATATTGAATTTGATTCTTATATAACCCCTATAAA
8 MT492034.1     CATTAATTACATTAAAATCAATTGGACATCAATGATATTGAAGTTATGAATATTCAGATTTTAATAATATTGAATTTGATTCTTATATAACCCCTATAAA
9 MT492035.1     CATTAATTACATTAAAATCAATTGGACATCAATGATATTGAAGTTATGAATATTCAGATTTTAATAATATTGAATTTGATTCTTATATAACCCCTATAAA

              3701       .         .         .         .         :         .         .         .         .         8 3800
                   E  M  N  K  N  N  F  R  L  L  E  V  D  N  R  I  I  L  P  M  N  N  Q  I  Q  I  M  V  T  A  T  D  V
1 MN793322.1     TGAAATAAATAAAAATAATTTTCGTCTTTTAGAAGTAGATAATCGAATTATTTTACCAATAAATAACCAAATTCAAATTATAGTTACCGCAACAGATGTT
2 MT492036.1     TGAAATAAATAAAAATAATTTTCGTCTTTTAGAAGTAGATAATCGAATTATTTTACCAATAAATAACCAAATTCAAATTATAGTTACCGCAACAGATGTT
3 MT492037.1     TGAAATAAATAAAAATAATTTTCGTCTTTTAGAAGTAGATAATCGAATTATTTTACCAATAAATAACCAAATTCAAATTATAGTTACCGCAACAGATGTT
4 MT492030.1     TGAAATAAATAAAAATAATTTTCGTCTTTTAGAAGTAGATAATCGAATTATTTTACCAATAAATAACCAAATTCAAATTATAGTTACCGCAACAGATGTT
5 MT492031.1     TGAAATAAATAAAAATAATTTTCGTCTTTTAGAAGTAGATAATCGAATTATTTTACCAATAAATAACCAAATTCAAATTATAGTTACCGCAACAGATGTT
6 MT492032.1     TGAAATAAATAAAAATAATTTTCGTCTTTTAGAAGTAGATAATCGAATTATTTTACCAATAAATAACCAAATTCAAATTATAGTTACCGCAACAGATGTT
7 MT492033.1     TGAAATAAATAAAAATAATTTTCGTCTTTTAGAAGTAGATAATCGAATTATTTTACCAATAAATAACCAAATTCAAATTATAGTTACCGCAACAGATGTT
8 MT492034.1     TGAAATAAATAAAAATAATTTTCGTCTTTTAGAAGTAGATAATCGAATTATTTTACCAATAAATAACCAAATTCAAATTATAGTTACCGCAACAGATGTT
9 MT492035.1     TGAAATAAATAAAAATAATTTTCGTCTTTTAGAAGTAGATAATCGAATTATTTTACCAATAAATAACCAAATTCAAATTATAGTTACCGCAACAGATGTT

              3801       .         .         .         .         :         .         .         .         .         9 3900
                  I  H  S  W  T  I  P  S  L  G  V  K  V  D  A  N  P  G  R  L  N  Q  T  N  F  F  I  N  R  P  G  I  F
1 MN793322.1     ATTCATTCATGAACTATCCCATCATTAGGAGTAAAAGTAGATGCTAATCCAGGTCGCCTAAATCAAACTAATTTTTTTATTAATCGACCTGGAATTTTTT
2 MT492036.1     ATTCATTCATGAACTATCCCATCATTAGGAGTAAAAGTAGATGCTAATCCAGGTCGCCTAAATCAAACTAATTTTTTTATTAATCGACCTGGAATTTTTT
3 MT492037.1     ATTCATTCATGAACTATCCCATCATTAGGAGTAAAAGTAGATGCTAATCCAGGTCGCCTAAATCAAACTAATTTTTTTATTAATCGACCTGGAATTTTTT
4 MT492030.1     ATTCATTCATGAACTATCCCATCATTAGGAGTAAAAGTAGATGCTAATCCAGGTCGCCTAAATCAAACTAATTTTTTTATTAATCGACCTGGAATTTTTT
5 MT492031.1     ATTCATTCATGAACTATCCCATCATTAGGAGTAAAAGTAGATGCTAATCCAGGTCGCCTAAATCAAACTAATTTTTTTATTAATCGACCTGGAATTTTTT
6 MT492032.1     ATTCATTCATGAACTATCCCATCATTAGGAGTAAAAGTAGATGCTAATCCAGGTCGCCTAAATCAAACTAATTTTTTTATTAATCGACCTGGAATTTTTT
7 MT492033.1     ATTCATTCATGAACTATCCCATCATTAGGAGTAAAAGTAGATGCTAATCCAGGTCGCCTAAATCAAACTAATTTTTTTATTAATCGACCTGGAATTTTTT
8 MT492034.1     ATTCATTCATGAACTATCCCATCATTAGGAGTAAAAGTAGATGCTAATCCAGGTCGCCTAAATCAAACTAATTTTTTTATTAATCGACCTGGAATTTTTT
9 MT492035.1     ATTCATTCATGAACTATCCCATCATTAGGAGTAAAAGTAGATGCTAATCCAGGTCGCCTAAATCAAACTAATTTTTTTATTAATCGACCTGGAATTTTTT

              3901       .         .         .         .         :         .         .         .         .         0 4000
                  F  G  Q  C  S  E  I  C  G  A  N  H  S  F  M  P  I  V  I  E  S  I  S  I  K  N  F  I  N  W  I  N  N  Y
1 MN793322.1     TCGGGCAATGTTCAGAAATTTGCGGAGCAAATCATAGATTTATACCTATTGTAATTGAAAGAATCTCAATTAAAAATTTTATTAATTGAATTAATAATTA
2 MT492036.1     TCGGGCAATGTTCAGAAATTTGCGGAGCAAATCATAGATTTATACCTATTGTAATTGAAAGAATCTCAATTAAAAATTTTATTAATTGAATTAATAATTA
3 MT492037.1     TCGGGCAATGTTCAGAAATTTGCGGAGCAAATCATAGATTTATACCTATTGTAATTGAAAGAATCTCAATTAAAAATTTTATTAATTGAATTAATAATTA
4 MT492030.1     TCGGGCAATGTTCAGAAATTTGCGGAGCAAATCATAGATTTATACCTATTGTAATTGAAAGAATCTCAATTAAAAATTTTATTAATTGAATTAATAATTA
5 MT492031.1     TCGGGCAATGTTCAGAAATTTGCGGAGCAAATCATAGATTTATACCTATTGTAATTGAAAGAATCTCAATTAAAAATTTTATTAATTGAATTAATAATTA
6 MT492032.1     TCGGGCAATGTTCAGAAATTTGCGGAGCAAATCATAGATTTATACCTATTGTAATTGAAAGAATCTCAATTAAAAATTTTATTAATTGAATTAATAATTA
7 MT492033.1     TCGGGCAATGTTCAGAAATTTGCGGAGCAAATCATAGATTTATACCTATTGTAATTGAAAGAATCTCAATTAAAAATTTTATTAATTGAATTAATAATTA
8 MT492034.1     TCGGGCAATGTTCAGAAATTTGCGGAGCAAATCATAGATTTATACCTATTGTAATTGAAAGAATCTCAATTAAAAATTTTATTAATTGAATTAATAATTA
9 MT492035.1     TCGGGCAATGTTCAGAAATTTGCGGAGCAAATCATAGATTTATACCTATTGTAATTGAAAGAATCTCAATTAAAAATTTTATTAATTCAATTAATAATTA
```

```
            | tRNA-Lys (CUU)                                                       | tRNA-Asp (GUC)
          4001         .         .         .         .         :         .         .         .         .         1 4100
              S *cattagatgactgaaagcaagtaatggtctcttaaaccatattatagtaaattagcaattacttctaatga
                                                                                        aaagaattagttaaaaaataacata
1 MN793322.1   CTCATCATTAGATGACTGAAAGCAAGTAATGGTCTCTTAAACCATATTATAGTAAATTAGCAATTACTTCTAATGAAAGAATTAGTTAAAAAATAACATA
2 MT492036.1   CTCATCATTAGATGACTGAAAGCAAGTAATGGTCTCTTAAACCATATTATAGTAAATTAGCAATTACTTCTAATGAAAGAATTAGTTAAAAAATAACATA
3 MT492037.1   CTCATCATTAGATGACTGAAAGCAAGTAATGGTCTCTTAAACCATATTATAGTAAATTAGCAATTACTTCTAATGAAAGAATTAGTTAAAAAATAACATA
4 MT492030.1   CTCATCATTAGATGACTGAAAGCAAGTAATGGTCTCTTAAACCATATTATAGTAAATTAGCAATTACTTCTAATGAAAGAATTAGTTAAAAAATAACATA
5 MT492031.1   CTCATCATTAGATGACTGAAAGCAAGTAATGGTCTCTTAAACCATATTATAGTAAATTAGCAATTACTTCTAATGAAAGAATTAGTTAAAAAATAACATA
6 MT492032.1   CTCATCATTAGATGACTGAAAGCAAGTAATGGTCTCTTAAACCATATTATAGTAAATTAGCAATTACTTCTAATGAAAGAATTAGTTAAAAAATAACATA
7 MT492033.1   CTCATCATTAGATGACTGAAAGCAAGTAATGGTCTCTTAAACCATATTATAGTAAATTAGCAATTACTTCTAATGAAAGAATTAGTTAAAAAATAACATA
8 MT492034.1   CTCATCATTAGATGACTGAAAGCAAGTAATGGTCTCTTAAACCATATTATAGTAAATTAGCAATTACTTCTAATGAAAGAATTAGTTAAAAAATAACATA
9 MT492035.1   CTCATCATTAGATGACTGAAAGCAAGTAATGGTCTCTTAAACCATATTATAGTAAATTAGCAATTACTTCTAATGAAAGAATTAGTTAAAAAATAACATA

                                                   | ATP8 ->
          4101         .         .         .         .         :         .         .         .         .         2 4200
              aatatgtcaaatttaaattattattatattaataatattctttt M  P  Q  M  M  P  I  N  W  M  L  S  L  F  F  F  I  I  V
1 MN793322.1   AATATGTCAAATTTAAATTATTATTATATTAATAATATTCTTTTATACCACAAATAATACCTATTAATTGAATATTATCTTTATTTTTCTTTATTATTGT
2 MT492036.1   AATATGTCAAATTTAAATTATT---ATATTAATAATATTCTTTTATACCACAAATAATACCTATTAATTGAATATTATCTTTATTTTTCTTTATTATTGT
3 MT492037.1   AATATGTCAAATTTAAATTATT---ATATTAATAATATTCTTTTATACCACAAATAATACCTATTAATTGAATATTATCTTTATTTTTCTTTATTATTGT
4 MT492030.1   AATATGTCAAATTTAAATTATT---ATATTAATAATATTCTTTTATACCACAAATAATACCTATTAATTGAATATTATCTTTATTTTTCTTTATTATTGT
5 MT492031.1   AATATGTCAAATTTATATTATT---ATATTAATAATATTCTTTTATACCACAAATAATACCTATTAATTGAATATTATCTTTATTTTTCTTTATTATTGT
6 MT492032.1   AATATGTCAAATTTAAATTATT---ATATTAATAATATTCTTTTATACCACAAATAATACCTATTAATTGAATATTATCTTTATTTTTCTTTATTATTGT
7 MT492033.1   AATATGTCAAATTTAAATTATT---ATAATAATAATATTCTTTTATACCACAAATAATACCTATTAATTGAATATTATCTTTATTTTTCTTTATTATTGT
8 MT492034.1   AATATGTCAAATTTATATTATT---ATATTAATAATATTCTTTTATACCACAAATAATACCTATTAATTGAATATTATCTTTATTTTTCTTTATTATTGT
9 MT492035.1   AATATGTCAAATTTAAATTATT---ATATTAATAATATTCTTTTATACCACAAATAATACCTATTAATTGAATATTATCTTTATTTTTCTTTATTATTGT

          4201         .         .         .         .         :         .         .         .         .         3 4300
                F  I  I  F  N  I  M  N  Y  Y  I  F  I  I  N  I  N  N  K  N  N  Y  F  F  N  N  K  K  N  N  Q  I  W
1 MN793322.1   TTTTATTATTTTTAATATTATAAATTATTATATTTTTATT---AATATTAATAATAAAAATAATTATTTTTTTAATAATAAAAAAAATAATCAAATTTGA
2 MT492036.1   TTTTATTATTTTTAATATTATAAATTATTATATTTTTATT---AATATTAATAATAAAAATAATTATTTTTTTAATAATAAAAAAAATAATCAAATTTGA
3 MT492037.1   TTTTATTATTTTTAATATTATAAATTATTATATTTTTATT---AATATTAATAATAAAAATAATTATTTTTTTAATAATAAAAAAAATAATCAAATTTGA
4 MT492030.1   TTTTATTATTTTTAATATTATAAATTATTATATTTTTATT---AATATTAATAATAAAAATAATTATTTTTTTAATAATAAAAAAAATAATCAAATTTGA
5 MT492031.1   TTTTATTATTTTTAATATTATAAATTATTATATTTTTATT---AATATTAATAATAAAAATAATTATTTTTTTAATAATAAAAAAAATAATCAAATTTGA
6 MT492032.1   TTTTATTATTTTTAATATTATAAATTATTATATTTTTATTATTAATATTAATAATAAAAATAATTATTTTTTTAATAATAAAAAAAATAATCAAATTTGA
7 MT492033.1   TTTTATTATTTTTAATATTATAAATTATTATATTTTTATT---AATATTAATAATAAAAATAATTATTTTTTTAATAATAAAAAAAATAATCAAATTTGA
8 MT492034.1   TTTTATTATTTTTAATATTATAAATTATTATATTTTTATT---AATATTAATAATAAAAATAATTATTTTTTTAATAATAAAAAAAATAATCAAATTTGA
9 MT492035.1   TTTTATTATTTTTAATATTATAAATTATTATATTTTTATT---AATATTAATAATAAAAATAATTATTTTTTTAATAATAAAAAAAATAATCAAATTTGA

                ATP6 ->
          4301         .         .         .         .         :         .         .         .         .         4 4400
               K  W  *
                 M  M  T  N  L  F  S  I  F  D  P  S  T  N  L  F  N  L  S  L  N  W  I  S  T  I  I  G  L  M  F  I  P
1 MN793322.1   AAATGATAACTAACTTATTTTCAATTTTTGATCCCTCAACTAATCTATTTAATCTATCTTTAAATTGAATTAGAACAATTATTGGATTAATATTTATTCC
2 MT492036.1   AAATGATAACTAACTTATTTTCAATTTTTGATCCCTCAACTAATCTATTTAATCTATCTTTAAATTGAATTAGAACAATTATTGGATTAATATTTATTCC
3 MT492037.1   AAATGATAACTAACTTATTTTCAATTTTTGATCCCTCAACTAATCTATTTAATCTATCTTTAAATTGAATTAGAACAATTATTGGATTAATATTTATTCC
4 MT492030.1   AAATGATAACTAACTTATTTTCAATTTTTGATCCCTCAACTAATCTATTTAATCTATCTTTAAATTGAATTAGAACAATTATTGGATTAATATTTATTCC
5 MT492031.1   AAATGATAACTAACTTATTTTCAATTTTTGATCCCTCAACTAATCTATTTAATCTATCTTTAAATTGAATTAGAACAATTATTGGATTAATATTTATTCC
6 MT492032.1   AAATGATAACTAACTTATTTTCAATTTTTGATCCCTCAACTAATCTATTTAATCTATCTTTAAATTGAATTAGAACAATTATTGGATTAATATTTATTCC
7 MT492033.1   AAATGATAACTAACTTATTTTCAATTTTTGATCCCTCAACTAATCTATTTAATCTATCTTTAAATTGAATTAGAACAATTATTGGATTAATATTTATTCC
8 MT492034.1   AAATGATAACTAACTTATTTTCAATTTTTGATCCCTCAACTAATCTATTTAATCTATCTTTAAATTGAATTAGAACAATTATTGGATTAATATTTATTCC
9 MT492035.1   AAATGATAACTAACTTATTTTCAATTTTTGATCCCTCAACTAATCTATTTAATCTATCTTTAAATTGAATTAGAACAATTATTGGATTAATATTTATTCC
```

```
          4401        .         .         .         .         :         .         .         .         .        5 4500
                 Y  S  F  W  L  I  P  N  R  Y  F  I  F  W  N  F  I  L  N  N  L  H  K  E  F  K  M  L  L  G  K  N  S
1 MN793322.1    ATATTCATTTTGATTAATTCCTAATCGTTATTTTATTTTTTGAAATTTTATTTTAAATAATCTTCATAAAGAATTTAAAATATTATTAGGAAAAAATTCT
2 MT492036.1    ATATTCATTTTGATTAATTCCTAATCGTTATTTTATTTTTTGAAATTTTATTTTAAATAATCTTCATAAAGAATTTAAAATATTATTAGGAAAAAATTCT
3 MT492037.1    ATATTCATTTTGATTAATTCCTAATCGTTATTTTATTTTTTGAAATTTTATTTTAAATAATCTTCATAAAGAATTTAAAATATTATTAGGAAAAAATTCT
4 MT492030.1    ATATTCATTTTGATTAATTCCTAATCGTTATTTTATTTTTTGAAATTTTATTTTAAATAATCTTCATAAAGAATTTAAAATATTATTAGGAAAAAATTCT
5 MT492031.1    ATATTCATTTTGATTAATTCCTAATCGTTATTTTATTTTTTGAAATTTTATTTTAAATAATCTTCATAAAGAATTTAAAATATTATTAGGAAAAAATTCT
6 MT492032.1    ATATTCATTTTGATTAATTCCTAATCGTTATTTTATTTTTTGAAATTTTATTTTAAATAATCTTCATAAAGAATTTAAAATATTATTAGGAAAAAATTCT
7 MT492033.1    ATATTCATTTTGATTAATTCCTAATCGTTATTTTATTTTTTGAAATTTTATTTTAAATAATCTTCATAAAGAATTTAAAATATTATTAGGAAAAAATTCT
8 MT492034.1    ATATTCATTTTGATTAATTCCTAATCGTTATTTTATTTTTTGAAATTTTATTTTAAATAATCTTCATAAAGAATTTAAAATATTATTAGGAAAAAATTCT
9 MT492035.1    ATATTCATTTTGATTAATTCCTAATCGTTATTTTATTTTTTGAAATTTTATTTTAAATAATCTTCATAAAGAATTTAAAATATTATTAGGAAAAAATTCT

          4501        .         .         .         .         :         .         .         .         .        6 4600
                N  G  S  T  F  I  F  I  S  M  F  T  F  I  L  F  N  N  F  L  G  L  F  P  Y  I  F  T  S  T  S  H  L
1 MN793322.1    AATGGATCTACTTTTATTTTTATTTCTATATTTACTTTTATTTTATTTAATAATTTTTTAGGGTTATTCCCATATATTTTTACTAGAACTAGACATCTTA
2 MT492036.1    AATGGATCTACTTTTATTTTTATTTCTATATTTACTTTTATTTTATTTAATAATTTTTTAGGGTTATTCCCATATATTTTTACTAGAACTAGACATCTTA
3 MT492037.1    AATGGATCTACTTTTATTTTTATTTCTATATTTACTTTTATTTTATTTAATAATTTTTTAGGGTTATTCCCATATATTTTTACTAGAACTAGACATCTTA
4 MT492030.1    AATGGATCTACTTTTATTTTTATTTCTATATTTACTTTTATTTTATTTAATAATTTTTTAGGGTTATTCCCATATATTTTTACTAGAACTAGACATCTTA
5 MT492031.1    AATGGATCTACTTTTATTTTTATTTCTATATTTACTTTTATTTTATTTAATAATTTTTTAGGGTTATTCCCATATATTTTTACTAGAACTAGACATCTTA
6 MT492032.1    AATGGATCTACTTTTATTTTTATTTCTATATTTACTTTTATTTTATTTAATAATTTTTTAGGGTTATTCCCATATATTTTTACTAGAACTAGACATCTTA
7 MT492033.1    AATGGATCTACTTTTATTTTTATTTCTATATTTACTTTTATTTTATTTAATAATTTTTTAGGGTTATTCCCATATATTTTTACTAGAACTAGACATCTTA
8 MT492034.1    AATGGATCTACTTTTATTTTTATTTCTATATTTACTTTTATTTTATTTAATAATTTTTTAGGGTTATTCCCATATATTTTTACTAGAACTAGACATCTTA
9 MT492035.1    AATGGATCTACTTTTATTTTTATTTCTATATTTACTTTTATTTTATTTAATAATTTTTTAGGGTTATTCCCATATATTTTTACTAGAACTAGACATCTTA

          4601        .         .         .         .         :         .         .         .         .        7 4700
                T  M  S  L  S  I  S  L  P  L  W  L  S  F  M  F  Y  G  W  L  N  N  T  Q  H  M  F  I  H  M  I  P  Q  G
1 MN793322.1    CTATGTCATTATCTATTTCATTACCATTATGATTAAGATTTATATTTTACGGATGATTAAATAACACTCAACACATATTTATTCATATAATTCCTCAAGG
2 MT492036.1    CTATGTCATTATCTATTTCATTACCATTATGATTAAGATTTATATTTTACGGATGATTAAATAACACTCAACACATATTTATTCATATAATTCCTCAAGG
3 MT492037.1    CTATGTCATTATCTATTTCATTACCATTATGATTAAGATTTATATTTTACGGATGATTAAATAACACTCAACACATATTTATTCATATAATTCCTCAAGG
4 MT492030.1    CTATGTCATTATCTATTTCATTACCATTATGATTAAGATTTATATTTTACGGATGATTAAATAACACTCAACACATATTTATTCATATAATTCCTCAAGG
5 MT492031.1    CTATGTCATTATCTATTTCATTACCATTATGATTAAGATTTATATTTTACGGATGATTAAATAACACTCAACACATATTTATTCATATAATTCCTCAAGG
6 MT492032.1    CTATGTCATTATCTATTTCATTACCATTATGATTAAGATTTATATTTTACGGATGATTAAATAACACTCAACACATATTTATTCATATAATTCCTCAAGG
7 MT492033.1    CTATGTCATTATCTATTTCATTACCATTATGATTAAGATTTATATTTTACGGATGATTAAATAACACTCAACACATATTTATTCATATAATTCCTCAAGG
8 MT492034.1    CTATGTCATTATCTATTTCATTACCATTATGATTAAGATTTATATTTTACGGATGATTAAATAACACTCAACACATATTTATTCATATAATTCCTCAAGG
9 MT492035.1    CTATGTCATTATCTATTTCATTACCATTATGATTAAGATTTATATTTTACGGATGATTAAATAACACTCAACACATATTTATTCATATAATTCCTCAAGG

          4701        .         .         .         .         :         .         .         .         .        8 4800
                 T  P  T  I  L  M  P  F  M  V  L  I  E  T  I  S  N  I  I  R  P  G  T  L  A  V  R  L  T  A  N  M  I
1 MN793322.1    AACACCAACAATTTTAATACCATTTATAGTATTAATTGAAACAATTAGAAATATTATTCGACCTGGAACTTTAGCAGTACGACTTACTGCTAATATAATT
2 MT492036.1    AACACCAACAATTTTAATACCATTTATAGTATTAATTGAAACAATTAGAAATATTATTCGACCTGGAACTTTAGCAGTACGACTTACTGCTAATATAATT
3 MT492037.1    AACACCAACAATTTTAATACCATTTATAGTATTAATTGAAACAATTAGAAATATTATTCGACCTGGAACTTTAGCAGTACGACTTACTGCTAATATAATT
4 MT492030.1    AACACCAACAATTTTAATACCATTTATAGTATTAATTGAAACAATTAGAAATATTATTCGACCTGGAACTTTAGCAGTACGACTTACTGCTAATATAATT
                 T  P  T  I  L  M  P  F  M  V  L  I  E  T  I  S  K  S  T  P  T  G  T  L  A  V  R  L  T  A  N  M  I
5 MT492031.1    AACACCAACAATTTTAATACCATTTATAGTATTAATTGAAACAATTAGAAAAAGTACTCCAACTGGAACTTTAGCAGTACGACTTACTGCTAATATAATT
6 MT492032.1    AACACCAACAATTTTAATACCATTTATAGTATTAATTGAAACAATTAGAAATATTATTCGACCTGGAACTTTAGCAGTACGACTTACTGCTAATATAATT
7 MT492033.1    AACACCAACAATTTTAATACCATTTATAGTATTAATTGAAACAATTAGAAATATTATTCGACCTGGAACTTTAGCAGTACGACTTACTGCTAATATAATT
8 MT492034.1    AACACCAACAATTTTAATACCATTTATAGTATTAATTGAAACAATTAGAAATATTATTCGACCTGGAACTTTAGCAGTACGACTTACTGCTAATATAATT
9 MT492035.1    AACACCAACAATTTTAATACCATTTATAGTATTAATTGAAACAATTAGAAATATTATTCGACCTGGAACTTTAGCAGTACGACTTACTGCTAATATAATT
```

```
             4801      .         .         .         .         :         .         .         .         .       9 4900
                  A  G  H  L  L  M  T  L  L  S  G  T  G  N  S  L  S  T  Y  M  I  I  L  L  V  I  I  Q  I  L  L  L  I
1 MN793322.1     GCAGGACATTTACTAATAACTTTACTTAGAGGTACTGGAAATAGATTAAGAACGTATATAATTATTTTATTAGTAATTATTCAAATTTTATTGTTAATTT
2 MT492036.1     GCAGGACATTTACTAATAACTTTACTTAGAGGTACTGGAAATAGATTAAGAACGTATATAATTATTTTATTAGTAATTATTCAAATTTTATTGTTAATTT
3 MT492037.1     GCAGGACATTTACTAATAACTTTACTTAGAGGTACTGGAAATAGATTAAGAACGTATATAATTATTTTATTAGTAATTATTCAAATTTTATTGTTAATTT
4 MT492030.1     GCAGGACATTTACTAATAACTTTACTTAGAGGTACTGGAAATAGATTAAGAACGTATATAATTATTTTATTAGTAATTATTCAAATTTTATTGTTAATTT
5 MT492031.1     GCAGGACATTTACTAATAACTTTACTTAGAGGTACTGGAAATAGATTAAGAACGTATATAATTATTTTATTAGTAATTATTCAAATTTTATTGTTAATTC
6 MT492032.1     GCAGGACATTTACTAATAACTTTACTTAGAGGTACTGGAAATAGATTAAGAACGTATATAATTATTTTATTAGTAATTATTCAAATTTTATTGTTAATTT
7 MT492033.1     GCAGGACATTTACTAATAACTTTACTTAGAGGTACTGGAAATAGATTAAGAACGTATATAATTATTTTATTAGTAATTATTCAAATTTTATTGTTAATTT
8 MT492034.1     GCAGGACATTTACTAATAACTTTACTTAGAGGTACTGGAAATAGATTAAGAACGTATATAATTATTTTATTAGTAATTATTCAAATTTTATTGTTAATTT
9 MT492035.1     GCAGGACATTTACTAATAACTTTACTTAGAGGTACTGGAAATAGATTAAGAACGTATATAATTATTTTATTAGTAATTATTCAAATTTTATTGTTAATTT

                                                                                            |COIII ->
             4901      .         .         .         .         :         .         .         .         .       0 5000
                 L  E  S  A  V  A  V  I  Q  S  Y  V  I  A  I  L  S  T  L  Y  S  S  E  V  N  *  M  K  N  N  F  Y  S  H
1 MN793322.1     TAGAATCTGCAGTGGCAGTAATTCAATCTTATGTAATTGCCATTTTAAGAACTTTATATTCTAGAGAAGTAAATTAATGAAAAATAATTTTTACAGACAT
2 MT492036.1     TAGAATCTGCAGTGGCAGTAATTCAATCTTATGTAATTGCCATTTTAAGAACTTTATATTCTAGAGAAGTAAATTAATGAAAAATAATTTTTACAGACAT
3 MT492037.1     TAGAATCTGCAGTGGCAGTAATTCAATCTTATGTAATTGCCATTTTAAGAACTTTATATTCTAGAGAAGTAAATTAATGAAAAATAATTTTTACAGACAT
4 MT492030.1     TAGAATCTGCAGTGGCAGTAATTCAATCTTATGTAATTGCCATTTTAAGAACTTTATATTCTAGAGAAGTAAATTAATGAAAAATAATTTTTACAGACAT
5 MT492031.1     TAGAATCTGCAGTGGCAGTAATTCAATCTTATGTAATTGCCATTTTAAGAACTTTATATTCTAGAGAAGTAAATTAATGAAAAATAATTTTTACAGACAT
6 MT492032.1     TAGAATCTGCAGTGGCAGTAATTCAATCTTATGTAATTGCCATTTTAAGAACTTTATATTCTAGAGAAGTAAATTAATGAAAAATAATTTTTACAGACAT
7 MT492033.1     TAGAATCTGCAGTGGCAGTAATTCAATCTTATGTAATTGCCATTTTAAGAACTTTATATTCTAGAGAAGTAAATTAATGAAAAATAATTTTTACAGACAT
8 MT492034.1     TAGAATCTGCAGTGGCAGTAATTCAATCTTATGTAATTGCCATTTTAAGAACTTTATATTCTAGAGAAGTAAATTAATGAAAAATAATTTTTACAGACAT
9 MT492035.1     TAGAATCTGCAGTGGCAGTAATTCAATCTTATGTAATTGCCATTTTAAGAACTTTATATTCTAGAGAAGTAAATTAATGAAAAATAATTTTTACAGACAT

             5001      .         .         .         .         :         .         .         .         .       1 5100
                  P  Y  H  L  V  D  Y  S  P  W  P  I  T  G  A  I  G  V  L  T  L  V  T  G  M  V  K  W  F  H  N  F  N
1 MN793322.1     CCATATCATTTAGTTGATTACAGACCTTGACCTATTACCGGAGCTATTGGAGTATTAACTTTAGTTACAGGAATAGTTAAATGATTTCACAATTTTAACA
2 MT492036.1     CCATATCATTTAGTTGATTACAGACCTTGACCTATTACCGGAGCTATTGGAGTATTAACTTTAGTTACAGGAATAGTTAAATGATTTCACAATTTTAACA
3 MT492037.1     CCATATCATTTAGTTGATTACAGACCTTGACCTATTACCGGAGCTATTGGAGTATTAACTTTAGTTACAGGAATAGTTAAATGATTTCACAATTTTAACA
4 MT492030.1     CCATATCATTTAGTTGATTACAGACCTTGACCTATTACCGGAGCTATTGGAGTATTAACTTTAGTTACAGGAATAGTTAAATGATTTCACAATTTTAACA
5 MT492031.1     CCATATCATTTAGTTGATTACAGACCTTGACCTATTACCGGAGCTATTGGAGTATTAACTTTAGTTACAGGAATAGTTGAATGATTTCACAATTTTAACA
6 MT492032.1     CCATATCATTTAGTTGATTACAGACCTTGACCTATTACCGGAGCTATTGGAGTATTAACTTTAGTTACAGGAATAGTTAAATGATTTCACAATTTTAACA
7 MT492033.1     CCATATCATTTAGTTGATTACAGACCTTGACCTATTACCGGAGCTATTGGAGTATTAACTTTAGTTACAGGAATAGTTAAATGATTTCACAATTTTAACA
8 MT492034.1     CCATATCATTTAGTTGATTACAGACCTTGACCTATTACCGGAGCTATTGGAGTATTAACTTTAGTTACAGGAATAGTTAAATGATTTCACAATTTTAACA
9 MT492035.1     CCATATCATTTAGTTGATTACAGACCTTGACCTATTACCGGAGCTATTGGAGTATTAACTTTAGTTACAGGAATAGTTAAATGATTTCACAATTTTAACA

             5101      .         .         .         .         :         .         .         .         .       2 5200
                 M  N  L  L  M  L  G  Y  L  I  I  I  L  T  M  Y  Q  W  W  R  D  V  S  R  E  G  T  F  Q  G  K  H  T  I
1 MN793322.1     TAAATTTATTAATATTAGGCTATTTAATTATTATTTTAACAATATATCAATGATGACGAGATGTATCACGAGAAGGAACATTTCAAGGTAAACATACCAT
2 MT492036.1     TAAATTTATTAATATTAGGCTATTTAATTATTATTTTAACAATATATCAATGATGACGAGATGTATCACGAGAAGGAACATTTCAAGGTAAACATACCAT
3 MT492037.1     TAAATTTATTAATATTAGGCTATTTAATTATTATTTTAACAATATATCAATGATGACGAGATGTATCACGAGAAGGAACATTTCAAGGTAAACATACCAT
4 MT492030.1     TAAATTTATTAATATTAGGCTATTTAATTATTATTTTAACAATATATCAATGATGACGAGATGTATCACGAGAAGGAACATTTCAAGGTAAACATACCAT
5 MT492031.1     TAAATTTATTAATATTAGGCTATTTAATTATTATTTTAACAATATATCAATGATGACGAGATGTATCACGAGAAGGAACATTTCAAGGTAAACATACCAT
6 MT492032.1     TAAATTTATTAATATTAGGCTATTTAATTATTATTTTAACAATATATCAATGATGACGAGATGTATCACGAGAAGGAACATTTCAAGGTAAACATACCAT
7 MT492033.1     TAAATTTATTAATATTAGGCTATTTAATTATTATTTTAACAATATATCAATGATGACGAGATGTATCACGAGAAGGAACATTTCAAGGTAAACATACCAT
8 MT492034.1     TAAATTTATTAATATTAGGCTATTTAATTATTATTTTAACAATATATCAATGATGACGAGATGTATCACGAGAAGGAACATTTCAAGGTAAACATACCAT
9 MT492035.1     TAAATTTATTAATATTAGGCTATTTAATTATTATTTTAACAATATATCAATGATGACGAGATGTATCACGAGAAGGAACATTTCAAGGTAAACATACCAT
```

```
           5201         .         .         .         .         :         .         .         .         .        3 5300
                 L  V  T  K  G  L  R  W  G  M  I  L  F  I  I  S  E  I  F  F  F  I  S  F  F  W  A  F  F  H  S  S  L
1 MN793322.1    TTTAGTAACTAAAGGATTACGATGAGGAATAATCTTATTTATTATTTCTGAAATTTTTTTTTCATTTCATTTTTTTGAGCATTTTTTCATAGTAGATTA
2 MT492036.1    TTTAGTAACTAAAGGATTACGATGAGGAATAATCTTATTTATTATTTCTGAAATTTTTTTTTCATTTCATTTTTTTGAGCATTTTTTCATAGTAGATTA
3 MT492037.1    TTTAGTAACTAAAGGATTACGATGAGGAATAATCTTATTTATTATTTCTGAAATTTTTTTTTCATTTCATTTTTTTGAGCATTTTTTCATAGTAGATTA
4 MT492030.1    TTTAGTAACTAAAGGATTACGATGAGGAATAATCTTATTTATTATTTCTGAAATTTTTTTTTCATTTCATTTTTTTGAGCATTTTTTCATAGTAGATTA
5 MT492031.1    TTTAGTAACTAAAGGATTACGATGAGGAATAATCTTATTTATTATTTCTGAAATTTTTTTTTCATTTCATTTTTTTGAGCATTTTTTCATAGTAGATTA
6 MT492032.1    TTTAGTAACTAAAGGATTACGATGAGGAATAATCTTATTTATTATTTCTGAAATTTTTTTTTCATTTCATTTTTTTGAGCATTTTTTCATAGTAGATTA
7 MT492033.1    TTTAGTAACTAAAGGATTACGATGAGGAATAATCTTATTTATTATTTCTGAAATTTTTTTTTCATTTCATTTTTTTGAGCATTTTTTCATAGTAGATTA
8 MT492034.1    TTTAGTAACTAAAGGATTACGATGAGGAATAATCTTATTTATTATTTCTGAAATTTTTTTTTCATTTCATTTTTTTGAGCATTTTTTCATAGTAGATTA
9 MT492035.1    TTTAGTAACTAAAGGATTACGATGAGGAATAATCTTATTTATTATTTCTGAAATTTTTTTTTCATTTCATTTTTTTGAGCATTTTTTCATAGTAGATTA

           5301         .         .         .         .         :         .         .         .         .        4 5400
                S  P  N  I  E  I  G  A  T  W  P  P  T  N  I  V  P  F  N  P  F  Q  I  P  L  L  N  T  I  I  L  I  T
1 MN793322.1    TCACCTAATATTGAAATTGGAGCAACTTGACCTCCTACAAATATTGTACCATTTAATCCATTTCAAATTCCATTACTAAATACAATCATTTTAATTACAT
2 MT492036.1    TCACCTAATATTGAAATTGGAGCAACTTGACCTCCTACAAATATTGTACCATTTAATCCATTTCAAATTCCATTACTAAATACAATCATTTTAATTACAT
3 MT492037.1    TCACCTAATATTGAAATTGGAGCAACTTGACCTCCTACAAATATTGTACCATTTAATCCATTTCAAATTCCATTACTAAATACAATCATTTTAATTACAT
4 MT492030.1    TCACCTAATATTGAAATTGGAGCAACTTGACCTCCTACAAATATTGTACCATTTAATCCATTTCAAATTCCATTACTAAATACAATCATTTTAATTACAT
5 MT492031.1    TCACCTAATATTGAAATTGGAGCAACTTGACCTCCTACAAATATTGTACCATTTAATCCATTTCAAATTCCATTACTAAATACAATCATTTTAATTACAT
6 MT492032.1    TCACCTAATATTGAAATTGGAGCAACTTGACCTCCTACAAATATTGTACCATTTAATCCATTTCAAATTCCATTACTAAATACAATCATTTTAATTACAT
7 MT492033.1    TCACCTAATATTGAAATTGGAGCAACTTGACCTCCTACAAATATTGTACCATTTAATCCATTTCAAATTCCATTACTAAATACAATCATTTTAATTACAT
8 MT492034.1    TCACCTAATATTGAAATTGGAGCAACTTGACCTCCTACAAATATTGTACCATTTAATCCATTTCAAATTCCATTACTAAATACAATCATTTTAATTACAT
9 MT492035.1    TCACCTAATATTGAAATTGGAGCAACTTGACCTCCTACAAATATTGTACCATTTAATCCATTTCAAATTCCATTACTAAATACAATCATTTTAATTACAT

           5401         .         .         .         .         :         .         .         .         .        5 5500
                S  G  V  T  V  T  W  A  H  H  A  I  M  E  N  N  H  S  Q  M  T  Q  G  L  F  L  T  I  I  L  G  I  Y  F
1 MN793322.1    CAGGAGTAACAGTAACATGGGCTCACCATGCAATTATAGAAAATAACCACTCTCAAATAACTCAAGGATTATTTTTAACTATTATTTTAGGAATTTATTT
2 MT492036.1    CAGGAGTAACAGTAACATGGGCTCACCATGCAATTATAGAAAATAACCACTCTCAAATAACTCAAGGATTATTTTTAACTATTATTTTAGGAATTTATTT
3 MT492037.1    CAGGAGTAACAGTAACATGGGCTCACCATGCAATTATAGAAAATAACCACTCTCAAATAACTCAAGGATTATTTTTAACTATTATTTTAGGAATTTATTT
4 MT492030.1    CAGGAGTAACAGTAACATGGGCTCACCATGCAATTATAGAAAATAACCACTCTCAAATAACTCAAGGATTATTTTTAACTATTATTTTAGGAATTTATTT
5 MT492031.1    CAGGAGTAACAGTAACATGGGCTCACCATGCAATTATAGAAAATAACCACTCTCAAATAACTCAAGGATTATTTTTAACTATTATTTTAGGAATTTATTT
6 MT492032.1    CAGGAGTAACAGTAACATGGGCTCATCATGCAATTATAGAAAATAACCACTCTCAAATAACTCAAGGATTATTTTTAACTATTATTTTAGGAATTTATTT
7 MT492033.1    CAGGAGTAACAGTAACATGGGCTCACCATGCAATTATAGAAAATAACCACTCTCAAATAACTCAAGGATTATTTTTAACTATTATTTTAGGAATTTATTT
8 MT492034.1    CAGGAGTAACAGTAACATGGGCTCATCATGCAATTATAGAAAATAACCACTCTCAAATAACTCAAGGATTATTTTTAACTATTATTTTAGGAATTTATTT
9 MT492035.1    CAGGAGTAACAGTAACATGGGCTCACCATGCAATTATAGAAAATAACCACTCTCAAATAACTCAAGGATTATTTTTAACTATTATTTTAGGAATTTATTT

           5501         .         .         .         .         :         .         .         .         .        6 5600
                  T  F  L  Q  A  Y  E  Y  L  E  A  P  F  T  I  A  D  S  I  Y  G  S  T  F  F  M  A  T  G  F  H  G  L
1 MN793322.1    CACTTTTCTTCAAGCATATGAATATTTAGAAGCACCTTTTACTATCGCAGATAGAATTTATGGATCTACATTTTTTATAGCAACAGGATTCCATGGATTA
2 MT492036.1    CACTTTTCTTCAAGCATATGAATATTTAGAAGCACCTTTTACTATCGCAGATAGAATTTATGGATCTACATTTTTTATAGCAACAGGATTCCATGGATTA
3 MT492037.1    CACTTTTCTTCAAGCATATGAATATTTAGAAGCACCTTTTACTATCGCAGATAGAATTTATGGATCTACATTTTTTATAGCAACAGGATTCCATGGATTA
4 MT492030.1    CACTTTTCTTCAAGCATATGAATATTTAGAAGCACCTTTTACTATCGCAGATAGAATTTATGGATCTACATTTTTTATAGCAACAGGATTCCATGGATTA
5 MT492031.1    CACTTTTCTTCAAGCATATGAATATTTAGAAGCACCTTTTACTATCGCAGATAGAATTTATGGATCTACATTTTTTATAGCAACAGGATTCCATGGATTA
6 MT492032.1    CACTTTTCTTCAAGCATATGAATATTTAGAAGCACCTTTTACTATCGCAGATAGAATTTATGGATCTACATTTTTTATAGCAACAGGATTCCATGGATTA
7 MT492033.1    CACTTTTCTTCAAGCATATGAATATTTAGAAGCACCTTTTACTATCGCAGATAGAATTTATGGATCTACATTTTTTATAGCAACAGGATTCCATGGATTA
8 MT492034.1    CACTTTTCTTCAAGCATATGAATATTTAGAAGCACCTTTTACTATCGCAGATAGAATTTATGGATCTACATTTTTTATAGCAACAGGATTCCATGGATTA
9 MT492035.1    CACTTTTCTTCAAGCATATGAATATTTAGAAGCACCTTTTACTATCGCAGATAGAATTTATGGATCTACATTTTTTATAGCAACAGGATTCCATGGATTA
```

```
              5601      .         .         .         .         :         .         .         .         .      7 5700
                    H  V  M  I  G  T  M  F  L  L  I  C  L  I  R  H  I  N  N  Q  F  S  N  N  H  H  F  G  F  E  A  A  A
1 MN793322.1     CATGTAATAATTGGAACAATATTTTTATTAATTTGTTTAATTCGTCATATTAATAATCAATTTTCTAATAACCATCATTTTGGATTTGAAGCAGCAGCTT
2 MT492036.1     CATGTAATAATTGGAACAATATTTTTATTAATTTGTTTAATTCGTCATATTAATAATCAATTTTCTAATAACCATCATTTTGGATTTGAAGCAGCAGCTT
3 MT492037.1     CATGTAATAATTGGAACAATATTTTTATTAATTTGTTTAATTCGTCATATTAATAATCAATTTTCTAATAACCATCATTTTGGATTTGAAGCAGCAGCTT
4 MT492030.1     CATGTAATAATTGGAACAATATTTTTATTAATTTGTTTAATTCGTCATATTAATAATCAATTTTCTAATAACCATCATTTTGGATTTGAAGCAGCAGCTT
5 MT492031.1     CATGTAATAATTGGAACAATATTTTTATTAATTTGTTTAATTCGTCATATTAATAATCAATTTTCTAATAACCATCATTTTGGATTTGAAGCAGCAGCTT
6 MT492032.1     CATGTAATAATTGGAACAATATTTTTATTAATTTGTTTAATTCGTCATATTAATAATCAATTTTCTAATAACCATCATTTTGGATTTGAAGCAGCAGCTT
7 MT492033.1     CATGTAATAATTGGAACAATATTTTTATTAATTTGTTTAATTCGTCATATTAATAATCAATTTTCTAATAACCATCATTTTGGATTTGAAGCAGCAGCTT
8 MT492034.1     CATGTAATAATTGGAACAATATTTTTATTAATTTGTTTAATTCGTCATATTAATAATCAATTTTCTAATAACCATCATTTTGGATTTGAAGCAGCAGCTT
9 MT492035.1     CATGTAATAATTGGAACAATATTTTTATTAATTTGTTTAATTCGTCATATTAATAATCAATTTTCTAATAACCATCATTTTGGATTTGAAGCAGCAGCTT

                                                                                        | tRNA-Glu (UCC) ->
              5701      .         .         .         .         :         .         .         .         .      8 5800
                 W  Y  W  H  F  V  D  V  V  W  L  F  L  Y  I  S  I  Y  W  W  G  N  *   atttatataatatatttagtatatttgact
1 MN793322.1     GATATTGACATTTTGTAGATGTAGTTTGATTATTCCTTTATATTTCTATTTATTGATGAGGAAATTAATTATTTATATAATATATTTAGTATATTTGACT
2 MT492036.1     GATATTGACATTTTGTAGATGTAGTTTGATTATTCCTTTATATTTCTATTTATTGATGAGGAAATTAATTATTTATATAATATATTTAGTATATTTGACT
3 MT492037.1     GATATTGACATTTTGTAGATGTAGTTTGATTATTCCTTTATATTTCTATTTATTGATGAGGAAATTAATTATTTATATAATATATTTAGTATATTTGACT
4 MT492030.1     GATATTGACATTTTGTAGATGTAGTTTGATTATTCCTTTATATTTCTATTTATTGATGAGGAAATTAATTATTTATATAATATATTTAGTATATTTGACT
5 MT492031.1     GATATTGACATTTTGTAGATGTAGTTTGATTATTCCTTTATATTTCTATTTATTGATGAGGAAATTAATTATTTATATAATATATTTAGTATATTTGACT
6 MT492032.1     GATATTGACATTTTGTAGATGTAGTTTGATTATTCCTTTATATTTCTATTTATTGATGAGGAAATTAATTATTTATATAATATATTTAGTATATTTGACT
7 MT492033.1     GATATTGACATTTTGTAGATGTAGTTTGATTATTCCTTTATATTTCTATTTATTGATGAGGAAATTAATTATTTATATAATATATTTAGTATATTTGACT
8 MT492034.1     GATATTGACATTTTGTAGATGTAGTTTGATTATTCCTTTATATTTCTATTTATTGATGAGGAAATTAATTATTTATATAATATATTTAGTATATTTGACT
9 MT492035.1     GATATTGACATTTTGTAGATGTAGTTTGATTATTCCTTTATATTTCTATTTATTGATGAGGAAATTAATTATTTATATAATATATTTAGTATATTTGACT

                                                      | ND3 ->
              5801      .         .         .         .         :         .         .         .         .      9 5900
                 tccaatcaaaaagtttaatattttattaatataaata M  I  L  I  S  Y  I  S  I  I  I  M  M  I  S  N  I  M  M  F  L
1 MN793322.1     TCCAATCAAAAAGTTTAATATTTTATTAATATAAATAATTATTTTAATTAGTTATATTTCTATTATTATTATAATAATCTCAAATATTATAATATTTTTA
2 MT492036.1     TCCAATCAAAAAGTTTAATATTTTATTAATATAAATAATTATTTTAATTAGTTATATTTCTATTATTATTATAATAATCTCAAATATTATAATATTTTTA
3 MT492037.1     TCCAATCAAAAAGTTTAATATTTTATTAATATAAATAATTATTTTAATTAGTTATATTTCTATTATTATTATAATAATCTCAAATATTATAATATTTTTA
4 MT492030.1     TCCAATCAAAAAGTTTAATATTTTATTAATATAAATAATTATTTTAATTAGTTATATTTCTATTATTATTATAATAATCTCAAATATTATAATATTTTTA
5 MT492031.1     TCCAATCAAAAAGTTTAATATTTTATTAATATAAATAATTATTTTAATTAGTTATATTTCTATTATTATTATAATAATCTCAAATATTATAATATTTTTA
6 MT492032.1     TCCAATCAAAAAGTTTAATATTTTATTAATATAAATAATTATTTTAATTAGTTATATTTCTATTATTATTATAATAATCTCAAATATTATAATATTTTTA
7 MT492033.1     TCCAATCAAAAAGTTTAATATTTTATTAATATAAATAATTATTTTAATTAGTTATATTTCTATTATTATTATAATAATCTCAAATATTATAATATTTTTA
8 MT492034.1     TCCAATCAAAAAGTTTAATATTTTATTAATATAAATAATTATTTTAATTAGTTATATTTCTATTATTATTATAATAATCTCAAATATTATAATATTTTTA
9 MT492035.1     TCCAATCAAAAAGTTTAATATTTTATTAATATAAATAATTATTTTAATTAGTTATATTTCTATTATTATTATAATAATCTCAAATATTATAATATTTTTA

              5901      .         .         .         .         :         .         .         .         .      0 6000
                    S  I  I  L  S  K  K  S  F  S  D  R  E  K  N  S  P  F  E  C  G  F  D  P  K  S  S  A  R  I  P  F  S
1 MN793322.1     TCCATTATTTTTATCAAAAAAATCATTTTCTGATCGAGAAAAAAAATTCCCCATTTGAATGTGGATTTGATCCAAAATCATCAGCTCGAATCCCATTCTCCT
2 MT492036.1     TCCATTATTTTTATCAAAAAAATCATTTTCTGATCGAGAAAAAAAATTCCCCATTTGAATGTGGATTTGATCCAAAATCATCAGCTCGAATCCCATTCTCCT
3 MT492037.1     TCCATTATTTTTATCAAAAAAATCATTTTCTGATCGAGAAAAAAAATTCCCCATTTGAATGTGGATTTGATCCAAAATCATCAGCTCGAATCCCATTCTCCT
4 MT492030.1     TCCATTATTTTTATCAAAAAAATCATTTTCTGATCGAGAAAAAAAATTCCCCATTTGAATGTGGATTTGATCCAAAATCATCAGCTCGAATCCCATTCTCCT
5 MT492031.1     TCCATTATTTTTATCAAAAAAATCATTTTCTGATCGAGAAAAAAAATTCCCCATTTGAATGTGGATTTGATCCAAAATCATCAGCTCGAATCCCATTCTCCT
6 MT492032.1     TCCATTATTTTTATCAAAAAAATCATTTTCTGATCGAGAAAAAAAATTCCCCATTTGAATGTGGATTTGATCCAAAATCATCAGCTCGAATCCCATTCTCCT
7 MT492033.1     TCCATTATTTTTATCAAAAAAATCATTTTCTGATCGAGAAAAAAAATTCCCCATTTGAATGTGGATTTGATCCAAAATCATCAGCTCGAATCCCATTCTCCT
8 MT492034.1     TCCATTATTTTTATCAAAAAAATCATTTTCTGATCGAGAAAAAAAATTCCCCATTTGAATGTGGATTTGATCCAAAATCATCAGCTCGAATCCCATTCTCCT
9 MT492035.1     TCCATTATTTTTATCAAAAAAATCATTTTCTGATCGAGAAAAAAAATTCCCCATTTGAATGTGGATTTGATCCAAAATCATCAGCTCGAATCCCATTCTCCT
```

```
              6001      .         .         .         .         :         .         .         .         .         1 6100
                 L  H  F  F  L  I  T  M  I  F  L  I  F  D  V  E  I  A  L  I  F  P  M  I  N  L  F  K  M  T  N  F  I  I
1 MN793322.1     TACATTTCTTCTTAATTACAATAATTTTTTTAATTTTTGATGTTGAAATTGCATTAATTTTTCCTATAATTAATTTATTTAAAATAACAAATTTTATTAT
2 MT492036.1     TACATTTCTTCTTAATTACAATAATTTTTTTAATTTTTGATGTTGAAATTGCATTAATTTTTCCTATAATTAATTTATTTAAAATAACAAATTTTATTAT
3 MT492037.1     TACATTTCTTCTTAATTACAATAATTTTTTTAATTTTTGATGTTGAAATTGCATTAATTTTTCCTATAATTAATTTATTTAAAATAACAAATTTTATTAT
4 MT492030.1     TACATTTCTTCTTAATTACAATAATTTTTTTAATTTTTGATGTTGAAATTGCATTAATTTTTCCTATAATTAATTTATTTAAAATAACAAATTTTATTAT
5 MT492031.1     TACATTTCTTCTTAATTACAATAATTTTTTTAATTTTTGATGTTGAAATTGCATTAATTTTTCCTATAATTAATTTATTTAAAATAACAAATTTTATTAT
6 MT492032.1     TACATTTCTTCTTAATTACAATAATTTTTTTAATTTTTGATGTTGAAATTGCATTAATTTTTCCTATAATTAATTTATTTAAAATAACAAATTTTATTAT
7 MT492033.1     TACATTTCTTCTTAATTACAATAATTTTTTTAATTTTTGATGTTGAAATTGCATTAATTTTTCCTATAATTAATTTATTTAAAATAACAAATTTTATTAT
8 MT492034.1     TACATTTCTTCTTAATTACAATAATTTTTTTAATTTTTGATGTTGAAATTGCATTAATTTTTCCTATAATTAATTTATTTAAAATAACAAATTTTATTAT
9 MT492035.1     TACATTTCTTCTTAATTACAATAATTTTTTTAATTTTTGATGTTGAAATTGCATTAATTTTTCCTATAATTAATTTATTTAAAATAACAAATTTTATTAT

              6101      .         .         .         .         :         .         .         .         .         2 6200
                  W  T  K  I  S  F  F  F  I  I  I  L  L  I  G  L  F  H  E  W  N  Q  N  M  L  N  W  T  K  *
1 MN793322.1     TTGAACTAAAATTAGTTTTTTTTTTATTATTATTTTATTAATTGGGCTATTTCATGAATGAAACCAAAATATATTAAATTGAACTAAATAAAAAATAAAT
2 MT492036.1     TTGAACTAAAATTAGTTTTTTTTTTATTATTATTTTATTAATTGGACTATTTCATGAATGAAACCAAAATATATTAAATTGAACTAAATAAAAAATAAAT
3 MT492037.1     TTGAACTAAAATTAGTTTTTTTTTTATTATTATTTTATTAATTGGGCTATTTCATGAATGAAACCAAAATATATTAAATTGAACTAAATAAAAAATAAAT
4 MT492030.1     TTGAACTAAAATTAGTTTTTTTTTTATTATTATTTTATTAATTGGACTATTTCATGAATGAAACCAAAATATATTAAATTGAACTAAATAAAAAATAAAT
5 MT492031.1     TTGAACTAAAATTAGTTTTTTTTTTATTATTATTTTATTAATTGGACTATTTCATGAATGAAACCAAAATATATTAAATTGAACTAAATAAAAAATAAAT
6 MT492032.1     TTGAACTAAAATTAGTTTTTTTTTTATTATTATTTTATTAATTGGGCTATTTCATGAATGAAACCAAAATATATTAAATTGAACTAAATAAAAAATAAAT
7 MT492033.1     TTGAACTAAAATTAGTTTTTTTTTTATTATTATTTTATTAATTGGGCTATTTCATGAATGAAACCAAAATATATTAAATTGAACTAAATAAAAAATAAAT
8 MT492034.1     TTGAACTAAAATTAGTTTTTTTTTTATTATTATTTTATTAATTGGGCTATTTCATGAATGAAACCAAAATATATTAAATTGAACTAAATAAAAAATAAAT
9 MT492035.1     TTGAACTAAAATTAGTTTTTTTTTTATTATTATTTTATTAATTGGGCTATTTCATGAATGAAACCAAAATATATTAAATTGAACTAAATAAAAAATAAAT

                 | tRNA-Ala (UGC) ->                                         | tRNA-Arg (UCG) ->
              6201      .         .         .         .         :         .         .         .         .         3 6300
                 Aggattatagtttaaataaaacatttgatttgcactcaaaaaatattgaataaatcaatttatctta
A                                                                                  aaataagaagc-aatatttgcatttaatttcga
1 MN793322.1     AAGGATTATAGTTTAAATAAAACATTTGATTTGCACTCAAAAAATATTGAATAAATCAATTTATCTTAAATAAGAAGC-AATATTTGCATTTAATTTCGA
2 MT492036.1     AAGGATTATAGTTTAAATAAAACATTTGATTTGCACTCAAAAAATATTGAATAAATCAATTTATCTTAAATAAGAAGC-AATATTTGCATTTAATTTCGA
3 MT492037.1     AAGGATTATAGTTTAAATAAAACATTTGATTTGCACTCAAAAAATATTGAATAAATCAATTTATCTTAAATAAGAAGC-AATATTTGCATTTAATTTCGA
4 MT492030.1     AAGGATTATAGTTTAAATAAAACATTTGATTTGCACTCAAAAAATATTGAATAAATCAATTTATCTTAAATAAGAAGC-AATATTTGCATTTAATTTCGA
5 MT492031.1     AAGGATTATAGTTTAAATAAAACATTTGATTTGCACTCAAAAAATATTGAATAAATCAATTTATCTTAAATAAGAAGC-AATATTTGCATTTAATTTCGA
6 MT492032.1     AAGGATTATAGTTTAAATAAA-CATTTGATTTGCACTCAAAAAATATTGAATAA-TCAATTTATCTTAAATAATTAATGAATATTTGCATTTAATTTCGA
7 MT492033.1     AAGGATTATAGTTTAAATAAAACATTTGATTTGCACTCAAAAAATATTGAATAAATCAATTTATCTTAAATAAGAAGC-AATATTTGCATTTAATTTCGA
8 MT492034.1     AAGGATTATAGTTTAAATAAAACATTTGATTTGCACTCAAAAAATATTGAATAAATCAATTTATCTTAAATAAGAAGC-AATATTTGCATTTAATTTCGA
9 MT492035.1     AAGGATTATAGTTTAAATAAAACATTTGATTTGCACTCAAAAAATATTGAATAAATCAATTTATCTTAAATAAGAAGC-AATATTTGCATTTAATTTCGA

                                                 | tRNA-Asn (GUU) ->
              6301      .         .         .         .         :         .         .         .         .         4 6400
                                                 ttagttgaaaccaaaatagaggtatatcactgttaatgataaaattgaattataaattccaactaaa
                 cttaaaagatagagttttaatactccttatttt
1 MN793322.1     CTTAAAAGATAGAGTTTTAATACTCCTTATTTTTAGTTGAAACCAAAATAGAGGTATATCACTGTTAATGATAAAATTGAATTATAAATTCCAACTAAAA
2 MT492036.1     CTTAAAAGATAGAGTTTTAATACTCCTTATTTTTAGTTGAAACCAAAATAGAGGTATATCACTGTTAATGATAAAATTGAATTATAAATTCCAACTAAAA
3 MT492037.1     CTTAAAAGATAGAGTTTTAATACTCCTTATTTTTAGTTGAAACCAAAATAGAGGTATATCACTGTTAATGATAAAATTGAATTATAAATTCCAACTAAAA
4 MT492030.1     CTTAAAAGATAGAGTTTTAATACTCCTTATTTTTAGTTGAAACCAAAATAGAGGTATATCACTGTTAATGATAAAATTGAATTATAAATTCCAACTAAAA
5 MT492031.1     CTTAAAAGATAGAGTTTTAATACTCCTTATTTTTAGTTGAAACCAAAATAGAGGTATATCACTGTTAATGATAAAATTGAATTATAAATTCCAACTAAAA
6 MT492032.1     CTTAAAAGATAGAGTTTTAATACTCCTTATTTTTAGTTGAAACCAAAATAGAGGTATATCACTGTTAATGATAAAATTGAATTATAAATTCCAACTAAAA
7 MT492033.1     CTTAAAAGATAGAGTTTTAATACTCCTTATTTTTAGTTGAAACCAAAATAGAGGTATATCACTGTTAATGATAAAATTGAATTATAAATTCCAACTAAAA
8 MT492034.1     CTTAAAAGATAGAGTTTTAATACTCCTTATTTTTAGTTGAAACCAAAATAGAGGTATATCACTGTTAATGATAAAATTGAATTATAAATTCCAACTAAAA
```

```
9 MT492035.1     CTTAAAAGATAGAGTTTTAATACTCCTTATTTTTAGTTGAAACCAAAATAGAGGTATATCACTGTTAATGATAAAATTGAATTATAAATTCCAACTAAAA


                  | tRNA-Ser (GCU) ->                                                 | tRNA-Gln (UUC) ->
             6401          .         .         .         .         :         .         .         .         .         5 6500
                  Gaaatataaagtataaaattaagctgctaacttaattttagtggtttaattccattaatatttct atttatatagtttaatatataaaacattacattt
1 MN793322.1     AGAAATATAAAGTATAAAATTAAGCTGCTAACTTAATTTTTAGTGGTTTAATTCCATTAATATTTCTTATTTATATAGTTTAATATAAAACATTACATTT
2 MT492036.1     AGAAATATAAAGTATAAAATTAAGCTGCTAACTTAATTTTTAGTGGTTTAATTCCATTAATATTTCTTATTTATATAGTTTAATATAAAACATTACATTT
3 MT492037.1     AGAAATATAAAGTATAAAATTAAGCTGCTAACTTAATTTTTAGTGGTTTAATTCCATTAATATTTCTTATTTATATAGTTTAATATAAAACATTACATTT
4 MT492030.1     AGAAATATAAAGTATAAAATTAAGCTGCTAACTTAATTTTTAGTGGTTTAATTCCATTAATATTTCTTATTTATATAGTTTAATATAAAACATTACATTT
5 MT492031.1     AGAAATATAAAGTATAAAATTAAGCTGCTAACTTAATTTTTAGTGGTTTAATTCCATTAATATTTCTTATTTATATAGTTTAATATAAAACATTACATTT
6 MT492032.1     AGAAATATAAAGTATAAAATTAAGCTGCTAACTTAATTTTTAGTGGTTTAATTCCATTAATATTTCTTATTTATATAGTTTAATATAAAACATTACATTT
7 MT492033.1     AGAAATATAAAGTATAAAATTAAGCTGCTAACTTAATTTTTAGTGGTTTAATTCCATTAATATTTCTTATTTATATAGTTTAATATAAAACATTACATTT
8 MT492034.1     AGAAATATAAAGTATAAAATTAAGCTGCTAACTTAATTTTTAGTGGTTTAATTCCATTAATATTTCTTATTTATATAGTTTAATATAAAACATTACATTT
9 MT492035.1     AGAAATATAAAGTATAAAATTAAGCTGCTAACTTAATTTTTAGTGGTTTAATTCCATTAATATTTCTTATTTATATAGTTTAATATAAAACATTACATTT


             6501          .         .         .         .         :         .         .         .         .         6 6600
                 tcattgtaaaaataaaaaaaaaattttttataaata
1 MN793322.1     TCATTGTAAAAATAAAAAAAAAATTTTTTATAAATATTTAAAGATTTAAATAATCTCCTTAATATCTTCAATATTATGCTCTAAAATTATAAGCTATTTA
2 MT492036.1     TCATTGTAAAAATAAAAAAAAA-TTTTTTATAAATATTTAAAGATTTAAATAATCTCCTTAATATCTTCAATATTATGCTCTAAAATTATAAGCTATTTA
3 MT492037.1     TCATTGTAAAAATAAAAAAAAA-TTTTTTATAAATATTTAAAGATTTAAATAATCTCCTTAATATCTTCAATATTATGCTCTAAAATTATAAGCTATTTA
4 MT492030.1     TCATTGTAAAAATAAAAAAAAA-TTTTTTATAAATATTTAAAGATTTAAATAATCTCCTTAATATCTTCAATATTATGCTCTAAAATTATAAGCTATTTA
5 MT492031.1     TCATTGTAAAAATAAAAAAAAA-TTTTTTATAAATATTTAAAGATTTAAATAATCTCCTTAATATCTTCAATATTATGCTCTAAAATTATAAGCTATTTA
6 MT492032.1     TCATTGTAAAAATAAAAAAAAA-TTTTTTATAAATATTTAAAGATTTAAATAATCTCCTTAATATCTTCAATATTATGCTCTAAAATTATAAGCTATTTA
7 MT492033.1     TCATTGTAAAAATAAAAAAAAA-TTTTTTATAAATATTTAAAGATTTAAATAATCTCCTTAATATCTTCAATATTATGCTCTAAAATTATAAGCTATTTA
8 MT492034.1     TCATTGTAAAAATAAAAAAAAA-TTTTTTATAAATATTTAAAGATTTAAATAATCTCCTTAATATCTTCAATATTATGCTCTAAAATTATAAGCTATTTA
9 MT492035.1     TCATTGTAAAAATAAAAAAAAA-TTTTTTATAAATATTTAAAGATTTAAATAATCTCCTTAATATCTTCAATATTATGCTCTAAAATTATAAGCTATTTA
                                                   ataaatttctaaatttattagaggaattatagaagttataatacgagattttaatattcgataaat
                                                                                                   <- tRNA-Phe (GAA)


             6601          .         .         .         .         :         .         .         .         .         7 6700
1 MN793322.1     AATATAAAAAAATTATTAAAATAATTAATATAAATATTCATAAAACAAATCTAAATAAATAAATTTTATAATTTTTTATAGAAAATAAATTAAATAAAAT
2 MT492036.1     AATATAAAAAAATTATTAAAATAATTAATATAAATATTCATAAAACAAATCTAAATAAATAAATTTTATAATTTTTTATAGAAAATAAATTAAATAAAAT
3 MT492037.1     AATATAAAAAAATTATTAAAATAATTAATATAAATATTCATAAAACAAATCTAAATAAATAAATTTTATAATTTTTTATAGAAAATAAATTAAATAAAAT
4 MT492030.1     AATATAAAAAAATTATTAAAATAATTAATATAAATATTCATAAAACAAATCTAAATAAATAAATTTTATAATTTTTTATAGAAAATAAATTAAATAAAAT
5 MT492031.1     AATATAAAAAAATTATTAAAATAATTAATATAAATATTCATAAAACAAATCTAAATAAATAAATTTTATAATTTTTTATAGAAAATAAATTAAATAAAAT
6 MT492032.1     AATATAAAAAAATTATTAAAATAATTAATATAAATATTCATAAAACAAATCTAAATAAATAAATTTTATAATTTTTTATAGAAAATAAATTAAATAAAAT
7 MT492033.1     AATATAAAAAAATTATTAAAATAATTAATATAAATATTCATAAAACAAATCTAAATAAATAAATTTTATAATTTTTTATAGAAAATAAATTAAATAAAAT
8 MT492034.1     AATATAAAAAAATTATTAAAATAATTAATATAAATATTCATAAAACAAATCTAAATAAATAAATTTTATAATTTTTTATAGAAAATAAATTAAATAAAAT
9 MT492035.1     AATATAAAAAAATTATTAAAATAATTAATATAAATATTCATAAAACAAATCTAAATAAATAAATTTTATAATTTTTTATAGAAAATAAATTAAATAAAAT
                 tta* L  F  I  M  L  I  I  L  M  F  M  W  L  V  F  S  F  L  Y  I  K  Y  N  K  M  S  F  L  N  F  L  I
                    TATTTTTTTAATAATTTTATTAATTATATTTATAAGTATTTTGTTTAGATTTATTTATTTAAAATATTAAAAAATATCTTTTATTTAATTTATTTTA


             6701          .         .         .         .         :         .         .         .         .         8 6800
1 MN793322.1     TGAATATTTTTTTATAATAAAAAATATACCTTGACCTCTATATATTTCTCTTCAACCCATATCAATATTTTTTAATATTATTTGACCAAAATTTAAAAAA
2 MT492036.1     TGAGTATTTTTTTATAATAAAAAATATACCTTGACCTCTATATATTTCTCTTCAACCCATATCAATATTTTTTAATATTATTTGACCAAAATTTAAAAAA
3 MT492037.1     TGAGTATTTTTTTATAATAAAAAATATACCTTGACCTCTATATATTTCTCTTCAACCCATATCAATATTTTTTAATATTATTTGACCAAAATTTAAAAAA
4 MT492030.1     TGAGTATTTTTTTATAATAAAAAATATACCTTGACCTCTATATATTTCTCTTCAACCCATATCAATATTTTTTAATATTATTTGACCAAAATTTAAAAAA
5 MT492031.1     TGAGTATTTTTTTATAATAAAAAATATACCTTGACCTCTATATATTTCTCTTCAACCCATATCAATATTTTTTAATATTATTTGACCAAAATTTAAAAAA
6 MT492032.1     TGAGTATTTTTTTATAATAAAAAATATACCTTGACCTCTATATATTTCTCTTCAACCCATATCAATATTTTTTAATATTATTTGACCAAAATTTAAAAAA
7 MT492033.1     TGAGTATTTTTTTATAATAAAAAATATACCTTGACCTCTATATATTTCTCTTCAACCCATATCAATATTTTTTAATATTATTTGACCAAAATTTAAAAAA
8 MT492034.1     TGAGTATTTTTTTATAATAAAAAATATACCTTGACCTCTATATATTTCTCTTCAACCCATATCAATATTTTTTAATATTATTTGACCAAAATTTAAAAAA
9 MT492035.1     TGAGTATTTTTTTATAATAAAAAATATACCTTGACCTCTATATATTTCTCTTCAACCCATATCAATATTTTTTAATATTATTTGACCAAAATTTAAAAAA
                  S  Y  K  K  M  I  F  F  M  G  Q  G  S  Y  M  E  S  W  G  M  D  I  N  K  L  M  M  Q  G  F  N  L  F
```

```
                 ACTCATAAAAAAATATTATTTTTTATATGGAACTGGAGATATATAAAGAGAAGTTGGGTATAGTTATAAAAAATTATAATAAACTGGTTTTAAATTTTTT

             6801              .         .         .         .         :         .         .         .         .    9 6900
1 MN793322.1     TAAAAATTTAAACCATAAGTAGATAAATTAGGTATAAATCATATTAAACATAAAAAATTTCTAATTTGATAAGTTATTAAAAATTTATTAACAGAATAAA
2 MT492036.1     TAAAAATTTAAACCATAAGTAGATAAATTAGGTATAAATCATATTAAACATAAAAAATTTCTAATTTGATAAGTTATTAAAAATTTATTAACAGAATAAA
3 MT492037.1     TAAAAATTTAAACCATAAGTAGATAAATTAGGTATAAATCATATTAAACATAAAAAATTTCTAATTTGATAAGTTATTAAAAATTTATTAACAGAATAAA
4 MT492030.1     TAAAAATTTAAACCATAAGTAGATAAATTAGGTATAAATCATATTAAACATAAAAAATTTCTAATTTGATAAGTTATTAAAAATTTATTAACAGAATAAA
5 MT492031.1     TAAAAATTTAAACCATAAGTAGATAAATTAGGTATAAATCATATTAAACATAAAAAATTTCTAATTTGATAAGTTATTAAAAATTTATTAACAGAATAAA
6 MT492032.1     TAAAAATTTAAACCATAAGTAGATAAATTAGGTATAAATCATATTAAACATAAAAAATTTCTAATTTGATAAGTTATTAAAAATTTATTAACAGAATAAA
7 MT492033.1     TAAAAATTTAAACCATAAGTAGATAAATTAGGTATAAATCATATTAAACATAAAAAATTTCTAATTTGATAAGTTATTAAAAATTTATTAACAGAATAAA
8 MT492034.1     TAAAAATTTAAACCATAAGTAGATAAATTAGGTATAAATCATATTAAACATAAAAAATTTCTAATTTGATAAGTTATTAAAAATTTATTAACAGAATAAA
9 MT492035.1     TAAAAATTTAAACCATAAGTAGATAAATTAGGTATAAATCATATTAAACATAAAAAATTTCTAATTTGATAAGTTATTAAAAATTTATTAACAGAATAAA
                 Y  F  N  L  G  Y  T  S  L  N  P  M  F  W  M  L  C  L  F  N  S  I  Q  Y  T  M  L  F  K  N  V  S  Y  I
                 ATTTTTAAATTTGGTATTCATCTATTTAATCCATATTTAGTATAATTTGTATTTTTTAAAGATTAAACTATTCAATAATTTTTAAATAATTGTCTTATTT


             6901              .         .         .         .         :         .         .         .         .    0 7000
1 MN793322.1     TATTTATATTTCTAACAATAAACCCCATAAAAACCCCTAAAATTCTTACATAAATAACTATTATTTTTATATTAAAAGGTAAAAAAATTATATAAGGATA
2 MT492036.1     TATTTATATTTCTAACAATAAACCCCATAAAAACCCCTAAAATTCTTACATAAATAACTATTATTTTTATATTAAAAGGTAAAAAAATTATATAAGGATA
3 MT492037.1     TATTTATATTTCTAACAATAAACCCCATAAAAACCCCTAAAATTCTTACATAAATAACTATTATTTTTATATTAAAAGGTAAAAAAATTATATAAGGATA
4 MT492030.1     TATTTATATTTCTAACAATAAACCCCATAAAAACCCCTAAAATTCTTACATAAATAACTATTATTTTTATATTAAAAGGTAAAAAAATTATATAAGGATA
5 MT492031.1     TATTTATATTTCTAACAATAAACCCCATAAAAACCCCTAAAATTCTTACATAAATAACTATTATTTTTATATTAAAAGGTAAAAAAATTATATAAGGATA
6 MT492032.1     TATTTATATTTCTAACAATAAACCCCATAAAAACCCCTAAAATTCTTACATAAATAACTATTATTTTTATATTAAAAGGTAAAAAAATTATATAAGGATA
7 MT492033.1     TATTTATATTTCTAACAATAAACCCCATAAAAACCCCTAAAATTCTTACATAAATAACTATTATTTTTATATTAAAAGGTAAAAAAATTATATAAGGATA
8 MT492034.1     TATTTATATTTCTAACAATAAACCCCATAAAAACCCCTAAAATTCTTACATAAATAACTATTATTTTTATATTAAAAGGTAAAAAAATTATATAAGGATA
9 MT492035.1     TATTTATATTTCTAACAATAAACCCCATAAAAACCCCTAAAATTCTTACATAAATAACTATTATTTTTATATTAAAAGGTAAAAAAATTATATAAGGATA
                   N  M  N  S  V  I  F  G  M  F  V  G  L  I  S  V  Y  I  V  M  M  K  M  N  F  P  L  F  I  M  Y  P  Y
                 ATAAATATAAAGATTGTTATTTGGGGTATTTTTGGGGATTTTAAGAATGTATTTATTGATAATAAAAATATAATTTTCCATTTTTTTAATATATTCCTAT


             7001              .         .         .         .         :         .         .         .         .    1 7100
1 MN793322.1     AGAGAAAATTAATCATATTAAAAATCTACCTCTAATAACTCTTATAAATAATAAAATTATTATTCTTTTTAATATAATAAAATCTTCATCATATAAATTA
2 MT492036.1     AGAGAAAATTAATCATATTAAAAATCTACCTCTAATAACTCTTATAAATAATAAAATTATTATTCTTTTTAATATAATAAAATCTTCATCATATAAATTA
3 MT492037.1     AGAGAAAATTAATCATATTAAAAATCTACCTCTAATAACTCTTATAAATAATAAAATTATTATTCTTTTTAATATAATAAAATCTTCATCATATAAATTA
4 MT492030.1     AGAGAAAATTAATCATATTAAAAATCTACCTCTAATAACTCTTATAAATAATAAAATTATTATTCTTTTTAATATAATAAAATCTTCATCATATAAATTA
5 MT492031.1     AGAGAAAATTAATCATATTAAAAATCTACCTCTAATAACTCTTATAAATAATAAAATTATTATTCTTTTTAATATAATAAAATCTTCATCATATAAATTA
6 MT492032.1     AGAGAAAATTAATCATATTAAAAATCTACCTCTAATAACTCTTATAAATAATAAAATTATTATTCTTTTTAATATAATAAAATCTTCATCATATAAATTA
7 MT492033.1     AGAGAAAATTAATCATATTAAAAATCTACCTCTAATAACTCTTATAAATAATAAAATTATTATTCTTTTTAATATAATAAAATCTTCATCATATAAATTA
8 MT492034.1     AGAGAAAATTAATCATATTAAAAATCTACCTCTAATAACTCTTATAAATAATAAAATTATTATTCTTTTTAATATAATAAAATCTTCATCATATAAATTA
9 MT492035.1     AGAGAAAATTAATCATATTAAAAATCTACCTCTAATAACTCTTATAAATAATAAAATTATTATTCTTTTTAATATAATAAAATCTTCATCATATAAATTA
                  S  F  I  L  W  M  L  F  S  G  S  I  V  S  M  F  L  L  I  M  M  S  K  L  M  I  F  D  E  D  Y  L  N
                 TCTCTTTTAATTAGTATAATTTTTAGATGGAGATTATTGAGAATATTTATTATTTTAATAATAAGAAAAATTATATTATTTTAGAAGTAGTATATTTAAT


             7101              .         .         .         .         :         .         .         .         .    2 7200
1 MN793322.1     TAAATAGATAATAAATTATAATCATTAATTATTAAATATATAGTTAAACGAAAACTATAAAATATAGTTAAACCTGTAGAAATATAATATAATAAAAAAA
2 MT492036.1     TAAATAGATAATAAATTATAATCATTAATTATTAAATATATAGTTAAACGAAAACTATAAAATATAGTTAAACCTGTAGAAATATAATATAATAAAAAAA
3 MT492037.1     TAAATAGATAATAAATTATAATCATTAATTATTAAATATATAGTTAAACGAAAACTATAAAATATAGTTAAACCTGTAGAAATATAATATAATAAAAAAA
4 MT492030.1     TAAATAGATAATAAATTATAATCATTAATTATTAAATATATAGTTAAACGAAAACTATAAAATATAGTTAAACCTGTAGAAATATAATATAATAAAAAAA
5 MT492031.1     TAAATAGATAATAAATTATAATCATTAATTATTAAATATATAGTTAAACGAAAACTATAAAATATAGTTAAACCTGTAGAAATATAATATAATAAAAAAA
6 MT492032.1     TAAATAGATAATAAATTATAATCATTAATTATTAAATATATAGTTAAACGAAAACTATAAAATATAGTTAAACCTGTAGAAATATAATATAATAAAAAAA
7 MT492033.1     TAAATAGATAATAAATTATAATCATTAATTATTAAATATATAGTTAAACGAAAACTATAAAATATAGTTAAACCTGTAGAAATATAATATAATAAAAAAA
8 MT492034.1     TAAATAGATAATAAATTATAATCATTAATTATTAAATATATAGTTAAACGAAAACTATAAAATATAGTTAAACCTGTAGAAATATAATATAATAAAAAAA
9 MT492035.1     TAAATAGATAATAAATTATAATCATTAATTATTAAATATATAGTTAAACGAAAACTATAAAATATAGTTAAACCTGTAGAAATATAATATAATAAAAAAA
                 Y  I  S  L  L  N  Y  D  N  I  M  L  Y  M  T  L  R  F  S  Y  F  M  T  L  G  T  S  I  Y  Y  L  L  F  I
                 ATTTATCTATTATTTAATATTAGTAATTAATAATTTATATATCAATTTGCTTTTGATATTTTATATCAATTTGGACATCTTTATATTATATTATTTTTTT
```

```
               7201        .         .         .         .         :         .         .         .         .        3 7300
1 MN793322.1     TAAAAAAATTTAAATTTCTTAAACTAACTATTTCTAAAATTAAATCCTTAGAATAAAACCCAGCTAAAAAAGGAATACCACATAAAGCTATATTAGACAC
2 MT492036.1     TAAAAAAATTTAAATTTCTTAAACTAACTATTTCTAAAATTAAATCCTTAGAATAAAACCCAGCTAAAAAAGGAATACCACATAAAGTTATATTAGACAC
3 MT492037.1     TAAAAAAATTTAAATTTCTTAAACTAACTATTTCTAAAATTAAATCCTTAGAATAAAACCCAGCTAAAAAAGGAATACCACATAAAGCTATATTAGACAC
4 MT492030.1     TAAAAAAATTTAAATTTCTTAAACTAACTATTTCTAAAATTAAATCCTTAGAATAAAACCCAGCTAAAAAAGGAATACCACATAAAGTTATATTAGACAC
5 MT492031.1     TAAAAAAATTTAAATTTCTTAAACTAACTATTTCTAAAATTAAATCCTTAGAATAAAACCCAGCTAAAAAAGGAATACCACATAAAGTTATATTAGACAC
6 MT492032.1     TAAAAAAATTTAAATTTCTTAAACTAACTATTTCTAAAATTAAATCCTTAGAATAAAACCCAGCTAAAAAAGGAATACCACATAAAGCTATATTAGACAC
7 MT492033.1     TAAAAAAATTTAAATTTCTTAAACTAACTATTTCTAAAATTAAATCCTTAGAATAAAACCCAGCTAAAAAAGGAATACCACATAAAGCTATATTAGACAC
8 MT492034.1     TAAAAAAATTTAAATTTCTTAAACTAACTATTTCTAAAATTAAATCCTTAGAATAAAACCCAGCTAAAAAAGGAATACCACATAAAGCTATATTAGACAC
9 MT492035.1     TAAAAAAATTTAAATTTCTTAAACTAACTATTTCTAAAATTAAATCCTTAGAATAAAACCCAGCTAAAAAAGGAATACCACATAAAGCTATATTAGACAC
                  F  F  N  L  N  S  L  S  V  M  E  L  I  L  D  K  S  Y  F  G  A  L  F  P  I  G  C  L  A  M  N  S  V
                 ATTTTTTTAAATTTAAAGAATTTGATTGATAAAGATTTTAATTTAGGAATCTTATTTTGGGTCGATTTTTTCCTTATGGTGTATTTCGATATAATCTGTG

               7301        .         .         .         .         :         .         .         .         .        4 7400
1 MN793322.1     ATTCATACATAAAGATGTCATAGGAATGAAATTTCTAATCCCCCCTATAAAACGAATATCTTGAATATCTATTATTATATGAATAATAACCCCAGCACAC
2 MT492036.1     ATTCATACATAAAGATGTCATAGGAATGAAATTTCTAACCCCCCCTATAAAACGAATATCTTGAATATCTATTATTATATGAATAATAACCCCAGCACAC
3 MT492037.1     ATTCATACATAAAGATGTCATAGGAATGAAATTTCTAATCCCCCCTATAAAACGAATATCTTGAATATCTATTATTATATGAATAATAACCCCAGCACAC
4 MT492030.1     ATTCATACATAAAGATGTCATAGGAATGAAATTTCTAACCCCCCCTATAAAACGAATATCTTGAATATCTATTATTATATGAATAATAACCCCAGCACAC
5 MT492031.1     ATTCATACATAAAGATGTCATAGGAATGAAATTTCTAACCCCCCCTATAAAACGAATATCTTGAATATCTATTATTATATGAATAATAACCCCAGCACAC
6 MT492032.1     ATTCATACATAAAGATGTCATAGGAATGAAATTTCTAATCCCCCCTATAAAACGAATATCTTGAATATCTATTATTATATGAATAATAACCCCAGCACAC
7 MT492033.1     ATTCATACATAAAGATGTCATAGGAATGAAATTTCTAATCCCCCCTATAAAACGAATATCTTGAATATCTATTATTATATGAATAATAACCCCAGCACAC
8 MT492034.1     ATTCATACATAAAGATGTCATAGGAATGAAATTTCTAATCCCCCCTATAAAACGAATATCTTGAATATCTATTATTATATGAATAATAACCCCAGCACAC
9 MT492035.1     ATTCATACATAAAGATGTCATAGGAATGAAATTTCTAATCCCCCCTATAAAACGAATATCTTGAATATCTATTATTATATGAATAATAACCCCAGCACAC
                  N  M  C  L  S  T  M  P  I  F  N  S  I  G  G  M  F  R  I  D  Q  I  D  M  M  M  H  I  I  V  G  A  C
                 TAAGTATGTATTTCTACAGTATCCTTACTTTAAAGATTAGGGGGGATATTTTGCTTATAGAACTTATAGATAATAATATACTTATTATTGGGGTCGTGTG

               7401        .         .         .         .         :         .         .         .         .        5 7500
1 MN793322.1     ATAAATAATAAAGCCTTAAATATAGCATGAGTTAATAAATGAAAAAAAGCTAAATCTGGTATTCCTATTCTTAAAATTCTTATTATTAATCCTAATTGTC
2 MT492036.1     ATAAATAATAAAGCCTTAAATATAGCATGAGTTAATAAATGAAAAAAAGCTAAATCTGGTATTCCTATTCTTAAAATTCTTATTATTAATCCTAATTGTC
3 MT492037.1     ATAAATAATAAAGCCTTAAATATAGCATGAGTTAATAAATGAAAAAAAGCTAAATCTGGTATTCCTATTCTTAAAATTCTTATTATTAATCCTAATTGTC
4 MT492030.1     ATAAATAATAAAGCCTTAAATATAGCATGAGTTAATAAATGAAAAAAAGCTAAATCTGGTATTCCTATTCTTAAAATTCTTATTATTAATCCTAATTGTC
5 MT492031.1     ATAAATAATAAAGCCTTAAATATAGCATGAGTTAATAAATGAAAAAAAGCTAAATCTGGTATTCCTATTCTTAAAATTCTTATTATTAATCCTAATTGTC
6 MT492032.1     ATAAATAATAAAGCCTTAAATATAGCATGAGTTAATAAATGAAAAAAAGCTAAATCTGGTATTCCTATTCTTAAAATTCTTATTATTAATCCTAATTGTC
7 MT492033.1     ATAAATAATAAAGCCTTAAATATAGCATGAGTTAATAAATGAAAAAAAGCTAAATCTGGTATTCCTATTCTTAAAATTCTTATTATTAATCCTAATTGTC
8 MT492034.1     ATAAATAATAAAGCCTTAAATATAGCATGAGTTAATAAATGAAAAAAAGCTAAATCTGGTATTCCTATTCTTAAAATTCTTATTATTAATCCTAATTGTC
9 MT492035.1     ATAAATAATAAAGCCTTAAATATAGCATGAGTTAATAAATGAAAAAAAGCTAAATCTGGTATTCCTATTCTTAAAATTCTTATTATTAATCCTAATTGTC
                 M  F  L  L  A  K  F  M  A  H  T  L  L  H  F  F  A  L  D  P  M  G  M  S  L  I  S  M  M  L  G  L  Q  S
                 TATTTATTATTTCGGAATTTATATCGTACTCAATTATTTACTTTTTTTCGATTTAGACCATAAGGATAAGAATTTTAAGAATAATAATTAGGATTAACAG

               7501        .         .         .         .         :         .         .         .         .        6 7600
1 MN793322.1     TTAAAGTCGATAAAGCAATAATTTTTTTTAAATCAAACTCATAATTAGCAGAAATACCAGCTATAAACATAGTTAAACCAGATAATAATAATAAAATTTT
2 MT492036.1     TTAAAGTCGATAAAGCAATAATTTTTTTTAAATCAAACTCATAATTAGCAGAAATACCAGCTATAAACATAGTTAAACCAGATAATAATAATAAAATTTT
3 MT492037.1     TTAAAGTCGATAAAGCAATAATTTTTTTTAAATCAAACTCATAATTAGCAGAAATACCAGCTATAAACATAGTTAAACCAGATAATAATAATAAAATTTT
4 MT492030.1     TTAAAGTCGATAAAGCAATAATTTTTTTTAAATCAAACTCATAATTAGCAGAAATACCAGCTATAAACATAGTTAAACCAGATAATAATAATAAAATTTT
5 MT492031.1     TTAAAGTCGATAAAGCAATAATTTTTTTTAAATCAAACTCATAATTAGCAGAAATACCAGCTATAAACATAGTTAAACCAGATAATAATAATAAAATTTT
6 MT492032.1     TTAAAGTCGATAAAGCAATAATTTTTTTTAAATCAAACTCATAATTAGCAGAAATACCAGCTATAAACATAGTTAAACCAGATAATAATAATAAAATTTT
7 MT492033.1     TTAAAGTCGATAAAGCAATAATTTTTTTTAAATCAAACTCATAATTAGCAGAAATACCAGCTATAAACATAGTTAAACCAGATAATAATAATAAAATTTT
8 MT492034.1     TTAAAGTCGATAAAGCAATAATTTTTTTTAAATCAAACTCATAATTAGCAGAAATACCAGCTATAAACATAGTTAAACCAGATAATAATAATAAAATTTT
9 MT492035.1     TTAAAGTCGATAAAGCAATAATTTTTTTTAAATCAAACTCATAATTAGCAGAAATACCAGCTATAAACATAGTTAAACCAGATAATAATAATAAAATTTT
                  L  T  S  L  A  I  I  K  K  L  D  F  E  Y  N  A  S  I  G  A  M  F  M  T  L  G  S  L  L  L  L  I  K
                 AATTTCAGCTATTTCGTTATTAAAAAAAAATTTAGTTTGAGTATTAATCGTCTTTATGGTCGATATTTGTATCAATTTGGTCTATTATTATTATTTTAAAA
```

```
            7601       .         .         .         .         :         .         .         .         .        7 7700
1 MN793322.1    AATAAATATTATATCTAGTAATAAAAAATTAAAACGAATTAATAAATAAATACCCGCAGTAACTAAAGTAGATGAATGAACTAAAGCAGAAACTGGGGTA
2 MT492036.1    AATAAATATTATATCTAGTAATAAAAAATTAAAACGAATTAATAAATAAATACCCGCAGTAACTAAAGTAGATGAATGAACTAAAGCAGAAACTGGGGTA
3 MT492037.1    AATAAATATTATATCTAATAATAAAAAATTAAAACGAATTAATAAATAAATACCCGCAGTAACTAAAGTAGATGAATGAACTAAAGCAGAAACTGGGGTA
4 MT492030.1    AATAAATATTATATCTAGTAATAAAAAATTAAAACGAATTAATAAATAAATACCCGCAGTAACTAAAGTAGATGAATGAACTAAAGCAGAAACTGGGGTA
5 MT492031.1    AATAAATATTATATCTAGTAATAAAAAATTAAAACGAATTAATAAATAAATACCCGCAGTAACTAAAGTAGATGAATGAACTAAAGCAGAAACTGGGGTA
6 MT492032.1    AATAAATATTATATCTAATAATAAAAAATTAAAACGAATTAATAAATAAATACCCGCAGTAACTAAAGTAGATGAATGAACTAAAGCAGAAACTGGGGTA
7 MT492033.1    AATAAATATTATATCTAGTAATAAAAAATTAAAACGAATTAATAAATAAATACCCGCAGTAACTAAAGTAGATGAATGAACTAAAGCAGAAACTGGGGTA
8 MT492034.1    AATAAATATTATATCTAATAATAAAAAATTAAAACGAATTAATAAATAAATACCCGCAGTAACTAAAGTAGATGAATGAACTAAAGCAGAAACTGGGGTA
9 MT492035.1    AATAAATATTATATCTAATAATAAAAAATTAAAACGAATTAATAAATAAATACCCGCAGTAACTAAAGTAGATGAATGAACTAAAGCAGAAACTGGGGTA
                 I  F  M  M  D  L  L  L  F  N  F  R  I  L  L  Y  I  G  A  T  V  L  T  S  S  H  V  L  A  S  V  P  T
                TTATTTATAATATAGATCATTATTTTTTAATTTTGCTTAATTATTTATTTATGGGCGTCATTGATTTCATCTACTTACTTGATTTCGTCTTTGACCCCAT

            7701       .         .         .         .         :         .         .         .         .        8 7800
1 MN793322.1    GGAGCTGCTATAGCAGCTGGTAATCAGGAACTAAAAGGAATTTGAGCACTTTTAGTTATAGCAGCAATAATAATTATAATTCTAATTATAAATATTGAAT
2 MT492036.1    GGAGCTGCTATAGCAGCTGGTAATCAGGAACTAAAAGGAATTTGAGCACTTTTAGTTATAGCAGCAATAATAATTATAATTCTAATTATAAATATTGAAT
3 MT492037.1    GGAGCTGCTATAGCAGCTGGTAATCAGGAACTAAAAGGAATTTGAGCACTTTTAGTTATAGCAGCAATAATAATTATAATTCTAATTATAAATATTGAAT
4 MT492030.1    GGAGCTGCTATAGCAGCTGGTAATCAGGAACTAAAAGGAATTTGAGCACTTTTAGTTATAGCAGCAATAATAATTATAATTCTAATTATAAATATTGAAT
5 MT492031.1    GGAGCTGCTATAGCAGCTGGTAATCAGGAACTAAAAGGAATTTGAGCACTTTTAGTTATAGCAGCAATAATAATTATAATTCTAATTATAAATATTGAAT
6 MT492032.1    GGAGCTGCTATAGCAGCTGGTAATCAGGAACTAAAAGGAATTTGAGCACTTTTAGTTATAGCAGCAATAATAATTATAATTCTAATTATAAATATTGAAT
7 MT492033.1    GGAGCTGCTATAGCAGCTGGTAATCAGGAACTAAAAGGAATTTGAGCACTTTTAGTTATAGCAGCAATAATAATTATAATTCTAATTATAAATATTGAAT
8 MT492034.1    GGAGCTGCTATAGCAGCTGGTAATCAGGAACTAAAAGGAATTTGAGCACTTTTAGTTATAGCAGCAATAATAATTATAATTCTAATTATAAATATTGAAT
9 MT492035.1    GGAGCTGCTATAGCAGCTGGTAATCAGGAACTAAAAGGAATTTGAGCACTTTTAGTTATAGCAGCAATAATAATTATAATTCTAATTATAAATATTGAAT
                P  A  A  M  A  A  P  L  W  S  S  F  P  I  Q  A  S  K  T  M  A  A  I  I  I  M  I  S  I  M  F  M  S  Y
                CCTCGACGATATCGTCGACCATTAGTCCTTGATTTTCCTTAAACTCGTGAAAATCAATATCGTCGTTATTATTAATATTAAGATTAATATTTATAACTTA

            7801       .         .         .         .         :         .         .         .         .        9 7900
1 MN793322.1    AATCATTTTTTATAAAAGATAAATAAAATAAATAATTTCATCTACCATAATTTATTATTCAACCAATTACCATTAAAATAAATAAATCCCCAATTCGATT
2 MT492036.1    AATCATTTTTTATAAAAGATAAATAAAATAAATAATTTCATCTACCATAATTTATTATTCAACCAATTACCATTAAAATAAATAAATCCCCAATTCGATT
3 MT492037.1    AATCATTTTTTATAAAAGATAAATAAAATAAATAATTTCATCTACCATAATTTATTATTCAACCAATTACCATTAAAATAAATAAATCCCCAATTCGATT
4 MT492030.1    AATCATTTTTTATAAAAGATAAATAAAATAAATAATTTCATCTTCCATAATTTATTATTCAACCAATTACCATTAAAATAAATAAATCCCCAATTCGATT
5 MT492031.1    AATCATTTTTTATAAAAGATAAATAAAATAAATAATTTCATCTACCATAATTTATTATTCAACCAATTACCATTAAAATAAATAAATCCCCAATTCGATT
6 MT492032.1    AATCATTTTTTATAAAAGATAAATAAAATAAATAATTTCATCTACCATAATTTATTATTCAACCAATTACCATTAAAATAAATAAATCCCCAATTCGATT
7 MT492033.1    AATCATTTTTTATAAAAGATAAATAAAATAAATAATTTCATCTACCATAATTTATTATTCAACCAATTACCATTAAAATAAATAAATCCCCAATTCGATT
8 MT492034.1    AATCATTTTTTATAAAAGATAAATAAAATAAATAATTTCATCTACCATAATTTATTATTCAACCAATTACCATTAAAATAAATAAATCCCCAATTCGATT
9 MT492035.1    AATCATTTTTTATAAAAGATAAATAAAATAAATAATTTCATCTACCATAATTTATTATTCAACCAATTACCATTAAAATAAATAAATCCCCAATTCGATT
                  D  N  K  M  F  S  L  Y  F  L  Y  N  W  S  G  Y  N  M  M  W  G  I  V  M  L  I  F  L  D  G  I  R  N
                TTAGTAAAAAATATTTTCTATTTATTTTATTTATTAAAGTAGATGGTATTAAATAATAAGTTGGTTAATGGTAATTTTATTTATTTAGGGGTTAAGCTAA

            7901       .         .         .         .         :         .         .         .         .        0 8000
1 MN793322.1    AGATAAAGCAGTTAATATACCAGCATTATAAGATTTAATATTTTGATAATAAATAACCAAACAATAAGAAACTAAACCTAAACCATCTCAACCTAATAAA
2 MT492036.1    AGATAAAGCAGTTAATATACCAGCATTATAAGATTTAATATTTTGATAATAAATAACCAAACAATAAGAAACTAAACCTAAACCATCTCAACCTAATAAA
3 MT492037.1    AGATAAAGCAGTTAATATACCAGCATTATAAGATTTAATATTTTGATAATAAATAACCAAACAATAAGAAACTAAACCTAAACCATCTCAACCTAATAAA
4 MT492030.1    AGATAAAGCAGTTAATATACCAGCATTATAAGATTTAATATTTTGATAATAAATAACCAAACAATAAGAAACTAAACCTAAACCATCTCAACCTAATAAA
5 MT492031.1    AGATAAAGCAGTTAATATACCAGCATTATAAGATTTAATATTTTGATAATAAATAACCAAACAATAAGAAACTAAACCTAAACCATCTCAACCTAATAAA
6 MT492032.1    AGATAAAGCAGTTAATATACCAGCATTATAAGATTTAATATTTTGATAATAAATAACCAAACAATAAGAAACTAAACCTAAACCATCTCAACCTAATAAA
7 MT492033.1    AGATAAAGCAGTTAATATACCAGCATTATAAGATTTAATATTTTGATAATAAATAACCAAACAATAAGAAACTAAACCTAAACCATCTCAACCTAATAAA
8 MT492034.1    AGATAAAGCAGTTAATATACCAGCATTATAAGATTTAATATTTTGATAATAAATAACCAAACAATAAGAAACTAAACCTAAACCATCTCAACCTAATAAA
9 MT492035.1    AGATAAAGCAGTTAATATACCAGCATTATAAGATTTAATATTTTGATAATAAATAACCAAACAATAAGAAACTAAACCTAAACCATCTCAACCTAATAAA
                 S  L  A  T  L  M  G  A  N  Y  S  K  I  N  Q  Y  Y  I  V  L  C  Y  S  V  L  G  L  G  D  W  G  L  L
```

```
            TCTATTTCGTCAATTATATGGTCGTAATATTCTAAATTATAAAACTATTATTTATTGGTTTGTTATTCTTTGATTTGGATTTGGTAGAGTTGGATTATTT

        8001          .         .         .         .         :         .         .         .         .          1 8100
1 MN793322.1    ATTCTTACAATATTTGGACTAATAATTAATAAAATTATTGAAAAAACAAATAATAAAACTAAAATAATAAAACGATTTAAATTTAATTCAGAATTTATAT
2 MT492036.1    ATTCTTACAATATTTGGACTAATAATTAATAAAATTATTGAAAAAACAAATAATAAAACTAAAATAATAAAACGATTTAAATTTAATTCAGAATTTATAT
3 MT492037.1    ATTCTTACAATATTTGGACTAATAATTAATAAAATTATTGAAAAAACAAATAATAAAACTAAAATAATAAAACGATTTAAATTTAATTCAGAATTTATAT
4 MT492030.1    ATTCTTACAATATTTGGACTAATAATTAATAAAATTATTGAAAAAACAAATAATAAAACTAAAATAATAAAACGATTTAAATTTAATTCAGAATTTATAT
5 MT492031.1    ATTCTTACAATATTTGGACTAATAATTAATAAAATTATTGAAAAAACAAATAATAAAACTAAAATAATAAAACGATTTAAATTTAATTCAGAATTTATAT
6 MT492032.1    ATTCTTACAATATTTGGACTAATAATTAATAAAATTATTGACAAAACAACTAATAAAACTAAAATAATAAAACGATTTAAATTTAATTCAGAATTTATAT
7 MT492033.1    ATTCTTACAATATTTGGACTAATAATTAATAAAATTATTGAAAAAACAAATAATAAAACTAAAATAATAAAACGATTTAAATTTAATTCAGAATTTATAT
8 MT492034.1    ATTCTTACAATATTTGGACTAATAATTAATAAAATTATTGAAAAAACAAATAATAAAACTAAAATAATAAAACGATTTAAATTTAATTCAGAATTTATAT
9 MT492035.1    ATTCTTACAATATTTGGACTAATAATTAATAAAATTATTGAAAAAACAAATAATAAAACTAAAATAATAAAACGATTTAAATTTAATTCAGAATTTATAT
              I  S  V  I  N  P  S  I  I  L  L  I  M  S  F  V  F  L  L  V  L  I  I  F  R  N  L  N  L  E  S  N  M  Y
              TAAGAATGTTATAAACCTGATTATTAATTATTTTAATAACTTTTTTGTTTATTATTTTGATTTTATTATTTTGCTAAATTTAAATTAAGTCTTAAATATA

        8101          .         .         .         .         :         .         .         .         .          2 8200
1 MN793322.1    ATCTTTTACTATAATAAATTACAACTGAAGAAATTAACGAAACAAATATTATAAATAATAAAGATATTCAATCCAATAAAATTCTTATAATAACTCTTAA
2 MT492036.1    ATCTTTTACTATAATAAATTACAACTGAAGAAATTAACGAAACAAATATTATAAATAATAAAGATATTCAATCCAATAAAATTCTTATAATAACTCTTAA
3 MT492037.1    ATCTTTTACTATAATAAATTACAACTGAAGAAATTAACGAAACAAATATTATAAATAATAAAGATATTCAATCCAATAAAATTCTTATAATAACTCTTAA
4 MT492030.1    ATCTTTTACTATAATAAATTACAACTGAAGAAATTAACGAAACAAATATTATAAATAATAAAGATATTCAATCCAATAAAATTCTTATAATAACTCTTAA
5 MT492031.1    ATCTTTTACTATAATAAATTACAACGGAAGAAATTAACGAAACAAATATTATAAATAATAAAGATATTCAATCCAATAAAATTCTTATAATAACTCTTAA
6 MT492032.1    ATCTTTTACTATAATAAATTACAACTGAAGAAATTAACGAAACAAATATTATAAATAATAAAGATATTCAATCCAATAAAATTCTTATAATAACTCTTAA
7 MT492033.1    ATCTTTTACTATAATAAATTACAACTGAAGAAATTAACGAAACAAATATTATAAATAATAAAGATATTCAATCCAATAAAATTCTTATAATAACTCTTAA
8 MT492034.1    ATCTTTTACTATAATAAATTACAACTGAAGAAATTAACGAAACAAATATTATAAATAATAAAGATATTCAATCCAATAAAATTCTTATAATAACTCTTAA
9 MT492035.1    ATCTTTTACTATAATAAATTACAACTGAAGAAATTAACGAAACAAATATTATAAATAATAAAGATATTCAATCCAATAAAATTCTTATAATAACTCTTAA
                S  K  S  Y  Y  I  V  V  S  S  I  L  S  V  F  M  M  F  L  L  S  M  W  D  L  L  I  S  M  I  V  S  L
              TAGAAAATGATATTATTTAATGTTGACTTCTTTAATTGCTTTGTTTATAATATTTATTATTTCTATAAGTTAGGTTATTTTAAGAATATTATTGAGAATT

        8201          .         .         .         .         :         .         .         .         .          3 8300
1 MN793322.1    AGAATTAAAAGAAATAATTTCCCACTCTAAAATAATAATAATATTATTCATAATAAAATAAATTACTATAAAAAAATTTATTAATCTAAAAAAAAAATAAA
2 MT492036.1    AGAATTAAAAGAAATAATTTCCCACTCTAAAATAATAATAATATTATTCATAATAAAATAAATTACTATAAAAAAATTTATTAATCTAAAAAAAAAATAAA
3 MT492037.1    AGAATTAAAAGAAATAATTTCCCACTCTAAAATAATAATAATATTATTCATAATAAAATAAATTACTATAAAAAAATTTATTAATCTAAAAAAAAAATAAA
4 MT492030.1    AGAATTAAAAGAAATAATTTCCCACTCTAAAATAATAATAATATTATTCATAATAAAATAAATTACTATAAAAAAATTTATTAATCTAAAAAAAAAATAAA
5 MT492031.1    AGAATTAAAAGAAATAATTTCCCACTCTAAAATAATAATAATATTATTCATAATAAAATAAATTACTATAAAAAAATTTATTAATCTAAAAAAAAAATAAA
6 MT492032.1    AGAATTAAAAGAAATAATTTCCCACTCTAAAATAATAATAATATTATTCATAATAAAATAAATTACTATAAAAAAATTTATTAATCTAAAAAAAAAATAAA
7 MT492033.1    AGAATTAAAAGAAATAATTTCCCACTCTAAAATAATAATAATATTATTCATAATAAAATAAATTACTATAAAAAAATTTATTAATCTAAAAAAAAAATAAA
8 MT492034.1    AGAATTAAAAGAAATAATTTCCCACTCTAAAATAATAATAATATTATTCATAATAAAATAAATTACTATAAAAAAATTTATTAATCTAAAAAAAAAATAAA
9 MT492035.1    AGAATTAAAAGAAATAATTTCCCACTCTAAAATAATAATAATATTATTCATAATAAAATAAATTACTATAAAAAAATTTATTAATCTAAAAAAAAAATAAA
               S  N  F  S  I  I  E  W  E  L  I  I  I  I  N  N  M  I  F  Y  I  V  M  F  F  N  M  L  S  F  F  F  L
              TCTTAATTTTCTTTATTAAAGGGTGAGATTTTATTATTATTATAATAAGTATTATTTTATTTAATGATATTTTTTAAATAATTAGATTTTTTTTTATTT

        8301          .         .         .         .         :         .         .         .         .          4 8400
1 MN793322.1    AAAAAAAATCTAATAAAACAAATTGAAAATTTTTGAATAACCTAAAATGATACTATCATATTTTTGAAACCACAAATCAATATTTTAATTTTAAATTATT
2 MT492036.1    AAAAAAAATCTAATAAAACAAATTGAAAATTTTTGAATAACCTAAAATGATATTATCATATTTTTGAAACCACAAATCAATATTTTAATTTTAAATTATT
3 MT492037.1    AAAAAAAATCTAATAAAACAAATTGAAAATTTTTGAATAACCTAAAATGATATTATCATATTTTTGAAACCACAAATCAATATTTTAATTTTAAATTATT
4 MT492030.1    AAAAAAAATCTAATAAAACAAATTGAAAATTTTTGAATAACCTAAAATGATACTATCATATTTTTGAAACCACAAATCAATATTTTAATTTTAAATTATT
5 MT492031.1    AAAAAAAATCTAATAAAACAAATTGAAAATTTTTGAATAACCTAAAATGATATTATCATATTTTTGAAACCACAAATCAATATTTTAATTTTAAATTATT
6 MT492032.1    AAAAAAAATCTAATAAAACAAATTGAAAATTTTTGAATAACCTAAAATGATATTATCATATTTTTGAAACCACAAATCAATATTTTAATTTTAAATTATT
7 MT492033.1    AAAAAAAATCTAATAAAACAAATTGAAAATTTTTGAATAACCTAAAATGATATTATCATATTTTTGAAACCACAAATCAATATTTTAATTTTAAATTATT
8 MT492034.1    AAAAAAAATCTAATAAAACAAATTGAAAATTTTTGAATAACCTAAAATGATATTATCATATTTTTGAAACCACAAATCAATATTTTAATTTTAAATTATT
9 MT492035.1    AAAAAAAATCTAATAAAACAAATTGAAAATTTTTGAATAACCTAAAATGATATTATCATATTTTTGAAACCACAAATCAATATTTTAATTTTAAATTATT
              F  F  F  S  I  F  C  M                    ttggattttactatgatagtataaaaactttggtgtttagttataaaattaaaatttaataa
```

```
              TTTTTTTTAGATTATTTTGTTTA                                                          <- tRNA-His (GUG)
                            <- ND5 |

          8401          .         .         .         .         :         .         .         .         .         5 8500
1 MN793322.1  TAAGTTAAAATCAAATTATCACATAATCAATTTTTAAAATTAAAATATTTAAAGGAAATCAATGTAATATTAATAATAGGTATTCACGAGATACCCCCAT
2 MT492036.1  TAAGTTAAAATCAAATTATCACATAATCAATTTTTAAAATTAAAATATTTAAAGGAAATCAATGTAATATTAATAATAGGTATTCACGAGATACCCCCAT
3 MT492037.1  TAAGTTAAAATCAAATTATCACATAATCAATTTTTAAAATTAAAATATTTAAAGGAAATCAATGTAATATTAATAATAGGTATTCACGAGATACCCCCAT
4 MT492030.1  TAAGTTAAAATCAAATTATCACATAATCAATTTTTAAAATTAAAATATTTAAAGGAAATCAATGTAATATTAATAATAGGTATTCACGAGATACCCCCAT
5 MT492031.1  TAAGTTAAAATCAAATTATCACATAATCAATTTTTAAAATTAAAATATTTAAAGGAAATCAATGTAATATTAATAATAGGTATTCACGAGATACCCCCAT
6 MT492032.1  TAAGTTAAAATCAAATTATCACATAATCAATTTTTAAAATTAAAATATTTAAAGGAAATCAATGTAATATTAATAATAGGTATTCACGAGATACCCCCAT
7 MT492033.1  TAAGTTAAAATCAAATTATCACATAATCAATTTTTAAAATTAAAATATTTAAAGGAAATCAATGTAATATTAATAATAGGTATTCACGAGATACCCCCAT
8 MT492034.1  TAAGTTAAAATCAAATTATCACATAATCAATTTTTAAAATTAAAATATTTAAAGGAAATCAATGTAATATTAATAATAGGTATTCACGAGATACCCCCAT
9 MT492035.1  TAAGTTAAAATCAAATTATCACATAATCAATTTTTAAAATTAAAATATTTAAAGGAAATCAATGTAATATTAATAATAGGTATTCACGAGATACCCCCAT
              attca*  F  W  I  M  V  Y  D  I  K  L  I  L  I  N  L  P  F  W  H  L  M  L  L  L  Y  E  R  S  V  G  M
                 AATTTTAGTTTAATAGTGTATTAGTTAAAAATTTTAATTTTATAAATTTCCTTTAGTTACATTATAATTATTATCCATAAGTGCTCTATGGGGGTA

          8501          .         .         .         .         :         .         .         .         .         6 8600
1 MN793322.1  ATAAAATCTATAAATTCCTAAATAATATTTTCCATGTTGTGTATATGAATATAAATATAATCTATACCCAGCACTAAAAAAAGAATCAACATTAATATA
2 MT492036.1  ATAAAATCTATAAATTCCTAAATAATATTTTCCATGTTGTGTATATGAATATAAATATAATCTATACCCAGCACTAAAAAAAGAATCAACATTAATATA
3 MT492037.1  ATAAAATCTATAAATTCCTAAATAATATTTTCCATGTTGTGTATATGAATATAAATATAATCTATACCCAGCACTAAAAAAAGAATCAACATTAATATA
4 MT492030.1  ATAAAATCTATAAATTCCTAAATAATATTTTCCATGTTGTGTATATGAATATAAATATAATCTATACCCAGCACTAAAAAAAGAATCAACATTAATATA
5 MT492031.1  ATAAAATCTATAAATTCCTAAATAATATTTTCCATGTTGTGTATATGAATATAAATATAATCTATACCCAGCACTAAAAAAAGAATCAACATTAATATA
6 MT492032.1  ATAAAATCTATAAATTCCTAAATAATATTTTCCATGTTGTGTATATGAATATAAATATAATCTATACCCAGCACTAAAAAAAGAATCAACATTAATATA
7 MT492033.1  ATAAAATCTATAAATTCCTAAATAATATTTTCCATGTTGTGTATATGAATATAAATATAATCTATACCCAGCACTAAAAAAAGAATCAACATTAATATA
8 MT492034.1  ATAAAATCTATAAATTCCTAAATAATATTTTCCATGTTGTGTATATGAATATAAATATAATCTATACCCAGCACTAAAAAAAGAATCAACATTAATATA
9 MT492035.1  ATAAAATCTATAAATTCCTAAATAATATTTTCCATGTTGTGTATATGAATATAAATATAATCTATACCCAGCACTAAAAAAAGAATCAACATTAATATA
               Y  F  S  Y  I  G  L  Y  Y  K  G  H  Q  T  Y  S  Y  L  Y  L  S  Y  G  A  S  F  F  S  I  L  M  L  M
              TATTTTAGATATTTAAGGATTTATTATAAAAGGTACAACACATATACTTATATTTATATTAGATATGGGTCGTGATTTTTTTCTTTAGTTGTAATTATAT

          8601          .         .         .         .         :         .         .         .         .         7 8700
1 MN793322.1  AATATAGAGATATAAGATCATCTTAATAAACTATTAATTAATCTAATTTCACCTATTAAATTTAATGAGGGGGGAGCTGCTATATTAGAAGATAAAAGTA
2 MT492036.1  AATATAGAGATATAGGATCATCTTAATAAACTATTAATTAATCTAATTTCACCTATTAAATTTAATGAGGGGGGAGCTGCTATATTAGAAGATAAAAGTA
3 MT492037.1  AATATAGAGATATAAGATCATCTTAATAAACTATTAATTAATCTAATTTCACCTATTAAATTTAATGAGGGGGGAGCTGCTATATTAGAAGATAAAAGTA
4 MT492030.1  AATATAGAGATATAAGATCATCTTAATAAACTATTAATTAATCTAATTTCACCTATTAAATTTAATGAGGGGGGAGCTGCTATATTAGAAGATAAAAGTA
5 MT492031.1  AATATAGAGATATAGGATCATCTTAATAAACTATTAATTAATCTAATTTCACCTATTAAATTTAATGAGGGGGGAGCTGCTATATTAGAAGATAAAAGTA
6 MT492032.1  AATATAGAGATATAAGATCATCTTAATAAACTATTAATTAATCTAATTTCACCTATTAAATTTAATGAGGGGGGAGCTGCTATATTAGAAGATAAAAGTA
7 MT492033.1  AATATAGAGATATAAGATCATCTTAATAAACTATTAATTAATCTAATTTCACCTATTAAATTTAATGAGGGGGGAGCTGCTATATTAGAAGATAAAAGTA
8 MT492034.1  AATATAGAGATATAAGATCATCTTAATAAACTATTAATTAATCTAATTTCACCTATTAAATTTAATGAGGGGGGAGCTGCTATATTAGAAGATAAAAGTA
9 MT492035.1  AATATAGAGATATAAGATCATCTTAATAAACTATTAATTAATCTAATTTCACCTATTAAATTTAATGAGGGGGGAGCTGCTATATTAGAAGATAAAAGTA

              F  M  S  I  Y  S  W  S  L  L  S  N  I  L  S  I  E  G  M  L  N  L  S  P  P  A  A  M  N  S  S  L  L  L
              TTATATCTCTATATYCTAGTAGAATTATTTGATAATTAATTAGATTAAAGTGGATAATTTAAATTACTCCCCCCTCGACGATATAATCTTCTATTTTCAT

          8701          .         .         .         .         :         .         .         .         .         8 8800
1 MN793322.1  AAAATCATCATAAACTTATTGAAGGTATTAAATTTATTATACCCTTATTAATAAATAATCTTCGACTATTTAAGCGTTCATAATTAATATTAGCTAAACA
2 MT492036.1  AAAATCATCATCAACTTATTGAAGGTATTAAATTTATTATACCCTTATTAATAAATAATCTTCGACTATTTAAACGTTCATAATTAATATTAGCTAAACA
3 MT492037.1  AAAATCATCATAAACTTATTGAAGGTATTAAATTTATTATACCCTTATTAATAAATAATCTTCGACTATTTAAACGTTCATAATTAATATTAGCTAAACA
4 MT492030.1  AAAATCATCATAAACTTATTGAAGGTATTAAATTTATTATACCCTTATTAATAAATAATCTTCGACTATTTAAACGTTCATAATTAATATTAGCTAAACA
5 MT492031.1  AAAATCATCATAAACTTATTGAAGGTATTAAATTTATTATACCCTTATTAATAAATAATCTTCGACTATTTAAACGTTCATAATTAATATTAGCTAAACA
6 MT492032.1  AAAATCATCATAAACTTATTGAAGGTATTAAATTTATTATACCCTTATTAATAAATAATCTTCGACTATTTAAACGTTCATAATTAATATTAGCTAAACA
7 MT492033.1  AAAATCATCATAAACTTATTGAAGGTATTAAATTTATTATACCCTTATTAATAAATAATCTTCGACTATTTAAACGTTCATAATTAATATTAGCTAAACA
8 MT492034.1  AAAATCATCATAAACTTATTGAAGGTATTAAATTTATTATACCCTTATTAATAAATAATCTTCGACTATTTAAACGTTCATAATTAATATTAGCTAAACA
9 MT492035.1  AAAATCATCATAAACTTATTGAAGGTATTAAATTTATTATACCCTTATTAATAAATAATCTTCGACTATTTAAACGTTCATAATTAATATTAGCTAAACA
                F  W  W  L  S  M  S  P  M  L  N  M  M  G  K  N  I  F  L  S  R  S  N  L  R  E  Y  N  I  N  A  L  C
```

```
                    TTTTAGTAGTATTTGAATAACTTCCATAATTTAAATAATATGGGAATAATTATTTATTAGAAGCTGATAAATTYGCAAGTATTAATTATAATCGATTTGT

                8801          .         .         .         .         :         .         .         .         .         9 8900
1 MN793322.1        AAATATTCCTGAAGAACATAAACCATGACCAATCATTAAAATATATGAACCAATAAACCCCCAATAATTTATTGTTATAATACCCCCAATTACTATTCTT
2 MT492036.1        AAATATTCCTGAAGAACATAAACCATGACCAATCATTAAAATATATGAACCAATAAACCCCCAATAATTTATTGTTATAATACCCCCAATTACTATTCTT
3 MT492037.1        AAATATTCCTGAAGAACATAAACCATGACCAATCATTAAAATATATGAACCAATAAACCCCCAATAATTTATTGTTATAATACCCCCAATTACTATTCTT
4 MT492030.1        AAATATTCCTGAAGAACATAAACCATGACCAATCATTAAAATATATGAACCAATAAACCCCCAATAATTTATTGTTATAATACCCCCAATTACTATTCTT
5 MT492031.1        AAATATTCCTGAAGAACATAAACCATGACCAATCATTAAAATATATGAACCAATAAACCCCCAATAATTTATTGTTATAATACCCCCAATTACTATTCTT
6 MT492032.1        AAATATTCCTGAAGAACATAAACCATGACCAATCATTAAAATATATGAACCAATAAACCCCCAATAATTTATTGTTATAATACCCCCAATTACTATTCTT
7 MT492033.1        AAATATTCCTGAAGAACATAAACCATGACCAATCATTAAAATATATGAACCAATAAACCCCCAATAATTTATTGTTATAATACCCCCAATTACTATTCTT
8 MT492034.1        AAATATTCCTGAAGAACATAAACCATGACCAATCATTAAAATATATGAACCAATAAACCCCCAATAATTTATTGTTATAATACCCCCAATTACTATTCTT
9 MT492035.1        AAATATTCCTGAAGAACATAAACCATGACCAATCATTAAAATATATGAACCAATAAACCCCCAATAATTTATTGTTATAATACCCCCAATTACTATTCTT
                     F  M  G  S  S  C  L  G  H  G  I  M  L  I  Y  S  G  I  F  G  W  Y  N  M  T  M  I  G  G  I  V  M  S
                    TTTATAAGGACTTCTTGTATTTGGTACTGGTTAGTAATTTTATATACTTGGTTATTTGGGGGTTATTAAATAACAATATTATGGGGGTTAATGATAAGAA

                8901          .         .         .         .         :         .         .         .         .         0 9000
1 MN793322.1        ATATGTGCAACCGAAGAATAAGCAATTAAAGATTTAATATCAACTTGACAAAAACATTTTAAACTAATATAAAATCCTCCAACTAATCTAATAATAATTC
2 MT492036.1        ATATGTGCAACCGAAGAATAAGCAATTAAAGATTTAATATCAACTTGACAAAAACATTTTAAACTAATATAAAATCCTCCAACTAATCTAATAATAATTC
3 MT492037.1        ATATGTGCAACCGAAGAATAAGCAATTAAAGATTTAATATCAACTTGACAAAAACATTTTAAACTAATATAAAATCCTCCAACTAATCTAATAATAATTC
4 MT492030.1        ATATGTGCAACCGAAGAATAAGCAATTAAAGATTTAATATCAACTTGACAAAAACATTTTAAACTAATATAAAATCCTCCAACTAATCTAATAATAATTC
5 MT492031.1        ATATGTGCAACCGAAGAATAAGCAATTAAAGATTTAATATCAACTTGACAAAAACATTTTAAACTAATATAAAATCCTCCAACTAATCTAATAATAATTC
6 MT492032.1        ATATGTGCAACCGAAGAATAAGCAATTAAAGATTTAATATCAACTTGACAAAAACATTTTAAACTAATATAAAATCCTCCAACTAATCTAATAATAATTC
7 MT492033.1        ATATGTGCAACCGAAGAATAAGCAATTAAAGATTTAATATCAACTTGACAAAAACATTTTAAACTAATATAAAATCCTCCAACTAATCTAATAATAATTC
8 MT492034.1        ATATGTGCAACCGAAGAATAAGCAATTAAAGATTTAATATCAACTTGACAAAAACATTTTAAACTAATATAAAATCCTCCAACTAATCTAATAATAATTC
9 MT492035.1        ATATGTGCAACCGAAGAATAAGCAATTAAAGATTTAATATCAACTTGACAAAAACATTTTAAACTAATATAAAATCCTCCAACTAATCTAATAATAATTC
                    M  H  A  V  S  S  Y  A  I  L  S  K  I  D  V  Q  C  F  C  K  L  S  I  Y  F  G  G  V  L  S  I  I  I  W
                    TATACACGTTGGCTTCTTATTCGTTAATTTCTAAATTATAGTTGAACTGTTTTTGTAAAATTTGATTATATTTTAGGAGGTTGATTAGATTATTATTAAG

                9001          .         .         .         .         :         .         .         .         .         1 9100
1 MN793322.1        AAATAATATTAAACTTTAAACCCAATCTTTGAAATATAATTATAATACGTAATATTCCATAACCTCCTAATTTTAATATAATACCCGCTAAAATTATTGA
2 MT492036.1        AAATAATATTAAACTTTAAACCCAATCTTTGAAATATAATTATAATACGTAATATTCCATAACCTCCTAATTTTAATATAATACCCGCTAAAATTATTGA
3 MT492037.1        AAATAATATTAAACTTTAAACCCAATCTTTGAAATATAATTATAATACGTAATATTCCATAACCTCCTAATTTTAATATAATACCCGCTAAAATTATTGA
4 MT492030.1        AAATAATATTAAACTTTAAACCCAATCTTTGAAATATAATTATAATACGTAATATTCCATAACCTCCTAATTTTAATATAATACCCGCTAAAATTATTGA
5 MT492031.1        AAATAATATTAAACTTTAAACCCAATCTTTGAAATATAATTATAATACGTAATATTCCATAACCTCCTAATTTTAATATAATACCCGCTAAAATTATTGA
6 MT492032.1        AAATAATATTAAACTTTAAACCCAATCTTTGAAATATAATTATAATACGTAATATTCCATAACCTCCTAATTTTAATATAATACCCGCTAAAATTATTGA
7 MT492033.1        AAATAATATTAAACTTTAAACCCAATCTTTGAAATATAATTATAATACGTAATATTCCATAACCTCCTAATTTTAATATAATACCCGCTAAAATTATTGA
8 MT492034.1        AAATAATATTAAACTTTAAACCCAATCTTTGAAATATAATTATAATACGTAATATTCCATAACCTCCTAATTTTAATATAATACCCGCTAAAATTATTGA
9 MT492035.1        AAATAATATTAAACTTTAAACCCAATCTTTGAAATATAATTATAATACGTAATATTCCATAACCTCCTAATTTTAATATAATACCCGCTAAAATTATTGA
                      I  I  N  F  K  L  G  L  S  Q  F  M  I  M  I  R  L  M  G  Y  G  G  L  K  L  M  I  G  A  L  I  M  S
                    TTTATTATAATTTGAAATTTGGGTTAGAAACTTTATATTAATATTATGCATTATAAGGTATTGGAGGATTAAAATTATATTATGGGCGATTTTAATAACT

                9101          .         .         .         .         :         .         .         .         .         2 9200
1 MN793322.1        ACCTGATACAGGAGCTTCAACATGAGCTTTTGGTAATCATAAATGAACAAAATATATAGGTATTTTAACTAAAAATGCTATTATTATACAAAAATATAAT
2 MT492036.1        ACCTGATACAGGAGCTTCAACATGAGCTTTTGGTAATCATAAATGAACAAAATATATAGGTATTTTAACTAAAAATGCTATTATTATACAAAAATATAAT
3 MT492037.1        ACCTGATACAGGAGCTTCAACATGAGCTTTTGGTAATCATAAATGAACAAAATATATAGGTATTTTAACTAAAAATGCTATTATTATACAAAAATATAAT
4 MT492030.1        ACCTGATACAGGAGCTTCAACATGAGCTTTTGGTAATCATAAATGAACAAAATATATAGGTATTTTAACTAAAAATGCTATTATTATACAAAAATATAAT
5 MT492031.1        ACCTGATACAGGAGCTTCAACATGAGCTTTTGGTAATCATAAATGAACAAAATATATAGGTATTTTAACTAAAAATGCTATTATTATACAAAAATATAAT
6 MT492032.1        ACCTGATACAGGAGCTTCAACATGAGCTTTTGGTAATCATAAATGAACAAAATATATAGGTATTTTAACTAAAAATGCTATTATTATACAAAAATATAAT
7 MT492033.1        ACCTGATACAGGAGCTTCAACATGAGCTTTTGGTAATCATAAATGAACAAAATATATAGGTATTTTAACTAAAAATGCTATTATTATACAAAAATATAAT
8 MT492034.1        ACCTGATACAGGAGCTTCAACATGAGCTTTTGGTAATCATAAATGAACAAAATATATAGGTATTTTAACTAAAAATGCTATTATTATACAAAAATATAAT
9 MT492035.1        ACCTGATACAGGAGCTTCAACATGAGCTTTTGGTAATCATAAATGAACAAAATATATAGGTATTTTAACTAAAAATGCTATTATTATACAAAAATATAAT
                     G  S  V  P  A  E  V  H  A  K  P  L  W  L  H  V  F  Y  M  P  M  K  V  L  F  A  M  M  M  C  F  Y  L
```

```
          TGGACTATGTCCTCGAAGTTGTACTCGAAAACCATTAGTATTTACTTGTTTTATATATCCATAAAATTGATTTTTACGATAATAATATGTTTTTATATTA

      9201        .         .         .         .         :         .         .         .         .         3 9300
1 MN793322.1      AAATATAAATCAAAATTAAAAAACTTTAAAAAATATATTATTATTGAATTAGTATAAAAAAAAATATAAAAAAATTCCTAACAATAAAGGTAAAGAAACAA
2 MT492036.1      AAATATAAATCAAAATTAAAAAACTTTAAAAAATATATTATTATTGAATTAGTATAAAAAAAAATATAAAAAAATTCCTAACAATAAAGGTAAAGAAACAA
3 MT492037.1      AAATATAAATCAAAATTAAAAAACTTTAAAAAATATATTATTATTGAATTAGTATAAAAAAAAATATAAAAAAATTCCTAACAATAAAGGTAAAGAAACAA
4 MT492030.1      AAATATAAATCAAAATTAAAAAACTTTAAAAAATATATTATTATTGAATTAGTATAAAAAAAAATATAAAAAAATTCCTAACAATAAAGGTAAAGAAACAA
5 MT492031.1      AAATATAAATCAAAATTAAAAAACTTTAAAAAATATATTATTATTGAATTAGTATAAAAAAAAATATAAAAAAATTCCTAACAATAAAGGTAAAGAAACAA
6 MT492032.1      AAATATAAATCAAAATTAAAAAACTTTAAAAAATATATTATTATTGAATTAGTATAAAAAAAAATATAAAAAAATTCCTAACAATAAAGGTAAAGAAACAA
7 MT492033.1      AAATATAAATCAAAATTAAAAAACTTTAAAAAATATATTATTATTGAATTAGTATAAAAAAAAATATAAAAAAATTCCTAACAATAAAGGTAAAGAAACAA
8 MT492034.1      AAATATAAATCAAAATTAAAAAACTTTAAAAAATATATTATTATTGAATTAGTATAAAAAAAAATATAAAAAAATTCCTAACAATAAAGGTAAAGAAACAA
9 MT492035.1      AAATATAAATCAAAATTAAAAAACTTTAAAAAATATATTATTATTGAATTAGTATAAAAAAAAATATAAAAAAATTCCTAACAATAAAGGTAAAGAAACAA
                  L  Y  L  D  F  N  F  F  K  L  F  Y  M  M  M  S  N  T  Y  F  F  I  Y  F  I  G  L  L  L  P  L  S  V  F
                  TTTATATTTAGTTTTAATTTTTTGAAATTTTTTATATAATAATAACTTAATCATATTTTTTTTTATATTTTTTTAAGGATTGTTATTTCCATTTCTTTGTT

      9301        .         .         .         .         :         .         .         .         .         4 9400
1 MN793322.1      ATAAAGTATAAAATAATAAATATATACCAGCCTGAATTCGCTCAGGCTGGTATCCCCACCCAATAATTAATATTAAAGTAGGAATTAATCTACCTTCAAA
2 MT492036.1      ATAAAGTATAAAATAATAAATATATACCAGCCTGAATTCGCTCAGGCTGGTATCCCCACCCAATAATTAATATTAAAGTAGGAATTAATCTACCTTCAAA
3 MT492037.1      ATAAAGTATAAAATAATAAATATATACCAGCCTGAATTCGCTCAGGCTGGTATCCCCACCCAATAATTAATATTAAAGTAGGAATTAATCTACCTTCAAA
4 MT492030.1      ATAAAGTATAAAATAATAAATATATACCAGCCTGAATTCGCTCAGGCTGGTATCCCCACCCAATAATTAATATTAAAGTAGGAATTAATCTACCTTCAAA
5 MT492031.1      ATAAAGTATAAAATAATAAATATATACCAGCCTGAATTCGCTCAGGCTGGTATCCCCACCCAATAATTAATATTAAAGTAGGAATTAATCTACCTTCAAA
6 MT492032.1      ATAAAGTATAAAATAATAAATATATACCAGCCTGAATTCGCTCAGGCTGGTATCCCCACCCAATAATTAATATTAAAGTAGGAATTAATCTACCTTCAAA
7 MT492033.1      ATAAAGTATAAAATAATAAATATATACCAGCCTGAATTCGCTCAGGCTGGTATCCCCACCCAATAATTAATATTAAAGTAGGAATTAATCTACCTTCAAA
8 MT492034.1      ATAAAGTATAAAATAATAAATATATACCAGCCTGAATTCGCTCAGGCTGGTATCCCCACCCAATAATTAATATTAAAGTAGGAATTAATCTACCTTCAAA
9 MT492035.1      ATAAAGTATAAAATAATAAATATATACCAGCCTGAATTCGCTCAGGCTGGTATCCCCACCCAATAATTAATATTAAAGTAGGAATTAATCTACCTTCAAA
                    L  T  Y  F  L  L  Y  M  G  A  Q  I  R  E  P  Q  Y  G  W  G  I  I  L  M  L  T  P  I  L  S  G  E  F
                  TATTTCATATTTTATTATTTATATATGGTCGGACTTAAGCGAGTCCGACCATAGGGGTGGGTTATTAATTATAATTTCATCCTTAATTAGATGGAAGTTT

      9401        .         .         .         .         :         .         .         .         .         5 9500
1 MN793322.1      AAATAAATAAAATATAAATAAATTTATCACTCTAAAAGTTATATATAATATAATTAATAAAAATATTAAATTAAAAATAAAAAAATTTATATAAAATCCT
2 MT492036.1      AAATAAATAAAATATAAATAAATTTATCACTCTAAAAGTTATATATAATATAATTAATAAAAATATTAAATTAAAAATAAAAAAATTTATATAAAATCCT
3 MT492037.1      AAATAAATAAAATATAAATAAATTTATCACTCTAAAAGTTATATATAATATAATTAATAAAAATATTAAATTAAAAATAAAAAAATTTATATAAAATCCT
4 MT492030.1      AAATAAATAAAATATAAATAAATTTATCACTCTAAAAGTTATATATAATATAATTAATAAAAATATTAAATTAAAAATAAAAAAATTTATATAAAATCCT
5 MT492031.1      AAATAAATAAAATATAAATAAATTTATCACTCTAAAAGTTATATATAATATAATTAATAAAAATATTAAATTAAAAATAAAAAAATTTATATAAAATCCT
6 MT492032.1      AAATAAATAAAATATAAATAAATTTATCACTCTAAAAGTTATATATAATATAATTAATAAAAATATTAAATTAAAAATAAAAAAATTTATATAAAATCCT
7 MT492033.1      AAATAAATAAAATATAAATAAATTTATCACTCTAAAAGTTATATATAATATAATTAATAAAAATATTAAATTAAAAATAAAAAAATTTATATAAAATCCT
8 MT492034.1      AAATAAATAAAATATAAATAAATTTATCACTCTAAAAGTTATATATAATATAATTAATAAAAATATTAAATTAAAAATAAAAAAATTTATATAAAATCCT
9 MT492035.1      AAATAAATAAAATATAAATAAATTTATCACTCTAAAAGTTATATATAATATAATTAATAAAAATATTAAATTAAAAATAAAAAAATTTATATAAAATCCT
                   F  L  Y  F  M  F  L  N  M  V  S  F  T  M  Y  L  M  I  L  L  F  M  L  N  F  I  F  F  N  M  Y  F  G
                  TTTATTTATTTTATATTTATTTAAATAGTGAGATTTTCAATATATATTATATTAATTATTTTTATAATTTAATTTTTATTTTTTTAAATATATTTTAGGA

      9501        .         .         .         .         :         .         .         .         .         6 9600
1 MN793322.1      ATTTTATATAAATTTTCTCTAGCTATAATTATTAAAATACAAATTCAAATTCTCAATAAAATTAAACCATATGATAATATATCACAAGAGTATATATATC
2 MT492036.1      ATTTTATATAAATTTTCTCTAGCTATAATTATTAAAATACAAATTCAAATTCTCAATAAAATTAAACCATATGATAATATATCACAAGAGTATATATATC
3 MT492037.1      ATTTTATATAAATTTTCTCTAGCTATAATTATTAAAATACAAATTCAAATTCTCAATAAAATTAAACCATATGATAATATATCACAAGAGTATATATATC
4 MT492030.1      ATTTTATATAAATTTTCTCTAGCTATAATTATTAAAATACAAATTCAAATTCTCAATAAAATTAAACCATATGATAATATATCACAAGAGTATATATATC
5 MT492031.1      ATTTTATATAAATTTTCTCTAGCTATAATTATTAAAATACAAATTCAAATTCTCAATAAAATTAAACCATATGATAATATATCACAAGAGTATATATATC
6 MT492032.1      ATTTTATATAAATTTTCTCTAGCTATAATTATTAAAATACAAATTCAAATTCTCAATAAAATTAAACCATATGATAATATATCACAAGAGTATATATATC
7 MT492033.1      ATTTTATATAAATTTTCTCTAGCTATAATTATTAAAATACAAATTCAAATTCTCAATAAAATTAAACCATATGATAATATATCACAAGAGTATATATATC
8 MT492034.1      ATTTTATATAAATTTTCTCTAGCTATAATTATTAAAATACAAATTCAAATTCTCAATAAAATTAAACCATATGATAATATATCACAAGAGTATATATATC
9 MT492035.1      ATTTTATATAAATTTTCTCTAGCTATAATTATTAAAATACAAATTCAAATTCTCAATAAAATTAAACCATATGATAATATATCACAAGAGTATATATATC
                  M  K  Y  L  N  E  S  A  M  I  M  L  I  C  I  W  I  S  L  L  I  L  G  Y  S  L  M  D  C  S  Y  M  Y  S
```

```
                TAAAATATATTTAAAAGAGATCGATATTAATAATTTTATGTTTAAGTTTAAGAGTTATTTTAATTTGGTATACTATTATATAGTGTTCTCATATATATAG

            9601         .         .         .         .         :         .         .         .         .         7 9700
1 MN793322.1    TTAAATTACAATAATTTTCAATATTAACCATTAAATTTATAAACATAAACATTATTAAAAATAATATTATTTGAACCATTCAATACATATTAACATTAAA
2 MT492036.1    TTAAATTACAATAATTTTCAATATTAACCATTAAATTTATAAACATAAACATTATTAAAAATAATATTATTTGAACCATTCAATACATATTAACATTAAA
3 MT492037.1    TTAAATTACAATAATTTTCAATATTAACCATTAAATTTATAAACATAAACATTATTAAAAATAATATTATTTGAACCATTCAATACATATTAACATTAAA
4 MT492030.1    TTAAATTACAATAATTTTCAATATTAACCATTAAATTTATAAACATAAACATTATTAAAAATAATATTATTTGAACCATTCAATACATATTAACATTAAA
5 MT492031.1    TTAAATTACAATAATTTTCAATATTAACCATTAAATTTATAAACATAAACATTATTAAAAATAATATTATTTGAACCATTCAATACATATTAACATTAAA
6 MT492032.1    TTAAATTACAATAATTTTCAATATTAACCATTAAATTTATAAACATAAACATTATTAAAAATAATATTATTTGAACCATTCAATACATATTAACATTAAA
7 MT492033.1    TTAAATTACAATAATTTTCAATATTAACCATTAAATTTATAAACATAAACATTATTAAAAATAATATTATTTGAACCATTCAATACATATTAACATTAAA
8 MT492034.1    TTAAATTACAATAATTTTCAATATTAACCATTAAATTTATAAACATAAACATTATTAAAAATAATATTATTTGAACCATTCAATACATATTAACATTAAA
9 MT492035.1    TTAAATTACAATAATTTTCAATATTAACCATTAAATTTATAAACATAAACATTATTAAAAATAATATTATTTGAACCATTCAATACATATTAACATTAAA
                 L  N  C  Y  N  E  I  N  V  M  L  N  M  F  M  F  M  M  L  F  L  M  M  Q  V  M  W  Y  M  N  V  N  F
                AATTTAATGTTATTAAAAGTTATAATTGGTAATTTAAATATTTGTATTTGTAATAATTTTTATTATATAATAAACTTGGTAAGTTATGTATAATTGTAATTT

            9701         .         .         .         .         :         .         .         .         .         8 9800
1 MN793322.1    ACATAAAGGAATTATAAAAATTATTATAAATAAAAATTTTATCATTTTTAAATTAAACTATATTAAAACTTTGAAAATAATCATTACCATGAGTACGGAT
2 MT492036.1    ACATAAAGGAATTATAAAAATTATTATAAATAAAAATTTTATCATTTTTAAATTAAACTATATTAAAACTTTGAAAATAATCATTACCATGAGTACGGAT
3 MT492037.1    ACATAAAGGAATTATAAAAATTATTATAAATAAAAATTTTATCATTTTTAAATTAAACTATATTAAAACTTTGAAAATAATCATTACCATGAGTACGGAT
4 MT492030.1    ACATAAAGGAATTATAAAAATTATTATAAATAAAAATTTTATCATTTTTAAATTAAACTATATTAAAACTTTGAAAATAATCATTACCATGAGTACGGAT
5 MT492031.1    ACATAAAGGAATTATAAAAATTATTATAAATAAAAATTTTATCATTTTTAAATTAAACTATATTAAAACTTTGAAAATAATCATTACCATGAGTACGGAT
6 MT492032.1    ACATAAAGGAATTATAAAAATTATTATAAATAAAAATTTTATCATTTTTAAATTAAACTATATTAAAACTTTGAAAATAATCATTACCATGAGTACGGAT
7 MT492033.1    ACATAAAGGAATTATAAAAATTATTATAAATAAAAATTTTATCATTTTTAAATTAAACTATATTAAAACTTTGAAAATAATCATTACCATGAGTACGGAT
8 MT492034.1    ACATAAAGGAATTATAAAAATTATTATAAATAAAAATTTTATCATTTTTAAATTAAACTATATTAAAACTTTGAAAATAATCATTACCATGAGTACGGAT
9 MT492035.1    ACATAAAGGAATTATAAAAATTATTATAAATAAAAATTTTATCATTTTTAAATTAAACTATATTAAAACTTTGAAAATAATCATTACCATGAGTACGGAT
                 C  L  P  I  M  F  I  M  M  F  L  F  K  M  M        *  V  M  N  F  S  Q  F  Y  D  N  G  H  T  R  I
                TGTATTTCCTTAATATTTTTAATAATATTTATTTTTAAAATAGTA       AATTTGATATAATTTTGAAACTTTATTAGTAATGGTACTCATGCCTA
                                                     <- ND4 |

            9801         .         .         .         .         :         .         .         .         .         9 9900
1 MN793322.1    TATTGAAACTAAAATAGATAGTCCTAAAGCACCCTCACAAACAGAAAAAACTAAAAAAACTATTAACATATATAAATCATATTCAATATAATTTAACATA
2 MT492036.1    TATTGAAACTAAAATAGATAGTCCTAAAGCACCCTCACAAACAGAAAAAACTAAAAAAACTATTAACATATATAAATCATATTCAATATAATTTAACATA
3 MT492037.1    TATTGAAACTAAAATAGATAGTCCTAAAGCACCCTCACAAACAGAAAAAACTAAAAAAACTATTAACATATATAAATCATATTCAATATAATTTAACATA
4 MT492030.1    TATTGAAACTAAAATAGATAGTCCTAAAGCACCCTCACAAACAGAAAAAACTAAAAAAACTATTAACATATATAAATCATATTCAATATAATTTAACATA
5 MT492031.1    TATTGAAACTAAAATAGATAGTCCTAAAGCACCCTCACAAACAGAAAAAACTAAAAAAACTATTAACATATATAAATCATATTCAATATAATTTAACATA
6 MT492032.1    TATTGAAACTAAAATAGATAGTCCTAAAGCACCCTCACAAACAGAAAAAACTAAAAAAACTATTAACATATATAAATCATATTCAATATAATTTAACATA
7 MT492033.1    TATTGAAACTAAAATAGATAGTCCTAAAGCACCCTCACAAACAGAAAAAACTAAAAAAACTATTAACATATATAAATCATATTCAATATAATTTAACATA
8 MT492034.1    TATTGAAACTAAAATAGATAGTCCTAAAGCACCCTCACAAACAGAAAAAACTAAAAAAACTATTAACATATATAAATCATATTCAATATAATTTAACATA
9 MT492035.1    TATTGAAACTAAAATAGATAGTCCTAAAGCACCCTCACAAACAGAAAAAACTAAAAAAACTATTAACATATATAAATCATATTCAATATAATTTAACATA
                 M  S  V  L  I  S  L  G  L  A  G  E  C  V  S  F  V  L  F  V  M  L  M  Y  L  D  Y  E  I  Y  N  L  M
                ATAACTTTGATTTTATCTATCAGGATTTCGTGGGAGTGTTTGTCTTTTTTGATTTTTTTGATAATTGTATATATTTAGTATAAGTTATATTAAATTGTAT

            9901         .         .         .         .         :         .         .         .         .         0 10000
1 MN793322.1    AAAATAAACAAAAAAAAAATTCTTAAAACAATAAATTCTAATCTTAATAAAACAATTAATAAATGTTTATGTTTAAAAACAAAAATTATATTCCCAAAAA
2 MT492036.1    AAAATAAACAAAAAAAAAATTCTTAAAACAATAAATTCTAATCTTAATAAAACAATTAATAAATGTTTATGTTTAAAAACAAAAATTATATTCCCAAAAA
3 MT492037.1    AAAATAAACAAAAAAAAAATTCTTAAAACAATAAATTCTAATCTTAATAAAACAATTAATAAATGTTTATGTTTAAAAACAAAAATTATATTCCCAAAAA
4 MT492030.1    AAAATAAACAAAAAAAAAATTCTTAAAACAATAAATTCTAATCTTAATAAAACAATTAATAAATGTTTATGTTTAAAAACAAAAATTATATTCCCAAAAA
5 MT492031.1    AAAATAAACAAAAAAAAAATTCTTAAAACAATAAATTCTAATCTTAATAAAACAATTAATAAATGTTTATGTTTAAAAACAAAAATTATATTCCCAAAAA
6 MT492032.1    AAAATAAACAAAAAAAAAATTCTTAAAACAATAAATTCTAATCTTAATAAAACAATTAATAAATGTTTATGTTTAAAAACAAAAATTATATTCCCAAAAA
7 MT492033.1    AAAATAAACAAAAAAAAAATTCTTAAAACAATAAATTCTAATCTTAATAAAACAATTAATAAATGTTTATGTTTAAAAACAAAAATTATATTCCCAAAAA
8 MT492034.1    AAAATAAACAAAAAAAAAATTCTTAAAACAATAAATTCTAATCTTAATAAAACAATTAATAAATGTTTATGTTTAAAAACAAAAATTATATTCCCAAAAA
9 MT492035.1    AAAATAAACAAAAAAAAAATTCTTAAAACAATAAATTCTAATCTTAATAAAACAATTAATAAATGTTTATGTTTAAAAACAAAAATTATATTCCCAAAAA
                F  I  F  L  F  F  I  S  L  V  I  F  E  L  S  L  L  V  I  L  L  H  K  H  K  F  V  F  I  M  N  G  F  I
                TTTTATTTGTTTTTTTTTTAAGAATTTTGTTATTTAAGATTAGAATTATTTTGTTAATTATTTACAAATACAAATTTTTGTTTTTAATATAAGGGTTTTT
```

```
                                                               | tRNA-Thr (UGU) ->
        10001         .         .         .         .         :         .         .         .         .         1 10100
                                                             gtttttataatttaaattaaaaatattggtcttgtaaaccaaaaa
1 MN793322.1     TAAATATAATAATAAAAATAATTCTTATATATAAAAAAATTATCATTAGTAAATTGTTTTTATAATTTAAATTAAAAATATTGGTCTTGTAAACCAAAAA
2 MT492036.1     TAAATATAATAATAAAAATAATTCTTATATATAAAAAAATTATCATTAGTAAATTGTTTTTATAATTTAAATTAAAAATATTGGTCTTGTAAACCAAAAA
3 MT492037.1     TAAATATAATAATAAAAATAATTCTTATATATAAAAAAATTATCATTAGTAAATTGTTTTTATAATTTAAATTAAAAATATTGGTCTTGTAAACCAAAAA
4 MT492030.1     TAAATATAATAATAAAAATAATTCTTATATATAAAAAAATTATCATTAGTAAATTGTTTTTATAATTTAAATTAAAAATATTGGTCTTGTAAACCAAAAA
5 MT492031.1     TAAATATAATAATAAAAATAATTCTTATATATAAAAAAATTATCATTAGTAAATTGTTTTTATAATTTAAATTAAAAATATTGGTCTTGTAAACCAAAAA
6 MT492032.1     TAAATATAATAATAAAAATAATTCTTATATATAAAAAAATTATCATTAGTAAATTGTTTTTATAATTTAAATTAAAAATATTGGTCTTGTAAACCAAAAA
7 MT492033.1     TAAATATAATAATAAAAATAATTCTTATATATAAAAAAATTATCATTAGTAAATTGTTTTTATAATTTAAATTAAAAATATTGGTCTTGTAAACCAAAAA
8 MT492034.1     TAAATATAATAATAAAAATAATTCTTATATATAAAAAAATTATCATTAGTAAATTGTTTTTATAATTTAAATTAAAAATATTGGTCTTGTAAACCAAAAA
9 MT492035.1     TAAATATAATAATAAAAATAATTCTTATATATAAAAAAATTATCATTAGTAAATTGTTTTTATAATTTAAATTAAAAATATTGGTCTTGTAAACCAAAAA
                   F  M  I  I  F  I  I  S  M  Y  L  F  I  M  M
                 ATTTATATTATTATTTTTATTAAGAATATATATTTTTTTAATAGTA
                                                     <- ND4L |

                                                                                                       | ND6 ->
         10101         .         .         .         .         :         .         .         .         .         2 10200
                 taagattatttctttaaaaact                                                                  M  M  K  V
1 MN793322.1     TAAGATTATTTCTTTAAAAACTTCAAAGAAAAAGAAAATCTTTATCAATAATCTCCAAAATTATTATTTTTATTAAACTACTCTTTGAAATTATAAAAGT
2 MT492036.1     TAAGATTATTTCTTTAAAAACTTCAAAGAAAAAGAAAATCTTTATCAATAATCTCCAAAATTATTATTTTTATTAAACTACTCTTTGAAATTATAAAAGT
3 MT492037.1     TAAGATTATTTCTTTAAAAACTTCAAAGAAAAAGAAAATCTTTATCAATAATCTCCAAAATTATTATTTTTATTAAACTACTCTTTGAAATTATAAAAGT
4 MT492030.1     TAAGATTATTTCTTTAAAAACTTCAAAGAAAAAGAAAATCTTTATCAATAATCTCCAAAATTATTATTTTTATTAAACTACTCTTTGAAATTATAAAAGT
5 MT492031.1     TAAGATTATTTCTTTAAAAACTTCAAAGAAAAAGAAAATCTTTATCAATAATCTCCAAAATTATTATTTTTATTAAACTACTCTTTGAAATTATAAAAGT
6 MT492032.1     TAAGATTATTTCTTTAAAAACTTCAAAGAAAAAGAAAATCTTTATCAATAATCTCCAAAATTATTATTTTTATTAAACTACTCTTTGAAATTATAAAAGT
7 MT492033.1     TAAGATTATTTCTTTAAAAACTTCAAAGAAAAAGAAAATCTTTATCAATAATCTCCAAAATTATTATTTTTATTAAACTACTCTTTGAAATTATAAAAGT
8 MT492034.1     TAAGATTATTTCTTTAAAAACTTCAAAGAAAAAGAAAATCTTTATCAATAATCTCCAAAATTATTATTTTTATTAAACTACTCTTTGAAATTATAAAAGT
9 MT492035.1     TAAGATTATTTCTTTAAAAACTTCAAAGAAAAAGAAAATCTTTATCAATAATCTCCAAAATTATTATTTTTATTAAACTACTCTTTGAAATTATAAAAGT
                                      agtttctttttcttttagaaatagttattagaggttttaataataaaaataatttgatgagaaac
                                                                                   <- tRNA-Pro (UGG) |

         10201         .         .         .         .         :         .         .         .         .         3 10300
                   F  I  S  L  L  I  I  L  L  S  F  I  M  L  F  L  N  H  P  L  S  M  G  F  M  I  L  I  Q  T  L  L  T
1 MN793322.1     ATTTATTTCTTTATTAATTATTTTTATTATCATTTATTATATTATTCCTTAATCATCCATTATCAATAGGTTTTATAATTTTAATTCAAACATTATTAACA
2 MT492036.1     ATTTATTTCTTTATTAATTATTTTTATTATCATTTATTATATTATTCCTTAATCATCCATTATCAATAGGTTTTATAATTTTAATTCAAACATTATTAACA
3 MT492037.1     ATTTATTTCTTTATTAATTATTTTTATTATCATTTATTATATTATTCCTTAATCATCCATTATCAATAGGTTTTATAATTTTAATTCAAACATTATTAACA
4 MT492030.1     ATTTATTTCTTTATTAATTATTTTTATTATCATTTATTATATTATTCCTTAATCATCCATTATCAATAGGTTTTATAATTTTAATTCAAACATTATTAACA
5 MT492031.1     ATTTATTTCTTTATTAATTATTTTTATTATCATTTATTATATTATTCCTTAATCATCCATTATCAATAGGTTTTATAATTTTAATTCAAACATTATTAACA
6 MT492032.1     ATTTATTTCTTTATTAATTATTTTTATTATCATTTATTATATTATTCCTTAATCATCCATTATCAATAGGTTTTATAATTTTAATTCAAACATTATTAACA
7 MT492033.1     ATTTATTTCTTTATTAATTATTTTTATTATCATTTATTATATTATTCCTTAATCATCCATTATCAATAGGTTTTATAATTTTAATTCAAACATTATTAACA
8 MT492034.1     ATTTATTTCTTTATTAATTATTTTTATTATCATTTATTATATTATTCCTTAATCATCCATTATCAATAGGTTTTATAATTTTAATTCAAACATTATTAACA
9 MT492035.1     ATTTATTTCTTTATTAATTATTTTTATTATCATTTATTATATTATTCCTTAATCATCCATTATCAATAGGTTTTATAATTTTAATTCAAACATTATTAACA

         10301         .         .         .         .         :         .         .         .         .         4 10400
                  C  I  I  T  S  M  I  M  K  T  Y  W  M  S  Y  I  L  F  L  T  F  L  G  G  L  L  V  L  F  I  Y  V  S
1 MN793322.1     TGTATTATTACAAGAATGATTATAAAAACATATTGAATATCTTATATTTTATTTTTAACTTTCTTAGGAGGATTATTAGTTCTATTTATCTATGTAAGAA
2 MT492036.1     TGTATTATTACAAGAATGATTATAAAAACATATTGAATATCTTATATTTTATTTTTAACTTTCTTAGGAGGATTATTAGTTCTATTTATCTATGTAAGAA
3 MT492037.1     TGTATTATTACAAGAATGATTATAAAAACATATTGAATATCTTATATTTTATTTTTAACTTTCTTAGGAGGATTATTAGTTCTATTTATCTATGTAAGAA
4 MT492030.1     TGTATTATTACAAGAATGATTATAAAAACATATTGAATATCTTATATTTTATTTTTAACTTTCTTAGGAGGATTATTAGTTCTATTTATCTATGTAAGAA
5 MT492031.1     TGTATTATTACAAGAATGATTATAAAAACATATTGAATATCTTATATTTTATTTTTAACTTTCTTAGGAGGATTATTAGTTCTATTTATCTATGTAAGAA
6 MT492032.1     TGTATTATTACAAGAATGATTATAAAAACATATTGAATATCTTATATTTTATTTTTAACTTTCTTAGGAGGATTATTAGTTCTATTTATCTATGTAAGAA
7 MT492033.1     TGTATTATTACAAGAATGATTATAAAAACATATTGAATATCTTATATTTTATTTTTAACTTTCTTAGGAGGATTATTAGTTCTATTTATCTATGTAAGAA
8 MT492034.1     TGTATTATTACAAGAATGATTATAAAAACATATTGAATATCTTATATTTTATTTTTAACTTTCTTAGGAGGATTATTAGTTCTATTTATCTATGTAAGAA
9 MT492035.1     TGTATTATTACAAGAATGATTATAAAAACATATTGAATATCTTATATTTTATTTTTAACTTTCTTAGGAGGATTATTAGTTCTATTTATCTATGTAAGAA
```

```
          10401         .         .         .         .         :         .         .         .         .        5 10500
                 S  I  A  S  N  E  L  F  K  L  S  I  S  L  K  M  L  L  L  F  N  L  I  I  I  L  M  I  S  M  F  F  N  N
1 MN793322.1     GAATTGCATCAAATGAATTATTTAAATTATCAATTTCACTAAAAATATTATTATTATTTAATCTAATCATTATTTTAATAATTAGAATATTTTTTAATAA
2 MT492036.1     GAATTGCATCAAATGAATTATTTAAATTATCAATTTCACTAAAAATATTATTATTATTTAATCTAATCATTATTTTAATAATTAGAATATTTTTTAATAA
3 MT492037.1     GAATTGCATCAAATGAATTATTTAAATTATCAATTTCACTAAAAATATTATTATTATTTAATCTAATCATTATTTTAATAATTAGAATATTTTTTAATAA
4 MT492030.1     GAATTGCATCAAATGAATTATTTAAATTATCAATTTCACTAAAAATATTATTATTATTTAATCTAATCATTATTTTAATAATTAGAATATTTTTTAATAA
5 MT492031.1     GAATTGCATCAAATGAATTATTTAAATTATCAATTTCACTAAAAATATTATTATTATTTAATCTAATCATTATTTTAATAATTAGAATATTTTTTAATAA
6 MT492032.1     GAATTGCATCAAATGAATTATTTAAATTATCAATTTCACTAAAAATATTATTATTATTTAATCTAATCATTATTTTAATAATTAGAATATTTTTTAATAA
7 MT492033.1     GAATTGCATCAAATGAATTATTTAAATTATCAATTTCACTAAAAATATTATTATTATTTAATCTAATCATTATTTTAATAATTAGAATATTTTTTAATAA
8 MT492034.1     GAATTGCATCAAATGAATTATTTAAATTATCAATTTCACTAAAAATATTATTATTATTTAATCTAATCATTATTTTAATAATTAGAATATTTTTTAATAA
9 MT492035.1     GAATTGCATCAAATGAATTATTTAAATTATCAATTTCACTAAAAATATTATTATTATTTAATCTAATCATTATTTTAATAATTAGAATATTTTTTAATAA

          10501         .         .         .         .         :         .         .         .         .        6 10600
                   N  L  Y  W  I  N  L  S  I  N  N  L  E  M  N  N  F  F  N  M  F  L  F  F  N  N  E  N  K  I  N  L  S
1 MN793322.1     TAATTTATATTGAATTAATTTAAGAATCAATAATCTAGAAATAAATAATTTTTTTAATATATTTTTATTTTTTAATAACGAAAATAAAATTAACTTATCT
2 MT492036.1     TAATTTATATTGAATTAATTTAAGAATCAATAATCTAGAAATAAATAATTTTTTTAATATATTTTTATTTTTTAATAACGAAAATAAAATTAACTTATCT
3 MT492037.1     TAATTTATATTGAATTAATTTAAGAATCAATAATCTAGAAATAAATAATTTTTTTAATATATTTTTATTTTTTAATAACGAAAATAAAATTAACTTATCT
4 MT492030.1     TAATTTATATTGAATTAATTTAAGAATCAATAATCTAGAAATAAATAATTTTTTTAATATATTTTTATTTTTTAATAACGAAAATAAAATTAACTTATCT
5 MT492031.1     TAATTTATATTGAATTAATTTAAGAATCAATAATCTAGAAATAAATAATTTTTTTAATATATTTTTATTTTTTAATAACGAAAATAAAATTAACTTATCT
6 MT492032.1     TAATTTATATTGAATTAATTTAAGAATCAATAATCTAGAAATAAATAATTTTTTTAATATATTTTTATTTTTTAATAACGAAAATAAAATTAACTTATCT
7 MT492033.1     TAATTTATATTGAATTAATTTAAGAATCAATAATCTAGAAATAAATAATTTTTTTAATATATTTTTATTTTTTAATAACGAAAATAAAATTAACTTATCT
8 MT492034.1     TAATTTATATTGAATTAATTTAAGAATCAATAATCTAGAAATAAATAATTTTTTTAATATATTTTTATTTTTTAATAACGAAAATAAAATTAACTTATCT
9 MT492035.1     TAATTTATATTGAATTAATTTAAGAATCAATAATCTAGAAATAAATAATTTTTTTAATATATTTTTATTTTTTAATAACGAAAATAAAATTAACTTATCT

          10601         .         .         .         .         :         .         .         .         .        7 10700
                  K  L  Y  N  N  Q  T  F  L  M  M  M  M  M  I  I  Y  L  F  I  A  L  I  A  V  V  K  I  T  N  I  F  Y
1 MN793322.1     AAATTATATAATAATCAAACATTTTTAATAATAATAATAATAATTATCTATTTATTTATTGCCTTAATTGCAGTAGTAAAAATCACAAATATTTTTTATG
2 MT492036.1     AAATTATATAATAATCAAACATTTTTAATAATAATAATAATAATTATCTATTTATTTATTGCCTTAATTGCAGTAGTAAAAATCACAAATATTTTTTATG
3 MT492037.1     AAATTATATAATAATCAAACATTTTTAATAATAATAATAATAATTATCTATTTATTTATTGCCTTAATTGCAGTAGTAAAAATCACAAATATTTTTTATG
4 MT492030.1     AAATTATATAATAATCAAACATTTTTAATAATAATAATAATAATTATCTATTTATTTATTGCCTTAATTGCAGTAGTAAAAATCACAAATATTTTTTATG
5 MT492031.1     AAATTATATAATAATCAAACATTTTTAATAATAATAATAATAATTATCTATTTATTTATTGCCTTAATTGCAGTAGTAAAAATCACAAATATTTTTTATG
6 MT492032.1     AAATTATATAATAATCAAACATTTTTAATAATAATAATAATAATTATCTATTTATTTATTGCCTTAATTGCAGTAGTAAAAATCACAAATATTTTTTATG
7 MT492033.1     AAATTATATAATAATCAAACATTTTTAATAATAATAATAATAATTATCTATTTATTTATTGCCTTAATTGCAGTAGTAAAAATCACAAATATTTTTTATG
8 MT492034.1     AAATTATATAATAATCAAACATTTTTAATAATAATAATAATAATTATCTATTTATTTATTGCCTTAATTGCAGTAGTAAAAATCACAAATATTTTTTATG
9 MT492035.1     AAATTATATAATAATCAAACATTTTTAATAATAATAATAATAATTATCTATTTATTTATTGCCTTAATTGCAGTAGTAAAAATCACAAATATTTTTTATG

                                          CYTB ->
          10701         .         .         .         .         :         .         .         .         .        8 10800
                 G  P  L  R  S  S  F  N  *     M  N  I  Y  K  P  I  R  K  T  H  P  I  F  K  I  I  N  G  S  L  I  D  L
1 MN793322.1     GTCCTTTACGATCTTCATTTAACTAATGATAAACATTTATAAGCCAATTCGAAAAACCCATCCTATTTTCAAAATCATTAATGGATCATTAATTGATTTA
2 MT492036.1     GTCCTTTACGATCTTCATTTAACTAATGATAAACATTTATAAGCCAATTCGAAAAACCCATCCTATTTTCAAAATCATTAATGGATCATTAATTGATTTA
3 MT492037.1     GTCCTTTACGATCTTCATTTAACTAATGATAAACATTTATAAGCCAATTCGAAAAACCCATCCTATTTTCAAAATCATTAATGGATCATTAATTGATTTA
4 MT492030.1     GTCCTTTACGATCTTCATTTAACTAATGATAAACATTTATAAGCCAATTCGAAAAACCCATCCTATTTTCAAAATCATTAATGGATCATTAATTGATTTA
5 MT492031.1     GTCCTTTACGATCTTCATTTAACTAATGATAAACATTTATAAGCCAATTCGAAAAACCCATCCTATTTTCAAAATCATTAATGGATCATTAATTGATTTA
6 MT492032.1     GTCCTTTACGATCTTCATTTAACTAATGATAAACATTTATAAGCCAATTCGAAAAACCCATCCTATTTTCAAAATCATTAATGGATCATTAATTGATTTA
7 MT492033.1     GTCCTTTACGATCTTCATTTAACTAATGATAAACATTTATAAGCCAATTCGAAAAACCCATCCTATTTTCAAAATCATTAATGGATCATTAATTGATTTA
8 MT492034.1     GTCCTTTACGATCTTCATTTAACTAATGATAAACATTTATAAGCCAATTCGAAAAACCCATCCTATTTTCAAAATCATTAATGGATCATTAATTGATTTA
9 MT492035.1     GTCCTTTACGATCTTCATTTAACTAATGATAAACATTTATAAGCCAATTCGAAAAACCCATCCTATTTTCAAAATCATTAATGGATCATTAATTGATTTA
```

```
          10801     .    .    .    .    :    .    .    .    .    9 10900
               P  T  P  S  N  I  S  S  L  W  N  F  G  S  L  L  A  M  C  L  I  I  Q  I  I  T  G  L  F  L  T  M  Y
1 MN793322.1     CCAACTCCATCAAATATTTCTTCCCTATGAAATTTTGGATCCTTATTAGCTATATGTTTAATTATTCAAATTATTACTGGACTATTTTTAACAATATATT
2 MT492036.1     CCAACTCCCTCAAATATTTCTTCCCTATGAAATTTTGGATCCTTATTAGCTATATGTTTAATTATTCAAATTATTACTGGACTATTTTTAACAATATATT
3 MT492037.1     CCAACTCCATCAAATATTTCTTCCCTATGAAATTTTGGATCCTTATTAGCTATATGTTTAATTATTCAAATTATTACTGGACTATTTTTAACAATATATT
4 MT492030.1     CCAACTCCCTCAAATATTTCTTCCCTATGAAATTTTGGATCCTTATTAGCTATATGTTTAATTATTCAAATTATTACTGGACTATTTTTAACAATATATT
5 MT492031.1     CCAACTCCCTCAAATATTTCTTCCCTATGAAATTTTGGATCCTTATTAGCTATATGTTTAATTATTCAAATTATTACTGGACTATTTTTAACAATATATT
6 MT492032.1     CCGACTCCCTCAAATATTTCTTCCCTATGAAATTTTGGATCCTTATTAGCTATATGTTTAATTATTCAAATTATTACTGGACTATTTTTAACCATATATT
7 MT492033.1     CCAACTCCATCAAATATTTCTTCCCTATGAAATTTTGGATCCTTATTAGCTATATGTTTAATTATTCAAATTATTACTGGACTATTTTTAACAATATATT
8 MT492034.1     CCAACTCCATCAAATATTTCTTCCCTATGAAATTTTGGATCCTTATTAGCTATATGTTTAATTATTCAAATTATTACTGGACTATTTTTAACAATATATT
9 MT492035.1     CCAACTCCATCAAATATTTCTTCCCTATGAAATTTTGGATCCTTATTAGCTATATGTTTAATTATTCAAATTATTACTGGACTATTTTTAACCATATATT

          10901     .    .    .    .    :    .    .    .    .    0 11000
               Y  T  A  N  I  E  L  A  F  Y  S  V  N  Y  I  C  R  N  V  N  Y  G  W  L  I  R  T  L  H  A  N  G  A  S
1 MN793322.1     ATACAGCTAATATTGAATTAGCTTTTTATAGAGTTAACTATATTTGTCGAAATGTAAATTATGGTTGATTAATTCGAACTCTTCATGCAAATGGAGCTTC
2 MT492036.1     ATACAGCTAATATTGAATTAGCTTTTTATAGAGTTAACTATATTTGTCGAAATGTAAATTATGGTTGATTAATTCGAACTCTTCATGCAAATGGAGCTTC
3 MT492037.1     ATACAGCTAATATTGAATTAGCTTTTTATAGAGTTAACTATATTTGTCGAAATGTAAATTATGGTTGATTAATTCGAACTCTTCATGCAAATGGAGCTTC
4 MT492030.1     ATACAGCTAATATTGAATTAGCTTTTTATAGAGTTAACTATATTTGTCGAAATGTAAATTATGGTTGATTAATTCGAACTCTTCATGCAAATGGAGCTTC
5 MT492031.1     ATACAGCTAATATTGAATTAGCTTTTTATAGAGTTAACTATATTTGTCGAAATGTAAATTATGGTTGATTAATTCGAACTCTTCATGCAAATGGAGCTTC
6 MT492032.1     ATACAGCTAATATTGAATTAGCTTTTTATAGAGTTAACTATATTTGTCGAAATGTAAATTATGGTTGATTAATTCGAACTCTTCATGCAAATGGAGCTTC
7 MT492033.1     ATACAGCTAATATTGAATTAGCTTTTTATAGAGTTAACTATATTTGTCGAAATGTAAATTATGGTTGATTAATTCGAACTCTTCATGCAAATGGAGCTTC
8 MT492034.1     ATACAGCTAATATTGAATTAGCTTTTTATAGAGTTAACTATATTTGTCGAAATGTAAATTATGGTTGATTAATTCGAACTCTTCATGCAAATGGAGCTTC
9 MT492035.1     ATACAGCTAATATTGAATTAGCTTTTTATAGAGTTAACTATATTTGTCGAAATGTAAATTATGGTTGATTAATTCGAACTCTTCATGCAAATGGAGCTTC

          11001     .    .    .    .    :    .    .    .    .    1 11100
                 F  F  F  I  C  I  Y  I  H  I  G  R  G  I  Y  Y  E  S  F  N  L  K  Y  T  W  M  V  G  V  I  I  L  F
1 MN793322.1     ATTTTTTTTTATTTGTATTTATATTCACATTGGACGAGGAATTTATTATGAATCATTTAATTTAAAATATACATGAATAGTTGGAGTAATTATTTTATTC
2 MT492036.1     ATTTTTTTTTATTTGTATTTATATTCACATTGGACGAGGAATTTATTATGAATCATTTAATTTAAAATATACATGAATAGTTGGAGTAATTATTTTATTC
3 MT492037.1     ATTTTTTTTTATTTGTATTTATATTCACATTGGACGAGGAATTTATTATGAATCATTTAATTTAAAATATACATGAATAGTTGGAGTAATTATTTTATTC
4 MT492030.1     ATTTTTTTTTATTTGTATTTATATTCACATTGGACGAGGAATTTATTATGAATCATTTAATTTAAAATATACATGAATAGTTGGAGTAATTATTTTATTC
5 MT492031.1     ATTTTTTTTTATTTGTATTTATATTCACATTGGACGAGGAATTTATTATGAATCATTTAATTTAAAATATACATGAATAGTTGGAGTAATTATTTTATTC
6 MT492032.1     ATTTTTTTTTATTTGTATTTATATTCACATTGGACGAGGAATTTATTATGAATCATTTAATTTAAAATATACATGAATAGTTGGAGTAATTATTTTATTC
7 MT492033.1     ATTTTTTTTTATTTGTATTTATATTCACATTGGACGAGGAATTTATTATGAATCATTTAATTTAAAATATACATGAATAGTTGGAGTAATTATTTTATTC
8 MT492034.1     ATTTTTTTTTATTTGTATTTATATTCACATTGGACGAGGAATTTATTATGAATCATTTAATTTAAAATATACATGAATAGTTGGAGTAATTATTTTATTC
9 MT492035.1     ATTTTTTTTTATTTGTATTTATATTCACATTGGACGAGGAATTTATTATGAATCATTTAATTTAAAATATACATGAATAGTTGGAGTAATTATTTTATTC

          11101     .    .    .    .    :    .    .    .    .    2 11200
                L  L  M  A  T  A  F  M  G  Y  V  L  P  W  G  Q  M  S  F  W  G  A  T  V  I  T  N  L  L  S  A  I  P
1 MN793322.1     TTATTAATAGCAACAGCTTTTATAGGATATGTATTACCTTGAGGACAAATATCATTTTGAGGAGCAACAGTTATTACAAATCTACTATCAGCAATTCCTT
2 MT492036.1     TTATTAATAGCAACAGCTTTTATAGGATATGTATTACCTTGAGGACAAATATCATTTTGAGGAGCAACAGTTATTACAAATCTACTATCAGCAATTCCTT
3 MT492037.1     TTATTAATAGCAACAGCTTTTATAGGATATGTATTACCTTGAGGACAAATATCATTTTGAGGAGCAACAGTTATTACAAATCTACTATCAGCAATTCCTT
4 MT492030.1     TTATTAATAGCAACAGCTTTTATAGGATATGTATTACCTTGAGGACAAATATCATTTTGAGGAGCAACAGTTATTACAAATCTACTATCAGCAATTCCTT
5 MT492031.1     TTATTAATAGCAACAGCTTTTATAGGATATGTATTACCTTGAGGACAAATATCATTTTGAGGAGCAACAGTTATTACAAATCTACTATCAGCAATTCCTT
6 MT492032.1     TTATTAATAGCAACAGCTTTTATAGGATATGTATTACCTTGAGGACAAATATCATTTTGAGGAGCAACAGTTATTACAAATCTACTATCAGCAATTCCTT
7 MT492033.1     TTATTAATAGCAACAGCTTTTATAGGATATGTATTACCTTGAGGACAAATATCATTTTGAGGAGCAACAGTTATTACAAATCTACTATCAGCAATTCCTT
8 MT492034.1     TTATTAATAGCAACAGCTTTTATAGGATATGTATTACCTTGAGGACAAATATCATTTTGAGGAGCAACAGTTATTACAAATCTACTATCAGCAATTCCTT
9 MT492035.1     TTATTAATAGCAACAGCTTTTATAGGATATGTATTACCTTGAGGACAAATATCATTTTGAGGAGCAACAGTTATTACAAATCTACTATCAGCAATTCCTT
```

```
          11201          .         .         .         .         :         .         .         .         .         3 11300
                 Y  L  G  T  M  L  V  N  W  I  W  G  G  F  A  I  D  N  A  T  L  T  R  F  Y  T  F  H  F  I  L  P  F  I
1 MN793322.1     ATTTAGGAACTATATTAGTAAATTGAATTTGAGGAGGATTTGCCATTGATAATGCAACATTAACTCGATTTTATACATTTCATTTTATTCTACCATTTAT
2 MT492036.1     ATTTAGGAACTATATTAGTAAATTGAATTTGAGGAGGATTTGCCATTGATAATGCAACATTAACTCGATTTTATACATTTCATTTTATTCTACCATTTAT
3 MT492037.1     ATTTAGGAACTATATTAGTAAATTGAATTTGAGGAGGATTTGCCATTGATAATGCAACATTAACTCGATTTTATACATTTCATTTTATTCTACCATTTAT
4 MT492030.1     ATTTAGGAACTATATTAGTAAATTGAATTTGAGGAGGATTTGCCATTGATAATGCAACATTAACTCGATTTTATACATTTCATTTTATTCTACCATTTAT
5 MT492031.1     ATTTAGGAACTATATTAGTAAATTGAATTTGAGGAGGATTTGCCATTGATAATGCAACATTAACTCGATTTTATACATTTCATTTTATTCTACCATTTAT
6 MT492032.1     ATTTAGGAACTATATTAGTAAATTGAATTTGAGGAGGATTTGCCATTGATAATGCAACATTAACTCGATTTTATACATTTCATTTTATTCTACCATTTAT
7 MT492033.1     ATTTAGGAACTATATTAGTAAATTGAATTTGAGGAGGATTTGCCATTGATAATGCAACATTAACTCGATTTTATACATTTCATTTTATTCTACCATTTAT
8 MT492034.1     ATTTAGGAACTATATTAGTAAATTGAATTTGAGGAGGATTTGCCATTGATAATGCAACATTAACTCGATTTTATACATTTCATTTTATTCTACCATTTAT
9 MT492035.1     ATTTAGGAACTATATTAGTAAATTGAATTTGAGGAGGATTTGCCATTGATAATGCAACATTAACTCGATTTTATACATTTCATTTTATTCTACCATTTAT

          11301          .         .         .         .         :         .         .         .         .         4 11400
                   I  L  M  M  T  M  I  H  L  L  F  L  H  Q  T  G  S  N  N  P  L  G  I  N  S  N  L  D  K  I  P  F  H
1 MN793322.1     TATTTTAATAATAACAATAATTCATCTATTATTTTTACATCAAACAGGATCTAATAACCCTTTAGGAATTAATAGAAACTTAGATAAAATTCCATTTCAC
2 MT492036.1     TATTTTAATAATAACAATAATTCATCTATTATTTTTACATCAAACAGGATCTAATAACCCTTTAGGAATTAATAGAAACTTAGATAAAATTCCATTTCAC
3 MT492037.1     TATTTTAATAATAACAATAATTCATCTATTATTTTTACATCAAACAGGATCTAATAACCCTTTAGGAATTAATAGAAACTTAGATAAAATTCCATTTCAC
4 MT492030.1     TATTTTAATAATAACAATAATTCATCTATTATTTTTACATCAAACAGGATCTAATAACCCTTTAGGAATTAATAGAAACTTAGATAAAATTCCATTTCAC
5 MT492031.1     TATTTTAATAATAACAATAATTCATCTATTATTTTTACATCAAACAGGATCTAATAACCCTTTAGGAATTAATAGAAACTTAGATAAAATTCCATTTCAC
6 MT492032.1     TATTTTAATAATAACAATAATTCATCTATTATTTTTACATCAAACAGGATCTAATAACCCTTTAGGAATTAATAGAAACTTAGATAAAATTCCATTTCAC
7 MT492033.1     TATTTTAATAATAACAATAATTCATCTATTATTTTTACATCAAACAGGATCTAATAACCCTTTAGGAATTAATAGAAACTTAGATAAAATTCCATTTCAC
8 MT492034.1     TATTTTAATAATAACAATAATTCATCTATTATTTTTACATCAAACAGGATCTAATAACCCTTTAGGAATTAATAGAAACTTAGATAAAATTCCATTTCAC
9 MT492035.1     TATTTTAATAATAACAATAATTCATCTATTATTTTTACATCAAACAGGATCTAATAACCCTTTAGGAATTAATAGAAACTTAGATAAAATTCCATTTCAC

          11401          .         .         .         .         :         .         .         .         .         5 11500
                  P  F  F  T  F  K  D  M  I  G  F  I  I  I  S  F  L  L  I  F  L  T  L  T  N  P  Y  L  L  G  D  P  D
1 MN793322.1     CCATTTTTTACATTTAAAGATATAATTGGATTTATTATTATTTCATTTTTATTAATTTTTTTAACATTAACCAATCCTTATTTATTAGGAGACCCTGATA
2 MT492036.1     CCATTTTTTACATTTAAAGATATAATTGGATTTATTATTATTTCATTTTTATTAATTTTTTTAACATTAACCAATCCTTATTTATTAGGAGACCCTGATA
3 MT492037.1     CCATTTTTTACATTTAAAGATATAATTGGATTTATTATTATTTCATTTTTATTAATTTTTTTAACATTAACCAATCCTTATTTATTAGGAGACCCTGATA
4 MT492030.1     CCATTTTTTACATTTAAAGATATAATTGGATTTATTATTATTTCATTTTTATTAATTTTTTTAACATTAACCAATCCTTATTTATTAGGAGACCCTGATA
5 MT492031.1     CCATTTTTTACATTTAAAGATATAATTGGATTTATTATTATTTCATTTTTATTAATTTTTTTAACATTAACCAATCCTTATTTATTAGGAGACCCTGATA
6 MT492032.1     CCATTTTTTACATTTAAAGATATAATTGGATTTATTATTATTTCATTTTTATTAATTTTTTTAACATTAACCAATCCTTATTTATTAGGAGACCCTGATA
7 MT492033.1     CCATTTTTTACATTTAAAGATATAATTGGATTTATTATTATTTCATTTTTATTAATTTTTTTAACATTAACCAATCCTTATTTATTAGGAGACCCTGATA
8 MT492034.1     CCATTTTTTACATTTAAAGATATAATTGGATTTATTATTATTTCATTTTTATTAATTTTTTTAACATTAACCAATCCTTATTTATTAGGAGACCCTGATA
9 MT492035.1     CCATTTTTTACATTTAAAGATATAATTGGATTTATTATTATTTCATTTTTATTAATTTTTTTAACATTAACCAATCCTTATTTATTAGGAGACCCTGATA

          11501          .         .         .         .         :         .         .         .         .         6 11600
                 N  F  T  P  A  N  P  L  V  T  P  I  H  I  Q  P  E  W  Y  F  L  F  A  Y  A  I  L  R  S  I  P  N  K  L
1 MN793322.1     ATTTTACACCAGCTAATCCATTAGTAACACCAATTCATATTCAACCTGAATGATATTTCTTATTTGCTTATGCAATTTTACGATCAATTCCAAATAAATT
2 MT492036.1     ATTTTACACCAGCTAATCCATTAGTAACACCAATTCATATTCAACCTGAATGATATTTCTTATTTGCTTATGCAATTTTACGATCAATTCCAAATAAATT
3 MT492037.1     ATTTTACACCAGCTAATCCATTAGTAACACCAATTCATATTCAACCTGAATGATATTTCTTATTTGCTTATGCAATTTTACGATCAATTCCAAATAAATT
4 MT492030.1     ATTTTACACCAGCTAATCCATTAGTAACACCAATTCATATTCAACCTGAATGATATTTCTTATTTGCTTATGCAATTTTACGATCAATTCCAAATAAATT
5 MT492031.1     ATTTTACACCAGCTAATCCATTAGTAACACCAATTCATATTCAACCTGAATGATATTTCTTATTTGCTTATGCAATTTTACGATCAATTCCAAATAAATT
6 MT492032.1     ATTTTACACCAGCTAATCCATTAGTAACACCAATTCATATTCAACCTGAATGATATTTCTTATTTGCTTATGCAATTTTACGATCAATTCCAAATAAATT
7 MT492033.1     ATTTTACACCAGCTAATCCATTAGTAACACCAATTCATATTCAACCTGAATGATATTTCTTATTTGCTTATGCAATTTTACGATCAATTCCAAATAAATT
8 MT492034.1     ATTTTACACCAGCTAATCCATTAGTAACACCAATTCATATTCAACCTGAATGATATTTCTTATTTGCTTATGCAATTTTACGATCAATTCCAAATAAATT
9 MT492035.1     ATTTTACACCAGCTAATCCATTAGTAACACCAATTCATATTCAACCTGAATGATATTTCTTATTTGCTTATGCAATTTTACGATCAATTCCAAATAAATT
```

```
            11601        .         .         .         .         :         .         .         .         .     7 11700
                      G  G  V  I  A  L  I  M  S  I  L  I  L  I  I  L  P  L  T  F  F  K  K  I  Q  G  I  Q  F  Y  P  M  N
1 MN793322.1     AGGAGGTGTTATTGCACTTATTATATCAATTTTAATTTTAATTATTTTACCATTAACATTCTTTAAAAAAATTCAAGGAATTCAATTTTATCCAATAAAC
2 MT492036.1     AGGAGGTGTTATTGCACTTATTATATCAATTTTAATTTTAATTATTTTACCATTAACATTCTTTAAAAAAATTCAAGGAATTCAATTTTATCCAATAAAC
3 MT492037.1     AGGAGGTGTTATTGCACTTATTATATCAATTTTAATTTTAATTATTTTACCATTAACATTCTTTAAAAAAATTCAAGGAATTCAATTTTATCCAATAAAC
4 MT492030.1     AGGAGGTGTTATTGCACTTATTATATCAATTTTAATTTTAATTATTTTACCATTAACATTCTTTAAAAAAATTCAAGGAATTCAATTTTATCCAATAAAC
5 MT492031.1     AGGAGGTGTTATTGCACTTATTATATCAATTTTAATTTTAATTATTTTACCATTAACATTCTTTAAAAAAATTCAAGGAATTCAATTTTATCCAATAAAC
6 MT492032.1     AGGAGGGGTTATTGCACTTATTATATCAATTTTAATTTTAATTATTTTACCATTAACATTCTTTAAAAAAATTCAAGGAATTCAATTTTATCCAATAAAC
7 MT492033.1     AGGAGGGGTTATTGCACTTATTATATCAATTTTAATTTTAATTATTTTACCATTAACATTCTTTAAAAAAATTCAAGGAATTCAATTTTATCCAATAAAC
8 MT492034.1     AGGAGGTGTTATTGCACTTATTATATCAATTTTAATTTTAATTATTTTACCATTAACATTCTTTAAAAAAATTCAAGGAATTCAATTTTATCCAATAAAC
9 MT492035.1     AGGAGGGGTTATTGCACTTATTATATCAATTTTAATTTTAATTATTTTACCATTAACATTCTTTAAAAAAATTCAAGGAATTCAATTTTATCCAATAAAC

            11701        .         .         .         .         :         .         .         .         .     8 11800
                   Q  I  L  F  W  I  M  V  V  T  I  I  L  L  T  W  I  G  A  R  P  V  E  D  P  Y  V  F  V  G  Q  V  L
1 MN793322.1     CAAATCTTATTTTGAATTATAGTAGTTACAATTATTTTACTAACATGAATTGGAGCACGACCAGTAGAAGATCCTTATGTATTTGTGGGACAAGTATTAA
2 MT492036.1     CAAATCTTATTTTGAATTATAGTAGTTACAATTATTTTACTAACATGAATTGGAGCACGACCAGTAGAAGATCCTTATGTATTTGTGGGACAAGTATTAA
3 MT492037.1     CAAATCTTATTTTGAATTATAGTAGTTACAATTATTTTACTAACATGAATTGGAGCACGACCAGTAGAAGATCCTTATGTATTTGTGGGACAAGTATTAA
4 MT492030.1     CAAATCTTATTTTGAATTATAGTAGTTACAATTATTTTACTAACATGAATTGGAGCACGACCAGTAGAAGATCCTTATGTATTTGTGGGACAAGTATTAA
5 MT492031.1     CAAATCTTATTTTGAATTATAGTAGTTACAATTATTTTACTAACATGAATTGGAGCACGACCAGTAGAAGATCCTTATGTATTTGTGGGACAAGTATTAA
6 MT492032.1     CAAATCTTATTTTGAATTATAGTAGTTACAATTATTTTACTAACATGAATTGGAGCACGACCAGTAGAAGATCCTTATGTATTTGTGGGACAAGTATTAA
7 MT492033.1     CAAATCTTATTTTGAATTATAGTAGTTACAATTATTTTACTAACATGAATTGGAGCACGACCAGTAGAAGATCCTTATGTATTTGTGGGACAAGTATTAA
8 MT492034.1     CAAATCTTATTTTGAATTATAGTAGTTACAATTATTTTACTAACATGAATTGGAGCACGACCAGTAGAAGATCCTTATGTATTTGTGGGACAAGTATTAA
9 MT492035.1     CAAATCTTATTTTGAATTATAGTAGTTACAATTATTTTACTAACATGAATTGGAGCACGACCAGTAGAAGATCCTTATGTATTTGTGGGACAAGTATTAA

                                                                                   | tRNA-Ser (UGA) ->
            11801        .         .         .         .         :         .         .         .         .     9 11900
                 T  I  M  Y  F  S  Y  Y  I  I  N  P  M  I  S  I  Y  W  D  K  L  I  F  N  *aattaatgagcttgtcaaataagcatt
1 MN793322.1     CAATTATATATTTTTCATATTATATTATTAACCCTATAATTAGAATTTATTGAGATAAATTAATCTTTAACTAAATTAATGAGCTTGTCAAATAAGCATT
2 MT492036.1     CAATTATATATTTTTCATATTATATTATTAACCCTATAATTAGAATTTATTGAGATAAATTAATCTTTAACTAAATTAATGAGCTTGTCAAATAAGCATT
3 MT492037.1     CAATTATATATTTTTCATATTATATTATTAACCCTATAATTAGAATTTATTGAGATAAATTAATCTTTAACTAAATTAATGAGCTTGTCAAATAAGCATT
4 MT492030.1     CAATTATATATTTTTCATATTATATTATTAACCCTATAATTAGAATTTATTGAGATAAATTAATCTTTAACTAAATTAATGAGCTTGTCAAATAAGCATT
5 MT492031.1     CAATTATATATTTTTCATATTATATTATTAACCCTATAATTAGAATTTATTGAGATAAATTAATCTTTAACTAAATTAATGAGCTTGTCAAATAAGCATT
6 MT492032.1     CAATTATATATTTTTCATATTATATTATTAACCCTATAATTAGAATTTATTGAGATAAATTAATCTTTAACTAAATTAATGAGCTTGTCAAATAAGCATT
7 MT492033.1     CAATTATATATTTTTCATATTATATTATTAACCCTATAATTAGAATTTATTGAGATAAATTAATCTTTAACTAAATTAATGAGCTTGTCAAATAAGCATT
8 MT492034.1     CAATTATATATTTTTCATATTATATTATTAACCCTATAATTAGAATTTATTGAGATAAATTAATCTTTAACTAAATTAATGAGCTTGTCAAATAAGCATT
9 MT492035.1     CAATTATATATTTTTCATATTATATTATTAACCCTATAATTAGAATTTATTGAGATAAATTAATCTTTAACTAAATTAATGAGCTTGTCAAATAAGCATT

            11901        .         .         .         .         :         .         .         .         .     0 12000
                 tgttttgaaaacttaagaaagaataaatattctattaattt
1 MN793322.1     TGTTTTGAAAACTTAAGAAAGAATAAATATTCTATTAATTTATACTAAAAATATTAACTTACTTACTTAATTAATTCTACTAAAATAATAAAATTTTTAA
2 MT492036.1     TGTTTTGAAAACTTAAGAAAGAATAAATATTCTATTAATTTATACTAAAAATATTAACTTACTTACTTAATTAATTCTACTAAAATAATAAAATTTTTAA
3 MT492037.1     TGTTTTGAAAACTTAAGAAAGAATAAATATTCTATTAATTTATACTAAAAATATTAACTTACTTACTTAATTAATTCTACTAAAATAATAAAATTTTTAA
4 MT492030.1     TGTTTTGAAAACTTAAGAAAGAATAAATATTCTATTAATTTATACTAAAAATATTAACTTACTTACTTAATTAATTCTACTAAAATAATAAAATTTTTAA
5 MT492031.1     TGTTTTGAAAACTTAAGAAAGAATAAATATTCTATTAATTTATACTAAAAATATTAACTTACTTACTTAATTAATTCTACTAAAATAATAAAATTTTTAA
6 MT492032.1     TGTTTTGAAAACTTAAGAAAGAATAAATATTCTATTAATTTATACTAAAAATATTAACTTACTTACTTAATTAATTCTACTAAAATAATAAAATTTTTAA
7 MT492033.1     TGTTTTGAAAACTTAAGAAAGAATAAATATTCTATTAATTTATACTAAAAATATTAACTTACTTACTTAATTAATTCTACTAAAATAATAACATTTTTAA
8 MT492034.1     TGTTTTGAAAACTTAAGAAAGAATAAATATTCTATTAATTTATACTAAAAATATTAACTTACTTACTTAATTAATTCTACTAAAATAATAACATTTTTAA
9 MT492035.1     TGTTTTGAAAACTTAAGAAAGAATAAATATTCTATTAATTTATACTAAAAATATTAACTTACTTACTTAATTAATTCTACTAAAATAATAACATTTTTAA
                                                                                                *  F  L  L  I  K  L
                                                                                               GATTTTATTATTTTAAAAATT
```

```
            12001         .         .         .         .         :         .         .         .         .         1 12100
1 MN793322.1     CCCTAAATAAAATATTAAAAAATTTAAAGAAATAGGTAAATAAATTTTTCAAGCTAAATATATTAATTTATCATAACGATACCGAGGTAATGTTCCACGA
2 MT492036.1     CCCTAAATAAAATATTAAAAAATTTAAAGAAATAGGTAAATAAATTTTTCAAGCTAAATATATTAATTTATCATAACGATACCGAGGTAATGTTCCACGA
3 MT492037.1     CCCTAAATAAAATATTAAAAAATTTAAAGAAATAGGTAAATAAATTTTTCAAGCTAAATATATTAATTTATCATAACGATACCGAGGTAATGTTCCACGA
4 MT492030.1     CCCTAAATAAAATATTAAAAAATTTAAAGAAATAGGTAAATAAATTTTTCAAGCTAAATATATTAATTTATCATAACGATACCGAGGTAATGTTCCACGA
5 MT492031.1     CCCTAAATAAAATATTAAAAAATTTAAAGAAATAGGTAAATAAATTTTTCAAGCTAAATATATTAATTTATCATAACGATACCGAGGTAATGTTCCACGA
6 MT492032.1     CCCTAAATAAAATATTAAAAAATTTAAAGAAATAGGTAAATAAATTTTTCAAGCTAAATATATTAATTTATCATAACGATACCGAGGTAATGTTCCACGA
7 MT492033.1     CCCTAAATAAAATATTAAAAAATTTAAAGAAATAGGTAAATAAATTTTTCAAGCTAAATATATTAATTTATCATAACGATACCGAGGTAATGTTCCACGA
8 MT492034.1     CCCTAAATAAAATATTAAAAAATTTAAAGAAATAGGTAAATAAATTTTTCAAGCTAAATATATTAATTTATCATAACGATACCGAGGTAATGTTCCACGA
9 MT492035.1     CCCTAAATAAAATATTAAAAAATTTAAAGAAATAGGTAAATAAATTTTTCAAGCTAAATATATTAATTTATCATAACGATACCGAGGTAATGTTCCACGA
                  G  L  Y  F  M  L  F  N  L  S  I  P  L  Y  I  K  W  A  L  Y  M  L  K  D  Y  R  Y  R  P  L  T  G  R
                 GGGATTTATTTTATAATTTTTTAAATTTCTTTATCCATTTATTTAAAAAGTTCGATTTATATAATTAAATAGTATTGCTATGGCTCCATTACAAGGTGCT

            12101         .         .         .         .         :         .         .         .         .         2 12200
1 MN793322.1     ACTCAAATAAATAAAAAAGAAATCAACGACAACTTTAAATAAAAAAAAATTCTTAATGAATATCCCCCCATATATAATAAAACAAATAATAAACTTATAA
2 MT492036.1     ACTCAAATAAATAAAAAAGAAATCAACGACAACTTTAAATAAAAAAAAATTCTTAATGAATATCCCCCCATATATAATAAAACAAATAATAAACTTATAA
3 MT492037.1     ACTCAAATAAATAAAAAAGAAATCAACGACAACTTTAAATAAAAAAAAATTCTTAATGAATATCCCCCCATATATAATAAAACAAATAATAAACTTATAA
4 MT492030.1     ACTCAAATAAATAAAAAAGAAATCAACGACAACTTTAAATAAAAAAAAATTCTTAATGAATATCCCCCCATATATAATAAAACAAATAATAAACTTATAA
5 MT492031.1     ACTCAAATAAATAAAAAAGAAATCAACGACAACTTTAAATAAAAAAAAATTCTTAATGAATATCCCCCCATATATAATAAAACAAATAATAAACTTATAA
6 MT492032.1     ACTCAAATAAATAAAAAAGAAATCAACGACAACTTTAAATAAAAAAAAATTCTTAATGAATATCCCCCCATATATAATAAAACAAATAATAAACTTATAA
7 MT492033.1     ACTCAAATAAATAAAAAAGAAATCAACGACAACTTTAAATAAAAAAAAATTCTTAATGAATATCCCCCCATATATAATAAAACAAATAATAAACTTATAA
8 MT492034.1     ACTCAAATAAATAAAAAAGAAATCAACGACAACTTTAAATAAAAAAAAATTCTTAATGAATATCCCCCCATATATAATAAAACAAATAATAAACTTATAA
9 MT492035.1     ACTCAAATAAATAAAAAAGAAATCAACGACAACTTTAAATAAAAAAAAATTCTTAATGAATATCCCCCCATATATAATAAAACAAATAATAAACTTATAA
                 V  W  I  F  L  F  S  I  L  S  L  K  L  Y  F  F  I  S  L  S  Y  G  G  M  Y  L  L  V  F  L  L  S  M  F
                 TGAGTTTATTTATTTTTTCTTTAGTTGCTGTTGAAATTTATTTTTTTTTAAGAATTACTTATAGGGGGGTATATATTATTTTGTTTATTATTTGAATATT

            12201         .         .         .         .         :         .         .         .         .         3 12300
1 MN793322.1     ATAAAATTCTAGAATATTCAGCTAAAAAAATTAAAGCAAACCCCCCACTTCTATACTCAATATTAAATCCAGAAACTAATTCTCTCTCACCTTCAGCAAA
2 MT492036.1     ATAAAATTCTAGAATATTCAGCTAAAAAAATTAAAGCAAACCCCCCACTTCTATACTCAATATTAAATCCAGAAACTAATTCTCTCTCACCTTCAGCAAA
3 MT492037.1     ATAAAATTCTAGAATATTCAGCTAAAAAAATTAAAGCAAACCCCCCACTTCTATACTCAATATTAAATCCAGAAACTAATTCTCTCTCACCTTCAGCAAA
4 MT492030.1     ATAAAATTCTAGAATATTCAGCTAAAAAAATTAAAGCAAACCCCCCACTTCTATACTCAATATTAAATCCAGAAACTAATTCTCTCTCACCTTCAGCAAA
5 MT492031.1     ATAAAATTCTAGAATATTCAGCTAAAAAAATTAAAGCAAACCCCCCACTTCTATACTCAATATTAAATCCAGAAACTAATTCTCTCTCACCTTCAGCAAA
6 MT492032.1     ATAAAATTCTAGAATATTCAGCTAAAAAAATTAAAGCAAACCCCCCACTTCTATACTCAATATTAAATCCAGAAACTAATTCTCTCTCACCTTCAGCAAA
7 MT492033.1     ATAAAATTCTAGAATATTCAGCTAAAAAAATTAAAGCAAACCCCCCACTTCTATACTCAATATTAAATCCAGAAACTAATTCTCTCTCACCTTCAGCAAA
8 MT492034.1     ATAAAATTCTAGAATATTCAGCTAAAAAAATTAAAGCAAACCCCCCACTTCTATACTCAATATTAAATCCAGAAACTAATTCTCTCTCACCTTCAGCAAA
9 MT492035.1     ATAAAATTCTAGAATATTCAGCTAAAAAAATTAAAGCAAACCCCCCACTTCTATACTCAATATTAAATCCAGAAACTAATTCTCTCTCACCTTCAGCAAA
                   L  I  S  S  Y  E  A  L  F  I  L  A  F  G  G  S  S  Y  E  I  N  F  G  S  V  L  E  S  E  G  E  A  F
                 TATTTTAAGATCTTATAAGTCGATTTTTTTAATTTCGTTTGGGGGGTGAAGATATGAGTTATAATTTAGGTCTTTGATTAAGAGAGAGTGGAAGTCGTTT

            12301         .         .         .         .         :         .         .         .         .         4 12400
1 MN793322.1     ATCAAATGGAGTTCGATTAGTTTCAGCTAATCTAGAAGAAAATCAACATAAACCCAAAGGAATTATTAAAAAAAAAAATCAAATTAAATTTTGATAATAT
2 MT492036.1     ATCAAATGGAGTTCGATTAGTTTCAGCTAATCTAGAAGAAAATCAACATAAACCCAAAGGAATTATTAAAAAAAAAAATCAAATTAAATTTTGATAATAT
3 MT492037.1     ATCAAATGGAGTTCGATTAGTTTCAGCTAATCTAGAAGAAAATCAACATAAACCCAAAGGAATTATTAAAAAAAAAAATCAAATTAAATTTTGATAATAT
4 MT492030.1     ATCAAATGGAGTTCGATTAGTTTCAGCTAATCTAGAAGAAAATCAACATAAACCCAAAGGAATTATTAAAAAAAAAAATCAAATTAAATTTTGATAATAT
5 MT492031.1     ATCAAATGGAGTTCGATTAGTTTCAGCTAATCTAGAAGAAAATCAACATAAACCCAAAGGAATTATTAAAAAAAAAAATCAAATTAAATTTTGATAATAT
6 MT492032.1     ATCAAATGGAGTTCGATTAGTTTCAGCTAATCTAGAAGAAAATCAACATAAACCCAAAGGAATTATTAAAAAAAAAAATCAAATTAAATTTTGATAATAT
7 MT492033.1     ATCAAATGGAGTTCGATTAGTTTCAGCTAATCTAGAAGAAAATCAACATAAACCCAAAGGAATTATTAAAAAAAAAAATCAAATTAAATTTTGATAATAT
8 MT492034.1     ATCAAATGGAGTTCGATTAGTTTCAGCTAATCTAGAAGAAAATCAACATAAACCCAAAGGAATTATTAAAAAAAAAAATCAAATTAAATTTTGATAATAT
9 MT492035.1     ATCAAATGGAGTTCGATTAGTTTCAGCTAATCTAGAAGAAAATCAACATAAACCCAAAGGAATTATTAAAAAAAAAAATCAAATTAAATTTTGATAATAT
```

```
                D  F  P  T  R  N  T  E  A  L  S  S  S  F  W  C  L  G  L  P  I  M  L  F  F  F  W  I  L  N  Q  Y  Y
               TAGTTTACCTCAAGCTAATCAAAGTCGATTAGATCTTCTTTTAGTTGTATTTGGGTTTCCTTAATAATTTTTTTTTTAGTTTAATTTAAAACTATTATA

          12401         .         .         .         .         :         .         .         .         .         5 12500
1 MN793322.1   ATAAAAATTACTAAATTAAAATCTATAACTATAATAATTCTTGATATTAAAATTAAAGCTAAACTAACCTCATATGAAATAGTTTGAGCTACTGCCCGTA
2 MT492036.1   ATAAAAATTACTAAATTAAAATCTATAACTATAATAATTCTTGATATTAAAATTAAAGCTAAACTAACCTCATATGAAATAGTTTGAGCTACTGCCCGTA
3 MT492037.1   ATAAAAATTACTAAATTAAAATCTATAACTATAATAATTCTTGATATTAAAATTAAAGCTAAACTAACCTCATATGAAATAGTTTGAGCTACTGCCCGTA
4 MT492030.1   ATAAAAATTACTAAATTAAAATCTATAACTATAATAATTCTTGATATTAAAATTAAAGCTAAACTAACCTCATATGAAATAGTTTGAGCTACTGCCCGTA
5 MT492031.1   ATAAAAATTACTAAATTAAAATCTATAACTATAATAATTCTTGATATTAAAATTAAAGCTAAACTAACCTCATATGAAATAGTTTGAGCTACTGCCCGTA
6 MT492032.1   ATAAAAATTACTAAATTAAAATCTATAACTATAATAATTCTTGATATTAAAATTAAAGCTAAACTAACCTCATATGAAATAGTTTGAGCTACTGCCCGTA
7 MT492033.1   ATAAAAATTACTAAATTAAAATCTATAACTATAATAATTCTTGATATTAAAATTAAAGCTAAACTAACCTCATATGAAATAGTTTGAGCTACTGCCCGTA
8 MT492034.1   ATAAAAATTACTAAATTAAAATCTATAACTATAATAATTCTTGATATTAAAATTAAAGCTAAACTAACCTCATATGAAATAGTTTGAGCTACTGCCCGTA
9 MT492035.1   ATAAAAATTACTAAATTAAAATCTATAACTATAATAATTCTTGATATTAAAATTAAAGCTAAACTAACCTCATATGAAATAGTTTGAGCTACTGCCCGTA
               M  F  I  V  L  N  F  D  M  V  M  I  I  S  S  M  L  I  L  A  L  S  V  E  Y  S  I  T  Q  A  V  A  R  L
               TATTTTTAATGATTTAATTTTAGATATTGATATTATTAAGAACTATAATTTTAATTTCGATTTGATTGGAGTATACTTTATCAAACTCGATGACGGGCAT

          12501         .         .         .         .         :         .         .         .         .         6 12600
1 MN793322.1   AACCCCCTAATAAAGCATAATTAGAATTAGATGACCAACCAGAAACTATTAAAGTATAAACTCCTAAACTAGTACAACATAAAAAAAATAAAATACCTAA
2 MT492036.1   AACCCCCTAATAAAGCATAATTAGAATTAGATGACCAACCAGAAACTATTAAAGTATAAACTCCTAAACTAGTACAACATAAAAAAAATAAAATACCTAA
3 MT492037.1   AACCCCCTAATAAAGCATAATTAGAATTAGATGACCAACCAGAAACTATTAAAGTATAAACTCCTAAACTAGTACAACATAAAAAAAATAAAATACCTAA
4 MT492030.1   AACCCCCTAATAAAGCATAATTAGAATTAGATGACCAACCAGAAACTATTAAAGTATAAACTCCTAAACTAGTACAACATAAAAAAAATAAAATACCTAA
5 MT492031.1   AACCCCCTAATAAAGCATAATTAGAATTAGATGACCAACCAGAAACTATTAAAGTATAAACTCCTAAACTAGTACAACATAAAAAAAATAAAATACCTAA
6 MT492032.1   AACCCCCTAATAAAGCATAATTAGAATTAGATGACCAACCAGAAACTATTAAAGTATAAACTCCTAAACTAGTACAACATAAAAAAAATAAAATACCTAA
7 MT492033.1   AACCCCCTAATAAAGCATAATTAGAATTAGATGACCAACCAGAAACTATTAAAGTATAAACTCCTAAACTAGTACAACATAAAAAAAATAAAATACCTAA
8 MT492034.1   AACCCCCTAATAAAGCATAATTAGAATTAGATGACCAACCAGAAACTATTAAAGTATAAACTCCTAAACTAGTACAACATAAAAAAAATAAAATACCTAA
9 MT492035.1   AACCCCCTAATAAAGCATAATTAGAATTAGATGACCAACCAGAAACTATTAAAGTATAAACTCCTAAACTAGTACAACATAAAAAAAATAAAATACCTAA
                  G  G  L  L  A  Y  N  S  N  S  S  W  G  S  V  M  L  T  Y  V  G  L  S  T  C  C  L  F  F  L  I  G  L
               TTGGGGGATTATTTCGTATTAATCTTAATCTACTGGTTGGTCTTTGATAATTTCATATTTGAGGATTTGATCATGTTGTATTTTTTTTATTTTATGGATT

          12601         .         .         .         .         :         .         .         .         .         7 12700
1 MN793322.1   ATTAAAATTTACTAAATTAAAATAATATGGAATTATAACTCAAATTATTAAAGATAAAATAAATCTAATAACAGGAGAAAAATAATAAGATAAATAATTT
2 MT492036.1   ATTAAAATTTACTAAATTAAAATAATATGGAATTATAACTCAAATTATTAAAGATAAAATAAATCTAATAACAGGAGAAAAATAATAAGATAAATAATTT
3 MT492037.1   ATTAAAATTTACTAAATTAAAATAATATGGAATTATAACTCAAATTATTAAAGATAAAATAAATCTAATAACAGGAGAAAAATAATAAGATAAATAATTT
4 MT492030.1   ATTAAAATTTACTAAATTAAAATAATATGGAATTATAACTCAAATTATTAAAGATAAAATAAATCTAATAACAGGAGAAAAATAATAAGATAAATAATTT
5 MT492031.1   ATTAAAATTTACTAAATTAAAATAATATGGAATTATAACTCAAATTATTAAAGATAAAATAAATCTAATAACAGGAGAAAAATAATAAGATAAATAATTT
6 MT492032.1   ATTAAAATTTACTAAATTAAAATAATATGGAATTATAACTCAAATTATTAAAGATAAAATAAATCTAATAACAGGAGAAAAATAATAAGATAAATAATTT
7 MT492033.1   ATTAAAATTTACTAAATTAAAATAATATGGAATTATAACTCAAATTATTAAAGATAAAATAAATCTAATAACAGGAGAAAAATAATAAGATAAATAATTT
8 MT492034.1   ATTAAAATTTACTAAATTAAAATAATATGGAATTATAACTCAAATTATTAAAGATAAAATAAATCTAATAACAGGAGAAAAATAATAAGATAAATAATTT
9 MT492035.1   ATTAAAATTTACTAAATTAAAATAATATGGAATTATAACTCAAATTATTAAAGATAAAATAAATCTAATAACAGGAGAAAAATAATAAGATAAATAATTT
                N  F  N  V  L  N  F  Y  Y  P  I  M  V  W  I  M  L  S  L  I  F  S  I  V  P  S  F  Y  Y  S  L  Y  N
               TAATTTTAAATGATTTAATTTTATTATACCTTAATATTGAGTTTAATAATTTCTATTTTATTTAGATTATTGTCCTCTTTTTATTATTCTATTTATTAAA

          12701         .         .         .         .         :         .         .         .         .         8 12800
1 MN793322.1   GAAAATAAAGGATAAGTCTGTTCCTTAGTAAATAATTTAATAGCATCTGAAAAAGGTTGTAAAATTCCTATAAACCCAACTTTATTAGGACCTTTACGAA
2 MT492036.1   GAAAATAAAGGATAAGTCTGTTCCTTAGTAAATAATTTAATAGCATCTGAAAAAGGTTGTAAAATTCCTATAAACCCAACTTTATTAGGACCTTTACGAA
3 MT492037.1   GAAAATAAAGGATAAGTCTGTTCCTTAGTAAATAATTTAATAGCATCTGAAAAAGGTTGTAAAATTCCTATAAACCCAACTTTATTAGGACCTTTACGAA
4 MT492030.1   GAAAATAAAGGATAAGTCTGTTCCTTAGTAAATAATTTAATAGCATCTGAAAAAGGTTGTAAAATTCCTATAAACCCAACTTTATTAGGACCTTTACGAA
5 MT492031.1   GAAAATAAAGGATAAGTCTGTTCCTTAGTAAATAATTTAATAGCATCTGAAAAAGGTTGTAAAATTCCTATAAACCCAACTTTATTAGGACCTTTACGAA
6 MT492032.1   GAAAATAAAGGATAAGTCTGTTCCTTAGTAAATAATTTAATAGCATCTGAAAAAGGTTGTAAAATTCCTATAAACCCAACTTTATTAGGACCTTTACGAA
7 MT492033.1   GAAAATAAAGGATAAGTCTGTTCCTTAGTAAATAATTTAATAGCATCTGAAAAAGGTTGTAAAATTCCTATAAACCCAACTTTATTAGGACCTTTACGAA
8 MT492034.1   GAAAATAAAGGATAAGTCTGTTCCTTAGTAAATAATTTAATAGCATCTGAAAAAGGTTGTAAAATTCCTATAAACCCAACTTTATTAGGACCTTTACGAA
9 MT492035.1   GAAAATAAAGGATAAGTCTGTTCCTTAGTAAATAATTTAATAGCATCTGAAAAAGGTTGTAAAATTCCTATAAACCCAACTTTATTAGGACCTTTACGAA
               S  F  L  P  Y  T  Q  E  K  T  F  L  K  I  A  D  S  F  P  Q  L  I  G  M  F  G  V  K  N  P  G  K  R  I
               CTTTTATTTCCTATTCAGACAAGGAATCATTTATTAAATTATCGTAGACTTTTTCCAACATTTTAAGGATATTTGGGTTGAAATAATCCTGGAAATGCTT
```

```
            12801         .         .         .         .         :         .         .         .         .       9 12900
1 MN793322.1     TTTGAATATAACCTAAAACCTTCCGCTCTAATAAAGTTAAATATGCAACCCCAATTAAAACACCAAGAATTAAAATAATAAATCCCAAAAAAATTAATAA
2 MT492036.1     TTTGAATATAACCTAAAACCTTCCGCTCTAATAAAGTTAAATATGCAACCCCAATTAAAACACCAAGAATTAAAATAATAAATCCCAAAAAAATTAATAA
3 MT492037.1     TTTGAATATAACCTAAAACCTTCCGCTCTAATAAAGTTAAATATGCAACCCCAATTAAAACACCAAGAATTAAAATAATAAATCCCAAAAAAATTAATAA
4 MT492030.1     TTTGAATATAACCTAAAACCTTCCGCTCTAATAAAGTTAAATATGCAACCCCAATTAAAACACCAAGAATTAAAATAATAAATCCCAAAAAAATTAATAA
5 MT492031.1     TTTGAATATAACCTAAAACCTTCCGCTCTAATAAAGTTAAATATGCAACCCCAATTAAAACACCAAGAATTAAAATAATAAATCCCAAAAAAATTAATAA
6 MT492032.1     TTTGAATATAACCTAAAACCTTCCGCTCTAATAAAGTTAAATATGCAACCCCAATTAAAACACCAAGAATTAAAATAATAAATCCCAAAAAAATTAATAA
7 MT492033.1     TTTGAATATAACCTAAAACCTTCCGCTCTAATAAAGTTAAATATGCAACCCCAATTAAAACACCAAGAATTAAAATAATAAATCCCAAAAAAATTAATAA
8 MT492034.1     TTTGAATATAACCTAAAACCTTCCGCTCTAATAAAGTTAAATATGCAACCCCAATTAAAACACCAAGAATTAAAATAATAAATCCCAAAAAAATTAATAA
9 MT492035.1     TTTGAATATAACCTAAAACCTTCCGCTCTAATAAAGTTAAATATGCAACCCCAATTAAAACACCAAGAATTAAAATAATAAATCCCAAAAAAATTAATAA
                  Q  I  Y  G  L  V  K  R  E  L  L  T  L  Y  A  V  G  I  L  V  G  L  I  L  I  I  F  G  L  F  I  L  L
                 AAACTTATATTGGATTTTGGAAGGCGAGATTATTTCAATTTATACGTTGGGGTTAATTTTGTGGTTCTTAATTTTATTATTTAGGGTTTTTTTAATTATT

            12901         .         .         .         .         :         .         .         .         .       0 13000
1 MN793322.1     AAAATCAATATAAAACATTTACTATCCATATAAATAAATTATACATTTATGATTTCTAAAATCACTACACTTTTCTGCCAAAATAGTTTTTAAATTTAAA
2 MT492036.1     AAAATCAATATAAAACATTTACTATCCATATAAATAAATTATACATTTATGATTTCTAAAATCACTACACTTTTCTGCCAAAATAGTTTTTAAATTTAAA
3 MT492037.1     AAAATCAATATAAAACATTTACTATCCATATAAATAAATTATACATTTATGATTTCTAAAATCACTACACTTTTCTGCCAAAATAGTTTTTAAATTTAAA
4 MT492030.1     AAAATCAATATAAAACATTTACTATCCATATAAATAAATTATACATTTATGATTTCTAAAATCACTACACTTTTCTGCCAAAATAGTTTTTAAATTTAAA
5 MT492031.1     AAAATCAATATAAAACATTTACTATCCATATAAATAAATTATACATTTATGATTTCTAAAATCACTACACTTTTCTGCCAAAATAGTTTTTAAATTTAAA
6 MT492032.1     AAAATCAATATAAAACATTTACTATCCATATAAATAAATTATACATTTATGATTTCTAAAATCACTACACTTTTCTGCCAAAATAGTTTTTAAATTTAAA
7 MT492033.1     AAAATCAATATAAAACATTTACTATCCATATAAATAAATTATACATTTATGATTTCTAAAATCACTACACTTTTCTGCCAAAATAGTTTTTAAATTTAAA
8 MT492034.1     AAAATCAATATAAAACATTTACTATCCATATAAATAAATTATACATTTATGATTTCTAAAATCACTACACTTTTCTGCCAAAATAGTTTTTAAATTTAAA
9 MT492035.1     AAAATCAATATAAAACATTTACTATCCATATAAATAAATTATACATTTATGATTTCTAAAATCACTACACTTTTCTGCCAAAATAGTTTTTAAATTTAAA
                  F  D  M           atgataggtatatttatttaatatgtaaatactaaagattttagtgatgtgaaaagacggttttatcaaaaatttaaattt
                 TTTTAGTTA          | tRNA-Leu (UGA) ->                                                 |
                  <- ND1 |

            13001         .         .         .         .         :         .         .         .         .       1 13100
1 MN793322.1     TTAAACATTAATAAAATTCATTAAATAAATTTAATTTAAATATTTATTCCTTTCGTACTAAAATATTTTAATATTTTAAAGATAGAAACCAACCTGGCTC
2 MT492036.1     TTAAACATTAATAAAATTCATTAAATAAATTTAATTTAAATATTTATTCCTTTCGTACTAAAATATTTTAATATTTTAAAGATAGAAACCAACCTGGCTC
3 MT492037.1     TTAAACATTAATAAAATTCATTAAATAAATTTAATTTAAATATTTATTCCTTTCGTACTAAAATATTTTAATATTTTAGTGCTAGAGGCCTACCTGGCTC
4 MT492030.1     TTAAACATTAATAAAATTCATTAAATAAATTTAATTTAAATATTTATTCCTTTCGTACTAAAATATTTTAATATTTTAAAGATAGAAACCAACCTGGCTC
5 MT492031.1     TTAAACATTAATAAAATTCATTAAATAAATTTAATTTAAATATTTATTCCTTTCGTACTAAAATATTTTAATATTTTAAAGATAGAAACCAACCTGGCTC
6 MT492032.1     TTAAACATTAATAAAATTCATTAAATAAATTTAATTTAAATATTTATTCCTTTCGTACTAAAATATTTTAATATTTTAAAGATAGAAACCAACCTGGCTC
7 MT492033.1     TTAAACATTAATAAAATTCATTAAATAAATTTAATTTAAATATTTATTCCTTTCGTACTAAAATATTTTAATATTTTAAAGATAGAAACCAACCTGGCTC
8 MT492034.1     TTAAACATTAATAAAATTCATTAAATAAATTTAATTTAAATATTTATTCCTTTCGTACTAAAATATTTTAATATTTTAAAGATAGAAACCAACCTGGCTC
9 MT492035.1     TTAAACATTAATAAAATTCATTAAATAAATTTAATTTAAATATTTATTCCTTTCGTACTAAAATATTTTAATATTTTAAAGATAGAAACCAACCTGGCTC
                 aatttgtaattattttaagtaatttatttaaattaaatttataaataaggaaagcatgattttataaaattataaaatttctatctttggttggaccgag

            13101         .         .         .         .         :         .         .         .         .       2 13200
1 MN793322.1     ACACCGGTTTGAACTCAGATCATGTAAGATTTTAATGATCGAACAGATCAAAATTTTATACTTTTGCATATAAATTTTATCTTAATCCAACATCGAGGTC
2 MT492036.1     ACACCGGTTTGAACTCAGATCATGTAAGATTTTAATGATCGAACAGATCAAAATTTTATACTTTTGCATATAAATTTTATCTTAATCCAACATCGAGGTC
3 MT492037.1     ACACCGGTTTGAACTCAGATCATGTAAGATTTTAATGATCGAACAGATCAAAATTTTATACTTTTGCATATAAATTTTATCTTAATCCAACATCGAGGTC
4 MT492030.1     ACACCGGTTTGAACTCAGATCATGTAAGATTTTAATGATCGAACAGATCAAAATTTTATACTTTTGCATATAAATTTTATCTTAATCCAACATCGAGGTC
5 MT492031.1     ACACCGGTTTGAACTCAGATCATGTAAGATTTTAATGATCGAACAGATCAAAATTTTATACTTTTGCATATAAATTTTATCTTAATCCAACATCGAGGTC
6 MT492032.1     ACACCGGTTTGAACTCAGATCATGTAAGATTTTAATGATCGAACAGATCAAAATTTTATACTTTTGCATATAAATTTTATCTTAATCCAACATCGAGGTC
7 MT492033.1     ACACCGGTTTGAACTCAGATCATGTAAGATTTTAATGATCGAACAGATCAAAATTTTATACTTTTGCATATAAATTTTATCTTAATCCAACATCGAGGTC
8 MT492034.1     ACACCGGTTTGAACTCAGATCATGTAAGATTTTAATGATCGAACAGATCAAAATTTTATACTTTTGCATATAAATTTTATCTTAATCCAACATCGAGGTC
9 MT492035.1     ACACCGGTTTGAACTCAGATCATGTAAGATTTTAATGATCGAACAGATCAAAATTTTATACTTTTGCATATAAATTTTATCTTAATCCAACATCGAGGTC
                 tgtggccaaacttgagtctagtacattctaaaattactagcttgtctagttttaaaatatgaaaacgtatatttaaaatagaattaggttgtagctccag
```

```
             13201         .         .         .         .         :         .         .         .         .        3 13300
1 MN793322.1     GCAAACTCTTTTTCTTATTTGAACTAAAAAAAAAAATTACGCTGTTATCCCTAAGGTAATTTTTTCTTATAATCAAAAATTTTGGATCAATTACTCACTT
2 MT492036.1     GCAAACTCTTTTTCTTATTTGAACTAAAAAAAAAAATTACGCTGTTATCCCTAAGGTAATTTTTTCTTATAATCAAAAATTTTGGATCAATTACTCACTT
3 MT492037.1     GCAAACTCTTTTTCTTATTTGAACTAAAAAAAAAAATTACGCTGTTATCCCTAAGGTAATTTTTTCTTATAATCAAAAATTTTGGATCAATTACTCACTT
4 MT492030.1     GCAAACTCTTTTTCTTATTTGAACTAAAAAAAAAAATTACGCTGTTATCCCTAAGGTAATTTTTTCTTATAATCAAAAATTTTGGATCAATTACTCACTT
5 MT492031.1     GCAAACTCTTTTTCTTATTTGAACTAAAAAAAAAAATTACGCTGTTATCCCTAAGGTAATTTTTTCTTATAATCAAAAATTTTGGATCAATTACTCACTT
6 MT492032.1     GCAAACTCTTTTTCTTATTTGAACTAAAAAAAAAAATTACGCTGTTATCCCTAAGGTAATTTTTTCTTATAATCAAAAATTTTGGATCAATTACTCACTT
7 MT492033.1     GCAAACTCTTTTTCTTATTTGAACTAAAAAAAAAAATTACGCTGTTATCCCTAAGGTAATTTTTTCTTATAATCAAAAATTTTGGATCAATTACTCACTT
8 MT492034.1     GCAAACTCTTTTTCTTATTTGAACTAAAAAAAAAAATTACGCTGTTATCCCTAAGGTAATTTTTTCTTATAATCAAAAATTTTGGATCAATTACTCACTT
9 MT492035.1     GCAAACTCTTTTTCTTATTTGAACTAAAAAAAAAAATTACGCTGTTATCCCTAAGGTAATTTTTTCTTATAATCAAAAATTTTGGATCAATTACTCACTT
                 cgtttgagaaaaagaataaacttgatttttttttttaatgcgacaatagggattccattaaaaaagaatattagtttttaaaacctagttaatgagtgaa

             13301         .         .         .         .         :         .         .         .         .        4 13400
1 MN793322.1     ATCTATGAAATAATATATTAAAAAAAGTTAATTTTATTTTTTTATCACCCCAATAAAATATTATTAAATAATTTAATTAATAATCTAAAAAATAATTTTA
2 MT492036.1     ATCTATGAAATAATATATTAAAAAAAGTTAATTTTATTTTTTTATCACCCCAATAAAATATTATTAAATAATTTAATTAATAATCTAAAAAATAATTTTA
3 MT492037.1     ATCTATGAAATAATATATTAAAAAAAGTTAATTTTATTTTTTTATCACCCCAATAAAATATTATTAAATAATTTAATTAATAATCTAAAAAATAATTTTA
4 MT492030.1     ATCTATGAAATAATATATTAAAAAAAGTTAATTTTATTTTTTTATCACCCCAATAAAATATTATTAAATAATTTAATTAATAATCTAAAAAATAATTTTA
5 MT492031.1     ATCTATGAAATAATATATTAAAAAAAGTTAATTTTATTTTTTTATCACCCCAATAAAATATTATTAAATAATTTAATTAATAATCTAAAAAATAATTTTA
6 MT492032.1     ATCTATGAAATAATATATTAAAAAAAGTTAATTTTATTTTTTTATCACCCCAATAAAATATTATTAAATAATTTAATTAATAATCTAAAAAATAATTTTA
7 MT492033.1     ATCTATGAAATAATATATTAAAAAAAGTTAATTTTATTTTTTTATCACCCCAATAAAATATTATTAAATAATTTAATTAATAATCTAAAAAATAATTTTA
8 MT492034.1     ATCTATGAAATAATATATTAAAAAAAGTTAATTTTATTTTTTTATCACCCCAATAAAATATTATTAAATAATTTAATTAATAATCTAAAAAATAATTTTA
9 MT492035.1     ATCTATGAAATAATATATTAAAAAAAGTTAATTTTATTTTTTTATCACCCCAATAAAATATTATTAAATAATTTAATTAATAATCTAAAAAATAATTTTA
                 tagatactttattatataatttttttcaattaaaataaaaaaatagtggggttattttataataatttattaaattaattattagattttttattaaaat

             13401         .         .         .         .         :         .         .         .         .        5 13500
1 MN793322.1     TTATATATTAATATAAAACTCTATAGGGTCTTCTCGTCTTTTAAATAAA-TTTTGACTTTTTAATCAAAAAATTAAATTAATTAATAATAAATAAGAGAC
2 MT492036.1     TTATATATTAATATAAAACTCTATAGGGTCTTCTCGTCTTTTAAATAAA-TTTTGACTTTTTAATCAAAAAATTAAATTAATTAATAATAAATAAGAGAC
3 MT492037.1     TTATATATTAATATAAAACTCTATAGGGTCTTCTCGTCTTTTAAATAAA-TTTTGACTTTTTAATCAAAAAATTAAATTAATTAATAATAAATAAGAGAC
4 MT492030.1     TTATATATTAATATAAAACTCTATAGGGTCTTCTCGTCTTTTAAATAAA-TTTTGACTTTTTAATCAAAAAATTAAATTAATTAATAATAAATAAGAGAC
5 MT492031.1     TTATATATTAATATAAAACTCTATAGGGTCTTCTCGTCTTTTAAATAAA-TTTTGACTTTTTAATCAAAAAATTAAATTAATTAATAATAAATAAGAGAC
6 MT492032.1     TTATATATTAATATAAAACTCTATAGGGTCTTCTCGTCTTTTAAATAAA-TTTTGACTTTTTAATCAAAAAATTAAATTAATTAATAATAAATAAGAGAC
7 MT492033.1     TTATATATTAATATAAAACTCTA-AGGGTCTTCTCGTCTTTTAAATAAAATTTTGACTTTTTAATCAAAAAATTAAATTAATTAATAATAAATAAGAGAC
8 MT492034.1     TTATATATTAATATAAAACTCTATAGGGTCTTCTCGTCTTTTAAATAAAATTTTGACTTTTTAATCAAAAAATTAAATTAATTAATAATAAATAAGAGAC
9 MT492035.1     TTATATATTAATATAAAACTCTATAGGGTCTTCTCGTCTTTTAAATAAA-TTTTGACTTTTTAATCAAAAAATTAAATTAATTAATAATAAATAAGAGAC
                 aatatataattatattttgagatatcccagaagagcagaaaatttattttaaaactgaaaaattagtttttaatttaattaattattatttattctctg

             13501         .         .         .         .         :         .         .         .         .        6 13600
1 MN793322.1     AGCTTATATTTCATCCAATCTTTCATACAAGTCATCAATTAAAAAACTATTTATTATGCTACCTTTGTACAGTCAATATACTGCAGCCCTTTAATAATTA
2 MT492036.1     AGCTTATATTTCATCCAATCTTTCATACAAGTCATCAATTAAAAAACTATTTATTATGCTACCTTTGTACAGTCAATATACTGCAGCCCTTTAATAATTA
3 MT492037.1     AGCTTATATTTCATCCAATCTTTCATACAAGTCATCAATTAAAAAACTATTTATTATGCTACCTTTGTACAGTCAATATACTGCAGCCCTTTAATAATTA
4 MT492030.1     AGCTTATATTTCATCCAATCTTTCATACAAGTCATCAATTAAAAAACTATTTATTATGCTACCTTTGTACAGTCAATATACTGCAGCCCTTTAATAATTA
5 MT492031.1     AGCTTATATTTCATCCAATCTTTCATACAAGTCATCAATTAAAAAACTATTTATTATGCTACCTTTGTACAGTCAATATACTGCAGCCCTTTAATAATTA
6 MT492032.1     AGCTTATATTTCATCCAATCTTTCATACAAGTCATCAATTAAAAAACTATTTATTATGCTACCTTTGTACAGTCAATATACTGCAGCCCTTTAATAATTA
7 MT492033.1     AGCTTATATTTCATCCAATCTTTCATACAAGTCATCAATTAAAAAACTATTTATTATGCTACCTTTGTACAGTCAATATACTGCAGCCCTTTAATAATTA
8 MT492034.1     AGCTTATATTTCATCCAATCTTTCATACAAGTCATCAATTAAAAAACTATTTATTATGCTACCTTTGTACAGTCAATATACTGCAGCCCTTTAATAATTA
9 MT492035.1     AGCTTATATTTCATCCAATCTTTCATACAAGTCATCAATTAAAAAACTATTTATTATGCTACCTTTGTACAGTCAATATACTGCAGCCCTTTAATAATTA
                 tcgaatataaagtaggttagaaagtatgttcagtagttaattttttgataaataatacgatggaaacatgtcagttatatgacgtcgggaaattattaat
```

```
             13601         .         .         .         .         :         .         .         .         .       7 13700
1 MN793322.1     TCAGTGGGCAGATTAGACTTTAAATTATTAACAAAAAGACATGTTTTTGATAAACAAGTGAACATAAAATTTTGCCGAATTCCTTTTATTTAAATTTTAA
2 MT492036.1     TCAGTGGGCAGATTAGACTTTAAATTATTAACAAAAAGACATGTTTTTGATAAACAAGTGAACATAAAATTTTGCCGAATTCCTTTTATTTAAATTTTAA
3 MT492037.1     TCAGTGGGCAGATTAGACTTTAAATTATTAACAAAAAGACATGTTTTTGATAAACAAGTGAACATAAAATTTTGCCGAATTCCTTTTATTTAAATTTTAA
4 MT492030.1     TCAGTGGGCAGATTAGACTTTAAATTATTAACAAAAAGACATGTTTTTGATAAACAAGTGAACATAAAATTTTGCCGAATTCCTTTTATTTAAATTTTAA
5 MT492031.1     TCAGTGGGCAGATTAGACTTTAAATTATTAACAAAAAGACATGTTTTTGATAAACAAGTGAACATAAAATTTTGCCGAATTCCTTTTATTTAAATTTTAA
6 MT492032.1     TCAGTGGGCAGATTAGACTTTAAATTATTAACAAAAAGACATGTTTTTGATAAACAAGTGAACATAAAATTTTGCCGAATTCCTTTTATTTAAATTTTAA
7 MT492033.1     TCAGTGGGCAGATTAGACTTTAAATTATTAACAAAAAGACATGTTTTTGATAAACAAGTGAACATAAAATTTTGCCGAATTCCTTTTATTTAAATTTTAA
8 MT492034.1     TCAGTGGGCAGATTAGACTTTAAATTATTAACAAAAAGACATGTTTTTGATAAACAAGTGAACATAAAATTTTGCCGAATTCCTTTTATTTAAATTTTAA
9 MT492035.1     TCAGTGGGCAGATTAGACTTTAAATTATTAACAAAAAGACATGTTTTTGATAAACAAGTGAACATAAAATTTTGCCGAATTCCTTTTATTTAAATTTTAA
                 agtcacccgtctaatctgaaatttaataattgtttttctgtacaaaaactatttgttcacttgtattttaaaacggcttaaggaaaataaatttaaaatt

             13701         .         .         .         .         :         .         .         .         .       8 13800
1 MN793322.1     AATATTAAATAAATTTTTTATAAATTTAATACTAATTTTATCATTATAACTAAATTTATAAAATTTAAAATTATTTTTTTATAAAAAATTAAATTAATTT
2 MT492036.1     AATATTAAATAAATTTTTTATAAATTTAATACTAATTTTATCATTATAACTAAATTTATAAAATTTAAAATTATTTTTTTATAAAAAATTAAATTAATTT
3 MT492037.1     AATATTAAATAAATTTTTTATAAATTTAATACTAATTTTATCATTATAACTAAATTTATAAAATTTAAAATTATTTTTTTATAAAAAATTAAATTAATTT
4 MT492030.1     AATATTAAATAAATTTTTTATAAATTTAATACTAATTTTATCATTATAACTAAATTTATAAAATTTAAAATTATTTTTTTATAAAAAATTAAATTAATTT
5 MT492031.1     AATATTAAATAAATTTTTTATAAATTTAATACTAATTTTATCATTATAACTAAATTTATAAAATTTAAAATTATTTTTTTATAAAAAATTAAATTAATTT
6 MT492032.1     AATATTAAATAAATTTTTTATAAATTTAATACTAATTTTATCATTATAACTAAATTTATAAAATTTAAAATTATTTTTTTATAAAAAATTAAATTAATTT
7 MT492033.1     AATATTAAATAAATTTTTTATAAATTTAATACTAATTTTATCATTATAACTAAATTTATAAAATTTAAAATTATTTTTTTATAAAAAATTAAATTAATTT
8 MT492034.1     AATATTAAATAAATTTTTTATAAATTTAATACTAATTTTATCATTATAACTAAATTTATAAAATTTAAAATTATTTTTTTATAAAAAATTAAATTAATTT
9 MT492035.1     AATATTAAATAAATTTTTTATAAATTTAATACTAATTTTATCATTATAACTAAATTTATAAAATTTAAAATTATTTTTTTATAAAAAATTAAATTAATTT
                 ttataatttatttaaaaaatatttaaattatgattaaaatagtaatattgatttaaatattttaaattttaataaaaaaatatttttttaatttaattaaa

             13801         .         .         .         .         :         .         .         .         .       9 13900
1 MN793322.1     AAAATTTTATATTTTAATTAAAATTATTTTATAATAAATTAAATTTTATAAACAATATAAATCTTAAAAATTTTAATTAAATAAAAATATAATTATAAAA
2 MT492036.1     AAAATTTTATATTTTAATTAAAATTATTTTATAATAAATTAAATTTTATAAACAATATAAATTTTAAAAATTTTAATTAAATAAAAATATAATTATAAAA
3 MT492037.1     AAAATTTTATATTTTAATTAAAATTATTTTATAATAAATTAAATTTTATAAACAATATAAATTTTAAAAATTTTAATTAAATAAAAATATAATTATAAAA
4 MT492030.1     AAAATTTTATATTTTAATTAAAATTATTTTATAATAAATTAAATTTTATAAACAATATAAATTTTAAAAATTTTAATTAAATAAAAATATAATTATAAAA
5 MT492031.1     AAAATTTTATATTTTAATTAAAATTATTTTATAATAAATTAAATTTTATAAACAATATAAATTTTAAAAATTTTAATTAAATAAAAATATAATTATAAAA
6 MT492032.1     AAAATTTTATATTTTAATTAAAATTATTTTATAATAAATTAAATTTTATAAACAATATAAATTTTAAAAATTTTAATTAAATAAAAATATAATTATAAAA
7 MT492033.1     AAAATTTTATATTTTAATTAAAATTATTTTATAATAAATTAAATTTTATAAACAATATAAATTTTAAAAATTTTAATTAAATAAAAATATAATTATAAAA
8 MT492034.1     AAAATTTTATATTTTAATTAAAATTATTTTATAATAAATTAAATTTTATAAACAATATAAATTTTAAAAATTTTAATTAAATAAAAATATAATTATAAAA
9 MT492035.1     AAAATTTTATATTTTAATTAAAATTATTTTATAATAAATTAAATTTTATAAACAATATAAATTTTAAAAATTTTAATTAAATAAAAATATAATTATAAAA
                 ttttaaaatataaaattaattttaataaaatattatttaatttaaaatatttgttatatttaaaatttttaaaattaatttatttttatattaatatttt

             13901         .         .         .         .         :         .         .         .         .       0 14000
1 MN793322.1     ATTTAATTTAAAGCTTATCCCTTAAAATATTAAAATTTATTTTAAATTAATTAATTAATTAATTAAATTTATAAATAAATTTAAAATTAAATTTTTTTCT
2 MT492036.1     ATTTAATTTAAAGCTTATCCCTTAAAATATTAAAATTTATTTTAAATTAATTAATTAATTAATTAAATTTATAAATAAATTTAAAATTAAATTTTTTTCT
3 MT492037.1     ATTTAATTTAAAGCTTATCCCTTAAAATATTAAAATTTATTTTAAATTAATTAATTAATTAATTAAATTTATAAATAAATTTAAAATTAAATTTTTTTCT
4 MT492030.1     ATTTAATTTAAAGCTTATCCCTTAAAATATTAAAATTTATTTTAAATTAATTAATTAATTAATTAAATTTATAAATAAATTTAAAATTAAATTTTTTTCT
5 MT492031.1     ATTTAATTTAAAGCTTATCCCTTAAAATATTAAAATTTATTTTAAATTAATTAATTAATTAATTAAATTTATAAATAAATTTAAAATTAAATTTTTTTCT
6 MT492032.1     ATTTAATTTAAAGCTTATCCCTTAAAATATTAAAATTTATTTTAAATTAATTAATTAATTAATTAAATTTATAAATAAATTTAAAATTAAATTTTTTTCT
7 MT492033.1     ATTTAATTTAAAGCTTATCCCTTAAAATATTAAAATTTATTTTAAATTAATTAATTAATTAATTAAATTTATAAATAAATTTAAAATTAAATTTTTTTCT
8 MT492034.1     ATTTAATTTAAAGCTTATCCCTTAAAATATTAAAATTTATTTTAAATTAATTAATTAATTAATTAAATTTATAAATAAATTTAAAATTAAATTTTTTTCT
9 MT492035.1     ATTTAATTTAAAGCTTATCCCTTAAAATATTAAAATTTATTTTAAATTAATTAATTAATTAATTAAATTTATAAATAAATTTAAAATTAAATTTTTTTCT
                 taaattaaatttcgaatagggaattttataattttaaataaaatttaattaattaattaattaatttaaatatttatttaaattttaatttaaaaaaaga
```

```
           14001         .         .         .         .         :         .         .         .         .         1 14100
1 MN793322.1     AAAAAAACTAGATATATTTTAAAACG-ATTAACATTTCATTTCTAATTAATTATTTAAAATATTTATGAAACAATAACTTTAATAATTAATTAACTCTTT
2 MT492036.1     AAAAAAACTAGATATATTTTAAAACGGATTAACATTTCATTTCTAATTAATTATTTAAAATATTTATGAAGCAATAACCTTAATAATTAATTAACTCTTT
3 MT492037.1     AAAAAAACTAGATATATTTTAAAACGGATTAACATTTCATTTCTAATTAATTATTTAAAATATTTATGAAGCAATAACCTTAATAATTAATTAACTCTTT
4 MT492030.1     AAAAAAACTAGATATATTTTAAAACGGATTAACATTTCATTTCTAATTAATTATTTAAAATATTTATGAAACAATAACCTTAATAATTAATTAACTCTTT
5 MT492031.1     AAAAAAACTAGATATATTTTAAAACG-ATTAACATTTCATTTCTAATTAATTATTTAAAATATTTATGAAGCAATAACCTTAATAATTAATTAACTCTTT
6 MT492032.1     AAAAAAACTAGATATATTTTAAAACGGATTAACATTTCATTTCTAATTAATTATTTAAAATATTTATGAAGCAATAACCTTAATAATTAATTAACTCTTT
7 MT492033.1     AAAAAAACTAGATATATTTTAAAACGGATTAACATTTCATTTCTAATTAATTATTTAAAATATTTATGAAGCAATAACCTTAATAATTAATTAACTCTTT
8 MT492034.1     AAAAAAACTAGATATATTTTAAAACGGATTAACATTTCATTTCTAATTAATTATTTAAAATATTTATGAAGCAATAACCTTAATAATTAATTAACTCTTT
9 MT492035.1     AAAAAAACTAGATATATTTTAAAACGGATTAACATTTCATTTCTAATTAATTATTTAAAATATTTATGAAACAATAACCTTAATAATTAATTAACTCTTT
                 tttttttgatctatataaaattttgcctaattgtaaagtaaagattaattaataaattttataaatactttgttattggaattattaattaattgagaaa

           14101         .         .         .         .         :         .         .         .         .         2 14200
1 MN793322.1     AAAATTCGAGAATTTTTATCTATAAAAATTTAATTTAATAAACTCTGATACACAAGATACAATAAATAATATTTACTTTAATATAAATATAATTTCAATA
2 MT492036.1     AAAATTCGAGAATTTTTATCTATAAAAATTTAATTTAATAAACTCTGATACACAAGATACAATAAATAATATTTACTTTAATATAAATATAATTTCAATA
3 MT492037.1     AAAATTCGAGAATTTTTTTCTATAAAAATTTAATTTAATAAACTCTGATACACAAGATACAATAAATAATATTTACTTTAATATAAATATAATTTCAATA
4 MT492030.1     AAAATTCGAGAATTTTTATCTATAAAAATTTAATTTAATAAACTCTGATACACAAGATACAATAAATAATATTTACTTTAATATAAATATAATTTCAATA
5 MT492031.1     AAAATTCGAGAATTTTTTTCTATAAAAATTTAATTTAATAAACTCTGATACACAAGATACAATAAATAATATTTACTTTAATATAAATATAATTTCAATA
6 MT492032.1     AAAATTCGAGAATTTTTTTCTATAAAAATTTAATTTAATAAACTCTGATACACAAGATACAATAAATAATATTTACTTTAATATAAATATAATTTCAATA
7 MT492033.1     AAAATTCGAGAATTTTTTTCTATAAAAATTTAATTTAATAAACTCTGATACACAAGATACAATAAATAATATTTACTTTAATATAAATATAATTTCAATA
8 MT492034.1     AAAATTCGAGAATTTTTTTCTATAAAAATTTAATTTAATAAACTCTGATACACAAGATACAATAAATAATATTTACTTTAATATAAATATAATTTCAATA
9 MT492035.1     AAAATTCGAGAATTTTTATCTATAAAAATTTAATTTAATAAACTCTGATACACAAGATACAATAAATAATATTTACTTTAATATAAATATAATTTCAATA
                 ttttaagctcttaaaaatagatatttttaaattaaattatttgagactatgtgttctatgttatttattataaatgaaattatatttatattaaagttat

           14201         .         .         .         .         :         .         .         .         .         3 14300
1 MN793322.1     TATTTTAAATTTCTTATACAATACTAATTAACTATAAAATTAATAATTTTTTCTTTATTAATACTAAAACCCCCCAATTTTAATATTAAAATTAATTTTA
2 MT492036.1     TATTTTAAATTTCTTATCCAATACTAATTAACTATAAAATTAATAATTTTTTCTTTATTAATACTAAAACCCCCCAATTTTAATATTAAAATTAATTTTA
3 MT492037.1     TATTTTAAATTTCTTATCCAATACTAATTAACTATAAAATTAATAATTTTTTCTTTATTAATACTAAAACCCCCCAATTTTAATATTAAAATTAATTTTA
4 MT492030.1     TATTTTAAATTTCTTATCCAATACTAATTAACTATAAAATTAATAATTTTTTCTTTATTAATACTAAAACCCCCCAATTTTAATATTAAAATTAATTTTA
5 MT492031.1     TATTTTAAATTTCTTATACAATACTAATTAACTATAAAATTAATAATTTTTTCTTTATTAATACTAAAACCCCCCAATTTTAATATTAAAATTAATTTTA
6 MT492032.1     TATTTTAAATTTCTTATCCAATACTAATTAACTATAAAATTAATAATTTTTTCTTTATTAATACTAAAACCCCCCAATTTTAATATTAAAATTAATTTTA
7 MT492033.1     TATTTTAAATTTCTTATACAATACTAATTAACTATAAAATTAATAATTTTTTCTTTATTAATACTAAAACCCCCCAATTTTAATATTAAAATTAATTTTA
8 MT492034.1     TATTTTAAATTTCTTATACAATACTAATTAACTATAAAATTAATAATTTTTTCTTTATTAATACTAAAACCCCCCAATTTTAATATTAAAATTAATTTTA
9 MT492035.1     TATTTTAAATTTCTTATACAATACTAATTAACTATAAAATTAATAATTTTTTCTTTATTAATACTAAAACCCCCCAATTTTAATATTAAAATTAATTTTA
                 ataaaatttaaagaataggttatgattaattgatattttaattattaaaaaagaaataattatgattttggggggttaaaattataattttaattaaaat

           14301         .         .         .         .         :         .         .         .         .         4 14400
1 MN793322.1     TTATTTTTTAATTATTAATTTTTACCCCTCAAATTAATTGAAAATTTCAATATCATTTCAATGTAAATGAAATACTTTATCAAGCTCTAATTTGTTATTT
2 MT492036.1     TTATTTTTTAATTATTAATTTTTACCCCTCAAATTAATTGAAAATTTCAATATCATTTCAATGTAAATGAAATACTTTATCAAGCTCTAATTTGTTATTT
3 MT492037.1     TTATTTTTTAATTATTAATTTTTACCCCTCAAATTAATTGAAAATTTCAATATCATTTCAATGTAAATGAAATACTTTATCAAGCTCTAATTTGTTATTT
4 MT492030.1     TTATTTTTTAATTATTAATTTTTACCCCTCAAATTAATTGAAAATTTCAATATCATTTCAATGTAAATGAAATACTTTATCAAGCTCTAATTTGTTATTT
5 MT492031.1     TTATTTTTTAATTATTAATTTTTACCCCTCAAATTAATTGAAAATTTCAATATCATTTCAATGTAAATGAAATACTTTATCAAGCTCTAATTTGTTATTT
6 MT492032.1     TTATTTTTTAATTATTAATTTTTACCCCTCAAATTAATTGAAAATTTCAATATCATTTCAATGTAAATGAAATACTTTATCAAGCTCTAATTTGTTATTT
7 MT492033.1     TTATTTTTTAATTATTAATTTTTACCCCTCAAATTAATTGAAAATTTCAATATCATTTCAATGTAAATGAAATACTTTATCAAGCTCTAATTTGTTATTT
8 MT492034.1     TTATTTTTTAATTATTAATTTTTACCCCTCAAATTAATTGAAAATTTCAATATCATTTCAATGTAAATGAAATACTTTATCAAGCTCTAATTTGTTATTT
9 MT492035.1     TTATTTTTTAATTATTAATTTTTACCCCTCAAATTAATTGAAAATTTCAATATCATTTCAATGTAAATGAAATACTTTATCAAGCTCTAATTTGTTATTT
                 aataaaaaattaataattaaaaatggggagtttaattaacttttaaagttatagtaaagttacatttactttatgaaatagttcgagattaaacaataaa
                                <- rRNA-LSU | tRNA-Val (UAC) ->                                      |
```

```
           14401         .         .         .         .         :         .         .         .         .        5 14500
1 MN793322.1     CTAGAAACACTTTCCAGTACCTCTACTTTGTTACGACTTATTCCAATTTATTTATGAAAGCGACGGGCAATATGTACATATTTTAATATATAATCATTTT
2 MT492036.1     CTAGAAACACTTTCCAGTACCTCTACTTTGTTACGACTTATTCCAATTTATTTATGAAAGCGACGGGCAATATGTACATATTTTAATATATAATCATTTT
3 MT492037.1     CTAGAAACACTTTCCAGTACCTCTACTTTGTTACGACTTATTCCAATTTATTTATGAAAGCGACGGGCAATATGTACATATTTTAATATATAATCATTTT
4 MT492030.1     CTAGAAACACTTTCCAGTACCTCTACTTTGTTACGACTTATTCCAATTTATTTATGAAAGCGACGGGCAATATGTACATATTTTAATATATAATCATTTT
5 MT492031.1     CTAGAAACACTTTCCAGTACCTCTACTTTGTTACGACTTATTCCAATTTATTTATGAAAGCGACGGGCAATATGTACATATTTTAATATATAATCATTTT
6 MT492032.1     CTAGAAACACTTTCCAGTACCTCTACTTTGTTACGACTTATTCCAATTTATTTATGAAAGCGACGGGCAATATGTACATATTTTAATATATAATCATTTT
7 MT492033.1     CTAGAAACACTTTCCAGTACCTCTACTTTGTTACGACTTATTCCAATTTATTTATGAAAGCGACGGGCAATATGTACATATTTTAATATATAATCATTTT
8 MT492034.1     CTAGAAACACTTTCCAGTACCTCTACTTTGTTACGACTTATTCCAATTTATTTATGAAAGCGACGGGCAATATGTACATATTTTAATATATAATCATTTT
9 MT492035.1     CTAGAAACACTTTCCAGTACCTCTACTTTGTTACGACTTATTCCAATTTATTTATGAAAGCGACGGGCAATATGTACATATTTTAATATATAATCATTTT
                 gatctttgtgaaaggtcatggagatgaaacaatgctgaataaggttaaataaatactttcgctgcccgttatacatgtataaaattatatattagtaaaa

           14501         .         .         .         .         :         .         .         .         .        6 14600
1 MN793322.1     ATTAAAATAAATAAAATTACATTTAAATCCACTTTCAAATTTTTATTACAAAAAAAAAATCCATTTAAATAAATTTATTGTAATCCATTATATTCTTAAT
2 MT492036.1     ATTAAAATAAATAAAATTACATTTAAATCCACTTTCAAATTTTTATTACAAAAAAAAA-TCCATTTAAATAAATTTATTGTAATCCATTATATTCTTAAT
3 MT492037.1     ATTAAAATAAATAAAATTACATTTAAATCCACTTTCAAATTTTTATTACAAAAAAAAA-TCCATTTAAATAAATTTATTGTAATCCATTATATTCTTAAT
4 MT492030.1     ATTAAAATAAATAAAATTACATTTAAATCCACAAACAAATTTTTATTACAAAAAAAAA-TCCATTTAAATAAATTTATTGTAATCCATTATATTCTTAAT
5 MT492031.1     ATTAAAATAAATAAAATTACATTTAAATCCACTTTCAAATTTTTATTACAAAAAAAAA-TCCATTTAAATAAATTTATTGTAATCCATTATATTCTTAAT
6 MT492032.1     ATTAAAATAAATAAAATTACATTTAAATCCACAAACACATTTTTATTACAAAAAAAAA-TCCATTTAAATAAATTTATTGTAATCCATTATATTCTTAAT
7 MT492033.1     ATTAAAATAAATAAAATTACATTTAAATCCACTTTCAAATTTTTATTACAAAAAAAAA-TCCATTTAAATAAATTTATTGTAATCCATTATATTCTTAAT
8 MT492034.1     ATTAAAATAAATAAAATTACATTTAAATCCACTTTCACATTTTTATTACAAAAAAAAA-TCCATTTAAATAAATTTATTGTAATCCATTATATTCTTAAT
9 MT492035.1     ATTAAAATAAATAAAATTACATTTAAATCCACTTTCAAATTTTTATTACAAAAAAAAA-TCCATTTAAATAAATTTATTGTAATCCATTATATTCTTAAT
                 Taattttatttattttaatgtaaatttaggtgtttgtgtaaaaataatgttttttttt-aggtaaatttatttaaataacattaggtaatataagaatta

           14601         .         .         .         .         :         .         .         .         .        7 14700
1 MN793322.1     TATAATCTGCATCTTGATCTGATTTAATTTTTAATTATAAAATTAAAATATTATTTTCATTTAAAAATATTTTTTTAACAACGATATACAAAATTTTAAA
2 MT492036.1     TATAATCTGCATCTTGATCTGATTTAATTTTTAATTATAAAATTAAAATATTATTTTCATTTAAAAATATTTTTTTAACAACGATATACAAAATTTTAAA
3 MT492037.1     TATAATCTGCATCTTGATCTGATTTAATTTTTAATTATAAAATTAAAATATTATTTTCATTTAAAAATATTTTTTTAACAACGATATACAAAATTTTAAA
4 MT492030.1     TATAATCTGCATCTTGATCTGATTTAATTTTTAATTATAAAATTAAAATATTATTTTCATTTAAAAATATTTTTTTAACAACGATATACAAAATTTTAAA
5 MT492031.1     TATAATCTGCATCTTGATCTGATTTAATTTTTAATTATAAAATTAAAATATTATTTTCATTTAAAAATATTTTTTTAACAACGATATACAAAATTTTAAA
6 MT492032.1     TATAATCTGCATCTTGATCTGATTTAATTTTTAATTATAAAATTAAAATATTATTTTCATTTAAAAATATTTTTTTAACAACGATATACAAAATTTTAAA
7 MT492033.1     TATAATCTGCATCTTGATCTGATTTAATTTTTAATTATAAAATTAAAATATTATTTTCATTTAAAAATATTTTTTTAACAACGATATACAAAATTTTAAA
8 MT492034.1     TATAATCTGCATCTTGATCTGATTTAATTTTTAATTATAAAATTAAAATATTATTTTCATTTAAAAATATTTTTTTAACAACGATATACAAAATTTTAAA
9 MT492035.1     TATAATCTGCATCTTGATCTGATTTAATTTTTAATTATAAAATTAAAATATTATTTTCATTTAAAAATATTTTTTTAACAACGATATACAAAATTTTAAA
                 Atattagacgtagaactagactaaattaaaaattaatattttaattttataataaaagtaaatttttataaaaaaattgttgctatatgttttaaaattt

           14701         .         .         .         .         :         .         .         .         .        8 14800
1 MN793322.1     TTAAGTAAATTCATTCGTGGATTATCAATTAATAAACAGATTCCTCTAAATGAACTAAAATACCGCCAAATTATTTAAGTTTCAATAAATAATTATATAC
2 MT492036.1     TTAAGTAAATTCATTCGTGGATTATCAATTAATAAACAGATTCCTCTAAATGAACTAAAATACCGCCAAATTATTTAAGTTTCAATAAATAATTATATAC
3 MT492037.1     TTAAGTAAATTCATTCGTGGATTATCAATTAATAAACAGATTCCTCTAAATGAACTAAAATACCGCCAAATTATTTAAGTTTCAATAAATAATTATATAC
4 MT492030.1     TTAAGTAAATTCATTCGTGGATTATCAATTAATAAACAGATTCCTCTAAATGAACTAAAATACCGCCAAATTATTTAAGTTTCAATAAATAATTATATAC
5 MT492031.1     TTAAGTAAATTCATTCGTGGATTATCAATTAATAAACAGATTCCTCTAAATGAACTAAAATACCGCCAAATTATTTAAGTTTCAATAAATAATTATATAC
6 MT492032.1     TTAAGTAAATTCATTCGTGGATTATCAATTAATAAACAGATTCCTCTAAATGAACTAAAATACCGCCAAATTATTTAAGTTTCAATAAATAATTATATAC
7 MT492033.1     TTAAGTAAATTCATTCGTGGATTATCAATTAATAAACAGATTCCTCTAAATGAACTAAAATACCGCCAAATTATTTAAGTTTCAATAAATAATTATATAC
8 MT492034.1     TTAAGTAAATTCATTCGTGGATTATCAATTAATAAACAGATTCCTCTAAATGAACTAAAATACCGCCAAATTATTTAAGTTTCAATAAATAATTATATAC
9 MT492035.1     TTAAGTAAATTCATTCGTGGATTATCAATTAATAAACAGATTCCTCTAAATGAACTAAAATACCGCCAAATTATTTAAGTTTCAATAAATAATTATATAC
                 aattcatttaagtaagcacctaatagttaattatttgtctaaggagatttacttgattttatggcggtttaataaattcaaagttatttattaatatatg
```

```
           14801         .         .         .         .         :         .         .         .         .        9 14900
1 MN793322.1     TATTTTAGTATTTTAAATTTAAATTTTTAATAATAGGGTATCTAATCCTAGTTTATAATATAAATTTATTAAATTATAAAATTAAATTAAATTAAATTAA
2 MT492036.1     TATTTTAGTATTTTAAATTTAAATTTTTAATAATAGGGTATCTAATCCTAGTTTATAATATAAATTTATTAAATTATAAAATTAAATTAAATTAAATTAA
3 MT492037.1     TATTTTAGTATTTTAAATTTAAATTTTTAATAATAGGGTATCTAATCCTAGTTTATAATATAAATTTATTAAATTATAAAATTAAATTAAATTAAATTAA
4 MT492030.1     TATTTTAGTATTTTAAATTTATATTATAAACTAGGATTAGATACCCTATTATTAAAAATTTAAATTTAAAATACTAAAATAGTATATAATTATTTAAGTT
5 MT492031.1     TATTTTAGTATTTTAAATTTAAATTTTTA-----------------------------------------------------------------------
6 MT492032.1     TATTTTAGTATTTTAAATTTAAATTTTTA-----------------------------------------------------------------------
7 MT492033.1     TATTTTAGTATTTTAAATTTAAATTTTTA-----------------------------------------------------------------------
8 MT492034.1     TATTTTAGTATTTTAAATTTAAATTTTTA-----------------------------------------------------------------------
9 MT492035.1     TATTTTAGTATTTTAAATTTAAATTTTTAATAATAGGGTATCTAATCCTAGTTTATAATATAAATTTATTAAATTATAAAATTAAATTAAATTAAATTAA
                 ataaaatcataaaatttaaatttaaaaat
                                 <- rRNA-SSU |

           14901         .         .         .         .         :         .         .         .         .        0 15000
1 MN793322.1     ATAAAAATTTCACCTATTAATTTAATATTTAATTAAATAAAATTTTTATAAATTAATTACTAATAAAATTTAATTTAATTTTTGTATAACCGCAACTGCT
2 MT492036.1     ATAAAAATTTCACCTATTAATTTAATATTTAATTAAATAAAATTTTTATAAATTAATTACTAATAAAATTTAATTTAATTTTTGTATAACCGCAACTGCT
3 MT492037.1     ATAAAAATTTCACCTATTAATTTAATATTTAATTAAATAAAATTTTTATAAATTAATTACTAATAAAATTTAATTTAATTTTTGTATAACCGCAACTGCT
4 MT492030.1     TCAATAAATAATTATATACTATTTTAGTATTTTAAATTTAAA----------------------------------------------------------
5 MT492031.1     ----------------------------------------------------------------------------------------------------
6 MT492032.1     ----------------------------------------------------------------------------------------------------
7 MT492033.1     ----------------------------------------------------------------------------------------------------
8 MT492034.1     ----------------------------------------------------------------------------------------------------
9 MT492035.1     ATAAAAATTTCACCTATTAATTTAATATTTAATTAAATAAAATTTTTATAAATTAATTACTAATAAAATTTAATTTAATTTTTGTATAACCGCAACTGCT

           15001         .         .         .         .         :         .         .         .         .        1 15100
1 MN793322.1     GGCACAAAATTTGTTATTAATTTAAATATTACTAAATCTTAATTTCTTAAATTTTTAATATTAATTGCTATATATTTTAAAATTCAAAAATTATTAAAAT
2 MT492036.1     GGC-------------------------------------------------------------------------------------------------
3 MT492037.1     GGCACAAAATTTGTTATTAATTTAAATATTACTAAATCTTAATTTCTTAAATTTTTAATATTAATTGCTATATATTTTAAAATTCAAAAATTATTAAAAT
4 MT492030.1     ----------------------------------------------------------------------------------------------------
5 MT492031.1     ----------------------------------------------------------------------------------------------------
6 MT492032.1     ----------------------------------------------------------------------------------------------------
7 MT492033.1     ----------------------------------------------------------------------------------------------------
8 MT492034.1     ----------------------------------------------------------------------------------------------------
9 MT492035.1     GGCACAAAATTTGTTATTAATTTAAATATTACTAAATCTTAATTTCTTAAATTTTTAATATTAATTGCTATATATTTTAAAATTCAAAAATTATTAAAAT

           15101         .         .         .         .         :         .         .         .         .        2 15200
1 MN793322.1     AATGAAAAATTAACACTAAAATTTATATGCAAAATAAATTTATAATAAATTTATAAACTATAAAAAATTTATTTAATACTTAAAATTTTTAACTTAGATT
2 MT492036.1     ----------------------------------------------------------------------------------------------------
3 MT492037.1     AATGAAAAATTAACACTAAAATTTATATGCAAAATAAATTTATAATAAATTTATAAA-------------------------------------------
4 MT492030.1     ----------------------------------------------------------------------------------------------------
5 MT492031.1     ----------------------------------------------------------------------------------------------------
6 MT492032.1     ----------------------------------------------------------------------------------------------------
7 MT492033.1     ----------------------------------------------------------------------------------------------------
8 MT492034.1     ----------------------------------------------------------------------------------------------------
9 MT492035.1     AATGAAAAATTAACACTAAAATTTATATGCAAAATAAATTTATAATAAATTTATAAACTATAAAAAATTTATTTAATACTTAAAATTTTTAAATTAGATT
```

```
            15201         .         .         .         .         :         .         .         .         .        3 15300
1 MN793322.1     TTTTTTTTTTTTTTTT-ATATTAAATATTTAATATAAATTATTAAATTTTAAATATTTATTTCTTTTTCTTTTTCATAATATTAATATTAAAAATTAATA
2 MT492036.1     ----------------------------------------------------------------------------------------------------
3 MT492037.1     ----------------------------------------------------------------------------------------------------
4 MT492030.1     ----------------------------------------------------------------------------------------------------
5 MT492031.1     ----------------------------------------------------------------------------------------------------
6 MT492032.1     ----------------------------------------------------------------------------------------------------
7 MT492033.1     ----------------------------------------------------------------------------------------------------
8 MT492034.1     ----------------------------------------------------------------------------------------------------
9 MT492035.1     TTTTTTTTTTTTTTTTTATATTAAATATTTAATATAAATTATTAAATTTTAAAT----------------------------------------------


            15301         .         .         .         .         :         .         .         .         .        4 15400
1 MN793322.1     TCATTATACAGCGATTTATAATCATTGAAATAAATAATTAATTATTAAGTTTAATAATTAATTAAATTGATTTATTAATATATTATAAATATAATTTCTA
2 MT492036.1     ----------------------------------------------------------------------------------------------------
3 MT492037.1     ----------------------------------------------------------------------------------------------------
4 MT492030.1     ----------------------------------------------------------------------------------------------------
5 MT492031.1     ----------------------------------------------------------------------------------------------------
6 MT492032.1     ----------------------------------------------------------------------------------------------------
7 MT492033.1     ----------------------------------------------------------------------------------------------------
8 MT492034.1     ----------------------------------------------------------------------------------------------------
9 MT492035.1     ----------------------------------------------------------------------------------------------------


            15401         .         .         .         .         :         .         .         .         .        5 15500
1 MN793322.1     TTATATATATATATATATATTAAATATAATAATTAATTAAATATTTAATATATATATATATATATATATATAAACCATTCCTAATTTTTTTTCTTTTAAT
2 MT492036.1     ----------------------------------------------------------------------------------------------------
3 MT492037.1     ----------------------------------------------------------------------------------------------------
4 MT492030.1     ----------------------------------------------------------------------------------------------------
5 MT492031.1     ----------------------------------------------------------------------------------------------------
6 MT492032.1     ----------------------------------------------------------------------------------------------------
7 MT492033.1     ----------------------------------------------------------------------------------------------------
8 MT492034.1     ----------------------------------------------------------------------------------------------------
9 MT492035.1     ----------------------------------------------------------------------------------------------------


            15501      ] 15506
1 MN793322.1     AATAAA
2 MT492036.1     ------
3 MT492037.1     ------
4 MT492030.1     ------
5 MT492031.1     ------
6 MT492032.1     ------
7 MT492033.1     ------
8 MT492034.1     ------
9 MT492035.1     ------
```

Figure S4: Mean and distribution of normalized read depths (reads per kilo base per million mapped reads; RPKM) estimated among contigs of putative mitochondrial origins obtained from *de novo* libraries from mitochondrion-enriched and unenriched samples. Enriched sampled had significantly higher read depth than unenriched samples ($t = 14.435$; $df = 1, 667$; $p < 2.2 \times 10^{-16}$).

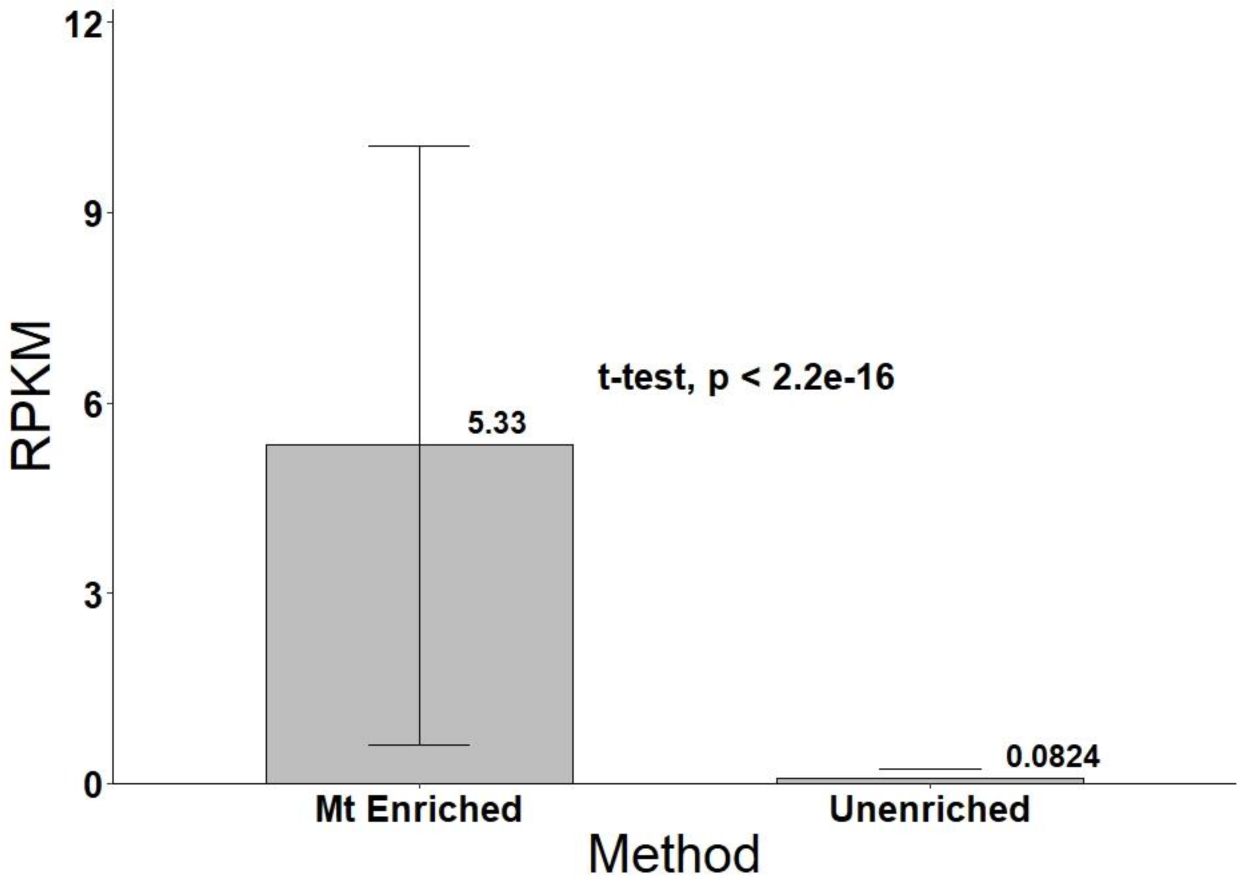

Figure S5: Normalized read depths (reads per kilo base per million mapped reads; RPKM) among *de novo* assembled contigs from libraries constructed from enriched (circles) and unenriched (triangles) extraction methods. Contigs corresponding to those of mitochondrial genome origin (red) are as defined by homology to the *Ostrinia nubilalis* reference AF4429573.1. Contigs of unknown origin are also shown (grey). Contigs within the unenriched libraries with lengths > 16,000 bp and RPKM > 16 are not shown.

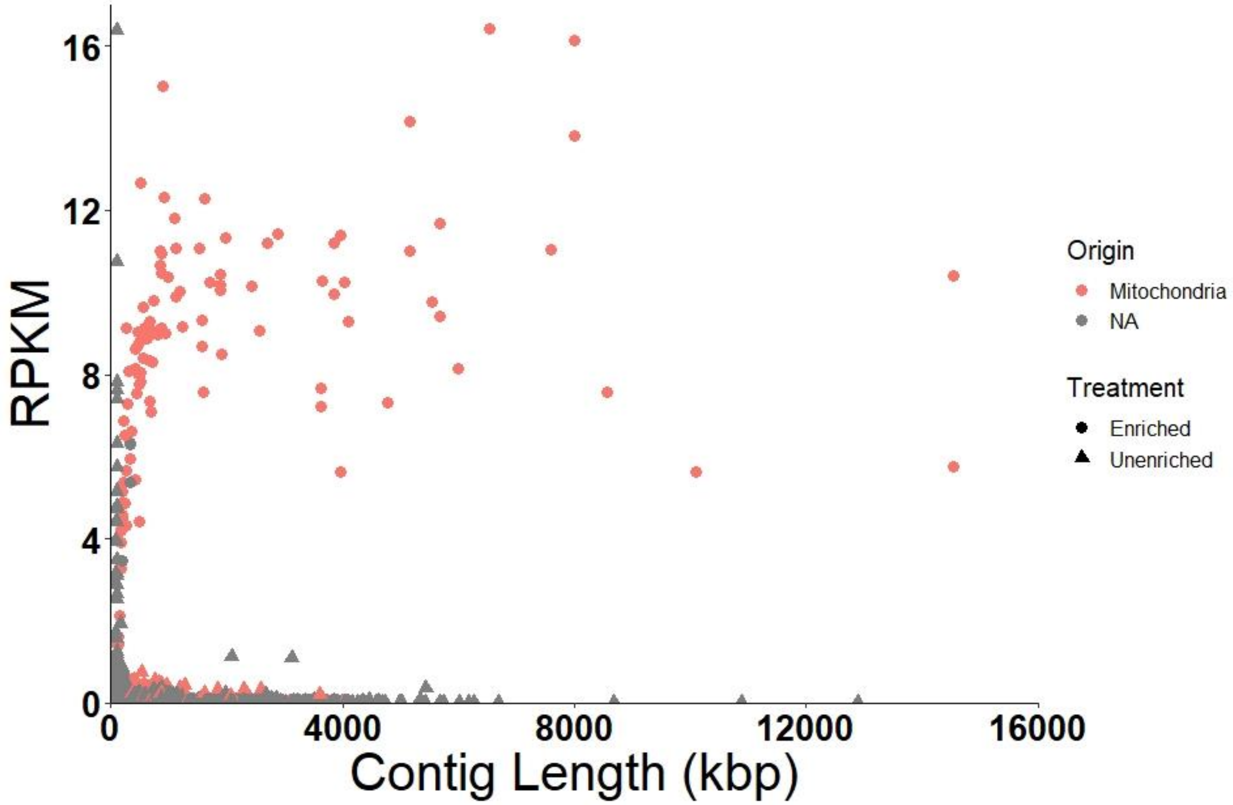

Table S1. Quantification of mitochondrion enrichment at optimized parameters by comparing real-time quantitative PCR (qPCR) C_T Values obtained with *apn*1 nuclear primers and *cox*I mitochondrial primers against nuclear fraction, mitochondrial fraction, and unenriched control templates. Additional quantification of mitochondrion enrichment at optimized parameters was estimates with high-throughput read alignment rates to a mitochondrial reference genome (AF442957.1) of enriched and unenriched samples (accessions MT492030.1-MT492037.1; SRX2498822-SRX2498825). Fold enrichment was calculated for each enriched sample with both quantification methods.

		Real-time qPCR C_T Values									Read Alignment			
		Nuclear Fraction				Mitochondrial Fraction				Fold		Aligned to	Alignment	Fold
Treatment	Sample	*apn*1	*apn*1	*cox*I	*cox*I	*apn*1	*apn*1	*cox*I	*cox*I	Enrichment	Total Reads	AF442957.1	Rate	Enrichment
Centrifugation	ID_02	25.37	24.35	18.77	18.16	29.81	29.25	14.52	14.69	59.30	2,784,696	632,453	23.7%	42.8
Centrifugation	ID_03	24.54	25.70	17.31	17.20	29.18	29.35	14.34	13.81	71.26	3,542,792	1,033,136	30.5%	55.0
Centrifugation	ID_04	24.33	24.41	17.83	17.41	30.51	28.69	14.68	14.91	54.57	3,556,284	918,312	27.1%	48.7
Centrifugation	ID_06	25.54	27.34	No Ct	17.36	30.44	31.05	15.14	14.86	104.69	2,542,850	806,607	33.0%	59.8
Centrifugation	ID_07	23.71	23.00	16.55	16.76	31.35	29.97	15.13	15.22	87.43	3,571,772	1,092,603	32.0%	57.7
Centrifugation	ID_11	24.72	24.05	17.97	17.84	30.68	30.08	15.16	14.74	84.16	3,021,608	632,342	21.9%	39.5
Centrifugation	ID_12	23.14	23.30	17.32	16.03	30.98	30.86	15.11	15.00	113.77	2,446,844	673,965	28.7%	52.0
Centrifugation	ID_18	24.90	No Ct	17.64	17.26	30.18	31.58	15.09	14.94	113.77	2,909,350	460,181	16.5%	29.8
		*apn*1	*apn*1	*cox*I	*cox*I									
Control	SDSS ♀1	26.68	27.09	18.32	18.15									
Control	SDSS ♀2	26.08	26.45	16.63	17.06									
Control	LVE										32,150,000	184,088	0.6%	
Control	LVZ										24,708,000	169,132	0.7%	
Control	RSE										47,636,000	274,872	0.6%	
Control	RSZ										36,782,000	105,302	0.3%	

Table S2. *Ostrinia nubilalis* mitochondrial genome sequence variation predicted from alignment of novel assemblies (MT492030.1 to MT492037.1) compared to the reference (Ref) AF442957.1 accession (RefSeq NC_003367.1). Position of alternate (Alt) single nucleotide variants (SNV; *n* = 73), insertion (*n* = 5), and deletion variations (*n* = 4) are with respect to the Ref, with each Alt nucleotide (A, T, G, or C) at each SNV, insertion, or deletion position provided. Predicted amino acid (AA) changes are shown for protein coding sequences, with those resulting in AA change (nonsynonymous; nonsyn) or no change (synonymous; syn) are indicated. Two insertion and deletion mutations resulting in a single frameshift in the *cox*I gene are indicated by an asterisk with codon-wise sequences CATATT (HI) and ATATTA (ML) listed, with alignment depicting predicted frameshift provided in Fig. 3B). # indicates the predicted deletion within *nd*4 within our assemblies MT492030.1 to MT492037.1 compared to the reference, and shown to be conserved among *Ostrinia* and other species of Lepidoptera (Fig. 4) and putatively resulting from Sanger sequencing error in AF442957.1. Positions marked with a plus (+) are mutations previously described by Coates et al. (2004), wherein substitutions also marked with H or S were validated using *Hae*III and *Sau*3AI PCR-RFLP assays, respectively.

	Variant				Ref		Alt		Change	
Feature	Position	Type	Ref	Alt	Codon	AA	Codon	AA	Type	Freq
tRNA^{Gln}	160	SNV	C	A						0.88
*nd*2	469	SNV	A	T	TTA	L	TTT	F	Nonsyn	0.88
*cox*I	1,462	SNV	C	A	ACC	T	ACA	T	Syn	1.00
*cox*I	2,047+	SNV	C	T	GCC	A	GCT	A	Syn	1.00
*cox*I	2,048	SNV	T	A	TTC	F	ATC	I	Nonsyn	0.88
*cox*I	2,060	SNV	AC	TT	ACA	T	TTA	L	Nonsyn	1.00
*cox*I	2,149	SNV	T	A	GGT	G	GGA	G	Syn	0.88
*cox*I	2,161+	SNV	G	T	GTG	V	GTT	V	Syn	1.00
*cox*I	2,196	Deletion*	A	-	TCC	S	TCA	S	Syn	0.50
*cox*I	2197-2202	Frameshift	ATA TTA	CAT ATT	ATA TTA	ML	CAT ATT	HI	Nonsyn	0.50
*cox*I	2,205	Insertion*	-	A	TTA	I	ATT	L	Nonsyn	0.50
*cox*I	2,233	SNV	T	A	ACT	T	ACA	T	Syn	1.00
*cox*I	2,513+	SNV	C	T	CCA	P	TCA	S	Nonsyn	0.88
*cox*I	2,554+H	SNV	G	A	GTG	V	GTA	V	Syn	0.88
*cox*I	2,826+	SNV	G	T	GGG	G	GTG	V	Nonsyn	0.88
*cox*I	2,908	SNV	G	T	ATG	M	ATT	I	Nonsyn	0.88
*cox*I	2,922	SNV	TC	AT	ATC or ATT	I	AAT	N	Nonsyn	0.88
*cox*II	3,050+S	SNV	C	G	TCG	S	TGG	W	Nonsyn	0.75
*cox*II	3,207	SNV	A	T	CTA	L	CTT	L	Syn	0.75
*cox*II	3,237	SNV	T	A	ACT	T	ACA	T	Syn	0.75
*cox*II	3,243	SNV	A	T	ATG	M	ATT	I	Nonsyn	0.75
*cox*II	3,384	SNV	C	T	AAC	N	AAT	N	Syn	0.63

	Variant				Ref		Alt		Change	
Feature	Position	Type	Ref	Alt	Codon	AA	Codon	AA	Type	Freq
*cox*II	3,554+	SNV	G	T	GGA	G	GTA	V	Nonsyn	1.00
*cox*II	3,704	SNV	G	C	TGG	W	TCG	S	Nonsyn	0.13
tRNA^{Asp}	3,832	SNV	T	A						0.75
tRNA^{Asp}	3,842	SNV	T	A						0.13
*atp*8	3,953	Insertion	---	ATT	---	-	ATT	I		0.13
*atp*6	4,462	SNV	T	A	AAT	N	AAA	K	Nonsyn	0.13
*atp*6	4,464	SNV	T	G	ATT	I	AGT	S	Nonsyn	0.13
*atp*6	4,467	SNV	T	C	ATT	I	ACT	T	Nonsyn	0.13
*atp*6	4,470	SNV	G	C	CGA	R	CCA	P	Nonsyn	0.13
*atp*6	4,472	SNV	C	A	CCT	P	ACT	T	Nonsyn	0.13
*atp*6	4,610	SNV	C	C	CTA	L	TTA	L	Syn	0.13
*cox*III	4,789	SNV	G	A	GAA	E	AAA	K	Nonsyn	0.88
*cox*III	5,136	SNV	T	C	CAT	H	CAC	H	Syn	0.75
nd 3	5,856	SNV	G	A	GGG	G	GGA	G	Syn	0.38
tRNA^{Ala}	5,931	Insertion	A-	AA						0.88
tRNA^{Ala}	5,963	Insertion	A-	AA						0.88
tRNA^{Glu}	6,190	SNV	T	A						1.00
*nd*5	6,507	SNV	G	A	ACC	T	ACT	T	Syn	1.00
*nd*5<comp>	6,570	SNV	T	A	ATG	M	ATT	I	Nonsyn	1.00
*nd*5<comp>	6,579	SNV	G	A	ACC	T	ACT	T	Syn	1.00
*nd*5<comp>	6,588	SNV	T	A	TTA	L	TTT	F	Nonsyn	1.00
*nd*5<comp>	6,995	SNV	C	T	GCT	A	ACT	T	Nonsyn	0.38
*nd*5<comp>	7,046	SNV	T	C	ATT	I	GTT	V	Nonsyn	0.38
*nd*5<comp>	7,325	SNV	G	A	CTA	L	TTA	L	Syn	0.50
*nd*5<comp>	7,551	SNV	T	A	GGA	G	GGT	G	Syn	0.88
*nd*5<comp>	7,653	SNV	GAT	ATA	ATC	I	TAT	Y	Nonsyn	1.00
*nd*5<comp>	7,654	SNV	A	T	ATC	I	TAT	Y	Nonsyn	1.00
*nd*5<comp>	7,655	SNV	T	A	ATC	I	TAT	Y	Nonsyn	1.00
*nd*5<comp>	7,713	SNV	A	T	GTT	V	GTA	V	Syn	1.00
*nd*5<comp>	7,749	SNV	C	A	TTG	L	TTT	F	Nonsyn	0.88
*nd*5<comp>	7,757	SNV	C	A	GTT	V	TTT	F	Nonsyn	0.88
tRNA^{His}	8,060	SNV	T	C						0.13
*nd*4<comp>	8,194	Deletion#	G	-	TCA	S	GTA	V	Nonsyn	1.00
*nd*4<comp>	8,318	SNV	G	A	CCT	P	CTT	L	Nonsyn	0.75
*nd*4<comp>	8,415	SNV	C	A	TGG	W	TTG	L	Nonsyn	0.88
*nd*4L<comp>	9,604	SNV	C	S	GTA	V	TTA	L	Nonsyn	1.00

	Variant				Ref		Alt		Change	
Feature	Position	Type	Ref	Alt	Codon	AA	Codon	AA	Type	Freq
*nd*4L<comp>	9,605	SNV	ATA	CAT	TAT	Y	ATG	M	Nonsyn	1.00
cytb	10,511	SNV	G	A	CCG	P	CCA	P	Syn	0.88
cytb	10,517	SNV	C	A	CCC	P	CCA	P	Syn	0.50
cytb	10,601	SNV	C	A	ACC	T	ACA	T	Syn	0.75
cytb	11,315	SNV	G	T	GGG	G	GGT	G	Syn	0.63
*nd*1<comp>	11,700	SNV	C	A	ATG	M	ATT	I	Nonsyn	0.63
*nd*1<comp>	12,574	SNV	G	A	CCT	P	CTT	L	Nonsyn	1.00
rRNA^{LSU}	12,787	SNV	A	G						0.13
rRNA^{LSU}	12,788	SNV	A	T						0.13
rRNA^{LSU}	12,790	SNV	A	C						0.13
rRNA^{LSU}	12,795	SNV	A	G						0.13
rRNA^{LSU}	12,796	SNV	A	G						0.13
rRNA^{LSU}	12,799	SNV	A	T						0.13
rRNA^{LSU}	13,131	Insertion	-	T						0.88
rRNA^{LSU}	13,153	Deletion	A	-						0.75
rRNA^{LSU}	13,733	Deletion	G	-						0.25
rRNA^{LSU}	13,778	SNV	G	A						0.38
rRNA^{LSU}	13,825	SNV	T	A						0.38
rRNA^{LSU}	13,925	SNV	C	A						0.50
rRNA^{SSU}	14,240	SNV	A	T						0.75
rRNA^{SSU}	14,241	SNV	A	T						0.75
rRNA^{SSU}	14,242	SNV	A	T						0.75
rRNA^{SSU}	14,245	SNV	C	A						0.75
rRNA^{SSU}	14,463	SNV	G	A						1.00
rRNA^{SSU}	14,528	SNV	A	T						0.13

Table S3. Location of substitutions and indels within the *Ostrinia nubilalis* mitochondrial genome reference (AF442957.1) and *de novo* assembled mitochondrial genomes from eight F_1 families [F1 Family ID (accession MT49xxxx.1)].

		Position in assemblies from each F_1 Family							
Feature	Ref	ID_02 (2030.1)	ID_03 (2030.1)	ID_04 (2030.1)	ID_06 (2030.1)	ID_07 (2030.1)	ID_11 (2030.1)	ID_12 (2030.1)	ID_18 (2030.1)
tRNA^{Gln}	160	351	443	315	247	247	283	261	361
*nd*2	469	660	752	624	556	556	592	570	670
*cox*I	1,462	1,652	1,745	1,616	1,548	1,548	1,584	1,562	1,663
*cox*I	2,047	2,237	2,330	2,201	2,133	2,133	2,169	2,147	2,248
*cox*I	2,048	2,238	2,331	2,202	2,134	2,134	2,170	2,148	2,249
*cox*I	2,060	2,250	2,343	2,214	2,146	2,146	2,182	2,160	2,261
*cox*I	2,061	2,251	2,344	2,215	2,147	2,147	2,183	2,161	2,262
*cox*I	2,149	2,339	2,432	2,303	2,235	2,235	2,271	2,249	2,350
*cox*I	2,161	2,351	2,444	2,315	2,247	2,247	2,283	2,261	2,362
*cox*I	2,196	2,386	2,479	2,350	2,282	2,282	2,318	2,296	2,397
*cox*I	2,205	2,396	2,488	2,359	2,291	2,291	2,328	2,306	2,407
*cox*I	2,233	2,423	2,516	2,387	2,319	2,319	2,355	2,333	2,434
*cox*I	2,513	2,703	2,796	2,667	2,599	2,599	2,635	2,613	2,714
*cox*I	2,554	2,744	2,837	2,708	2,640	2,640	2,676	2,654	2,755
*cox*I	2,826	3,016	3,109	2,980	2,912	2,912	2,948	2,926	3,027
*cox*I	2,908	3,098	3,191	3,062	2,994	2,994	3,030	3,008	3,109
*cox*I	2,922	3,112	3,205	3,076	3,008	3,008	3,044	3,022	3,123
*cox*I	2,923	3,113	3,206	3,077	3,009	3,009	3,045	3,023	3,124
*cox*II	3,050	3,240	3,333	3,204	3,136	3,136	3,172	3,150	3,251
*cox*II	3,207	3,397	3,490	3,361	3,293	3,293	3,329	3,307	3,408
*cox*II	3,237	3,427	3,520	3,391	3,323	3,323	3,359	3,337	3,438
*cox*II	3,243	3,433	3,526	3,397	3,329	3,329	3,365	3,343	3,444
*cox*II	3,384	3,574	3,667	3,538	3,470	3,470	3,506	3,484	3,585
*cox*II	3,554	3,744	3,837	3,708	3,640	3,640	3,676	3,654	3,755
*cox*II	3,704	3,894	3,987	3,858	3,790	3,790	3,826	3,804	3,905
tRNA^{Asp}	3,832	4,022	4,115	3,986	3,918	3,918	3,954	3,932	4,033
tRNA^{Asp}	3,842	4,032	4,125	3,996	3,928	3,928	3,964	4,032	4,043
*atp*8	3,953	4,143	4,236	4,107	4,039	4,039	4,075	4,053	4,154
*atp*6	4,462	4,652	4,745	4,619	4,548	4,548	4,584	4,562	4,663
*atp*6	4,464	4,654	4,747	4,621	4,550	4,550	4,586	4,564	4,665
*atp*6	4,467	4,657	4,750	4,624	4,553	4,553	4,589	4,567	4,668
*atp*6	4,470	4,660	4,753	4,627	4,556	4,556	4,592	4,570	4,671
*atp*6	4,472	4,662	4,755	4,629	4,558	4,558	4,594	4,572	4,673
*atp*6	4,610	4,800	4,893	4,767	4,696	4,696	4,732	4,710	4,811
*cox*III	4,789	4,979	5,072	4,946	4,875	4,875	4,911	4,889	4,990
*cox*III	5,136	5,326	5,419	5,293	5,222	5,222	5,258	5,236	5,337
*nd*3	5,856	6,046	6,139	6,013	5,942	5,942	5,978	5,956	6,057
tRNA^{Ala}	5,931	6,121	6,214	6,088	6,017	6,017	6,053	6,031	6,132
tRNA^{Ala}	5,963	6,154	6,247	6,120	6,050	6,050	6,086	6,064	6,165
tRNA^{Glu}	6,190	6,381	6,474	6,347	6,277	6,277	6,313	6,291	6,392
*nd*5	6,507	6,698	6,791	6,664	6,594	6,594	6,630	6,608	6,709
*nd*5	6,570	6,761	6,854	6,727	6,657	6,657	6,693	6,671	6,772
*nd*5	6,579	6,770	6,863	6,736	6,666	6,666	6,702	6,680	6,781
*nd*5	6,588	6,779	6,872	6,745	6,675	6,675	6,711	6,689	6,790
*nd*5	6,995	7,186	7,279	7,152	7,082	7,082	7,118	7,096	7,197
*nd*5	7,046	7,237	7,330	7,203	7,133	7,133	7,169	7,147	7,248
*nd*5	7,325	7,516	7,609	7,482	7,412	7,412	7,448	7,426	7,527
*nd*5	7,551	7,742	7,835	7,708	7,638	7,638	7,674	7,652	7,753
*nd*5	7,653	7,844	7,937	7,810	7,740	7,740	7,776	7,754	7,855
*nd*5	7,654	7,845	7,938	7,811	7,741	7,741	7,777	7,755	7,856
*nd*5	7,655	7,846	7,939	7,812	7,742	7,742	7,778	7,756	7,857
*nd*5	7,713	7,904	7,997	7,870	7,800	7,800	7,836	7,814	7,915
*nd*5	7,749	7,940	8,033	7,906	7,836	7,836	7,872	7,850	7,951
*nd*5	7,757	7,948	8,041	7,914	7,844	7,844	7,880	7,858	7,959
tRNA^{His}	8,060	8,251	8,344	8,217	8,147	8,147	8,183	8,161	8,262
*nd*4	8,194	8,391	8,484	8,357	8,287	8,287	8,323	8,301	8,402

Feature	Ref	Position in assemblies from each F_1 Family							
		ID_02 (2030.1)	ID_03 (2030.1)	ID_04 (2030.1)	ID_06 (2030.1)	ID_07 (2030.1)	ID_11 (2030.1)	ID_12 (2030.1)	ID_18 (2030.1)
*nd*4	8,318	8,513	8,606	8,479	8,409	8,409	8,445	8,423	8,524
*nd*4	8,415	8,610	8,703	8,576	8,506	8,506	8,542	8,520	8,621
*nd*4L	9,604	9,604	9,887	9,760	9,690	9,690	9,726	9,704	9,805
*nd*4L	9,605	9,605	9,888	9,761	9,691	9,691	9,727	9,705	9,806
*nd*4L	9,606	9,606	9,889	9,762	9,692	9,692	9,728	9,706	9,807
*nd*4L	9,607	9,607	9,890	9,763	9,693	9,693	9,729	9,707	9,808
cytb	10,511	10,701	10,794	10,667	10,597	10,597	10,633	10,611	10,712
cytb	10,517	10,707	10,800	10,673	10,603	10,603	10,639	10,617	10,718
cytb	10,601	10,791	10,884	10,757	10,687	10,687	10,723	10,701	10,802
cytb	11,315	11,505	11,598	11,471	11,401	11,401	11,437	11,415	11,516
*nd*1	11,700	11,890	11,983	11,856	11,786	11,786	11,822	11,800	11,901
*nd*1	12,574	12,764	12,857	12,730	12,660	12,660	12,696	12,674	12,775
$rRNA^{LSU}$	12,787	12,977	13,070	12,961	12,891	12,891	12,927	12,905	13,006
$rRNA^{LSU}$	12,788	12,978	13,071	12,962	12,892	12,892	12,928	12,906	13,007
$rRNA^{LSU}$	12,790	12,980	13,073	12,964	12,894	12,894	12,930	12,908	13,009
$rRNA^{LSU}$	12,795	12,985	13,078	12,969	12,899	12,899	12,935	12,913	13,014
$rRNA^{LSU}$	12,796	12,986	13,079	12,970	12,900	12,900	12,936	12,914	13,015
$rRNA^{LSU}$	12,799	12,989	13,082	12,973	12,903	12,903	12,939	12,917	13,018
$rRNA^{LSU}$	13,131	13,321	13,414	13,305	13,235	13,235	13,271	13,249	13,350
$rRNA^{LSU}$	13,153	13,344	13,437	13,328	13,257	13,258	12,820	13,272	13,373
$rRNA^{LSU}$	13,733	13,923	14,015	13,907	13,837	13,838	13,873	13,851	13,952
$rRNA^{LSU}$	13,778	13,968	14,060	13,952	13,882	13,883	13,918	13,896	13,997
$rRNA^{LSU}$	13,825	14,015	14,107	13,999	13,929	13,930	13,965	13,943	14,044
$rRNA^{LSU}$	13,925	14,115	14,207	14,099	14,029	14,030	14,065	14,043	14,144
$rRNA^{SSU}$	14,240	14,430	14,522	14,396	14,326	14,327	14,362	14,340	14,441
$rRNA^{SSU}$	14,241	14,431	14,523	14,397	14,327	14,328	14,363	14,341	14,442
$rRNA^{SSU}$	14,242	14,432	14,524	14,398	14,328	14,329	14,364	14,342	14,443
$rRNA^{SSU}$	14,245	14,435	14,527	14,401	14,331	14,332	14,367	14,345	14,446
$rRNA^{SSU}$	14,463	14,653	14,745	14,619	14,549	14,550	14,585	14,563	14,664
$rRNA^{SSU}$	14,528	14,718	14,810	14,684	14,614	14,615	14,650	14,628	14,729

Table S4. Intraspecies sequence variation within *Ostrinia nubilalis* predicted from a multiple sequence alignment of full mitochondrial genome assemblies. Position (Pos) of single nucleotide variant (SNV; *n* = 66) and insertion/deletion variations (Indel; *n* = 13) are with respect to a consensus alignment between GenBank accession (Fig. S1). Predicted amino acid (AA) changes are shown for protein coding sequences (those on complementary strand are indicated <comp>, with those resulting in AA change (nonsynonymous; nonsyn) or no change (synonymous; syn) are indicated. The corresponding position for each alternate nucleotide (A, T, G, or C) at each SNV or Indel (-) position are provided following the forward slash (/) for corresponding accessions. Two indels resulting in a single frameshift mutation are shown are indicated by an asterisk (*), where affected sequences CATATT- and -ATATTA are demarcated as # and +, respectively. Positions (Pos) marked with a plus (+) were previously described by Coates et al. (2004).

	Variant		Codon (AA) change			GenBank nr nucleotide database accessions								
Feature	Pos	Type	Consensus	Alternate	Change	MN792233.1	MT492030.1	MT492031.1	MT492032.1	MT492033.1	MT492034.1	MT492035.1	MT492036.1	MT492037.1
tRNA^{Gln}	443	SNV	NA	NA		A/193	A/351	A/443	C/310	A/247	A/247	A/283	A/261	A/361
*nd*2	752	SNV	TTT (F)	TTA (L)	Nonsyn.	T/481	T/639	T/731	T/603	A/556	T/556	T/292	T/549	T/570
*nd*2	1241	SNV	TTA (L)	TTG (L)	Nonsyn.	G/991	A/1149	A/1241	A/1113	A/1045	A/1045	A/1081	A/1059	A/1159
Non-coding	1712	SNV	NA	NA		-/1462	-/1620	T/1712	-/1584	-/1516	-/1516	-/1552	-/1530	T/1630
*cox*I	2331	SNV	ATC (I)	TTC (F)	Nonsyn.	A/2080	A/2238	A/2331	A/2202	A/2134	T/2134	A/2170	A/2148	A/2149
*cox*I	2432	SNV	GGA (G)	GGT (G)	Syn.	A/2181	A/2339	A/2432	A/2303	A/2235	T/2235	A/2271	A/2249	A/2350
*cox*I	2480	indel*	TCA	TCC	Nonsyn	A/2229	A/2387	-/NA	-/NA	-/NA	-/NA	A/NA	A/2297	A/2398
*cox*I	2481-6	Frameshift	CAT ATT (HI)	ATA TTA (ML)	Nonsyn	/2230-5	#/2388-93	+/2480-5	+/2351-6	+/2283-8	+/2283-8	#/2320-5	#/2298-2303	#/2399-2404
*cox*I	2490	indel*	ATT (I)	TTA (L)	Nonsyn	-/NA	-/NA	A/2489	A/2360	A/2292	A/2292	-/NA	-/NA	-/NA
*cox*I	2797	SNV	TCA (S)	CCA (P)	Nonsyn.	T/2545	T/2703	T/2796	T/2667	T/2599	T/2599	C/2635	T/2613	T/2714
*cox*I	+2838	SNV	GTA (V)	GTG (V)	Syn.	A/2586	A/2743	A/2837	A/2708	A/2640	A/2640	G/2676	A/2654	A/2755
*cox*I	+3110	SNV	GTG (V)	GGG (G)	Nonsyn.	T/2858	T/3016	T/3109	T/1980	T/2912	T/2912	G/2948	T/2926	T/3028
*cox*I	3192	SNV	ATT (I)	ATG (M)	Nonsyn.	T/2940	T/3098	G/3191	T/3062	T/2994	T/2994	T/3030	T/3008	T/3109
*cox*I	3206	SNV	AAT (N)	ATC (I)	Nonsyn.	A/2954	A/3112	A/3205	A/3076	A/3008	T/3008	A/3044	A/3022	A/3122
*cox*I	3207	SNV	ATT (N)	AAC (N)	Syn.	T/2955	T/3113	C/3206	T/3077	T/3009	T/3009	T/3045	T/3023	T/3123
*cox*II	+3334	SNV	TGA (W)	TCA (S)	Nonsyn.	G/3082	G/3240	C/3333	G/3204	G/3136	C/3136	G/3172	G/3150	G/3251
*cox*II	3497	SNV	CTA (L)	CTT (L)	Syn.	T/3245	T/3402	A/3496	T/3367	T/3299	A/3299	T/3334	T/3313	T/3414
*cox*II	3521	SNV	ACA (T)	ACT (T)	Syn.	A/3269	A/3427	T/3520	A/3391	A/3323	T/3323	A/3359	A/3337	A/3438
*cox*II	3527	SNV	ATT (I)	ATA (M)	Nonsyn.	T/3275	T/3433	A/3526	T/3397	T/3329	A/3329	T/3365	T/3343	T/3444
*cox*II	3668	SNV	AAT (N)	AAC (N)	Syn.	C/3416	C/3574	C/3667	T/3538	T/3470	T/3470	T/3506	C/3484	T/3585
*cox*II	3988	SNV	TGA (W)	TCA (S)	Nonsyn.	G/3766	G/3894	G/3987	G/3858	G/3790	G/3790	C/3826	G/3804	G/3905
tRNA^{Asp}	4116	SNV	NA	NA		A/3864	A/4022	T/4115	A/3986	A/3918	T/3918	A/3954	A/3932	A/4033
tRNA^{Asp}	4123-5	indel	NA	NA		ATT/3871-3	-/NA	-/NA	-/NA	-/NA	-/NA	-/NA	-/NA	-/NA
tRNA^{Asp}	4129	SNV	NA	NA		T/3876	T/4032	T/4125	T/3996	A/3928	T/3928	T/3964	T/3942	T/4043
*atp*8	4241-3	indel	ATT (I)	--- (-)		-/	-/	-/	ATT/4108-10	-/	-/	-/	-/	-/
*atp*6	4752	SNV	AAT (N)	AAA (K)	Nonsyn.	T/4497	T/4652	A/4745	T/4619	T/4548	T/4548	T/4584	T/4562	T/4663
*atp*6	4754	SNV	ATT (I)	AGT (S)	Nonsyn.	T/4499	T/4654	G/4747	T/4621	T/4550	T/4550	T/4586	T/4564	T/4665
*atp*6	4757	SNV	ATT (I)	ACT (T)	Nonsyn.	T/4502	T/4657	C/4750	T/4624	T/4553	T/4553	T/4589	T/4567	T/4668
*atp*6	4760	SNV	CGA (R)	CCA (P)	Nonsyn.	G/4505	G/4660	C/4753	G/4627	G/4556	G/4556	G/4592	G/4570	G/4671
*atp*6	4762	SNV	CCT (P)	ACT (T)	Nonsyn.	C/4507	C/4662	A/4755	C/4629	C/4558	C/4558	C/4594	C/4572	C/4673
*atp*6	4885	SNV	TTA (I)	CTA (L)	Nonsyn.	T/4630	T/4785	C/4878	T/4752	T/4681	T/4681	T/4732	T/4695	T/4796
*cox*III	5079	SNV	AAA (K)	GAA (E)	Nonsyn.	A/4824	A/4979	G/5072	A/4946	A/4875	A/4875	A/4911	A/4889	A/4990
*cox*III	5426	SNV	CAC (H)	CAT (H)	Syn.	C/5171	C/5326	C/5419	T/5293	C/5222	T/5222	C/5258	C/5236	C/5337
*nd*3	4146	SNV	GGG (G)	GGA (G)	Syn.	G/5891	A/6046	A/6139	G/6013	G/5942	G/5942	G/5978	A/5956	G/6057
tRNA^{Ala}	6222	indel	NA	NA		A/5967	A/6122	A/6215	-/-	A/6018	A/6018	A/6054	A/6032	A/6133
tRNA^{Ala}	6255	indel	NA	NA		A/6000	A/6155	A/6248	-/-	A/6051	A/6051	A/6087	A/6065	A/6166
tRNA^{Arg}	6274	SNV	NA	NA		G/6019	G/6174	G/6267	T/6139	G//6070	G/6106	G//6106	G/6084	G/6185
tRNA^{Arg}	6275	SNV	NA	NA		A/6020	A/6175	A/6268	T/6140	A/6071	A/6071	A/6107	A/6085	A/6186
tRNA^{Arg}	6277	Indel	NA	NA		-/	-/	-/	A/6142	-/	-/	-/	-/	-/
tRNA^{Arg}	6278	SNV	NA	NA		G/6022	G/6177	G/6269	T/6143	G/6073	G/6073	G/6109	G/6087	G/6188
tRNA^{Arg}	6278	SNV	NA	NA		C/6023	C/6178	C/6270	G/6144	C/6074	C/6074	C/6110	C/6088	C/6189
tRNA^{Arg}	6279	indel	NA	NA		-/-	-/-	-/-	G/6144	-/-	-/-	-/-	-/-	-/-
tRNA^{Gln}	6523	indel	NA	NA		A/6267	-/-	-/-	-/-	-/-	-/-	-/-	-/-	-/-

Feature	Variant		Codon (AA) change			GenBank nr nucleotide database accessions								
	Pos	Type	Consensus	Alternate	Type	MN792233.1	MT492030.1	MT492031.1	MT492032.1	MT492033.1	MT492034.1	MT492035.1	MT492036.1	MT492037.1
*nd*5<compl>	6704	SNV	TAC (Y)	TAT (Y)	Syn.	A/6448	G/6602	G/6695	G/6568	G/6498	G/6498	G/6534	G/6512	G/6613
*nd*5<comp>	7288	SNV	GCT (A)	ACT (T)	Nonsyn.	C/7032	T/7186	T/7279	C/7152	C/7082	C/7082	C/7188	T/7096	C/7167
*nd*5<comp>	7339	SNV	GGA (G)	GGC (G)	Syn.	T/7083	C/7237	C/7330	T/7203	T/7133	T/7133	T/7169	C/7147	T/7248
*nd*5<comp>	7618	SNV	CTA (L)	TTA (L)	Syn.	G/7362	G/7516	G/7609	A/7482	G/7412	A/7412	A/7448	G/7416	A/7517
*nd*5<comp>	7844	SNV	GGT (G)	GGA (G)	Syn.	A/7588	T/7742	A/7835	A/7708	A/7844	A/7844	A/7674	A/7652	A/7753
*nd*5<comp>	8042	SNV	TTT (F)	TTG (L)	Nonsyn.	A/7786	A/7940	A/8033	C/7906	A/7836	A/7836	A/7872	A/7850	A/7951
*nd*5<comp>	8050	SNV	TTA (L)	TTG (L)	Syn.	A/7794	A/7948	A/8041	C/7914	A/7844	A/7844	A/7880	A/7858	A/7959
*nd*5<comp>	8126	SNV	TCA (S)	TCC (S)	Syn.	T/7870	T/8024	G/8117	T/7990	T/7920	T/7920	T/7956	T/7934	T/8035
tRNA^{His}	8353	SNV	NA	NA		C/8097	C/8251	A/8344	A/8217	A/8147	A/8147	A/8183	A/8161	A/8262
*nd*4<comp>	8615	SNV	TCT (S)	TCC (S)	Syn.	A/8359	A/8513	G/8606	A/8479	A/8409	A/8409	A/8445	A/8423	G/8524
*nd*4<comp>	8712	SNV	TTA (L)	GTA (V)	Nonsyn.	A/8456	A/8610	A/8703	A/8576	A/8506	A/8506	A/8542	C/8520	A/8621
*nd*4<comp>	8744	SNV	CGT (R)	CGC (R)	Syn.	G/8518	A/8672	A/8765	A/8638	A/8568	A/8568	A/8604	A/8582	A/8683
cytb	10803	SNV	CCA (P)	CCG (P)	Syn.	A/10547	A/10701	A/10794	G/10667	A/10597	A/10597	A/10633	A/10611	A/10712
cytb	10809	SNV	CCA (P)	CCC (P)	Syn.	A/10553	C/10707	C/10800	C/10673	A/10603	A/10603	A/10639	C/10617	A/10718
cytb	10893	SNV	ACA (T)	CCA (P)	Nonsyn.	A/10637	A/10791	A/10884	C/10757	A/10687	A/10687	C/10723	A/10701	A/10802
cytb	11607	SNV	GGT (G)	GGG (G)	Syn.	T/11351	T/11505	T/11598	G/11471	G/11401	T/11401	G/11437	T/11415	T/11516
*nd*1<comp>	11992	SNV	ATT (I)	ATG (M)	Nonsyn.	A/11736	A/11890	A/11983	A/11856	C/11786	C/11786	C/11822	A/11800	A/11901
rRNA^{LSU}	13079	SNV	NA	NA		A/12823	A/12977	A/13070	A/12943	A/12873	A/12873	A/12909	A/12887	G/12988
rRNA^{LSU}	13080	SNV	NA	NA		A/12824	A/12978	A/13071	A/12944	A/12874	A/12874	A/12910	A/12888	T/12989
rRNA^{LSU}	13082	SNV	NA	NA		A/12826	A/12980	A/13073	A/12946	A/12876	A/12876	A/12912	A/12890	C/12991
rRNA^{LSU}	13087	SNV	NA	NA		A/12831	A/12985	A/13078	A/12951	A/12881	A/12881	A/12917	A/12895	G/12996
rRNA^{LSU}	13088	SNV	NA	NA		A/12832	A/12986	A/13079	A/12952	A/12882	A/12882	A/12918	A/12896	G/12997
rRNA^{LSU}	13091	SNV	NA	NA		A/12835	A/12989	A/13082	A/12955	A/12885	A/12885	A/12921	A/12899	T/13000
rRNA^{LSU}	13424	indel	NA	NA		T/13168	T/13322	T/13415	T/13288	-/NA	T/13218	T/13254	T/13232	T/13333
rRNA^{LSU}	13450	indel	NA	NA		-/NA	-/NA	-/NA	-/NA	A/13243	A/13244	-/NA	-/NA	-/NA
rRNA^{LSU}	13863	SNV	NA	NA		C/13606	T/13760	T/13853	T/13726	T/13656	T/13657	T/13692	T/13670	T/13771
rRNA^{LSU}	14027	indel	NA	NA		-/NA	G/13924	-/NA	G/13890	G/13820	G/13821	G/13856	G/13834	G/13935
rRNA^{LSU}	14071	SNV	NA	NA		A/13813	A/13968	G/14060	G/13934	G/13864	G/13865	A/13900	G/13878	G/13979
rRNA^{LSU}	14079	SNV	NA	NA		T/13821	C/13976	C/14068	C/13942	C/13872	C/13873	C/13908	C/13886	C/13987
rRNA^{LSU}	14118	SNV	NA	NA		A/13860	A/14015	T/14107	T/13981	T/13911	T/13912	A/13947	A/13925	T/14026
rRNA^{LSU}	14218	SNV	NA	NA		A/13960	C/14115	A/14207	C/14081	A/14011	A/14012	A/14047	C/14025	C/14126
rRNA^{SSU}	14533-5	SNVs	NA	NA		TTT/14275	AAA/14430	TTT/14522	AAA/14396	TTT/14326	TTT/14327	TTT/14362	TTT/14340	TTT/14441
rRNA^{SSU}	14538	SNV	NA	NA		A/14280	A/14435	A/14527	C/14401	A/14331	C/14332	A/14367	A/14345	A/14446
rRNA^{SSU}	14559	indel	NA	NA		A/14301	-/NA	-/NA	-/NA	-/NA	-/NA	-/NA	-/NA	-/NA
rRNA^{SSU}	14822	SNV	NA	NA		A/14564	T/14718	A/14810	A/14684	A/14614	A/14615	A/14650	A/14628	A/14729
rRNA^{SSU}	14826	SNV	NA	NA		T/14568	A/14722	T/14814	T/14688	T/14618	T/14619	T/14654	T/14632	T/14733
rRNA^{SSU}	14828	SNV	NA	NA		T/14570	A/14724	T/14816	T/14690	T/14620	T/14621	T/14656	T/14634	T/14735

Table S5. Example Linux shell commands to 1) align fastq reads to a reference genome and identify reads that meet Phred quality, alignment, and mapq scores, 2) de novo assemble reads and prepare them for annotation, and 3) identify variable positions from de novo assembled sequences and extract sequences that code for associated genes.

Objective	Specifically	Code
1	Check quality of reads for all fastq files received from ISU DNA Facility	`module load fastqc/0.11.7-d5mgqc7`<br>`fastqc ./06Mt-D_S4_L001_R1_001.fastq -o .`<br>`fastqc ./06Mt-D_S4_L001_R2_001.fastq -o .`<br><br>`module load py-multiqc/1.5-py2-lqqx3ht`<br>`multiqc .`
1	After viewing quality reports, trim the first 15 and last 10 nucleotides from each read	`module load trimmomatic/0.36-lkktrba`<br><br>`java -jar /opt/rit/spack-app/linux-rhel7-x86_64/gcc-4.8.5/trimmomatic-0.36-lkktrbakk2a32djk45nokwrtgc6h5mzo/bin/trimmomatic-0.36.jar PE -phred33 -trimlog ./logfile_06mt ./06Mt-D_S4_L001_R1_001.fastq ./06Mt-D_S4_L001_R2_001.fastq ./06mt_R1_paired.fq ./06mt_R1_unpaired.fq ./06mt_R2_paired.fq ./06mt_R2_unpaired.fq HEADCROP:15 TAILCROP:10`
1	Count to ensure that the proper number of nucleotides have been removed	`head -n 2 ./04Mt-C_S3_L001_R1_001.fastq | tail -n 1 |awk '{print length}'`<br>`head -n 2 ./04mt_R1_paired.fq | tail -n 1 | awk '{print length}'`
1	Align reads to the reference genome and filter for those reads that map to the reference and have a mapq > 30	`module load bowtie2/2.3.4.1-py2-jl5zqym`<br>`bowtie2-build -f ../../MtGenome_ECB.fasta ecbmt`<br>`bowtie2 -x ecbmt -t -p 4 -U ./06mt_R1_paired.fq,./06mt_R2_paired.fq -S 06mt.sam`<br><br>`module load samtools/1.9-k6deoga`<br>`samtools view -S -b 06mt.sam > 06mt.bam`

		samtools view -bhF 2 06mt.bam > 06mtmap.bam<br>samtools view -bhq 30 06mtmap.bam > 06mtmapq.bam<br>samtools view -c 06mt.bam<br>samtools view -c 06mtmap.bam<br>samtools view -c 06mtmapq.bam
2	Sort mitochondrial aligned, mitochondrion-enriched reads	module load bowtie2/2.3.4.1-py2-jl5zqym<br>bowtie2-build -f ../../MtGenome_ECB.fasta ecbmt<br>bowtie2 -x ecbmt -t -p 4 -U<br>./06mt_R1_paired.fq,./06mt_R2_paired.fq -S 06mt.sam<br><br>module load samtools/1.9-k6deoga<br>samtools view -S -b 06mt.sam > 06mt.bam<br>samtools sort -o 06mt_s.bam 06mt.bam<br>samtools bam2fq 06mt_s.bam > 06mt_s_all.fastq
2	de novo assemble sorted reads	module load spades/3.11.1-py2-arfy7sc<br><br>plasmidspades.py -s ./06mt_s_all.fastq -o 06mt_s_all_spades
2	Blast kmer contigs against reference genome	module load ncbi-rmblastn/2.6.0-2kyyml7<br><br>blastn -query K21final_contigs.fasta -subject MtGenome_ECB.fasta<br>-outfmt "6 qseqid sseqid pident length mismatch gapopen qstart<br>qend sstart send evalue bitscore"
2	Extract contigs that mapped to reference genome and compile in one fasta file	awk '{print $1}' 06k21blast.out > 06k21list.txt<br>awk '{print $1}' 06k33blast.out > 06k33list.txt<br>awk '{print $1}' 06k55blast.out > 06k55list.txt<br><br>module load seqtk/1.2-by5hlhw<br><br>seqtk subseq K21final_contigs.fasta 06k21list.txt ><br>06k21_final.fasta

		seqtk subseq K33final_contigs.fasta 06k33list.txt > 06k33_final.fasta<br>seqtk subseq K55final_contigs.fasta 06k55list.txt > 06k55_final.fasta<br><br>cat *.fasta > ./06all.fasta
2	Use CAP3 to create one contig	module load cap3/2015-02-11-2jwa5sb<br><br>cap3 06all.fasta
3	Identify variable positions in comparison to reference genome	module load bcftools/1.9-womp5fh<br><br>bcftools mpileup -f ./MtGenome_ECB.fasta ./02mt_s.bam | bcftools call --ploidy 1 -mv -Ob -o 02mtcalls.bcf<br><br>bcftools view 02mtcalls.bcf | vcfutils.pl varFilter -D 2000 -W 5 > 02mtvar.vcf
3	Extract stretch of sequence that codes for genes with variable positions	module load bedtools2/2.27.1-s2mtpsu<br><br>bedtools getfasta -fi AllECBmtGenomes_Submit_BankIT.fasta -bed COI.bed -fo COI.fasta

Table S6. Blast table for *de novo* assembled *Ostrinia nubilalis* genomes from enriched and unenriched samples. Total reads acquired from each sample were *de novo* assembled and Blast aligned to determine which contigs represent potential mitochondrial sequences. Higher read depths (RPKM) for contig indicates contigs that were formulated from mitochondrial origin.

Query Sequence Accession *qseqid*	Assembly ID	Subject Sequence ID/Contig ID *sseqid*	Contig Length	Alignment Length *length*	Percentage of Identical Matches *pident*	Mismatch *mismatch*	Gap Opening *gapopen*	Start of Alignment in Query *qstart*	End of Alignment in Query *qend*	Start of Alignment in Subject *sstart*	End of Alignment in Subject *send*	Expect Value *evalue*	Bit Score *bitscore*	True Alignment Length	Percent Coverage	Total Reads	Reads to Align	RPKM
AF442957.1	02	NODE_4_length_6530_cov_3705.010005	6,530	6,533	99.648	17	6	8,005	14,535	1	6,529	0	11932	6,527	99.95	2,784,696	298,415	16.40
AF442957.1	02	NODE_6_length_8006_cov_3516.763452	8,006	8,010	99.363	40	9	1	8,003	1	8,006	0	14499	8,001	99.94	2,784,696	359,637	16.12
AF442957.1	03	NODE_1_length_263_cov_4820.376033	263	262	99.618	1	0	1	262	262	1	2.36E-136	479	262	99.62	3,542,792	8,515	9.17
AF442957.1	03	NODE_10_length_44_cov_6822.272727	44	44	100	0	0	4,896	4,939	44	1	7.76E-17	82.4	44	100.00	3,542,792	10	0.06
AF442957.1	03	NODE_109_length_39_cov_13.666667	39	39	97.436	1	0	12,896	12,934	39	1	2.17E-12	67.6	39	100.00	3,542,792	3	0.02
AF442957.1	03	NODE_111_length_194_cov_5445.745342	194	194	100	0	0	11,873	12,066	194	1	3.20E-100	359	194	100.00	3,542,792	3,082	4.48
AF442957.1	03	NODE_115_length_61_cov_34.785714	61	61	98.361	1	0	12,868	12,928	1	61	1.28E-24	108	61	100.00	3,542,792	6	0.03
AF442957.1	03	NODE_116_length_61_cov_6862.714286	61	61	100	0	0	12,868	12,928	1	61	2.75E-26	113	61	100.00	3,542,792	64	0.30
AF442957.1	03	NODE_117_length_807_cov_5695.714470	807	807	99.876	1	0	12,094	12,900	807	1	0	1485	807	100.00	3,542,792	25,958	9.08
AF442957.1	03	NODE_118_length_39_cov_34.500000	39	39	97.436	1	0	12,896	12,934	39	1	2.17E-12	67.6	39	100.00	3,542,792	2	0.01
AF442957.1	03	NODE_119_length_1708_cov_5265.923582	1,708	1,709	99.239	11	2	6,232	7,939	1,708	1	0	3083	1,707	99.94	3,542,792	61,966	10.23
AF442957.1	03	NODE_121_length_44_cov_20.181818	44	44	97.727	1	0	4,896	4,939	44	1	3.61E-15	76.8	44	100.00	3,542,792	4	0.03
AF442957.1	03	NODE_122_length_657_cov_4376.812500	657	658	98.784	5	3	5,575	6,230	1	657	0	1168	655	99.70	3,542,792	21,160	9.08
AF442957.1	03	NODE_125_length_38_cov_4.800000	38	38	97.368	1	0	264	301	1	38	7.82E-12	65.8	38	100.00	3,542,792	-	-
AF442957.1	03	NODE_126_length_64_cov_7.935484	64	64	98.438	1	0	238	301	64	1	2.75E-26	113	64	100.00	3,542,792	43	0.19
AF442957.1	03	NODE_127_length_36_cov_10.333333	36	36	97.222	1	0	269	304	1	36	1.01E-10	62.1	36	100.00	3,542,792	-	-
AF442957.1	03	NODE_136_length_46_cov_4565.461538	46	46	100	0	0	251	296	46	1	6.00E-18	86.1	46	100.00	3,542,792	11	0.07
AF442957.1	03	NODE_137_length_38_cov_4454.800000	38	38	100	0	0	264	301	1	38	1.68E-13	71.3	38	100.00	3,542,792	2	0.01
AF442957.1	03	NODE_14_length_479_cov_6492.801310	479	479	100	0	0	3,952	4,430	479	1	0	885	479	100.00	3,542,792	15,340	9.04
AF442957.1	03	NODE_140_length_37_cov_5228.000000	37	37	100	0	0	12,034	12,070	37	1	6.04E-13	69.4	37	100.00	3,542,792	4	0.03
AF442957.1	03	NODE_141_length_678_cov_5749.992248	678	678	99.853	1	0	4,930	5,607	1	678	0	1247	678	100.00	3,542,792	22,304	9.29
AF442957.1	03	NODE_142_length_56_cov_33.217391	56	56	98.214	1	0	4,907	4,962	56	1	7.71E-22	99	56	100.00	3,542,792	3	0.02
AF442957.1	03	NODE_143_length_56_cov_6867.304348	56	56	100	0	0	4,907	4,962	56	1	1.66E-23	104	56	100.00	3,542,792	50	0.25
AF442957.1	03	NODE_150_length_35_cov_4484.000000	35	35	100	0	0	272	306	1	35	7.82E-12	65.8	35	100.00	3,542,792	2	0.02
AF442957.1	03	NODE_155_length_63_cov_2.800000	63	63	96.825	2	0	12,038	12,100	1	63	4.61E-24	106	63	100.00	3,542,792	3	0.01
AF442957.1	03	NODE_156_length_37_cov_4.250000	37	37	97.297	1	0	12,034	12,070	37	1	2.81E-11	63.9	37	100.00	3,542,792	1	0.01
AF442957.1	03	NODE_16_length_63_cov_7074.047619	63	63	100	0	0	4,439	4,501	63	1	2.13E-27	117	63	100.00	3,542,792	70	0.31
AF442957.1	03	NODE_163_length_42_cov_7.222222	42	43	97.674	0	1	6,222	6,264	42	1	4.67E-14	73.1	42	100.00	3,542,792	-	-
AF442957.1	03	NODE_164_length_43_cov_4900.200000	43	43	100	0	0	6,222	6,264	43	1	2.79E-16	80.5	43	100.00	3,542,792	3	0.02
AF442957.1	03	NODE_167_length_61_cov_33.285714	61	61	98.361	1	0	280	340	61	1	1.28E-24	108	61	100.00	3,542,792	1	0.00
AF442957.1	03	NODE_17_length_1200_cov_5736.015424	1,200	1,201	99.667	3	1	8,005	9,205	1,200	1	0	2194	1,200	100.00	3,542,792	42,580	10.01
AF442957.1	03	NODE_17_length_35_cov_7182.428571	35	35	100	0	0	4,481	4,515	1	35	7.82E-12	65.8	35	100.00	3,542,792	14	0.11
AF442957.1	03	NODE_170_length_214_cov_4046.502762	214	213	99.531	1	0	1	213	213	1	4.08E-109	388	213	99.53	3,542,792	4,083	5.41
AF442957.1	03	NODE_173_length_62_cov_16.068966	62	62	98.387	1	0	235	296	1	62	3.56E-25	110	62	100.00	3,542,792	27	0.12
AF442957.1	03	NODE_174_length_725_cov_5243.169075	725	726	99.725	0	2	12,930	13,654	1	725	0	1328	724	99.86	3,542,792	21,329	8.29
AF442957.1	03	NODE_176_length_61_cov_6312.821429	61	61	100	0	0	12,902	12,962	61	1	2.75E-26	113	61	100.00	3,542,792	52	0.24
AF442957.1	03	NODE_177_length_59_cov_4.807692	59	59	96.61	2	0	238	296	1	59	7.71E-22	99	59	100.00	3,542,792	-	-

Query Sequence Accession *qseqid*	Assembly ID	Subject Sequence ID/Contig ID *sseqid*	Contig Length	Alignment Length *length*	Percentage of Identical Matches *pident*	Mismatch *mismatch*	Gap Opening *gapopen*	Start of Alignment in Query *qstart*	End of Alignment in Query *qend*	Start of Alignment in Subject *sstart*	End of Alignment in Subject *send*	Expect Value *evalue*	Bit Score *bitscore*	True Alignment Length	Percent Coverage	Total Reads	Reads to Align	RPKM
AF442957.1	03	NODE_178_length_64_cov_6.193548	64	64	95.312	3	0	238	301	1	64	5.96E-23	102	64	100.00	3,542,792	1	0.00
AF442957.1	03	NODE_179_length_941_cov_5097.903084	941	941	99.894	1	0	314	1,254	1	941	0	1733	941	100.00	3,542,792	29,975	8.99
AF442957.1	03	NODE_18_length_36_cov_4360.666667	36	36	100	0	0	269	304	1	36	2.17E-12	67.6	36	100.00	3,542,792	1	0.01
AF442957.1	03	NODE_19_length_63_cov_3.866667	63	63	98.413	1	0	12,038	12,100	63	1	9.90E-26	111	63	100.00	3,542,792	4	0.02
AF442957.1	03	NODE_23_length_1894_cov_5852.649760	1,894	1,895	99.261	11	3	5,587	7,479	1,894	1	0	3419	1,892	99.89	3,542,792	68,344	10.18
AF442957.1	03	NODE_29_length_1898_cov_5693.200536	1,898	1,899	99.789	2	2	9,209	11,106	1,898	1	0	3483	1,897	99.95	3,542,792	67,688	10.06
AF442957.1	03	NODE_30_length_35_cov_6895.428571	35	35	100	0	0	4,410	4,444	35	1	7.82E-12	65.8	35	100.00	3,542,792	5	0.04
AF442957.1	03	NODE_33_length_521_cov_5543.370000	521	522	98.659	5	2	7,483	8,003	521	1	0	924	520	99.81	3,542,792	16,708	9.03
AF442957.1	03	NODE_35_length_1113_cov_7002.973443	1,113	1,113	99.73	3	0	4,495	5,607	1,113	1	0	2039	1,113	100.00	3,542,792	46,564	11.81
AF442957.1	03	NODE_38_length_749_cov_6011.403631	749	749	99.733	2	0	11,108	11,856	1	749	0	1373	749	100.00	3,542,792	25,966	9.79
AF442957.1	03	NODE_39_length_5674_cov_6560.328498	5,674	5,677	99.771	8	5	8,005	13,679	1	5,674	0	10406	5,672	99.96	3,542,792	234,621	11.67
AF442957.1	03	NODE_4_length_39_cov_4563.166667	39	39	100	0	0	274	312	1	39	4.67E-14	73.1	39	100.00	3,542,792	5	0.04
AF442957.1	03	NODE_45_length_56_cov_6544.565217	56	56	100	0	0	4,873	4,928	56	1	1.66E-23	104	56	100.00	3,542,792	49	0.25
AF442957.1	03	NODE_46_length_2894_cov_6629.362687	2,894	2,896	99.137	19	4	1,063	3,954	2,894	1	0	5204	2,892	99.93	3,542,792	117,193	11.42
AF442957.1	03	NODE_46_length_44_cov_36.636364	44	44	97.727	1	0	4,896	4,939	1	44	3.61E-15	76.8	44	100.00	3,542,792	1	0.01
AF442957.1	03	NODE_48_length_354_cov_5815.882883	354	354	98.87	4	0	13,996	14,349	1	354	0	632	354	100.00	3,542,792	8,298	6.62
AF442957.1	03	NODE_48_length_61_cov_4681.714286	61	61	100	0	0	280	340	1	61	2.75E-26	113	61	100.00	3,542,792	45	0.21
AF442957.1	03	NODE_5_length_35_cov_7219.714286	35	35	100	0	0	4,425	4,459	35	1	7.82E-12	65.8	35	100.00	3,542,792	1	0.01
AF442957.1	03	NODE_5_length_39_cov_30.166667	39	39	97.436	1	0	274	312	1	39	2.17E-12	67.6	39	100.00	3,542,792	1	0.01
AF442957.1	03	NODE_53_length_49_cov_5299.437500	49	49	100	0	0	12,038	12,086	49	1	1.29E-19	91.6	49	100.00	3,542,792	22	0.13
AF442957.1	03	NODE_6_length_65_cov_2.093750	65	69	94.203	0	1	1,222	1,290	65	1	5.96E-23	102	68	104.62	3,542,792	2	0.01
AF442957.1	03	NODE_60_length_62_cov_4.827586	62	62	98.387	1	0	235	296	1	62	3.56E-25	110	62	100.00	3,542,792	29	0.13
AF442957.1	03	NODE_7_length_69_cov_5592.694444	69	69	100	0	0	1,222	1,290	69	1	9.83E-31	128	69	100.00	3,542,792	47	0.19
AF442957.1	03	NODE_70_length_63_cov_6.933333	63	63	98.413	1	0	12,038	12,100	63	1	9.90E-26	111	63	100.00	3,542,792	3	0.01
AF442957.1	03	NODE_73_length_865_cov_4499.622596	865	865	98.844	9	1	13,671	14,535	865	2	0	1541	864	99.88	3,542,792	33,705	11.00
AF442957.1	03	NODE_74_length_39_cov_6419.000000	39	39	100	0	0	12,896	12,934	1	39	4.67E-14	73.1	39	100.00	3,542,792	4	0.03
AF442957.1	03	NODE_8_length_820_cov_5848.227785	820	820	99.878	1	0	264	1,083	1	820	0	1509	820	100.00	3,542,792	26,071	8.97
AF442957.1	03	NODE_81_length_2699_cov_5776.120780	2,699	2,701	99.074	19	4	1,258	3,954	2,699	1	0	4844	2,697	99.93	3,542,792	107,171	11.20
AF442957.1	03	NODE_9_length_189_cov_4425.261905	189	188	99.468	1	0	14,348	14,535	1	188	3.23E-95	342	188	99.47	3,542,792	3,265	4.90
AF442957.1	03	NODE_95_length_933_cov_6044.946667	933	933	99.786	2	0	3,973	4,905	933	1	0	1712	933	100.00	3,542,792	40,698	12.31
AF442957.1	03	NODE_96_length_56_cov_36.130435	56	56	98.214	1	0	4,873	4,928	1	56	7.71E-22	99	56	100.00	3,542,792	7	0.04
AF442957.1	04	NODE_1_length_39_cov_39.000000	39	39	97.436	1	0	274	312	1	39	1.97E-12	67.6	39	100.00	3,556,284	-	-
AF442957.1	04	NODE_1_length_4097_cov_5979.746075	4,097	4,099	99.756	6	4	9,583	13,679	1	4,097	0	7511	4,095	99.95	3,556,284	135,617	9.30
AF442957.1	04	NODE_14_length_189_cov_3985.767857	189	188	99.468	1	0	14,348	14,535	1	188	2.92E-95	342	188	99.47	3,556,284	2,842	4.25
AF442957.1	04	NODE_14_length_61_cov_4395.857143	61	61	100	0	0	280	340	1	61	2.49E-26	113	61	100.00	3,556,284	26	0.12
AF442957.1	04	NODE_17_length_4029_cov_4913.654154	4,029	4,031	99.454	17	5	3,976	8,003	4,029	1	0	7317	4,026	99.93	3,556,284	146,804	10.24
AF442957.1	04	NODE_2_length_39_cov_4354.500000	39	39	100	0	0	274	312	1	39	4.22E-14	73.1	39	100.00	3,556,284	3	0.02
AF442957.1	04	NODE_21_length_1240_cov_6152.636587	1,240	1,240	98.952	10	1	2,648	3,884	1,240	1	0	2215	1,239	99.92	3,556,284	40,385	9.16
AF442957.1	04	NODE_21_length_877_cov_4209.104265	877	877	98.86	9	1	13,659	14,535	877	2	0	1563	876	99.89	3,556,284	32,630	10.46
AF442957.1	04	NODE_22_length_267_cov_4582.276423	267	267	99.625	1	0	1	267	1	267	3.54E-139	488	267	100.00	3,556,284	5,370	5.66
AF442957.1	04	NODE_23_length_468_cov_6078.536913	468	468	100	0	0	3,963	4,430	1	468	0	865	468	100.00	3,556,284	13,367	8.03
AF442957.1	04	NODE_23_length_5668_cov_5142.774623	5,668	5,671	99.771	8	5	8,005	13,673	1	5,668	0	10395	5,666	99.96	3,556,284	189,678	9.41

Query Sequence Accession *qseqid*	Assembly ID	Subject Sequence ID/Contig ID *sseqid*	Contig Length	Alignment Length *length*	Percentage of Identical Matches *pident*	Mismatch *mismatch*	Gap Opening *gapopen*	Start of Alignment in Query *qstart*	End of Alignment in Query *qend*	Start of Alignment in Subject *sstart*	End of Alignment in Subject *send*	Expect Value *evalue*	Bit Score *bitscore*	True Alignment Length	Percent Coverage	Total Reads	Reads to Align	RPKM
AF442957.1	04	NODE_26_length_100_cov_5119.544304	100	100	100	0	0	269	368	1	100	5.20E-48	185	100	100.00	3,556,284	519	1.46
AF442957.1	04	NODE_29_length_306_cov_4139.410256	306	306	99.673	1	0	1	306	1	306	7.40E-161	560	306	100.00	3,556,284	8,791	8.08
AF442957.1	04	NODE_29_length_34_cov_6004.769231	34	34	100	0	0	2,635	2,668	34	1	2.54E-11	63.9	34	100.00	3,556,284	1	0.01
AF442957.1	04	NODE_3_length_35_cov_6382.571429	35	35	100	0	0	4,425	4,459	35	1	7.07E-12	65.8	35	100.00	3,556,284	-	-
AF442957.1	04	NODE_31_length_243_cov_5706.333333	243	243	100	0	0	5,316	5,558	1	243	1.67E-127	449	243	100.00	3,556,284	4,205	4.87
AF442957.1	04	NODE_34_length_525_cov_5158.390873	525	526	98.669	5	2	7,491	8,015	525	1	0	931	524	99.81	3,556,284	14,606	7.81
AF442957.1	04	NODE_36_length_35_cov_6126.571429	35	35	100	0	0	4,410	4,444	35	1	7.07E-12	65.8	35	100.00	3,556,284	4	0.03
AF442957.1	04	NODE_37_length_1585_cov_5748.097826	1,585	1,587	99.181	10	3	1,070	2,655	1,585	1	0	2856	1,584	99.94	3,556,284	52,586	9.32
AF442957.1	04	NODE_38_length_341_cov_5416.590625	341	341	98.827	4	0	14,009	14,349	341	1	2.60E-175	608	341	100.00	3,556,284	7,211	5.95
AF442957.1	04	NODE_40_length_705_cov_5458.679825	705	705	99.858	1	0	371	1,075	1	705	0	1297	705	100.00	3,556,284	17,825	7.11
AF442957.1	04	NODE_41_length_61_cov_22.357143	61	61	98.361	1	0	280	340	61	1	1.16E-24	108	61	100.00	3,556,284	4	0.02
AF442957.1	04	NODE_43_length_3643_cov_5033.820776	3,643	3,645	99.259	21	4	314	3,954	1	3,643	0	6577	3,641	99.95	3,556,284	133,118	10.27
AF442957.1	04	NODE_44_length_880_cov_6476.083818	880	880	99.659	3	0	4,439	5,318	880	1	0	1609	880	100.00	3,556,284	28,618	9.14
AF442957.1	04	NODE_45_length_39_cov_6816.388889	39	39	100	0	0	5,298	5,336	1	39	4.22E-14	73.1	39	100.00	3,556,284	5	0.04
AF442957.1	04	NODE_51_length_1919_cov_5292.404110	1,919	1,920	99.375	9	3	5,562	7,479	1	1,919	0	3476	1,917	99.90	3,556,284	58,035	8.50
AF442957.1	04	NODE_52_length_1580_cov_5789.840282	1,580	1,581	99.81	2	1	8,005	9,585	1	1,580	0	2902	1,580	100.00	3,556,284	48,857	8.69
AF442957.1	06	NODE_1_length_511_cov_4590.776151	511	511	100	0	0	13,163	13,673	1	511	0	944	511	100.00	2,542,850	16,441	12.65
AF442957.1	06	NODE_104_length_187_cov_5200.373494	187	187	100	0	0	14,009	14,195	1	187	2.31E-96	346	187	100.00	2,542,850	1,861	3.91
AF442957.1	06	NODE_11_length_5156_cov_4949.127464	5,156	5,159	99.748	8	5	8,005	13,161	5,156	1	0	9450	5,154	99.96	2,542,850	185,493	14.14
AF442957.1	06	NODE_117_length_172_cov_3616.251656	172	171	99.415	1	0	14,365	14,535	172	2	8.43E-86	311	171	99.42	2,542,850	1,913	4.40
AF442957.1	06	NODE_121_length_38_cov_4889.352941	38	38	100	0	0	269	306	1	38	1.56E-13	71.3	38	100.00	2,542,850	1	0.01
AF442957.1	06	NODE_13_length_895_cov_4070.013921	895	895	98.883	9	1	13,641	14,535	1	894	0	1596	894	99.89	2,542,850	34,173	15.02
AF442957.1	06	NODE_154_length_1535_cov_5870.640687	1,535	1,535	99.674	5	0	10,189	11,723	1,535	1	0	2808	1,535	100.00	2,542,850	43,268	11.09
AF442957.1	06	NODE_156_length_45_cov_6009.041667	45	45	100	0	0	5,322	5,366	45	1	2.00E-17	84.2	45	100.00	2,542,850	3	0.03
AF442957.1	06	NODE_158_length_213_cov_5299.416667	213	213	100	0	0	5,346	5,558	1	213	8.14E-111	394	213	100.00	2,542,850	2,792	5.15
AF442957.1	06	NODE_166_length_86_cov_5450.938462	86	86	100	0	0	9,041	9,126	1	86	3.23E-40	159	86	100.00	2,542,850	46	0.21
AF442957.1	06	NODE_170_length_438_cov_5566.424460	438	438	100	0	0	9,731	10,168	1	438	0	809	438	100.00	2,542,850	9,069	8.14
AF442957.1	06	NODE_181_length_624_cov_5407.877280	624	624	100	0	0	11,725	12,348	624	1	0	1153	624	100.00	2,542,850	14,066	8.86
AF442957.1	06	NODE_184_length_865_cov_5966.774882	865	865	99.653	3	0	4,439	5,303	865	1	0	1581	865	100.00	2,542,850	23,455	10.66
AF442957.1	06	NODE_185_length_44_cov_6167.782609	44	44	100	0	0	5,283	5,326	1	44	7.20E-17	82.4	44	100.00	2,542,850	16	0.14
AF442957.1	06	NODE_190_length_559_cov_6003.403346	559	559	99.106	5	0	3,127	3,685	559	1	0	1005	559	100.00	2,542,850	13,721	9.65
AF442957.1	06	NODE_191_length_38_cov_6149.352941	38	38	100	0	0	3,665	3,702	1	38	1.56E-13	71.3	38	100.00	2,542,850	-	-
AF442957.1	06	NODE_204_length_28_cov_5849.000000	28	28	100	0	0	3,698	3,725	1	28	5.64E-08	52.8	28	100.00	2,542,850	2	0.03
AF442957.1	06	NODE_220_length_1894_cov_5061.431393	1,894	1,895	99.367	9	3	5,587	7,479	1	1,894	0	3430	1,892	99.89	2,542,850	50,232	10.42
AF442957.1	06	NODE_231_length_37_cov_6197.937500	37	37	100	0	0	5,306	5,342	1	37	5.60E-13	69.4	37	100.00	2,542,850	-	-
AF442957.1	06	NODE_24_length_35_cov_5824.857143	35	35	100	0	0	4,425	4,459	1	35	7.25E-12	65.8	35	100.00	2,542,850	1	0.01
AF442957.1	06	NODE_240_length_216_cov_5638.179487	216	217	99.078	0	2	12,946	13,161	1	216	3.79E-109	388	215	99.54	2,542,850	3,769	6.83
AF442957.1	06	NODE_248_length_593_cov_5739.346154	593	593	99.831	1	0	12,352	12,944	1	593	0	1090	593	100.00	2,542,850	13,764	9.13
AF442957.1	06	NODE_259_length_440_cov_4784.124105	440	441	98.413	5	2	7,500	7,939	1	440	0	774	439	99.77	2,542,850	8,432	7.52
AF442957.1	06	NODE_263_length_143_cov_4829.254098	143	143	100	0	0	9,443	9,585	143	1	6.66E-72	265	143	100.00	2,542,850	595	1.64
AF442957.1	06	NODE_27_length_28_cov_6139.142857	28	28	100	0	0	3,683	3,710	28	1	5.64E-08	52.8	28	100.00	2,542,850	-	-
AF442957.1	06	NODE_287_length_557_cov_5286.820896	557	557	99.82	1	0	371	927	557	1	0	1024	557	100.00	2,542,850	11,879	8.39

Query Sequence Accession *qseqid*	Assembly ID	Subject Sequence ID/Contig ID *sseqid*	Contig Length	Alignment Length *length*	Percentage of Identical Matches *pident*	Mismatch *mismatch*	Gap Opening *gapopen*	Start of Alignment in Query *qstart*	End of Alignment in Query *qend*	Start of Alignment in Subject *sstart*	End of Alignment in Subject *send*	Expect Value *evalue*	Bit Score *bitscore*	True Alignment Length	Percent Coverage	Total Reads	Reads to Align	RPKM
AF442957.1	06	NODE_290_length_281_cov_5343.992308	281	281	100	0	0	13,163	13,443	1	281	1.29E-148	520	281	100.00	2,542,850	5,210	7.29
AF442957.1	06	NODE_34_length_205_cov_5629.619565	205	205	100	0	0	9,226	9,430	205	1	2.28E-106	379	205	100.00	2,542,850	2,385	4.58
AF442957.1	06	NODE_6_length_8009_cov_4718.277332	8,009	8,009	99.376	39	9	2	8,003	8,005	1	0	14502	8,000	99.89	2,542,850	281,284	13.81
AF442957.1	06	NODE_70_length_138_cov_5063.931624	138	138	97.101	4	0	14,196	14,333	138	1	1.88E-62	233	138	100.00	2,542,850	503	1.43
AF442957.1	06	NODE_75_length_252_cov_4186.562771	252	248	99.597	1	0	2	249	248	1	1.32E-128	453	248	98.41	2,542,850	4,182	6.63
AF442957.1	06	NODE_8_length_992_cov_5570.472709	992	993	99.698	2	1	8,005	8,997	992	1	0	1816	992	100.00	2,542,850	26,176	10.37
AF442957.1	06	NODE_86_length_1978_cov_5493.501788	1,978	1,980	99.091	15	3	1,147	3,125	1	1,978	0	3554	1,977	99.95	2,542,850	56,989	11.32
AF442957.1	06	NODE_88_length_35_cov_5638.214286	35	35	100	0	0	4,410	4,444	1	35	7.25E-12	65.8	35	100.00	2,542,850	1	0.01
AF442957.1	06	NODE_89_length_417_cov_5749.729798	417	417	100	0	0	4,014	4,430	417	1	0	771	417	100.00	2,542,850	9,154	8.63
AF442957.1	06	NODE_99_length_151_cov_5142.161538	151	151	98.013	0	1	3,705	3,852	151	1	3.10E-70	259	150	99.34	2,542,850	821	2.14
AF442957.1	07	NODE_1_length_36_cov_7786.133333	36	36	100	0	0	4,424	4,459	36	1	1.93E-12	67.6	36	100.00	3,571,772	3	0.02
AF442957.1	07	NODE_1_length_511_cov_5531.399582	511	511	100	0	0	13,163	13,673	1	511	0	944	511	100.00	3,571,772	14,723	8.07
AF442957.1	07	NODE_10_length_2443_cov_5421.632780	2,443	2,445	99.223	14	5	5,562	8,003	2,443	1	0	4405	2,440	99.88	3,571,772	88,689	10.16
AF442957.1	07	NODE_11_length_483_cov_7096.108225	483	483	100	0	0	3,962	4,444	1	483	0	893	483	100.00	3,571,772	15,048	8.72
AF442957.1	07	NODE_12_length_3854_cov_6856.873728	3,854	3,856	99.274	22	4	1	3,852	1	3,854	0	6961	3,852	99.95	3,571,772	154,256	11.20
AF442957.1	07	NODE_13_length_3956_cov_5930.787663	3,956	3,958	99.293	22	4	1	3,954	1	3,956	0	7149	3,954	99.95	3,571,772	160,870	11.38
AF442957.1	07	NODE_15_length_189_cov_4655.809524	189	188	99.468	1	0	14,348	14,535	189	2	2.86E-95	342	188	99.47	3,571,772	3,296	4.91
AF442957.1	07	NODE_16_length_678_cov_5889.984779	678	679	98.675	8	1	13,671	14,349	678	1	0	1203	678	100.00	3,571,772	20,175	8.32
AF442957.1	07	NODE_19_length_7600_cov_6831.448608	7,600	7,605	99.579	22	10	5,562	13,161	1	7,600	0	13858	7,595	99.93	3,571,772	300,156	11.05
AF442957.1	07	NODE_20_length_5156_cov_6173.207886	5,156	5,159	99.748	8	5	8,005	13,161	5,156	1	0	9450	5,154	99.96	3,571,772	202,931	11.01
AF442957.1	07	NODE_24_length_1622_cov_6432.490875	1,622	1,622	99.815	3	0	3,949	5,570	1,622	1	0	2979	1,622	100.00	3,571,772	71,255	12.30
AF442957.1	07	NODE_26_length_1120_cov_7580.522293	1,120	1,120	99.732	3	0	4,439	5,558	1,120	1	0	2052	1,120	100.00	3,571,772	44,352	11.09
AF442957.1	07	NODE_30_length_517_cov_6321.286290	517	517	100	0	0	13,163	13,679	1	517	0	955	517	100.00	3,571,772	16,314	8.83
AF442957.1	07	NODE_7_length_877_cov_4968.174171	877	877	98.86	9	1	13,659	14,535	1	876	0	1563	876	99.89	3,571,772	34,271	10.94
AF442957.1	11	NODE_1_length_8570_cov_3477.689587	8,570	8,575	99.534	31	9	5,964	14,535	8,570	2	0	15605	8,566	99.95	3,021,608	196,062	7.57
AF442957.1	11	NODE_11_length_262_cov_3015.178423	262	262	99.618	1	0	1	262	1	262	2.05E-136	479	262	100.00	3,021,608	3,421	4.32
AF442957.1	11	NODE_12_length_189_cov_2721.035714	189	188	99.468	1	0	14,348	14,535	189	2	2.81E-95	342	188	99.47	3,021,608	1,877	3.30
AF442957.1	11	NODE_18_length_423_cov_3333.383085	423	422	99.526	0	2	5,562	5,981	1	422	0	767	420	99.29	3,021,608	6,949	5.45
AF442957.1	11	NODE_2_length_5986_cov_3560.874853	5,986	5,987	99.449	25	6	1	5,981	5,986	2	0	10866	5,981	99.92	3,021,608	147,074	8.13
AF442957.1	11	NODE_23_length_1597_cov_4397.979695	1,597	1,597	99.812	3	0	3,962	5,558	1,597	1	0	2933	1,597	100.00	3,021,608	36,486	7.56
AF442957.1	11	NODE_24_length_3620_cov_3956.544596	3,620	3,623	99.448	16	4	5,964	9,585	3,620	1	0	6577	3,619	99.97	3,021,608	78,936	7.21
AF442957.1	11	NODE_4_length_4766_cov_4060.396628	4,766	4,769	99.602	14	5	9,583	14,349	1	4,766	0	8696	4,764	99.96	3,021,608	105,437	7.32
AF442957.1	11	NODE_7_length_3636_cov_4122.243154	3,636	3,638	99.258	21	4	251	3,884	1	3,636	0	6564	3,634	99.94	3,021,608	84,137	7.65
AF442957.1	12	NODE_1_length_14538_cov_3786.549397	14,538	14,544	99.491	57	15	1	14,535	14,537	2	0	26432	14,529	99.94	2,446,844	369,807	10.39
AF442957.1	12	NODE_10_length_3855_cov_4361.195618	3,855	3,856	99.3	21	4	1	3,852	3,854	1	0	6966	3,852	99.92	2,446,844	94,041	9.97
AF442957.1	12	NODE_14_length_36_cov_4941.133333	36	36	100	0	0	4,424	4,459	1	36	1.89E-12	67.6	36	100.00	2,446,844	-	-
AF442957.1	12	NODE_17_length_2560_cov_4013.250098	2,560	2,562	99.141	17	5	5,562	8,120	1	2,560	0	4604	2,557	99.88	2,446,844	56,795	9.06
AF442957.1	12	NODE_21_length_678_cov_3644.980213	678	679	98.675	8	1	13,671	14,349	1	678	0	1203	678	100.00	2,446,844	12,196	7.34
AF442957.1	12	NODE_24_length_1120_cov_4864.645132	1,120	1,120	99.732	3	0	4,439	5,558	1,120	1	0	2052	1,120	100.00	2,446,844	27,091	9.89
AF442957.1	12	NODE_3_length_493_cov_4565.942797	493	493	100	0	0	3,952	4,444	1	493	0	911	493	100.00	2,446,844	9,375	7.77
AF442957.1	12	NODE_5_length_189_cov_2921.744048	189	188	99.468	1	0	14,348	14,535	1	188	2.81E-95	342	188	99.47	2,446,844	1,949	4.24
AF442957.1	12	NODE_8_length_5543_cov_4490.119884	5,543	5,546	99.784	7	5	8,136	13,679	1	5,543	0	10170	5,541	99.96	2,446,844	132,408	9.76

Query Sequence Accession *qseqid*	Assembly ID	Subject Sequence ID/Contig ID *sseqid*	Contig Length	Alignment Length *length*	Percentage of Identical Matches *pident*	Mismatch *mismatch*	Gap Opening *gapopen*	Start of Alignment in Query *qstart*	End of Alignment in Query *qend*	Start of Alignment in Subject *sstart*	End of Alignment in Subject *send*	Expect Value *evalue*	Bit Score *bitscore*	True Alignment Length	Percent Coverage	Total Reads	Reads to Align	RPKM
AF442957.1	18	NODE_1_length_10097_cov_3080.803890	10,097	10,102	99.555	34	11	4,439	14,535	1	10,096	0	18395	10,091	99.94	2,909,350	165,299	5.62
AF442957.1	18	NODE_1_length_14537_cov_2701.439327	14,537	14,544	99.498	56	15	1	14,535	14,537	2	0	26437	14,529	99.94	2,909,350	243,682	5.76
AF442957.1	18	NODE_3_length_36_cov_3457.266667	36	36	100	0	0	4,424	4,459	36	1	1.89E-12	67.6	36	100.00	2,909,350	1	0.01
AF442957.1	18	NODE_5_length_3966_cov_3056.022307	3,966	3,968	99.294	22	4	1	3,964	3,966	1	0	7167	3,964	99.95	2,909,350	64,865	5.62
AF442957.1	18	NODE_6_length_501_cov_3241.864583	501	501	100	0	0	3,944	4,444	501	1	0	926	501	100.00	2,909,350	6,464	4.43
AF442957.1	LVE	10213	56	56	100	0	0	12,934	12,989	1	56	3.33E-20	104	56	100.00	32,150,000	-	-
AF442957.1	LVE	10287	56	55	100	0	0	8,005	8,059	56	2	1.20E-19	102	55	98.21	32,150,000	-	-
AF442957.1	LVE	10289	56	56	100	0	0	8,005	8,060	56	1	3.33E-20	104	56	100.00	32,150,000	-	-
AF442957.1	LVE	45085	56	56	100	0	0	1,219	1,274	56	1	3.33E-20	104	56	100.00	32,150,000	-	-
AF442957.1	LVE	72417	56	56	100	0	0	12,031	12,086	1	56	3.33E-20	104	56	100.00	32,150,000	-	-
AF442957.1	LVE	106430	56	56	100	0	0	251	306	1	56	3.33E-20	104	56	100.00	32,150,000	-	-
AF442957.1	LVE	117166	56	56	100	0	0	6,222	6,277	1	56	3.33E-20	104	56	100.00	32,150,000	-	-
AF442957.1	LVE	119348	56	56	100	0	0	7,995	8,050	56	1	3.33E-20	104	56	100.00	32,150,000	-	-
AF442957.1	LVE	128477	56	56	100	0	0	1,173	1,228	56	1	3.33E-20	104	56	100.00	32,150,000	-	-
AF442957.1	LVE	141675	56	56	100	0	0	12,889	12,944	1	56	3.33E-20	104	56	100.00	32,150,000	-	-
AF442957.1	LVE	143377	56	55	100	0	0	8,006	8,060	2	56	1.20E-19	102	55	98.21	32,150,000	-	-
AF442957.1	LVE	144291	56	54	100	0	0	8,006	8,059	1	54	4.30E-19	100	54	96.43	32,150,000	-	-
AF442957.1	LVE	156545	56	54	100	0	0	8,006	8,059	2	55	4.30E-19	100	54	96.43	32,150,000	-	-
AF442957.1	LVE	166288	56	56	100	0	0	3,054	3,109	1	56	3.33E-20	104	56	100.00	32,150,000	-	-
AF442957.1	LVE	355749	60	60	100	0	0	12,970	13,029	60	1	1.99E-22	111	60	100.00	32,150,000	-	-
AF442957.1	LVE	381347	60	60	98.333	1	0	8,007	8,066	60	1	9.25E-21	106	60	100.00	32,150,000	-	-
AF442957.1	LVE	390030	61	61	100	0	0	12,242	12,302	1	61	5.53E-23	113	61	100.00	32,150,000	-	-
AF442957.1	LVE	445956	63	63	100	0	0	7,987	8,049	63	1	4.27E-24	117	63	100.00	32,150,000	-	-
AF442957.1	LVE	449620	63	63	100	0	0	54	116	63	1	4.27E-24	117	63	100.00	32,150,000	-	-
AF442957.1	LVE	451504	63	63	98.413	0	1	7,988	8,049	63	1	7.15E-22	110	62	98.41	32,150,000	-	-
AF442957.1	LVE	453420	63	60	100	0	0	1	60	60	1	1.99E-22	111	60	95.24	32,150,000	-	-
AF442957.1	LVE	469923	64	64	100	0	0	7,996	8,059	1	64	1.19E-24	119	64	100.00	32,150,000	-	-
AF442957.1	LVE	471347	64	64	100	0	0	1,967	2,030	64	1	1.19E-24	119	64	100.00	32,150,000	-	-
AF442957.1	LVE	504986	65	65	100	0	0	4,994	5,058	1	65	3.30E-25	121	65	100.00	32,150,000	-	-
AF442957.1	LVE	536761	67	67	100	0	0	252	318	67	1	2.55E-26	124	67	100.00	32,150,000	-	-
AF442957.1	LVE	574552	70	70	98.571	0	1	12,920	12,988	1	70	9.19E-26	122	69	98.57	32,150,000	-	-
AF442957.1	LVE	575260	70	70	98.571	1	0	12,919	12,988	70	1	2.55E-26	124	70	100.00	32,150,000	-	-
AF442957.1	LVE	575262	70	70	100	0	0	12,919	12,988	70	1	5.49E-28	130	70	100.00	32,150,000	-	-
AF442957.1	LVE	582033	70	70	97.143	1	1	12,920	12,988	70	1	4.27E-24	117	69	98.57	32,150,000	-	-
AF442957.1	LVE	610703	73	73	100	0	0	8,043	8,115	1	73	1.18E-29	135	73	100.00	32,150,000	-	-
AF442957.1	LVE	631184	75	75	100	0	0	10,370	10,444	1	75	9.12E-31	139	75	100.00	32,150,000	-	-
AF442957.1	LVE	641870	76	71	100	0	0	1,976	2,046	1	71	1.53E-28	132	71	93.42	32,150,000	-	-
AF442957.1	LVE	643087	76	76	100	0	0	1,920	1,995	1	76	2.54E-31	141	76	100.00	32,150,000	-	-
AF442957.1	LVE	643237	77	77	100	0	0	8,508	8,584	77	1	7.05E-32	143	77	100.00	32,150,000	-	-
AF442957.1	LVE	644083	77	77	100	0	0	8,452	8,528	77	1	7.05E-32	143	77	100.00	32,150,000	-	-
AF442957.1	LVE	660682	79	79	100	0	0	6,916	6,994	79	1	5.45E-33	147	79	100.00	32,150,000	-	-
AF442957.1	LVE	661578	79	79	100	0	0	4,919	4,997	79	1	5.45E-33	147	79	100.00	32,150,000	-	-

Query Sequence Accession *qseqid*	Assembly ID	Subject Sequence ID/Contig ID *sseqid*	Contig Length	Alignment Length *length*	Percentage of Identical Matches *pident*	Mismatch *mismatch*	Gap Opening *gapopen*	Start of Alignment in Query *qstart*	End of Alignment in Query *qend*	Start of Alignment in Subject *sstart*	End of Alignment in Subject *send*	Expect Value *evalue*	Bit Score *bitscore*	True Alignment Length	Percent Coverage	Total Reads	Reads to Align	RPKM
AF442957.1	LVE	661644	79	79	100	0	0	10,687	10,765	1	79	5.45E-33	147	79	100.00	32,150,000	-	-
AF442957.1	LVE	662032	79	79	100	0	0	10,663	10,741	79	1	5.45E-33	147	79	100.00	32,150,000	-	-
AF442957.1	LVE	674517	81	81	100	0	0	1,941	2,021	1	81	4.21E-34	150	81	100.00	32,150,000	-	-
AF442957.1	LVE	698083	84	84	100	0	0	12,890	12,973	84	1	9.06E-36	156	84	100.00	32,150,000	-	-
AF442957.1	LVE	701237	85	85	100	0	0	4,964	5,048	85	1	2.52E-36	158	85	100.00	32,150,000	-	-
AF442957.1	LVE	703765	85	85	98.824	0	1	12,890	12,973	85	1	4.21E-34	150	84	98.82	32,150,000	-	-
AF442957.1	LVE	710019	87	87	100	0	0	6,940	7,026	87	1	1.95E-37	161	87	100.00	32,150,000	-	-
AF442957.1	LVE	714321	87	87	100	0	0	10,711	10,797	87	1	1.95E-37	161	87	100.00	32,150,000	-	-
AF442957.1	LVE	720426	89	89	100	0	0	8,474	8,562	1	89	1.50E-38	165	89	100.00	32,150,000	-	-
AF442957.1	LVE	725496	90	90	100	0	0	12,935	13,024	90	1	4.18E-39	167	90	100.00	32,150,000	-	-
AF442957.1	LVE	731888	91	91	100	0	0	10,334	10,424	1	91	1.16E-39	169	91	100.00	32,150,000	-	-
AF442957.1	LVE	737736	92	92	100	0	0	8,006	8,097	92	1	3.23E-40	171	92	100.00	32,150,000	-	-
AF442957.1	LVE	746904	95	95	100	0	0	12,248	12,342	95	1	6.95E-42	176	95	100.00	32,150,000	-	-
AF442957.1	LVE	750730	96	99	92.929	4	1	1,967	2,065	96	1	2.54E-31	141	98	102.08	32,150,000	-	-
AF442957.1	LVE	751170	96	96	98.958	1	0	12,934	13,029	1	96	8.99E-41	172	96	100.00	32,150,000	-	-
AF442957.1	LVE	758166	98	98	98.98	0	1	209	305	1	98	2.50E-41	174	97	98.98	32,150,000	-	-
AF442957.1	LVE	760879	98	98	100	0	0	208	305	1	98	1.49E-43	182	98	100.00	32,150,000	-	-
AF442957.1	LVE	762546	99	99	100	0	0	12,032	12,130	1	99	4.15E-44	183	99	100.00	32,150,000	-	-
AF442957.1	LVE	763644	99	100	99	0	1	207	306	1	99	1.93E-42	178	99	100.00	32,150,000	-	-
AF442957.1	LVE	763756	99	100	99	0	1	7,960	8,059	1	99	1.93E-42	178	99	100.00	32,150,000	-	-
AF442957.1	LVE	765030	100	101	99.01	0	1	12,889	12,989	1	100	5.37E-43	180	100	100.00	32,150,000	-	-
AF442957.1	LVE	765770	100	100	99	0	1	12,032	12,130	1	100	1.93E-42	178	99	99.00	32,150,000	-	-
AF442957.1	LVE	765900	100	100	100	0	0	1,174	1,273	1	100	1.16E-44	185	100	100.00	32,150,000	-	-
AF442957.1	LVE	766636	100	101	99.01	0	1	12,031	12,131	1	100	5.37E-43	180	100	100.00	32,150,000	-	-
AF442957.1	LVE	766878	100	100	99	0	1	7,961	8,059	100	1	1.93E-42	178	99	99.00	32,150,000	-	-
AF442957.1	LVE	768783	101	101	100	0	0	3,055	3,155	1	101	3.21E-45	187	101	100.00	32,150,000	-	-
AF442957.1	LVE	769443	101	101	96.04	4	0	14,211	14,311	1	101	1.50E-38	165	101	100.00	32,150,000	-	-
AF442957.1	LVE	769827	101	101	99.01	0	1	1,174	1,273	1	101	5.37E-43	180	100	99.01	32,150,000	-	-
AF442957.1	LVE	769985	101	101	99.01	0	1	4,919	5,018	101	1	5.37E-43	180	100	99.01	32,150,000	-	-
AF442957.1	LVE	771847	101	101	99.01	1	0	6,176	6,276	1	101	1.49E-43	182	101	100.00	32,150,000	-	-
AF442957.1	LVE	771863	101	102	99.02	0	1	1,173	1,274	101	1	1.49E-43	182	101	100.00	32,150,000	-	-
AF442957.1	LVE	772193	101	101	98.02	0	1	7,959	8,059	1	99	2.50E-41	174	100	99.01	32,150,000	-	-
AF442957.1	LVE	772779	102	102	99.02	0	1	3,055	3,155	1	102	1.49E-43	182	101	99.02	32,150,000	-	-
AF442957.1	LVE	773441	102	102	95.098	4	1	14,211	14,311	1	102	7.00E-37	159	101	99.02	32,150,000	-	-
AF442957.1	LVE	775601	102	103	99.029	0	1	3,054	3,156	1	102	4.15E-44	183	102	100.00	32,150,000	-	-
AF442957.1	LVE	775847	102	103	99.029	0	1	7,948	8,050	1	102	4.15E-44	183	102	100.00	32,150,000	-	-
AF442957.1	LVE	775935	102	102	98.039	1	1	6,176	6,276	1	102	6.95E-42	176	101	99.02	32,150,000	-	-
AF442957.1	LVE	776197	102	103	99.029	0	1	10,663	10,765	1	102	4.15E-44	183	102	100.00	32,150,000	-	-
AF442957.1	LVE	776345	102	103	98.058	1	1	6,175	6,277	1	102	1.93E-42	178	102	100.00	32,150,000	-	-
AF442957.1	LVE	777525	103	103	99.029	1	0	7,995	8,097	103	1	1.16E-44	185	103	100.00	32,150,000	-	-
AF442957.1	LVE	779193	103	103	100	0	0	6	108	1	103	2.48E-46	191	103	100.00	32,150,000	-	-
AF442957.1	LVE	780037	103	103	99.029	0	1	12,242	12,343	103	1	4.15E-44	183	102	99.03	32,150,000	-	-

Query Sequence Accession *qseqid*	Assembly ID	Subject Sequence ID/Contig ID *sseqid*	Contig Length	Alignment Length *length*	Percentage of Identical Matches *pident*	Mismatch *mismatch*	Gap Opening *gapopen*	Start of Alignment in Query *qstart*	End of Alignment in Query *qend*	Start of Alignment in Subject *sstart*	End of Alignment in Subject *send*	Expect Value *evalue*	Bit Score *bitscore*	True Alignment Length	Percent Coverage	Total Reads	Reads to Align	RPKM
AF442957.1	LVE	783381	104	104	99.038	1	0	8,012	8,115	1	104	3.21E-45	187	104	100.00	32,150,000	4	0.00
AF442957.1	LVE	785868	105	105	100	0	0	7,327	7,431	1	105	1.92E-47	195	105	100.00	32,150,000	-	-
AF442957.1	LVE	789668	105	106	99.057	0	1	4,943	5,048	1	105	8.93E-46	189	105	100.00	32,150,000	-	-
AF442957.1	LVE	791078	106	106	100	0	0	12,975	13,080	106	1	5.34E-48	196	106	100.00	32,150,000	42	0.01
AF442957.1	LVE	804358	108	108	97.222	0	3	8,012	8,116	1	108	5.37E-43	180	105	97.22	32,150,000	12	0.00
AF442957.1	LVE	812112	110	111	99.099	0	1	12,970	13,080	110	1	1.48E-48	198	110	100.00	32,150,000	188	0.05
AF442957.1	LVE	814685	110	110	99.091	1	0	209	318	110	1	1.48E-48	198	110	100.00	32,150,000	100	0.03
AF442957.1	LVE	818637	111	111	99.099	1	0	8,452	8,562	1	111	4.12E-49	200	111	100.00	32,150,000	166	0.05
AF442957.1	LVE	827385	111	111	100	0	0	4,676	4,786	111	1	8.87E-51	206	111	100.00	32,150,000	156	0.04
AF442957.1	LVE	827411	111	111	98.198	2	0	7,321	7,431	1	111	1.92E-47	195	111	100.00	32,150,000	76	0.02
AF442957.1	LVE	831109	111	111	99.099	1	0	12,248	12,358	111	1	4.12E-49	200	111	100.00	32,150,000	182	0.05
AF442957.1	LVE	837023	111	111	98.198	2	0	2,499	2,609	111	1	1.92E-47	195	111	100.00	32,150,000	132	0.04
AF442957.1	LVE	838339	111	111	99.099	1	0	6,916	7,026	111	1	4.12E-49	200	111	100.00	32,150,000	154	0.04
AF442957.1	LVE	842955	111	111	100	0	0	3,329	3,439	111	1	8.87E-51	206	111	100.00	32,150,000	200	0.06
AF442957.1	LVE	855051	111	108	99.074	1	0	1	108	108	1	1.92E-47	195	108	97.30	32,150,000	136	0.04
AF442957.1	LVE	855111	111	111	99.099	1	0	6	116	111	1	4.12E-49	200	111	100.00	32,150,000	84	0.02
AF442957.1	LVE	865073	111	111	98.198	2	0	4,531	4,641	1	111	1.92E-47	195	111	100.00	32,150,000	34	0.01
AF442957.1	LVE	865075	111	111	99.099	1	0	4,531	4,641	1	111	4.12E-49	200	111	100.00	32,150,000	144	0.04
AF442957.1	LVE	867005	111	111	99.099	1	0	4,948	5,058	111	1	4.12E-49	200	111	100.00	32,150,000	108	0.03
AF442957.1	LVE	877219	111	111	99.099	1	0	3,329	3,439	111	1	4.12E-49	200	111	100.00	32,150,000	28	0.01
AF442957.1	LVE	877939	111	111	99.099	1	0	4,676	4,786	111	1	4.12E-49	200	111	100.00	32,150,000	-	-
AF442957.1	LVE	878765	111	111	99.099	1	0	10,334	10,444	1	111	4.12E-49	200	111	100.00	32,150,000	142	0.04
AF442957.1	LVE	887331	111	111	99.099	1	0	2,499	2,609	1	111	4.12E-49	200	111	100.00	32,150,000	10	0.00
AF442957.1	LVE	891747	111	111	98.198	2	0	11,432	11,542	1	111	1.92E-47	195	111	100.00	32,150,000	14	0.00
AF442957.1	LVE	897297	111	106	99.057	1	0	1,941	2,046	1	106	2.48E-46	191	106	95.50	32,150,000	152	0.04
AF442957.1	LVE	902593	111	111	100	0	0	11,432	11,542	111	1	8.87E-51	206	111	100.00	32,150,000	164	0.05
AF442957.1	LVE	904683	111	111	99.099	1	0	10,314	10,424	1	111	4.12E-49	200	111	100.00	32,150,000	150	0.04
AF442957.1	LVE	907221	111	111	99.099	1	0	10,687	10,797	1	111	4.12E-49	200	111	100.00	32,150,000	110	0.03
AF442957.1	LVE	909431	111	111	99.099	1	0	8,474	8,584	1	111	4.12E-49	200	111	100.00	32,150,000	112	0.03
AF442957.1	LVE	909531	111	111	99.099	1	0	1,920	2,030	1	111	4.12E-49	200	111	100.00	32,150,000	78	0.02
AF442957.1	LVE	931378	127	127	99.213	1	0	62	188	1	127	5.26E-58	230	127	100.00	32,150,000	524	0.13
AF442957.1	LVE	934092	130	130	100	0	0	6,972	7,101	1	130	2.43E-61	241	130	100.00	32,150,000	240	0.06
AF442957.1	LVE	943219	144	144	99.306	1	0	4,587	4,730	144	1	1.87E-67	261	144	100.00	32,150,000	750	0.16
AF442957.1	LVE	950731	162	162	98.765	2	0	6,940	7,101	162	1	8.56E-76	289	162	100.00	32,150,000	680	0.13
AF442957.1	LVE	1165150	186	186	100	0	0	11,488	11,673	186	1	1.80E-92	344	186	100.00	32,150,000	1,356	0.23
AF442957.1	LVE	1183928	188	188	98.936	2	0	11,669	11,856	1	188	3.01E-90	337	188	100.00	32,150,000	1,362	0.23
AF442957.1	LVE	1322655	220	220	100	0	0	12,077	12,296	1	220	2.26E-111	407	220	100.00	32,150,000	1,864	0.26
AF442957.1	LVE	1330589	223	60	95	2	1	10,879	10,937	61	2	7.20E-17	93.5	59	26.46	32,150,000	16	0.01
AF442957.1	LVE	1357997	226	236	100	0	0	11,850	12,085	236	1	2.89E-120	436	236	104.42	32,150,000	2,060	0.27
AF442957.1	LVE	1367963	242	242	99.587	1	0	4,732	4,973	1	242	6.21E-122	442	242	100.00	32,150,000	2,228	0.29
AF442957.1	LVE	1406702	279	279	100	0	0	7,047	7,325	279	1	3.61E-144	516	279	100.00	32,150,000	2,394	0.27
AF442957.1	LVE	1408634	282	282	98.936	3	0	3,102	3,383	282	1	7.81E-141	505	282	100.00	32,150,000	2,646	0.29

Query Sequence Accession *qseqid*	Assembly ID	Subject Sequence ID/Contig ID *sseqid*	Contig Length	Alignment Length *length*	Percentage of Identical Matches *pident*	Mismatch *mismatch*	Gap Opening *gapopen*	Start of Alignment in Query *qstart*	End of Alignment in Query *qend*	Start of Alignment in Subject *sstart*	End of Alignment in Subject *send*	Expect Value *evalue*	Bit Score *bitscore*	True Alignment Length	Percent Coverage	Total Reads	Reads to Align	RPKM
AF442957.1	LVE	1412028	288	288	98.611	4	0	13,978	14,265	1	288	1.68E-142	510	288	100.00	32,150,000	2,622	0.28
AF442957.1	LVE	1426808	328	328	99.085	3	0	10,390	10,717	328	1	2.09E-166	590	328	100.00	32,150,000	3,066	0.29
AF442957.1	LVE	1434156	374	375	98.133	4	3	5,857	6,229	374	1	0	651	372	99.47	32,150,000	3,914	0.32
AF442957.1	LVE	1438448	431	431	100	0	0	8,530	8,960	431	1	0	797	431	100.00	32,150,000	4,398	0.32
AF442957.1	LVE	1439078	444	445	99.326	2	1	8,062	8,506	444	1	0	804	444	100.00	32,150,000	4,658	0.33
AF442957.1	LVE	1441824	543	544	98.162	8	2	2,011	2,553	1	543	0	948	542	99.82	32,150,000	5,582	0.32
AF442957.1	LVE	1442014	554	554	99.097	5	0	2,555	3,108	554	1	0	996	554	100.00	32,150,000	5,970	0.34
AF442957.1	LVE	1442428	577	578	98.789	5	2	7,377	7,953	1	577	0	1027	576	99.83	32,150,000	6,604	0.36
AF442957.1	LVE	1442760	600	600	99.833	1	0	12,344	12,943	600	1	0	1103	600	100.00	32,150,000	6,924	0.36
AF442957.1	LVE	1443268	642	279	99.642	1	0	14,257	14,535	1	279	1.68E-142	510	279	43.46	32,150,000	7,136	0.80
AF442957.1	LVE	1443458	662	663	99.698	0	2	8,965	9,626	1	662	0	1212	661	99.85	32,150,000	7,558	0.35
AF442957.1	LVE	1443510	668	668	100	0	0	9,619	10,286	668	1	0	1234	668	100.00	32,150,000	8,190	0.38
AF442957.1	LVE	1444092	744	744	99.866	1	0	10,743	11,486	744	1	0	1369	744	100.00	32,150,000	8,784	0.37
AF442957.1	LVE	1444118	748	748	99.465	4	0	6,223	6,970	1	748	0	1360	748	100.00	32,150,000	8,672	0.36
AF442957.1	LVE	1444180	754	755	99.735	1	1	1,220	1,974	1	754	0	1382	754	100.00	32,150,000	8,356	0.34
AF442957.1	LVE	1444558	831	831	99.88	1	0	5,004	5,834	831	1	0	1530	831	100.00	32,150,000	10,072	0.38
AF442957.1	LVE	1445068	955	957	99.269	4	3	13,026	13,981	955	1	0	1725	954	99.90	32,150,000	11,818	0.38
AF442957.1	LVE	1445096	964	964	99.896	1	0	264	1,227	1	964	0	1775	964	100.00	32,150,000	11,608	0.37
AF442957.1	LVE	1445642	1,204	1,204	99.668	1	1	3,385	4,585	1,204	1	0	2198	1,203	99.92	32,150,000	14,204	0.37
AF442957.1	LVZ	4622	56	55	100	0	0	8,005	8,059	56	2	7.25E-20	102	55	98.21	24,708,000	-	-
AF442957.1	LVZ	4624	56	56	100	0	0	8,005	8,060	56	1	2.02E-20	104	56	100.00	24,708,000	-	-
AF442957.1	LVZ	4626	56	55	100	0	0	8,005	8,059	2	56	7.25E-20	102	55	98.21	24,708,000	-	-
AF442957.1	LVZ	9455	56	55	100	0	0	14,257	14,311	55	1	7.25E-20	102	55	98.21	24,708,000	-	-
AF442957.1	LVZ	11117	56	56	100	0	0	12,934	12,989	1	56	2.02E-20	104	56	100.00	24,708,000	-	-
AF442957.1	LVZ	29502	56	56	100	0	0	1,219	1,274	56	1	2.02E-20	104	56	100.00	24,708,000	-	-
AF442957.1	LVZ	32480	56	56	100	0	0	4,918	4,973	1	56	2.02E-20	104	56	100.00	24,708,000	-	-
AF442957.1	LVZ	43193	56	56	100	0	0	12,031	12,086	1	56	2.02E-20	104	56	100.00	24,708,000	-	-
AF442957.1	LVZ	61731	56	56	100	0	0	251	306	56	1	2.02E-20	104	56	100.00	24,708,000	-	-
AF442957.1	LVZ	67281	56	56	100	0	0	7,995	8,050	56	1	2.02E-20	104	56	100.00	24,708,000	-	-
AF442957.1	LVZ	79871	56	56	100	0	0	1,173	1,228	56	1	2.02E-20	104	56	100.00	24,708,000	-	-
AF442957.1	LVZ	85417	56	56	100	0	0	12,889	12,944	1	56	2.02E-20	104	56	100.00	24,708,000	-	-
AF442957.1	LVZ	91736	56	55	100	0	0	8,006	8,060	2	56	7.25E-20	102	55	98.21	24,708,000	-	-
AF442957.1	LVZ	97439	56	54	100	0	0	8,006	8,059	2	55	2.61E-19	100	54	96.43	24,708,000	-	-
AF442957.1	LVZ	107352	57	57	100	0	0	11,921	11,977	1	57	5.61E-21	106	57	100.00	24,708,000	-	-
AF442957.1	LVZ	127848	57	57	100	0	0	6,972	7,028	57	1	5.61E-21	106	57	100.00	24,708,000	-	-
AF442957.1	LVZ	167218	58	58	100	0	0	12,927	12,984	58	1	1.56E-21	108	58	100.00	24,708,000	-	-
AF442957.1	LVZ	194163	59	59	100	0	0	12,930	12,988	1	59	4.33E-22	110	59	100.00	24,708,000	-	-
AF442957.1	LVZ	197203	59	59	100	0	0	11,973	12,031	59	1	4.33E-22	110	59	100.00	24,708,000	-	-
AF442957.1	LVZ	199853	59	59	100	0	0	12,923	12,981	1	59	4.33E-22	110	59	100.00	24,708,000	-	-
AF442957.1	LVZ	203425	59	59	100	0	0	2,935	2,993	59	1	4.33E-22	110	59	100.00	24,708,000	-	-
AF442957.1	LVZ	212621	59	59	100	0	0	11,917	11,975	1	59	4.33E-22	110	59	100.00	24,708,000	-	-
AF442957.1	LVZ	219149	60	60	100	0	0	14,280	14,339	60	1	1.20E-22	111	60	100.00	24,708,000	-	-

Query Sequence Accession *qseqid*	Assembly ID	Subject Sequence ID/Contig ID *sseqid*	Contig Length	Alignment Length *length*	Percentage of Identical Matches *pident*	Mismatch *mismatch*	Gap Opening *gapopen*	Start of Alignment in Query *qstart*	End of Alignment in Query *qend*	Start of Alignment in Subject *sstart*	End of Alignment in Subject *send*	Expect Value *evalue*	Bit Score *bitscore*	True Alignment Length	Percent Coverage	Total Reads	Reads to Align	RPKM
AF442957.1	LVZ	222449	60	60	96.667	2	0	4,560	4,619	60	1	2.61E-19	100	60	100.00	24,708,000	-	-
AF442957.1	LVZ	222451	60	60	98.333	1	0	4,560	4,619	60	1	5.61E-21	106	60	100.00	24,708,000	-	-
AF442957.1	LVZ	232022	60	60	95	3	0	4,560	4,619	1	60	1.21E-17	95.3	60	100.00	24,708,000	-	-
AF442957.1	LVZ	233870	60	60	96.667	2	0	4,560	4,619	60	1	2.61E-19	100	60	100.00	24,708,000	-	-
AF442957.1	LVZ	245161	61	61	98.361	1	0	2,995	3,055	61	1	1.56E-21	108	61	100.00	24,708,000	-	-
AF442957.1	LVZ	257837	61	61	100	0	0	2,939	2,999	1	61	3.35E-23	113	61	100.00	24,708,000	-	-
AF442957.1	LVZ	258187	61	61	98.361	1	0	8,006	8,066	61	1	1.56E-21	108	61	100.00	24,708,000	-	-
AF442957.1	LVZ	263131	62	62	100	0	0	4,524	4,585	62	1	9.31E-24	115	62	100.00	24,708,000	-	-
AF442957.1	LVZ	270773	62	62	100	0	0	8,530	8,591	62	1	9.31E-24	115	62	100.00	24,708,000	-	-
AF442957.1	LVZ	275852	62	62	100	0	0	8,474	8,535	1	62	9.31E-24	115	62	100.00	24,708,000	-	-
AF442957.1	LVZ	282661	63	63	100	0	0	11,965	12,027	1	63	2.59E-24	117	63	100.00	24,708,000	-	-
AF442957.1	LVZ	287501	63	63	98.413	1	0	3,001	3,063	1	63	1.20E-22	111	63	100.00	24,708,000	-	-
AF442957.1	LVZ	293223	64	64	100	0	0	7,996	8,059	1	64	7.20E-25	119	64	100.00	24,708,000	-	-
AF442957.1	LVZ	294867	64	64	100	0	0	1,932	1,995	64	1	7.20E-25	119	64	100.00	24,708,000	-	-
AF442957.1	LVZ	299623	64	64	100	0	0	11,977	12,040	64	1	7.20E-25	119	64	100.00	24,708,000	-	-
AF442957.1	LVZ	305906	64	64	100	0	0	1,967	2,030	64	1	7.20E-25	119	64	100.00	24,708,000	-	-
AF442957.1	LVZ	313118	65	65	100	0	0	11,986	12,050	1	65	2.00E-25	121	65	100.00	24,708,000	-	-
AF442957.1	LVZ	315270	65	65	100	0	0	12,021	12,085	65	1	2.00E-25	121	65	100.00	24,708,000	-	-
AF442957.1	LVZ	315456	65	65	100	0	0	12,042	12,106	1	65	2.00E-25	121	65	100.00	24,708,000	-	-
AF442957.1	LVZ	315660	65	65	96.923	2	0	2,991	3,055	65	1	4.33E-22	110	65	100.00	24,708,000	-	-
AF442957.1	LVZ	317442	65	65	100	0	0	12,032	12,096	65	1	2.00E-25	121	65	100.00	24,708,000	-	-
AF442957.1	LVZ	319092	65	65	100	0	0	3,055	3,119	65	1	2.00E-25	121	65	100.00	24,708,000	-	-
AF442957.1	LVZ	331952	67	67	100	0	0	1,920	1,986	1	67	1.55E-26	124	67	100.00	24,708,000	-	-
AF442957.1	LVZ	333510	67	67	98.507	1	0	4,548	4,614	1	67	7.20E-25	119	67	100.00	24,708,000	-	-
AF442957.1	LVZ	335838	67	67	100	0	0	1,976	2,042	1	67	1.55E-26	124	67	100.00	24,708,000	-	-
AF442957.1	LVZ	337322	67	68	98.529	0	1	239	306	1	67	7.20E-25	119	67	100.00	24,708,000	-	-
AF442957.1	LVZ	340024	67	67	98.507	1	0	4,604	4,670	67	1	7.20E-25	119	67	100.00	24,708,000	-	-
AF442957.1	LVZ	346908	68	68	100	0	0	12,910	12,977	68	1	4.30E-27	126	68	100.00	24,708,000	-	-
AF442957.1	LVZ	353153	69	69	100	0	0	8,460	8,528	69	1	1.20E-27	128	69	100.00	24,708,000	-	-
AF442957.1	LVZ	358903	69	69	100	0	0	8,516	8,584	1	69	1.20E-27	128	69	100.00	24,708,000	-	-
AF442957.1	LVZ	361274	70	70	100	0	0	4,509	4,578	1	70	3.33E-28	130	70	100.00	24,708,000	-	-
AF442957.1	LVZ	361290	70	70	100	0	0	7,349	7,418	1	70	3.33E-28	130	70	100.00	24,708,000	-	-
AF442957.1	LVZ	361292	70	70	100	0	0	7,364	7,433	70	1	3.33E-28	130	70	100.00	24,708,000	-	-
AF442957.1	LVZ	369036	71	71	100	0	0	1,174	1,244	1	71	9.25E-29	132	71	100.00	24,708,000	-	-
AF442957.1	LVZ	375948	72	72	100	0	0	6,923	6,994	1	72	2.57E-29	134	72	100.00	24,708,000	-	-
AF442957.1	LVZ	379292	72	72	98.611	1	0	4,587	4,658	72	1	1.20E-27	128	72	100.00	24,708,000	-	-
AF442957.1	LVZ	380366	72	72	100	0	0	252	323	72	1	2.57E-29	134	72	100.00	24,708,000	-	-
AF442957.1	LVZ	381060	72	72	100	0	0	4,531	4,602	1	72	2.57E-29	134	72	100.00	24,708,000	-	-
AF442957.1	LVZ	381589	72	72	100	0	0	11,041	11,112	1	72	2.57E-29	134	72	100.00	24,708,000	-	-
AF442957.1	LVZ	383660	73	73	100	0	0	12,966	13,038	1	73	7.15E-30	135	73	100.00	24,708,000	-	-
AF442957.1	LVZ	383820	73	73	100	0	0	11,002	11,074	73	1	7.15E-30	135	73	100.00	24,708,000	-	-
AF442957.1	LVZ	388268	73	73	100	0	0	11,058	11,130	73	1	7.15E-30	135	73	100.00	24,708,000	-	-

Query Sequence Accession *qseqid*	Assembly ID	Subject Sequence ID/Contig ID *sseqid*	Contig Length	Alignment Length *length*	Percentage of Identical Matches *pident*	Mismatch *mismatch*	Gap Opening *gapopen*	Start of Alignment in Query *qstart*	End of Alignment in Query *qend*	Start of Alignment in Subject *sstart*	End of Alignment in Subject *send*	Expect Value *evalue*	Bit Score *bitscore*	True Alignment Length	Percent Coverage	Total Reads	Reads to Align	RPKM
AF442957.1	LVZ	398445	75	75	98.667	0	1	12,032	12,105	75	1	9.25E-29	132	74	98.67	24,708,000	-	-
AF442957.1	LVZ	400317	75	75	100	0	0	12,890	12,964	1	75	5.53E-31	139	75	100.00	24,708,000	-	-
AF442957.1	LVZ	401477	75	75	100	0	0	11,945	12,019	1	75	5.53E-31	139	75	100.00	24,708,000	-	-
AF442957.1	LVZ	405506	76	76	100	0	0	11,020	11,095	76	1	1.54E-31	141	76	100.00	24,708,000	-	-
AF442957.1	LVZ	407905	77	77	100	0	0	11,923	11,999	77	1	4.27E-32	143	77	100.00	24,708,000	-	-
AF442957.1	LVZ	409919	77	77	98.701	1	0	4,565	4,641	1	77	1.99E-30	137	77	100.00	24,708,000	-	-
AF442957.1	LVZ	412109	77	77	100	0	0	7,327	7,403	1	77	4.27E-32	143	77	100.00	24,708,000	-	-
AF442957.1	LVZ	413227	78	78	100	0	0	14,257	14,334	78	1	1.19E-32	145	78	100.00	24,708,000	-	-
AF442957.1	LVZ	424819	80	80	100	0	0	11,996	12,075	80	1	9.18E-34	148	80	100.00	24,708,000	-	-
AF442957.1	LVZ	428059	81	81	100	0	0	1,941	2,021	1	81	2.55E-34	150	81	100.00	24,708,000	-	-
AF442957.1	LVZ	428171	81	81	98.765	1	0	7,379	7,459	81	1	1.19E-32	145	81	100.00	24,708,000	-	-
AF442957.1	LVZ	428173	81	81	97.531	2	0	7,379	7,459	81	1	5.53E-31	139	81	100.00	24,708,000	-	-
AF442957.1	LVZ	428219	81	81	100	0	0	7,379	7,459	1	81	2.55E-34	150	81	100.00	24,708,000	-	-
AF442957.1	LVZ	428655	81	81	98.765	1	0	7,379	7,459	1	81	1.19E-32	145	81	100.00	24,708,000	-	-
AF442957.1	LVZ	433790	82	82	100	0	0	269	350	1	82	7.10E-35	152	82	100.00	24,708,000	-	-
AF442957.1	LVZ	436774	83	83	100	0	0	11,889	11,971	83	1	1.97E-35	154	83	100.00	24,708,000	-	-
AF442957.1	LVZ	440168	84	84	100	0	0	1,190	1,273	84	1	5.49E-36	156	84	100.00	24,708,000	-	-
AF442957.1	LVZ	444332	85	85	98.824	1	0	7,349	7,433	1	85	7.10E-35	152	85	100.00	24,708,000	-	-
AF442957.1	LVZ	444774	85	85	98.824	1	0	7,405	7,489	85	1	7.10E-35	152	85	100.00	24,708,000	-	-
AF442957.1	LVZ	445614	85	85	100	0	0	7,405	7,489	85	1	1.53E-36	158	85	100.00	24,708,000	-	-
AF442957.1	LVZ	447458	86	86	100	0	0	12,935	13,020	86	1	4.24E-37	159	86	100.00	24,708,000	-	-
AF442957.1	LVZ	450880	87	87	100	0	0	6,940	7,026	87	1	1.18E-37	161	87	100.00	24,708,000	-	-
AF442957.1	LVZ	453622	87	88	98.864	0	1	207	294	1	87	5.49E-36	156	87	100.00	24,708,000	-	-
AF442957.1	LVZ	460129	89	89	100	0	0	7,961	8,049	89	1	9.12E-39	165	89	100.00	24,708,000	-	-
AF442957.1	LVZ	463350	90	90	100	0	0	8,481	8,570	90	1	2.54E-39	167	90	100.00	24,708,000	-	-
AF442957.1	LVZ	463472	91	91	100	0	0	3,065	3,155	1	91	7.05E-40	169	91	100.00	24,708,000	-	-
AF442957.1	LVZ	464976	91	91	98.901	1	0	11,996	12,086	91	1	3.28E-38	163	91	100.00	24,708,000	-	-
AF442957.1	LVZ	466204	91	91	100	0	0	215	305	1	91	7.05E-40	169	91	100.00	24,708,000	-	-
AF442957.1	LVZ	474330	94	94	100	0	0	1,134	1,227	1	94	1.52E-41	174	94	100.00	24,708,000	-	-
AF442957.1	LVZ	478009	96	99	92.929	4	1	1,967	2,065	96	1	1.54E-31	141	98	102.08	24,708,000	-	-
AF442957.1	LVZ	478179	96	96	100	0	0	4,468	4,563	1	96	1.17E-42	178	96	100.00	24,708,000	-	-
AF442957.1	LVZ	478929	96	96	98.958	1	0	6,182	6,277	96	1	5.45E-41	172	96	100.00	24,708,000	-	-
AF442957.1	LVZ	486279	99	99	98.99	0	1	7,961	8,058	99	1	4.21E-42	176	98	98.99	24,708,000	-	-
AF442957.1	LVZ	486537	99	99	98.99	0	1	208	305	1	99	4.21E-42	176	98	98.99	24,708,000	-	-
AF442957.1	LVZ	487393	99	100	99	0	1	7,960	8,059	99	1	1.17E-42	178	99	100.00	24,708,000	-	-
AF442957.1	LVZ	487687	100	100	95	4	1	14,212	14,310	1	100	5.49E-36	156	99	99.00	24,708,000	-	-
AF442957.1	LVZ	487731	100	101	99.01	0	1	12,889	12,989	1	100	3.26E-43	180	100	100.00	24,708,000	-	-
AF442957.1	LVZ	488151	100	100	99	0	1	12,890	12,988	1	100	1.17E-42	178	99	99.00	24,708,000	-	-
AF442957.1	LVZ	488295	100	100	100	0	0	4,919	5,018	100	1	7.00E-45	185	100	100.00	24,708,000	-	-
AF442957.1	LVZ	488969	100	101	99.01	0	1	12,031	12,131	1	100	3.26E-43	180	100	100.00	24,708,000	-	-
AF442957.1	LVZ	490002	101	101	96.04	4	0	14,211	14,311	101	1	9.12E-39	165	101	100.00	24,708,000	-	-
AF442957.1	LVZ	490140	101	101	94.059	4	1	14,212	14,310	1	101	7.10E-35	152	100	99.01	24,708,000	-	-

Query Sequence Accession *qseqid*	Assembly ID	Subject Sequence ID/Contig ID *sseqid*	Contig Length	Alignment Length *length*	Percentage of Identical Matches *pident*	Mismatch *mismatch*	Gap Opening *gapopen*	Start of Alignment in Query *qstart*	End of Alignment in Query *qend*	Start of Alignment in Subject *sstart*	End of Alignment in Subject *send*	Expect Value *evalue*	Bit Score *bitscore*	True Alignment Length	Percent Coverage	Total Reads	Reads to Align	RPKM
AF442957.1	LVZ	490470	101	101	99.01	0	1	4,919	5,018	1	101	3.26E-43	180	100	99.01	24,708,000	-	-
AF442957.1	LVZ	490984	101	102	99.02	0	1	4,918	5,019	101	1	9.05E-44	182	101	100.00	24,708,000	-	-
AF442957.1	LVZ	491844	101	101	99.01	1	0	3,009	3,109	101	1	9.05E-44	182	101	100.00	24,708,000	-	-
AF442957.1	LVZ	491882	101	101	99.01	0	1	7,950	8,049	1	101	3.26E-43	180	100	99.01	24,708,000	-	-
AF442957.1	LVZ	491928	101	101	99.01	1	0	2,945	3,045	1	101	9.05E-44	182	101	100.00	24,708,000	-	-
AF442957.1	LVZ	492192	101	101	99.01	0	1	1,174	1,273	1	101	3.26E-43	180	100	99.01	24,708,000	-	-
AF442957.1	LVZ	492196	101	102	99.02	0	1	1,173	1,274	101	1	9.05E-44	182	101	100.00	24,708,000	-	-
AF442957.1	LVZ	492644	102	102	99.02	0	1	3,055	3,155	1	102	9.05E-44	182	101	99.02	24,708,000	-	-
AF442957.1	LVZ	494678	102	103	98.058	1	1	6,175	6,277	1	102	1.17E-42	178	102	100.00	24,708,000	-	-
AF442957.1	LVZ	494742	102	103	99.029	0	1	7,948	8,050	1	102	2.52E-44	183	102	100.00	24,708,000	-	-
AF442957.1	LVZ	495018	102	103	99.029	0	1	10,663	10,765	1	102	2.52E-44	183	102	100.00	24,708,000	-	-
AF442957.1	LVZ	496636	103	79	98.734	0	1	1	79	78	1	5.53E-31	139	78	75.73	24,708,000	-	-
AF442957.1	LVZ	497902	103	103	100	0	0	10,663	10,765	1	103	1.50E-46	191	103	100.00	24,708,000	-	-
AF442957.1	LVZ	498648	104	104	100	0	0	12,221	12,324	1	104	4.18E-47	193	104	100.00	24,708,000	-	-
AF442957.1	LVZ	499914	104	104	99.038	1	0	8,012	8,115	1	104	1.95E-45	187	104	100.00	24,708,000	-	-
AF442957.1	LVZ	500746	104	104	99.038	1	0	6,126	6,229	1	104	1.95E-45	187	104	100.00	24,708,000	2	0.00
AF442957.1	LVZ	501024	104	79	100	0	0	1	79	26	104	3.30E-33	147	79	75.96	24,708,000	-	-
AF442957.1	LVZ	502193	105	105	100	0	0	2,945	3,049	1	105	1.16E-47	195	105	100.00	24,708,000	-	-
AF442957.1	LVZ	503133	105	106	99.057	0	1	6,923	7,028	105	1	5.41E-46	189	105	100.00	24,708,000	-	-
AF442957.1	LVZ	505149	106	106	98.113	2	0	4,509	4,614	106	1	7.00E-45	185	106	100.00	24,708,000	-	-
AF442957.1	LVZ	506353	106	106	98.113	2	0	4,565	4,670	106	1	7.00E-45	185	106	100.00	24,708,000	-	-
AF442957.1	LVZ	509154	107	110	97.273	0	1	3,944	4,053	1	107	2.52E-44	183	109	101.87	24,708,000	4	0.00
AF442957.1	LVZ	513773	108	108	97.222	0	3	8,012	8,116	1	108	3.26E-43	180	105	97.22	24,708,000	12	0.00
AF442957.1	LVZ	517337	109	109	99.083	1	0	7,950	8,058	109	1	3.23E-48	196	109	100.00	24,708,000	64	0.02
AF442957.1	LVZ	520455	110	110	100	0	0	8,006	8,115	1	110	1.93E-50	204	110	100.00	24,708,000	236	0.09
AF442957.1	LVZ	520621	110	110	100	0	0	3,944	4,053	1	110	1.93E-50	204	110	100.00	24,708,000	214	0.08
AF442957.1	LVZ	525833	111	111	99.099	1	0	6,916	7,026	111	1	2.50E-49	200	111	100.00	24,708,000	150	0.05
AF442957.1	LVZ	527243	111	111	100	0	0	4,224	4,334	1	111	5.37E-51	206	111	100.00	24,708,000	254	0.09
AF442957.1	LVZ	531499	111	111	99.099	1	0	11,867	11,977	1	111	2.50E-49	200	111	100.00	24,708,000	192	0.07
AF442957.1	LVZ	533427	111	111	99.099	1	0	1,932	2,042	111	1	2.50E-49	200	111	100.00	24,708,000	110	0.04
AF442957.1	LVZ	533449	111	111	99.099	1	0	11,002	11,112	111	1	2.50E-49	200	111	100.00	24,708,000	90	0.03
AF442957.1	LVZ	534809	111	111	99.099	1	0	1,134	1,244	1	111	2.50E-49	200	111	100.00	24,708,000	166	0.06
AF442957.1	LVZ	535539	111	111	98.198	2	0	8,325	8,435	111	1	1.16E-47	195	111	100.00	24,708,000	6	0.00
AF442957.1	LVZ	539197	111	111	99.099	1	0	4,468	4,578	1	111	2.50E-49	200	111	100.00	24,708,000	100	0.04
AF442957.1	LVZ	539325	111	111	98.198	2	0	7,308	7,418	1	111	1.16E-47	195	111	100.00	24,708,000	86	0.03
AF442957.1	LVZ	542979	111	111	99.099	1	0	11,889	11,999	111	1	2.50E-49	200	111	100.00	24,708,000	108	0.04
AF442957.1	LVZ	544605	111	111	99.099	1	0	2,939	3,049	1	111	2.50E-49	200	111	100.00	24,708,000	14	0.01
AF442957.1	LVZ	544773	111	111	99.099	1	0	8,460	8,570	111	1	2.50E-49	200	111	100.00	24,708,000	48	0.02
AF442957.1	LVZ	547191	111	111	99.099	1	0	11,917	12,027	1	111	2.50E-49	200	111	100.00	24,708,000	64	0.02
AF442957.1	LVZ	551079	111	111	99.099	1	0	9,281	9,391	111	1	2.50E-49	200	111	100.00	24,708,000	10	0.00
AF442957.1	LVZ	552639	111	111	99.099	1	0	4,224	4,334	111	1	2.50E-49	200	111	100.00	24,708,000	8	0.00
AF442957.1	LVZ	552987	111	112	98.214	1	1	12,927	13,038	111	1	1.16E-47	195	111	100.00	24,708,000	30	0.01

Query Sequence Accession *qseqid*	Assembly ID	Subject Sequence ID/Contig ID *sseqid*	Contig Length	Alignment Length *length*	Percentage of Identical Matches *pident*	Mismatch *mismatch*	Gap Opening *gapopen*	Start of Alignment in Query *qstart*	End of Alignment in Query *qend*	Start of Alignment in Subject *sstart*	End of Alignment in Subject *send*	Expect Value *evalue*	Bit Score *bitscore*	True Alignment Length	Percent Coverage	Total Reads	Reads to Align	RPKM
AF442957.1	LVZ	554489	111	111	99.099	1	0	12,221	12,331	111	1	2.50E-49	200	111	100.00	24,708,000	146	0.05
AF442957.1	LVZ	554811	111	111	99.099	1	0	11,921	12,031	1	111	2.50E-49	200	111	100.00	24,708,000	98	0.04
AF442957.1	LVZ	557309	111	111	98.198	2	0	4,531	4,641	1	111	1.16E-47	195	111	100.00	24,708,000	74	0.03
AF442957.1	LVZ	558989	111	111	99.099	1	0	12,910	13,020	1	111	2.50E-49	200	111	100.00	24,708,000	162	0.06
AF442957.1	LVZ	559981	111	112	98.214	1	1	239	350	1	111	1.16E-47	195	111	100.00	24,708,000	68	0.02
AF442957.1	LVZ	560705	111	111	98.198	2	0	2,935	3,045	111	1	1.16E-47	195	111	100.00	24,708,000	8	0.00
AF442957.1	LVZ	561473	111	111	100	0	0	9,281	9,391	111	1	5.37E-51	206	111	100.00	24,708,000	204	0.07
AF442957.1	LVZ	562447	111	111	98.198	2	0	6,126	6,236	1	111	1.16E-47	195	111	100.00	24,708,000	188	0.07
AF442957.1	LVZ	566293	111	111	99.099	1	0	11,965	12,075	1	111	2.50E-49	200	111	100.00	24,708,000	122	0.04
AF442957.1	LVZ	566387	111	111	98.198	2	0	3,009	3,119	111	1	1.16E-47	195	111	100.00	24,708,000	98	0.04
AF442957.1	LVZ	568315	111	111	99.099	1	0	213	323	111	1	2.50E-49	200	111	100.00	24,708,000	144	0.05
AF442957.1	LVZ	568379	111	111	99.099	1	0	11,020	11,130	111	1	2.50E-49	200	111	100.00	24,708,000	114	0.04
AF442957.1	LVZ	569315	111	111	99.099	1	0	8,325	8,435	111	1	2.50E-49	200	111	100.00	24,708,000	122	0.04
AF442957.1	LVZ	572007	111	111	98.198	2	0	4,548	4,658	111	1	1.16E-47	195	111	100.00	24,708,000	96	0.04
AF442957.1	LVZ	576685	111	106	99.057	1	0	1,941	2,046	1	106	1.50E-46	191	106	95.50	24,708,000	124	0.05
AF442957.1	LVZ	578019	111	111	99.099	1	0	11,986	12,096	111	1	2.50E-49	200	111	100.00	24,708,000	176	0.06
AF442957.1	LVZ	579689	111	111	99.099	1	0	11,041	11,151	111	1	2.50E-49	200	111	100.00	24,708,000	244	0.09
AF442957.1	LVZ	583839	111	111	99.099	1	0	8,481	8,591	111	1	2.50E-49	200	111	100.00	24,708,000	68	0.02
AF442957.1	LVZ	584663	111	111	99.099	1	0	8,474	8,584	1	111	2.50E-49	200	111	100.00	24,708,000	114	0.04
AF442957.1	LVZ	585133	111	111	99.099	1	0	1,920	2,030	1	111	2.50E-49	200	111	100.00	24,708,000	48	0.02
AF442957.1	LVZ	600240	128	128	100	0	0	6,974	7,101	128	1	1.91E-60	237	128	100.00	24,708,000	254	0.08
AF442957.1	LVZ	602922	134	134	99.254	1	0	8,381	8,514	1	134	4.09E-62	243	134	100.00	24,708,000	546	0.16
AF442957.1	LVZ	611674	162	162	98.765	2	0	6,940	7,101	162	1	5.19E-76	289	162	100.00	24,708,000	722	0.18
AF442957.1	LVZ	624040	166	166	99.398	1	0	11,691	11,856	1	166	6.66E-80	302	166	100.00	24,708,000	992	0.24
AF442957.1	LVZ	694534	176	176	99.432	1	0	25	200	1	176	1.84E-85	320	176	100.00	24,708,000	892	0.21
AF442957.1	LVZ	765444	190	191	98.953	1	1	8,189	8,379	190	1	1.41E-91	340	190	100.00	24,708,000	1,306	0.28
AF442957.1	LVZ	787421	197	197	100	0	0	3,999	4,195	1	197	8.38E-99	364	197	100.00	24,708,000	1,308	0.27
AF442957.1	LVZ	795775	199	199	100	0	0	12,077	12,275	1	199	6.48E-100	368	199	100.00	24,708,000	1,394	0.28
AF442957.1	LVZ	806877	204	44	95.455	2	0	10,879	10,922	160	203	2.04E-10	71.3	44	21.57	24,708,000	14	0.01
AF442957.1	LVZ	817543	209	209	99.522	1	0	14,285	14,493	209	1	8.32E-104	381	209	100.00	24,708,000	1,502	0.29
AF442957.1	LVZ	819353	209	209	93.301	14	0	14,285	14,493	209	1	3.98E-82	309	209	100.00	24,708,000	34	0.01
AF442957.1	LVZ	847463	228	228	100	0	0	7,047	7,274	228	1	4.90E-116	422	228	100.00	24,708,000	1,604	0.28
AF442957.1	LVZ	858153	239	239	100	0	0	9,908	10,146	1	239	3.76E-122	442	239	100.00	24,708,000	1,992	0.34
AF442957.1	LVZ	861635	243	243	100	0	0	4,280	4,522	243	1	2.25E-124	449	243	100.00	24,708,000	1,792	0.30
AF442957.1	LVZ	875581	270	270	100	0	0	9,619	9,888	1	270	2.20E-139	499	270	100.00	24,708,000	2,438	0.37
AF442957.1	LVZ	880517	286	287	97.909	3	3	5,896	6,180	286	1	1.02E-137	494	284	99.30	24,708,000	2,396	0.34
AF442957.1	LVZ	881471	890	291	99.313	0	2	9,337	9,626	1	290	3.63E-147	525	289	32.47	24,708,000	2,510	0.35
AF442957.1	LVZ	882001	293	266	92.857	15	3	12,716	12,977	266	1	2.31E-104	383	263	89.76	24,708,000	500	0.08
AF442957.1	LVZ	888893	346	346	100	0	0	10,711	11,056	1	346	0	640	346	100.00	24,708,000	2,958	0.35
AF442957.1	LVZ	889785	357	357	99.72	1	0	4,616	4,972	1	357	0	654	357	100.00	24,708,000	3,380	0.38
AF442957.1	LVZ	890651	371	371	100	0	0	8,965	9,335	1	371	0	686	371	100.00	24,708,000	3,370	0.37
AF442957.1	LVZ	892755	420	420	100	0	0	488	907	1	420	0	776	420	100.00	24,708,000	4,252	0.41

Query Sequence Accession *qseqid*	Assembly ID	Subject Sequence ID/Contig ID *sseqid*	Contig Length	Alignment Length *length*	Percentage of Identical Matches *pident*	Mismatch *mismatch*	Gap Opening *gapopen*	Start of Alignment in Query *qstart*	End of Alignment in Query *qend*	Start of Alignment in Subject *sstart*	End of Alignment in Subject *send*	Expect Value *evalue*	Bit Score *bitscore*	True Alignment Length	Percent Coverage	Total Reads	Reads to Align	RPKM
AF442957.1	LVZ	892901	424	424	100	0	0	8,537	8,960	1	424	0	784	424	100.00	24,708,000	4,056	0.39
AF442957.1	LVZ	894719	497	497	99.396	3	0	10,221	10,717	1	497	0	902	497	100.00	24,708,000	5,002	0.41
AF442957.1	LVZ	895883	568	569	98.77	5	2	7,435	8,002	1	568	0	1011	567	99.82	24,708,000	5,908	0.42
AF442957.1	LVZ	895989	577	577	99.827	1	0	11,097	11,673	1	577	0	1061	577	100.00	24,708,000	6,112	0.43
AF442957.1	LVZ	896465	618	618	99.838	1	0	12,326	12,943	1	618	0	1136	618	100.00	24,708,000	6,352	0.42
AF442957.1	LVZ	897367	743	740	99.324	5	0	3,101	3,840	1	740	0	1339	740	99.60	24,708,000	7,920	0.43
AF442957.1	LVZ	897387	748	748	99.465	4	0	6,223	6,970	1	748	0	1360	748	100.00	24,708,000	8,382	0.45
AF442957.1	LVZ	897425	754	755	99.735	1	1	1,220	1,974	1	754	0	1382	754	100.00	24,708,000	7,942	0.43
AF442957.1	LVZ	897861	870	870	99.885	1	0	4,965	5,834	1	870	0	1602	870	100.00	24,708,000	9,782	0.46
AF442957.1	LVZ	898189	979	980	98.469	13	2	2,011	2,989	1	979	0	1725	978	99.90	24,708,000	10,420	0.43
AF442957.1	LVZ	898467	1,135	1,147	81.604	118	55	5,431	6,496	1,135	1	0	863	1,092	96.21	24,708,000	224	0.01
AF442957.1	LVZ	898641	1,281	1,283	99.143	8	3	12,984	14,265	1	1,281	0	2305	1,280	99.92	24,708,000	13,632	0.43
AF442957.1	LVZ	898767	1,432	1,454	84.525	154	53	12,930	14,334	1	1,432	0	1373	1,401	97.84	24,708,000	584	0.02
AF442957.1	LVZ	898931	1,822	1,831	85.309	237	25	6,702	8,514	1	1,817	0	1862	1,806	99.12	24,708,000	846	0.02
AF442957.1	LVZ	899029	2,249	2,254	85.093	284	37	2,645	4,863	2,237	1	0	2254	2,217	98.58	24,708,000	2,324	0.04
AF442957.1	LVZ	899123	3,079	2,705	85.213	350	45	1	2,675	385	3,069	0	2734	2,660	86.39	24,708,000	1,442	0.02
AF442957.1	LVZ	899141	4,005	4,032	83.259	557	85	8,737	12,700	9	3,990	0	3598	3,947	98.55	24,708,000	2,146	0.02
AF442957.1	RSE	23164	56	55	100	0	0	8,006	8,060	2	56	1.66E-20	102	55	98.21	47,636,000	-	-
AF442957.1	RSE	24660	56	54	100	0	0	8,006	8,059	2	55	5.97E-20	100	54	96.43	47,636,000	-	-
AF442957.1	RSE	32609	57	57	100	0	0	10,388	10,444	57	1	1.28E-21	106	57	100.00	47,636,000	-	-
AF442957.1	RSE	59249	60	60	96.667	2	0	4,560	4,619	60	1	5.97E-20	100	60	100.00	47,636,000	-	-
AF442957.1	RSE	59251	60	60	98.333	1	0	4,560	4,619	60	1	1.28E-21	106	60	100.00	47,636,000	-	-
AF442957.1	RSE	62167	60	60	98.333	1	0	2,799	2,858	1	60	1.28E-21	106	60	100.00	47,636,000	-	-
AF442957.1	RSE	62253	60	60	95	3	0	4,560	4,619	1	60	2.78E-18	95.3	60	100.00	47,636,000	-	-
AF442957.1	RSE	62807	60	60	96.667	2	0	4,560	4,619	60	1	5.97E-20	100	60	100.00	47,636,000	-	-
AF442957.1	RSE	67530	61	61	100	0	0	8,006	8,066	1	61	7.67E-24	113	61	100.00	47,636,000	-	-
AF442957.1	RSE	80102	64	64	100	0	0	7,027	7,090	64	1	1.65E-25	119	64	100.00	47,636,000	-	-
AF442957.1	RSE	80679	64	64	100	0	0	1,932	1,995	64	1	1.65E-25	119	64	100.00	47,636,000	-	-
AF442957.1	RSE	81351	64	64	100	0	0	11,220	11,283	64	1	1.65E-25	119	64	100.00	47,636,000	-	-
AF442957.1	RSE	82120	64	64	100	0	0	6,971	7,034	64	1	1.65E-25	119	64	100.00	47,636,000	18	0.01
AF442957.1	RSE	83811	64	64	100	0	0	11,164	11,227	1	64	1.65E-25	119	64	100.00	47,636,000	-	-
AF442957.1	RSE	106785	72	73	97.26	0	2	7,610	7,681	1	72	1.27E-26	122	71	98.61	47,636,000	-	-
AF442957.1	RSE	108025	73	73	98.63	1	0	2,478	2,550	73	1	7.62E-29	130	73	100.00	47,636,000	240	0.07
AF442957.1	RSE	108583	73	73	98.63	1	0	2,534	2,606	1	73	7.62E-29	130	73	100.00	47,636,000	340	0.10
AF442957.1	RSE	108887	73	73	100	0	0	10,370	10,442	1	73	1.64E-30	135	73	100.00	47,636,000	120	0.03
AF442957.1	RSE	114109	76	76	100	0	0	11,199	11,274	1	76	3.52E-32	141	76	100.00	47,636,000	50	0.01
AF442957.1	RSE	115684	77	77	100	0	0	4,509	4,585	77	1	9.79E-33	143	77	100.00	47,636,000	160	0.04
AF442957.1	RSE	116064	77	77	98.701	1	0	4,565	4,641	1	77	4.55E-31	137	77	100.00	47,636,000	44	0.01
AF442957.1	RSE	122077	81	81	100	0	0	11,173	11,253	1	81	5.85E-35	150	81	100.00	47,636,000	88	0.02
AF442957.1	RSE	125403	84	84	98.81	1	0	4,531	4,614	1	84	5.85E-35	150	84	100.00	47,636,000	284	0.07
AF442957.1	RSE	125661	84	84	98.81	1	0	4,587	4,670	84	1	5.85E-35	150	84	100.00	47,636,000	374	0.09
AF442957.1	RSE	126160	84	85	98.824	0	1	7,975	8,059	84	1	5.85E-35	150	84	100.00	47,636,000	36	0.01

Query Sequence Accession *qseqid*	Assembly ID	Subject Sequence ID/Contig ID *sseqid*	Contig Length	Alignment Length *length*	Percentage of Identical Matches *pident*	Mismatch *mismatch*	Gap Opening *gapopen*	Start of Alignment in Query *qstart*	End of Alignment in Query *qend*	Start of Alignment in Subject *sstart*	End of Alignment in Subject *send*	Expect Value *evalue*	Bit Score *bitscore*	True Alignment Length	Percent Coverage	Total Reads	Reads to Align	RPKM
AF442957.1	RSE	127354	86	86	100	0	0	7,975	8,060	86	1	9.72E-38	159	86	100.00	47,636,000	638	0.16
AF442957.1	RSE	133195	91	91	100	0	0	10,334	10,424	1	91	1.61E-40	169	91	100.00	47,636,000	160	0.04
AF442957.1	RSE	134989	93	93	97.849	2	0	2,496	2,588	93	1	2.70E-38	161	93	100.00	47,636,000	512	0.12
AF442957.1	RSE	135859	94	95	96.842	1	2	7,627	7,720	94	1	3.49E-37	158	93	98.94	47,636,000	630	0.14
AF442957.1	RSE	135967	94	95	97.895	0	2	7,571	7,664	94	1	7.51E-39	163	93	98.94	47,636,000	622	0.14
AF442957.1	RSE	136385	95	95	100	0	0	4,918	5,012	95	1	9.65E-43	176	95	100.00	47,636,000	980	0.22
AF442957.1	RSE	141571	101	101	96.04	4	0	14,211	14,311	101	1	2.09E-39	165	101	100.00	47,636,000	582	0.12
AF442957.1	RSE	141909	101	102	99.02	0	1	4,918	5,019	101	1	2.07E-44	182	101	100.00	47,636,000	496	0.10
AF442957.1	RSE	142373	101	102	99.02	0	1	1,173	1,274	101	1	2.07E-44	182	101	100.00	47,636,000	18	0.00
AF442957.1	RSE	142567	102	102	100	0	0	6,980	7,081	1	102	1.24E-46	189	102	100.00	47,636,000	546	0.11
AF442957.1	RSE	142577	102	102	95.098	4	1	14,211	14,311	102	1	9.72E-38	159	101	99.02	47,636,000	56	0.01
AF442957.1	RSE	143061	102	102	100	0	0	1,941	2,042	102	1	1.24E-46	189	102	100.00	47,636,000	1,014	0.21
AF442957.1	RSE	143317	102	103	98.058	1	1	6,175	6,277	1	102	2.68E-43	178	102	100.00	47,636,000	40	0.01
AF442957.1	RSE	143425	102	102	100	0	0	1,173	1,274	102	1	1.24E-46	189	102	100.00	47,636,000	518	0.11
AF442957.1	RSE	144325	103	103	99.029	1	0	6,175	6,277	1	103	1.60E-45	185	103	100.00	47,636,000	504	0.10
AF442957.1	RSE	145251	104	104	100	0	0	4,965	5,068	1	104	9.58E-48	193	104	100.00	47,636,000	978	0.20
AF442957.1	RSE	146135	105	105	100	0	0	8,012	8,116	105	1	2.66E-48	195	105	100.00	47,636,000	780	0.16
AF442957.1	RSE	146677	106	106	99.057	1	0	2,748	2,853	1	106	3.45E-47	191	106	100.00	47,636,000	1,268	0.25
AF442957.1	RSE	146891	106	106	98.113	2	0	4,509	4,614	106	1	1.60E-45	185	106	100.00	47,636,000	460	0.09
AF442957.1	RSE	146905	106	104	99.038	1	0	2,804	2,907	1	104	4.46E-46	187	104	98.11	47,636,000	1,876	0.38
AF442957.1	RSE	147327	106	106	98.113	2	0	4,565	4,670	106	1	1.60E-45	185	106	100.00	47,636,000	536	0.11
AF442957.1	RSE	149785	108	108	97.222	0	3	8,012	8,116	1	108	7.46E-44	180	105	97.22	47,636,000	24	0.00
AF442957.1	RSE	153305	111	111	99.099	1	0	6,971	7,081	1	111	5.73E-50	200	111	100.00	47,636,000	372	0.07
AF442957.1	RSE	154063	111	111	97.297	3	0	2,478	2,588	111	1	1.24E-46	189	111	100.00	47,636,000	906	0.17
AF442957.1	RSE	155847	111	111	99.099	1	0	1,932	2,042	111	1	5.73E-50	200	111	100.00	47,636,000	718	0.14
AF442957.1	RSE	158883	111	111	99.099	1	0	11,164	11,274	1	111	5.73E-50	200	111	100.00	47,636,000	336	0.06
AF442957.1	RSE	159525	111	111	97.297	3	0	2,496	2,606	1	111	1.24E-46	189	111	100.00	47,636,000	792	0.15
AF442957.1	RSE	160657	111	111	99.099	1	0	6,410	6,520	111	1	5.73E-50	200	111	100.00	47,636,000	76	0.01
AF442957.1	RSE	161791	111	111	98.198	2	0	2,748	2,858	111	1	2.66E-48	195	111	100.00	47,636,000	374	0.07
AF442957.1	RSE	163105	111	111	98.198	2	0	4,531	4,641	1	111	2.66E-48	195	111	100.00	47,636,000	720	0.14
AF442957.1	RSE	163325	111	111	100	0	0	10,838	10,948	111	1	1.23E-51	206	111	100.00	47,636,000	1,658	0.31
AF442957.1	RSE	163681	111	111	100	0	0	11,493	11,603	111	1	1.23E-51	206	111	100.00	47,636,000	1,188	0.22
AF442957.1	RSE	163901	111	111	99.099	1	0	3,329	3,439	111	1	5.73E-50	200	111	100.00	47,636,000	204	0.04
AF442957.1	RSE	164433	111	111	99.099	1	0	11,143	11,253	1	111	5.73E-50	200	111	100.00	47,636,000	514	0.10
AF442957.1	RSE	166195	111	111	99.099	1	0	4,958	5,068	1	111	5.73E-50	200	111	100.00	47,636,000	474	0.09
AF442957.1	RSE	167053	111	111	99.099	1	0	10,334	10,444	1	111	5.73E-50	200	111	100.00	47,636,000	376	0.07
AF442957.1	RSE	167575	111	112	97.321	1	2	7,571	7,681	1	111	1.24E-46	189	110	99.10	47,636,000	480	0.09
AF442957.1	RSE	168509	111	111	99.099	1	0	6,980	7,090	1	111	5.73E-50	200	111	100.00	47,636,000	428	0.08
AF442957.1	RSE	169047	111	106	99.057	1	0	1,941	2,046	1	106	3.45E-47	191	106	95.50	47,636,000	550	0.11
AF442957.1	RSE	169419	111	111	99.099	1	0	10,838	10,948	1	111	5.73E-50	200	111	100.00	47,636,000	72	0.01
AF442957.1	RSE	169763	111	111	100	0	0	3,329	3,439	111	1	1.23E-51	206	111	100.00	47,636,000	1,128	0.21
AF442957.1	RSE	169871	111	111	99.099	1	0	11,173	11,283	111	1	5.73E-50	200	111	100.00	47,636,000	798	0.15

Query Sequence Accession *qseqid*	Assembly ID	Subject Sequence ID/Contig ID *sseqid*	Contig Length	Alignment Length *length*	Percentage of Identical Matches *pident*	Mismatch *mismatch*	Gap Opening *gapopen*	Start of Alignment in Query *qstart*	End of Alignment in Query *qend*	Start of Alignment in Subject *sstart*	End of Alignment in Subject *send*	Expect Value *evalue*	Bit Score *bitscore*	True Alignment Length	Percent Coverage	Total Reads	Reads to Align	RPKM
AF442957.1	RSE	169921	111	109	98.165	2	0	2,799	2,907	111	3	3.45E-47	191	109	98.20	47,636,000	176	0.03
AF442957.1	RSE	169997	111	111	99.099	1	0	10,314	10,424	1	111	5.73E-50	200	111	100.00	47,636,000	306	0.06
AF442957.1	RSE	170091	111	111	100	0	0	6,410	6,520	1	111	1.23E-51	206	111	100.00	47,636,000	524	0.10
AF442957.1	RSE	170635	111	112	96.429	2	2	7,610	7,720	111	1	5.77E-45	183	110	99.10	47,636,000	520	0.10
AF442957.1	RSE	174825	121	121	97.521	3	0	2,935	3,055	121	1	3.42E-52	207	121	100.00	47,636,000	188	0.03
AF442957.1	RSE	174865	121	121	99.174	1	0	2,935	3,055	121	1	1.58E-55	219	121	100.00	47,636,000	1,928	0.33
AF442957.1	RSE	175775	125	125	100	0	0	11,549	11,673	1	125	2.03E-59	231	125	100.00	47,636,000	1,316	0.22
AF442957.1	RSE	177513	135	135	97.778	3	0	2,855	2,989	1	135	5.64E-60	233	135	100.00	47,636,000	2,046	0.32
AF442957.1	RSE	178399	142	142	98.592	2	0	10,390	10,531	142	1	1.56E-65	252	142	100.00	47,636,000	2,188	0.32
AF442957.1	RSE	178687	144	144	97.222	4	0	10,388	10,531	144	1	2.61E-63	244	144	100.00	47,636,000	144	0.02
AF442957.1	RSE	195883	192	192	88.542	22	0	1,664	1,855	1	192	5.64E-60	233	192	100.00	47,636,000	32	0.00
AF442957.1	RSE	202533	217	217	91.244	19	0	10,790	11,006	217	1	7.10E-79	296	217	100.00	47,636,000	60	0.01
AF442957.1	RSE	203441	222	220	91.818	6	9	14,028	14,239	222	7	7.10E-79	296	211	95.05	47,636,000	46	0.00
AF442957.1	RSE	205915	239	239	100	0	0	7,036	7,274	1	239	8.62E-123	442	239	100.00	47,636,000	5,020	0.44
AF442957.1	RSE	206253	242	242	100	0	0	6,223	6,464	1	242	1.85E-124	448	242	100.00	47,636,000	1,246	0.11
AF442957.1	RSE	207351	251	248	100	0	0	2,555	2,802	248	1	8.55E-128	459	248	98.80	47,636,000	6,546	0.55
AF442957.1	RSE	209109	270	250	89.2	27	0	3,157	3,406	270	21	7.05E-84	313	250	92.59	47,636,000	628	0.05
AF442957.1	RSE	211013	299	299	99.666	1	0	7,327	7,625	1	299	1.77E-154	547	299	100.00	47,636,000	4,228	0.30
AF442957.1	RSE	211267	304	304	100	0	0	10,894	11,197	304	1	6.34E-159	562	304	100.00	47,636,000	6,418	0.44
AF442957.1	RSE	211505	308	308	98.701	4	0	7,666	7,973	1	308	1.77E-154	547	308	100.00	47,636,000	4,328	0.29
AF442957.1	RSE	212025	319	319	99.687	1	0	11,229	11,547	1	319	1.35E-165	584	319	100.00	47,636,000	7,664	0.50
AF442957.1	RSE	213527	357	357	99.72	1	0	4,616	4,972	1	357	0	654	357	100.00	47,636,000	8,718	0.51
AF442957.1	RSE	214013	373	374	98.128	4	3	5,858	6,229	1	373	0	649	371	99.46	47,636,000	5,892	0.33
AF442957.1	RSE	214267	383	383	98.956	4	0	3,001	3,383	1	383	0	686	383	100.00	47,636,000	8,400	0.46
AF442957.1	RSE	215061	416	416	99.279	3	0	10,477	10,892	1	416	0	752	416	100.00	47,636,000	11,268	0.57
AF442957.1	RSE	215917	459	456	99.561	2	0	3,385	3,840	459	4	0	832	456	99.35	47,636,000	10,306	0.47
AF442957.1	RSE	216863	536	537	98.138	8	2	1,997	2,532	1	536	0	935	535	99.81	47,636,000	19,284	0.75
AF442957.1	RSE	217065	560	560	99.286	4	0	6,466	7,025	1	560	0	1013	560	100.00	47,636,000	6,006	0.23
AF442957.1	RSE	217947	719	719	100	0	0	3,845	4,563	1	719	0	1328	719	100.00	47,636,000	12,754	0.37
AF442957.1	RSE	218111	765	279	99.642	1	0	14,257	14,535	1	279	2.33E-143	510	279	36.47	47,636,000	5,262	0.40
AF442957.1	RSE	218117	766	767	99.739	1	1	1,220	1,986	1	766	0	1404	766	100.00	47,636,000	20,022	0.55
AF442957.1	RSE	218359	843	843	99.881	1	0	5,014	5,856	843	1	0	1552	843	100.00	47,636,000	19,456	0.48
AF442957.1	RSE	218947	1,316	1,227	99.837	2	0	1	1,227	1,227	1	0	2255	1,227	93.24	47,636,000	11,366	0.19
AF442957.1	RSE	219177	2,306	2,308	99.783	2	3	8,062	10,368	2,306	1	0	4231	2,305	99.96	47,636,000	38,758	0.35
AF442957.1	RSE	219205	2,596	2,598	99.5	10	3	11,669	14,265	1	2,596	0	4723	2,595	99.96	47,636,000	42,734	0.35
AF442957.1	RSZ	660	56	55	100	0	0	8,005	8,059	56	2	1.05E-20	102	55	98.21	36,782,000	-	-
AF442957.1	RSZ	662	56	56	100	0	0	8,005	8,060	56	1	2.93E-21	104	56	100.00	36,782,000	-	-
AF442957.1	RSZ	9773	56	56	100	0	0	6,222	6,277	1	56	2.93E-21	104	56	100.00	36,782,000	-	-
AF442957.1	RSZ	12850	56	55	100	0	0	8,006	8,060	2	56	1.05E-20	102	55	98.21	36,782,000	-	-
AF442957.1	RSZ	13597	56	54	100	0	0	8,006	8,059	2	55	3.79E-20	100	54	96.43	36,782,000	-	-
AF442957.1	RSZ	33153	60	60	96.667	2	0	4,560	4,619	60	1	3.79E-20	100	60	100.00	36,782,000	-	-
AF442957.1	RSZ	33155	60	60	98.333	1	0	4,560	4,619	60	1	8.14E-22	106	60	100.00	36,782,000	-	-

Query Sequence Accession *qseqid*	Assembly ID	Subject Sequence ID/Contig ID *sseqid*	Contig Length	Alignment Length *length*	Percentage of Identical Matches *pident*	Mismatch *mismatch*	Gap Opening *gapopen*	Start of Alignment in Query *qstart*	End of Alignment in Query *qend*	Start of Alignment in Subject *sstart*	End of Alignment in Subject *send*	Expect Value *evalue*	Bit Score *bitscore*	True Alignment Length	Percent Coverage	Total Reads	Reads to Align	RPKM
AF442957.1	RSZ	34769	60	60	95	3	0	4,560	4,619	1	60	1.76E-18	95.3	60	100.00	36,782,000	-	-
AF442957.1	RSZ	35119	60	60	96.667	2	0	4,560	4,619	60	1	3.79E-20	100	60	100.00	36,782,000	-	-
AF442957.1	RSZ	39052	61	61	98.361	1	0	8,006	8,066	61	1	2.26E-22	108	61	100.00	36,782,000	-	-
AF442957.1	RSZ	45515	64	64	100	0	0	1,932	1,995	64	1	1.05E-25	119	64	100.00	36,782,000	-	-
AF442957.1	RSZ	65983	77	77	100	0	0	4,509	4,585	77	1	6.21E-33	143	77	100.00	36,782,000	44	0.02
AF442957.1	RSZ	66221	77	77	98.701	1	0	4,565	4,641	1	77	2.89E-31	137	77	100.00	36,782,000	12	0.00
AF442957.1	RSZ	69677	81	81	98.765	1	0	7,379	7,459	81	1	1.73E-33	145	81	100.00	36,782,000	36	0.01
AF442957.1	RSZ	69955	81	81	100	0	0	7,379	7,459	1	81	3.71E-35	150	81	100.00	36,782,000	110	0.04
AF442957.1	RSZ	72067	84	84	98.81	1	0	4,531	4,614	1	84	3.71E-35	150	84	100.00	36,782,000	96	0.03
AF442957.1	RSZ	72233	84	84	98.81	1	0	4,587	4,670	84	1	3.71E-35	150	84	100.00	36,782,000	146	0.05
AF442957.1	RSZ	72569	85	85	100	0	0	7,975	8,059	1	85	2.22E-37	158	85	100.00	36,782,000	182	0.06
AF442957.1	RSZ	81505	99	100	99	0	1	7,960	8,059	99	1	1.70E-43	178	99	100.00	36,782,000	138	0.04
AF442957.1	RSZ	81655	100	101	99.01	0	1	12,889	12,989	1	100	4.73E-44	180	100	100.00	36,782,000	18	0.00
AF442957.1	RSZ	81679	100	101	99.01	0	1	12,031	12,131	100	1	4.73E-44	180	100	100.00	36,782,000	16	0.00
AF442957.1	RSZ	82169	101	101	96.04	4	0	14,211	14,311	101	1	1.32E-39	165	101	100.00	36,782,000	200	0.05
AF442957.1	RSZ	82199	101	101	100	0	0	12,889	12,989	1	101	2.83E-46	187	101	100.00	36,782,000	284	0.08
AF442957.1	RSZ	82535	101	101	100	0	0	12,031	12,131	1	101	2.83E-46	187	101	100.00	36,782,000	404	0.11
AF442957.1	RSZ	82671	101	101	99.01	1	0	6,176	6,276	1	101	1.32E-44	182	101	100.00	36,782,000	132	0.04
AF442957.1	RSZ	82837	102	102	95.098	4	1	14,211	14,311	102	1	6.16E-38	159	101	99.02	36,782,000	18	0.00
AF442957.1	RSZ	83179	102	102	100	0	0	1,941	2,042	102	1	7.86E-47	189	102	100.00	36,782,000	254	0.07
AF442957.1	RSZ	83327	102	103	98.058	1	1	6,175	6,277	1	102	1.70E-43	178	102	100.00	36,782,000	40	0.01
AF442957.1	RSZ	83335	102	102	98.039	1	1	6,176	6,276	102	1	6.12E-43	176	101	99.02	36,782,000	6	0.00
AF442957.1	RSZ	84539	104	104	99.038	1	0	8,012	8,115	1	104	2.83E-46	187	104	100.00	36,782,000	112	0.03
AF442957.1	RSZ	85637	106	106	98.113	2	0	4,509	4,614	106	1	1.02E-45	185	106	100.00	36,782,000	162	0.04
AF442957.1	RSZ	85901	106	106	98.113	2	0	4,565	4,670	106	1	1.02E-45	185	106	100.00	36,782,000	232	0.06
AF442957.1	RSZ	87437	108	108	97.222	0	3	8,012	8,116	1	108	4.73E-44	180	105	97.22	36,782,000	14	0.00
AF442957.1	RSZ	88721	110	110	100	0	0	8,006	8,115	1	110	2.81E-51	204	110	100.00	36,782,000	182	0.04
AF442957.1	RSZ	89281	111	111	98.198	2	0	6,585	6,695	1	111	1.69E-48	195	111	100.00	36,782,000	28	0.01
AF442957.1	RSZ	89283	111	111	99.099	1	0	6,585	6,695	1	111	3.63E-50	200	111	100.00	36,782,000	156	0.04
AF442957.1	RSZ	89369	111	106	99.057	1	0	1,941	2,046	1	106	2.19E-47	191	106	95.50	36,782,000	220	0.06
AF442957.1	RSZ	91255	111	111	99.099	1	0	1,932	2,042	111	1	3.63E-50	200	111	100.00	36,782,000	286	0.07
AF442957.1	RSZ	95739	111	111	98.198	2	0	4,531	4,641	1	111	1.69E-48	195	111	100.00	36,782,000	264	0.06
AF442957.1	RSZ	128737	376	376	100	0	0	11,710	12,085	376	1	0	695	376	100.00	36,782,000	3,302	0.24
AF442957.1	RSZ	129371	417	417	99.041	4	0	6,223	6,639	417	1	0	749	417	100.00	36,782,000	1,358	0.09
AF442957.1	RSZ	130721	569	570	98.772	5	2	7,405	7,973	569	1	0	1013	568	99.82	36,782,000	3,664	0.17
AF442957.1	RSZ	131101	667	279	99.642	1	0	14,257	14,535	667	389	1.48E-143	510	279	41.83	36,782,000	1,794	0.17
AF442957.1	RSZ	131219	719	719	100	0	0	3,845	4,563	1	719	0	1328	719	100.00	36,782,000	4,790	0.18
AF442957.1	RSZ	131389	793	793	100	0	0	6,641	7,433	1	793	0	1465	793	100.00	36,782,000	5,090	0.17
AF442957.1	RSZ	131497	867	867	99.885	1	0	12,077	12,943	1	867	0	1596	867	100.00	36,782,000	7,898	0.25
AF442957.1	RSZ	131815	1,330	1,332	99.174	8	3	12,935	14,265	1,330	1	0	2396	1,329	99.92	36,782,000	4,362	0.09
AF442957.1	RSZ	131869	1,615	1,616	99.443	6	3	4,616	6,229	1,615	1	0	2931	1,613	99.88	36,782,000	14,370	0.24
AF442957.1	RSZ	131905	1,847	1,845	98.862	19	2	1,997	3,840	1,847	4	0	3290	1,843	99.78	36,782,000	22,324	0.33

Query Sequence Accession *qseqid*	Assembly ID	Subject Sequence ID/Contig ID *sseqid*	Contig Length	Alignment Length *length*	Percentage of Identical Matches *pident*	Mismatch *mismatch*	Gap Opening *gapopen*	Start of Alignment in Query *qstart*	End of Alignment in Query *qend*	Start of Alignment in Subject *sstart*	End of Alignment in Subject *send*	Expect Value *evalue*	Bit Score *bitscore*	True Alignment Length	Percent Coverage	Total Reads	Reads to Align	RPKM
AF442957.1	RSZ	131927	2,076	1,986	99.799	3	1	1	1,986	92	2,076	0	3644	1,985	95.62	36,782,000	12,628	0.17
AF442957.1	RSZ	131973	3,611	3,613	99.751	6	3	8,062	11,673	3,611	1	0	6619	3,610	99.97	36,782,000	28,482	0.21
